# Supplementary material for: A DNA assembly toolkit to unlock the CRISPR/Cas9 potential for metabolic engineering
Source: Commun Biol. 2023 Aug 18;6:858. doi: 10.1038/s42003-023-05202-5 (PMC10439232; doi:10.1038/s42003-023-05202-5)
Supplement: Supplementary file 2 — Supplementary Material [file 42003_2023_5202_MOESM2_ESM.pdf]

Imperial College London
BioMediCan Inc

YaliCraft
manual

- 2023 -

Table of Contents

Supplementary Note 1. ASSEMBLY OF DNA CONSTRUCTS 3
1.1 Introduction 3
1.2 (Lvl0 Module) Assembly of basic parts 5
1.3 (Exp Module) Assembly of overexpression constructs 8
1.3.1 Assembly of empty Lvl1 vector 11
1.3.2 Assembly of empty Lvl2 vector 13
1.3.3 Assembly of single TU on the Lvl1 14
1.3.4 Assembly of several TUs on the Lvl2 16
1.4 (Del Module) Assembly of gene disruption construct 17
1.5 (Int Module) Exchange of homology arms 20
1.5.1 Exchange of homology arms between Lvl1 and Lvl2 20
1.5.2 Transfer of homology arms from pDel-series to Lvl1 and Lvl2 21
1.6 (MEx Module) Excision of yeast selectable marker 22
1.7 (Cas Module) Re-encoding Cas9-helper 23
1.8 (Pro Module) Introduction of new promoters 25

Supplementary Note 2. YARROWIA ENGINEERING 26
2.1 Functional characterization of standard integration loci 27
2.2 Marker-free CRISPR/Cas9-mediated integration 29
2.3 Marker-based integration 30
2.4 Combining marker-based and Cas9-mediated integration 30
2.5 Selectable marker recovery 31
2.6 Promoter library screening 32

Supplementary Note 3. PROTOCOLS 34
3.1 GG reaction using BsaI/BsmBI/BpiI/AarI with thermal inactivation 34
3.2 GG reaction using AarI without thermal inactivation 34
3.3 GG reaction using LguI with thermal inactivation 35
3.4 pDelUK-RG plasmid isolation 35
3.5 *EcoRed* competent culture preparation 36
3.6 *EcoCre* competent culture preparation 36
3.7 *EcoRed* and *EcoCre* electroporation 37
3.8 *Y. lipolytica* transformation 37
3.9 *Y. lipolytica* colony PCR 38

Supplementary Note 4. APPENDIX 39
4.1 Media recipes 39
4.2 List of plasmids 40
4.3 List of strains 42
4.4 List of primers 42

5. SUPPLEMENTARY REFERENCES 44

Supplementary Note 1. ASSEMBLY OF DNA CONSTRUCTS

1.1 Introduction

YaliCraft is a multi-modular toolkit for constructing integrative vectors and Cas9-helper plasmids for metabolic engineering in *Y. lipolytica* (Supplementary Figure 1). Six modules (Lvl0, Pro, Exp, Del, Int, MEx) are intended for the assembly of integrative constructs that are targeted to the *Y. lipolytica* genome. The Cas Module is designed to assemble episomal Cas9-helpers. There is also variation in the order in which modules can be utilised, leading to different integrative constructs for both gene overexpression and inactivation or both. These two types of modifications were selected because they are the most common in metabolic engineering. Nevertheless, the toolkit can be expanded with new modules and easily adapted for tasks such as introducing point mutations, short insertions/deletions or for the substitution of promoters upstream of native genes.

Most of the modules, except the Exp Module, include single-step manipulation of the construct. Modules Lvl0, Pro, Exp, Del, and Int are based on Golden Gate (GG) reactions. Each GG step requires only two days to get constructs ready either for the next modification step or for yeast transformation (Supplementary Table 1). Due to the addition of fluorescent reporters and use of alternating antibiotic resistance markers, it is possible to select transformants with high efficiency by phenotypic selection alone. Chloramphenicol (Cm), spectinomycin (Sp), ampicillin (Ap) and kanamycin (Km) resistance are reserved for Lvl0-, Lvl1-, Lvl2-, and pDel-series, respectively. Additionally, the essential advantage of GG is that it does not require sequence verification after each assembly step – DNA only needs to be sequenced once after initial cloning of a PCR amplified fragment. The sequences of all overhangs required for GG assembly in this toolkit are summarized in Supplementary Table 2.

The Cas and MEx Modules are based on *EcoRed* and *EcoCre E. coli* strains respectively. Both strains contained the modified λ prophage for temperature-inducible expression of λ-Red genes (*gam, bet, exo*) or *cre* gene of phage P1, respectively. Application of *in vivo* recombination does not require any *in vitro* step, and therefore, significantly accelerates and simplifies the workflow.

As a result, all procedures in this toolkit are based on the application of uncut plasmids and do not require a laborious gel purification step. At the same time, we highly recommend carrying out restriction analysis for accurate qualitative and quantitative analysis of constructs obtained following each assembly step. It is important to mention that incorrect spectrophotometric estimation of DNA concentration is the most common cause of DNA assembly failures.

The *YaliCraft* system is capable of growth and can be extended with new modules. It can be modified for overexpression of more than 3 transcriptional units (TUs) at a single integration locus, supplied with alternative yeast selectable markers or expanded to include additional integration loci. The toolkit also contains a set of empty vectors with Zeta sequences as integration flanks, which are commonly used for random integration[1]. Random integration requires active non-homologous end-joining system, and therefore, can be preferentially applied on the strains with Ku70+ genetic background. Furthermore, the promoters, genes, terminators, yeast markers and homologous arms from this system are fully compatible with the current most widely used *Y. lipolytica* GG system[2, 3, 4].

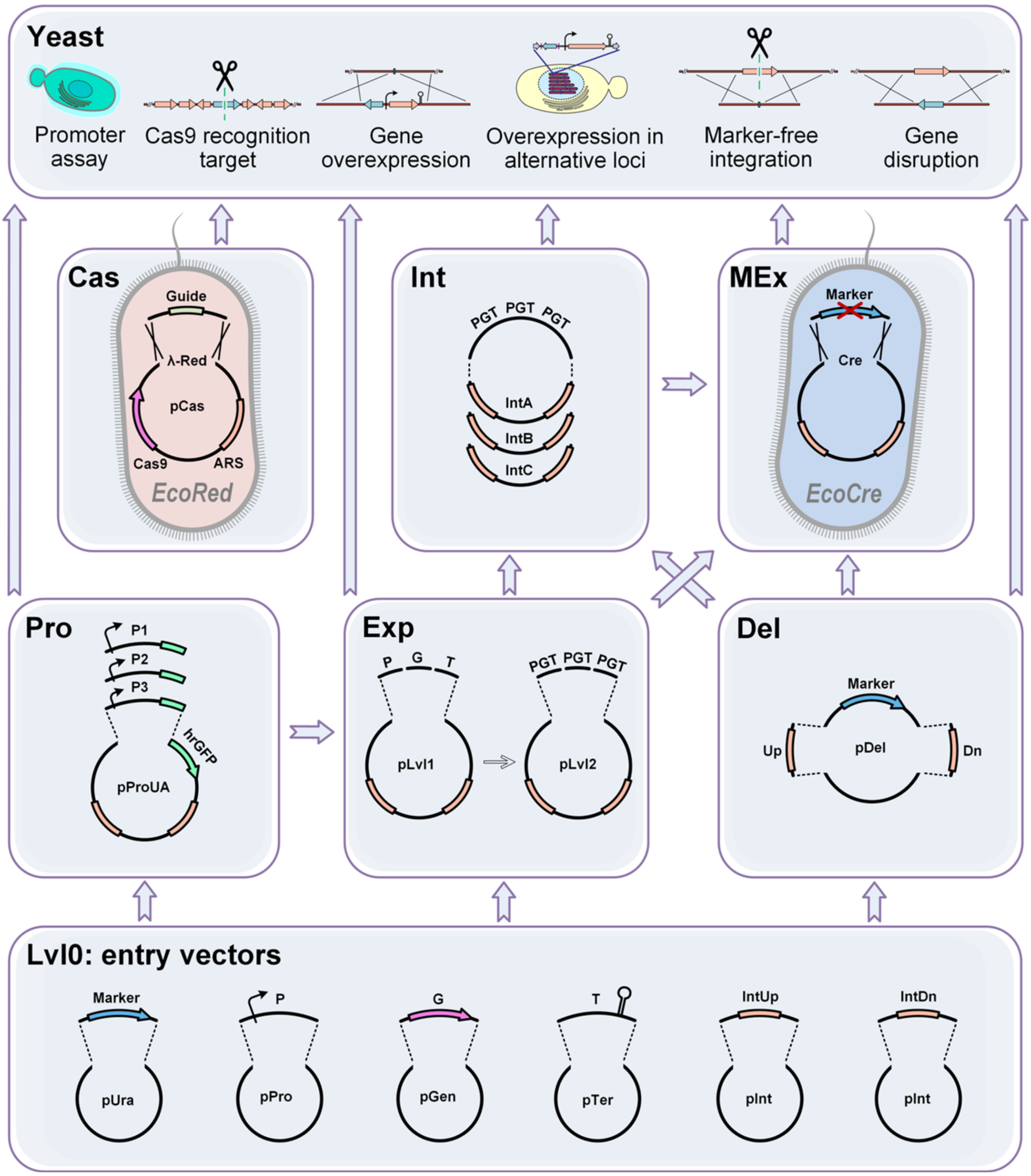

Supplementary Figure 1 Blueprint of the *YaliCraft* system. Seven modules are shown. Lvl0 Module: single parts in entry vectors. Exp Module: assembly of overexpression constructs. Pro Module: assembly and screening of new promoters. Del Module: assembly of disruption constructs. Int Module: changing integration loci by homology arms exchange. MEx Module: assembly of marker-free constructs by selectable marker excision. Cas Module: redirection of Cas9-helper to new genome loci. The arrows between modules indicate different orders in which they can be applied to enable variable genome engineering techniques as shown on the top panel - Yeast.

Supplementary Table 1 Time required for single GG step (days)

Assembly method	Day 1	Day 2	Total time spent
**GG assembly**	● (opt) PCR and purification<br>● GG assembly or exchange<br>● *E. coli* transformation	● Culturing and miniprep<br>● Restriction analysis*<br>● Yeast transformation or next GG step	2 days

* - sequence verification is recommended only for DNA fragments amplified by PCR before assembling

Supplementary Table 2 Overhangs used for different types of GG assemblies in *YaliCraft*

GG assembly	GGA enzyme	Bb*		IntUp		Marker		Pro (PGT)		Gen (PGT)		Ter (PGT)		IntDn		Bb
**Lvl0**	BsmBI		**TCGG**												**GACC**	
**preLvl**	BsaI		**GCCT**		**AGGT**		**ACGG**						**GAGT**		**TGCG**	
**Empty Lvl**	BpiI						**ACGG**						**GAGT**			
**Lvl1**	BsaI						**ACGG**		**AATG**		**TCTA**		**GAGT**			
**Lvl2.2**	BsmBI						**CTGA**		**CCAA**		**GATG**					
**Lvl2.3**	BsmBI						**CTGA**		**CCAA**		**GATG**		**GTTC**			
**Pro**	BpiI						**ACGG**		**AATG**							
**Del**	LguI		**CAG**		**CCA**								**TAA**		**GGA**	
**Int**	AarI				**AGGT**								**GAGT**			

* – Blue columns show the genetic elements in the order they appear in assembled Lvl1 or Lvl2 plasmids, while green columns show overhangs that are used in corresponding assembly types to combine these elements. Detailed description of each assembly type is provided in the following sections. Genetic elements are abbreviated as follows. Bb, plasmid backbone. IntUp and IntDn, upstream and downstream homologous arms, respectively. Marker, *Y. lipolytica* selectable marker. Pro, promoter. Gen, gene. Ter, terminator. PGT, transcription unit.

1.2 (Lvl0 Module) Assembly of basic parts

During the first step, genetic elements are cloned and stored separately in the Lvl0 Module. Each new element, whose sequence has been verified, can be used in subsequent assemblies without further sequencing. These genetic elements include *Y. lipolytica* parts such as promoters, genes, terminators, homologous arms, and selectable markers. The Lvl0 Module also contains *E. coli* parts like fluorescent reporters carrying overhangs for different levels and backbones with antibiotic resistance markers on the multicopy ColE1 replication origin for easy phenotypic selection (Section 4.2).

Since elements cloned in this module are used for GG assemblies, it is necessary to first remove recognition sites of certain restriction enzymes. This could be done during design by selecting appropriate regions of natural sequences or by introduction of specific codon substitutions. To remove a small number of undesirable sites, site-directed mutagenesis can be achieved using overlap extension PCR[5]. The full list of restriction sites that need to be excluded is provided in Supplementary Table 3. Depending on the exact element the list can be shortened as specified in the table.

Supplementary Table 3 Restriction sites that need to be excluded depending on the genetic element

Enzyme (isoschizomer)	Recognition site	Reverse complement [a]	Module [b]	IntUp and IntDn for Exp Module, and Marker [3]	Promoter Gene Terminator [c]	IntUp and IntDn for Del Module [c]
**BpiI** (BbsI)	GAAGAC	GTCTTC	Exp/Pro	-	+	+
**BsaI** (Eco31I)	GGTCTC	GAGACC	Exp	-	-	+
**BsmBI** (Esp3I)	CGTCTC	GAGACG	Exp/Lvl0	-	-	+
**AarI**	CACCTGC	GCAGGTG	Int	-	-	-
**LguI** (SapI)	GCTCTTC	GAAGAGC	Del	+	+	-
**NotI**	GCGGCCGC		Yali	-	-	+/-
**SgrDI**	CGTCGACG		Yali	-	-	+/-
**MssI** (PmeI)	GTTTAAAC		Yali	-	-	+/-
**SmiI** (SwaI)	ATTTAAAT		Yali	-	-	+/-

[a] – Recognition sites are specified in both orientation for non-palindromic sequences.
[b] – Name of the module where an enzyme is used.

[c] – Last three columns describe which recognition sites should be excluded (-) or might be retained (+) for exact genetic element lists. Please pay attention that different requirements apply to homology arms (designated as IntUp and IntDn) depending on which module they are appropriated for. To assure linearization of deletion cassette before *Y. lipolytica* transformation, at least one of the four sites (marked as +/-) need to be excluded from IntUp and IntDn sequences designed for the Del Module.

Lvl0 entry vectors can be assembled using BsmBI and the pYTK001 plasmid which contains *sfGFP*[6]. Resultant clones can be selected on LB-Cm plates (Section 4.1) by the lack of green fluorescence. An example of Lvl0 assembly for a PCR-amplified gene is shown in Supplementary Figure 2. The resultant construct represents the pGen-series in the Lvl0 module. An example of the primers used to assemble a Lvl0 plasmid with *Y. lipolytica HPD1* gene is provided in Supplementary Figure 3. Note if genes are purchased commercially, vectors containing spectinomycin resistance must be avoided as this is used in the Lvl1-series of plasmids. Examples of primer structures used for assembly of Lvl0 plasmids with terminator shown in Supplementary Figure 4. Supplementary Figures 5 and 6 contain primers for amplification of up and down homology arms (IntUp and IntDn), respectively. We recommend to use 500bp at least for IntUp and IntDn sequences for purpose of assembly of overexpression constructs. Such sequence length is satisfactory for efficient homologous integration while keeping the probability of including undesired restriction sites low (Supplementary Table 3). To design the homology arms for linearization with SmiI or MssI, which do not produce undesirable extra base at the ends please refer to Section 1.4. Primers for assembly of pPro-series of plasmid with promoter parts are shown in description of the Pro Module (Section 1.8).

The basic set of the toolkit is provided with set Lvl0 plasmids required for assembly of empty Lvl1 and Lvl2 vectors (Supplementary Table 8). It contains four previously characterized strong constitutive *Y. lipolytica* promoters, including promoters of genes *TEF1*, *EXP1*, *TDH1*, *FBA1*[7], and four characterized terminators, including terminators of *Y. lipolytica* gene *LIP2*[8] and *S. cerevisiae* gene *ADH1*, *PGK1*, and *ENO2*[6]. The nomenclature used for Lvl0 plasmids is provided in Supplementary Figure 7.

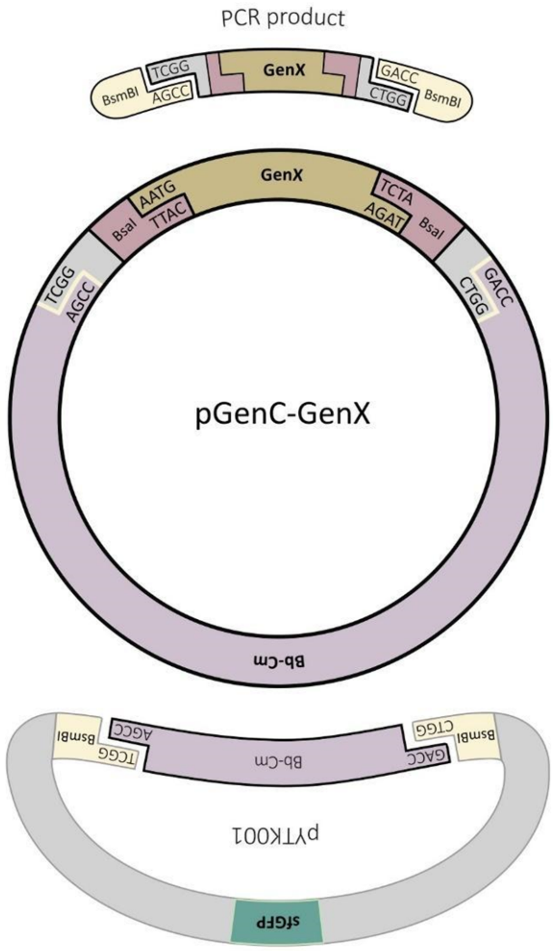

Supplementary Figure 2 Assembly of pGenC. A PCR-amplified gene is inserted into pYTK001 using GG assembly with BsmBI. The backbone contains a Cm-resistance marker (Bb-Cm).

HPD-F gcatCGTCTCATCGGGGTCTCAAATGTCACCTTCCGTCGAAGTC
HPD-R ctgaCGTCTCTGGTCGGTCTCATAGATTAAAGGTTGCCTCGCTTGGCCTG

BsmBI BsaI Start codon Stop codon

Supplementary Figure 3 pGen-series primer structure for insertion into vector. Primers shown here are an example for cloning *HPD1*. Lowercase bases are junk DNA to allow the Type IIs enzyme to cut. Underlined bases are 3'-terminal annealing regions that need to be designed specifically for the desired gene.

ScADH-F gcatCGTCTCATCGGGGTCTCTTCTATAACTCGAGGCGAATTTCTTATGA
ScADH-R atgcCGTCTCAGGTCGGTCTCTACTCGAAATGGGGAGCGATTTGCAGGCA

BsmBI BsaI

Supplementary Figure 4 pTer-series primer structure for insertion into vector. Primers shown here are an example for cloning the *ADH1* terminator. Lowercase bases are junk DNA to allow the Type IIs enzyme to cut. Underlined bases are 3'-terminal annealing regions that need to be designed specifically for the desired terminator.

IntC2Up-F

gcatCGTCTCATCGGGGTCTCTGCCTGCGGCCGCGTTTAAACACGCCAGACTTGGTTTATTAC

IntC2Up-R
gcatCGTCTCAGGTCGGTCTCTACCTACTAGCAGGTGTAGACTATCGGTAGAGCCAATTAGCTCCTCAAACGGAACTTTCCTG

BsmBI BsaI NotI MssI AarI Barcode1

Supplementary Figure 5 pYalC-IntUp-series primer structure for insertion into vector. Primers shown here are an example for cloning the upstream homology arm for integration into locus IntC2 of *Y. lipolytica*. Lowercase bases are junk DNA to allow the Type IIs enzyme to cut. Barcode1 is used as an annealing region for standard primer Barcode1-R to verify correct integration into the *Y. lipolytica* genome. This artificial sequence was also used as the first author authentic signature. Underlined bases are 3'-terminal annealing regions that need to be designed specifically for the desired upstream homology region.

IntC2Dn-F
gcatCGTCTCATCGGGGTCTCTGAGTACTAGCAGGTGTAAGAAGTTGGTGAAAACATTTACGCCTAACGCATTGCTTTTTCAGCCTT
C

IntC2Dn-R
gcatCGTCTCAGGTCGGTCTCTCGCAGCGGCCGCGTTTAAACGTGCAAAGGTGGAGAC

BsmBI BsaI NotI MssI AarI Barcode2

Supplementary Figure 6 pYalC-IntDn-series primer structure. Example primers for cloning the downstream homology arm for integration into locus IntC2 of *Y. lipolytica*. Lowercase bases are junk DNA to allow the Type IIs enzyme to cut. Barcode2 is used as an annealing region for standard primer Barcode2-F to verify correct integration into the *Y. lipolytica* genome. This artificial sequence was also used as the second author authentic signature. Underlined bases are 3'-terminal annealing regions that need to be designed specifically for the desired downstream homology region.

pYalA-Bb
pYalC-URA3
pYalC-IntB11Dn
pProC-TEF1
pGenC-hrGFP
pTerC-ScADH1

Series First letter of *E. coli* antibiotic resistance Genetic element

Supplementary Figure 7 Nomenclature of Lvl0 plasmids. pYal: plasmids suitable for assembling empty Lvl1 and empty Lvl2. pPro, pGen or pTer: plasmids suitable for assembling promoter, gene or terminator on Lvl1. Bb: plasmid backbone - includes high copy ColE1 origin of replication and antibiotic resistance.

1.3 (Exp Module) Assembly of overexpression constructs

The Exp Module is a multilevel GG platform for assembling up to three TUs together (Supplementary Figure 8). A single TU can be assembled by combining an empty Lvl1 vector with three Lvl0 plasmids each containing a promoter, gene or terminator. To construct a Lvl2.2 plasmid containing two TUs, an empty Lvl2.2 vector needs to be combined with a Lvl1.1 and Lvl1.2 plasmid.

To construct a Lvl2.3 plasmid containing three TUs, a Lvl2.3 vector needs to be combined with Lvl1.1, Lvl1.2 and Lvl1.3 plasmids. In all cases, each Lvl1 plasmid contains a single TU.

With the Exp Module, a variety of empty Lvl1 and Lvl2 vectors can be assembled with Lvl0 plasmids. Lvl1 vectors contain Sp resistance gene whereas Lvl2 vectors contain Ap resistance gene. Depending on the Lvl0 plasmid set that is selected, an assembled empty vector will differ by locus of integration, levels and sublevels. The sublevel of the Lvl1 plasmids determines the position of the TU in the subsequent Lvl2 assembly. The sublevel of the Lvl2 plasmid indicates the number of TUs that might be assembled on it. The basic plasmid set (Supplementary Table 8) only contains the *URA3* gene as the selectable marker as the marker-free approach is most commonly used for genomic integration.

YaliCraft parts are interchangeable with the most widely used *Y. lipolytica* GG system[2, 3]. However, the previous system utilised the same enzyme, BsaI, for the assembly of empty Lvl1 plasmids and for the subsequent assembly of a TU. As a result, an intermediate step for assembling empty vectors was introduced in the Exp Module. The intermediate plasmid set is named preLvl. A preLvl plasmid is assembled in a GG reaction using BsaI and contains the *mCherry* gene. To assemble an empty Lvl1 or Lvl2 vector from a preLvl plasmid, a GG reaction with BpiI is used instead. During this reaction, *sfGFP* replaces *mCherry* and appropriate overhangs for transcriptional unit assembly are inserted. The levels and sublevels of resultant empty vectors will depend on the selected Lvl0 plasmid with *sfGFP* which contains specific restriction sites and overhangs.

As part of the toolkit, there are 80 empty Lvl1 and Lvl2 plasmids designed for integration in 16 different genomic loci. These can be used for overexpression of up to 48 genes in the *Y. lipolytica* genome and this means that complex biosynthetic pathways can be built (Supplementary Table 8). The nomenclature used for Lvl1 and Lvl2 plasmids is described in Supplementary Figure 9.

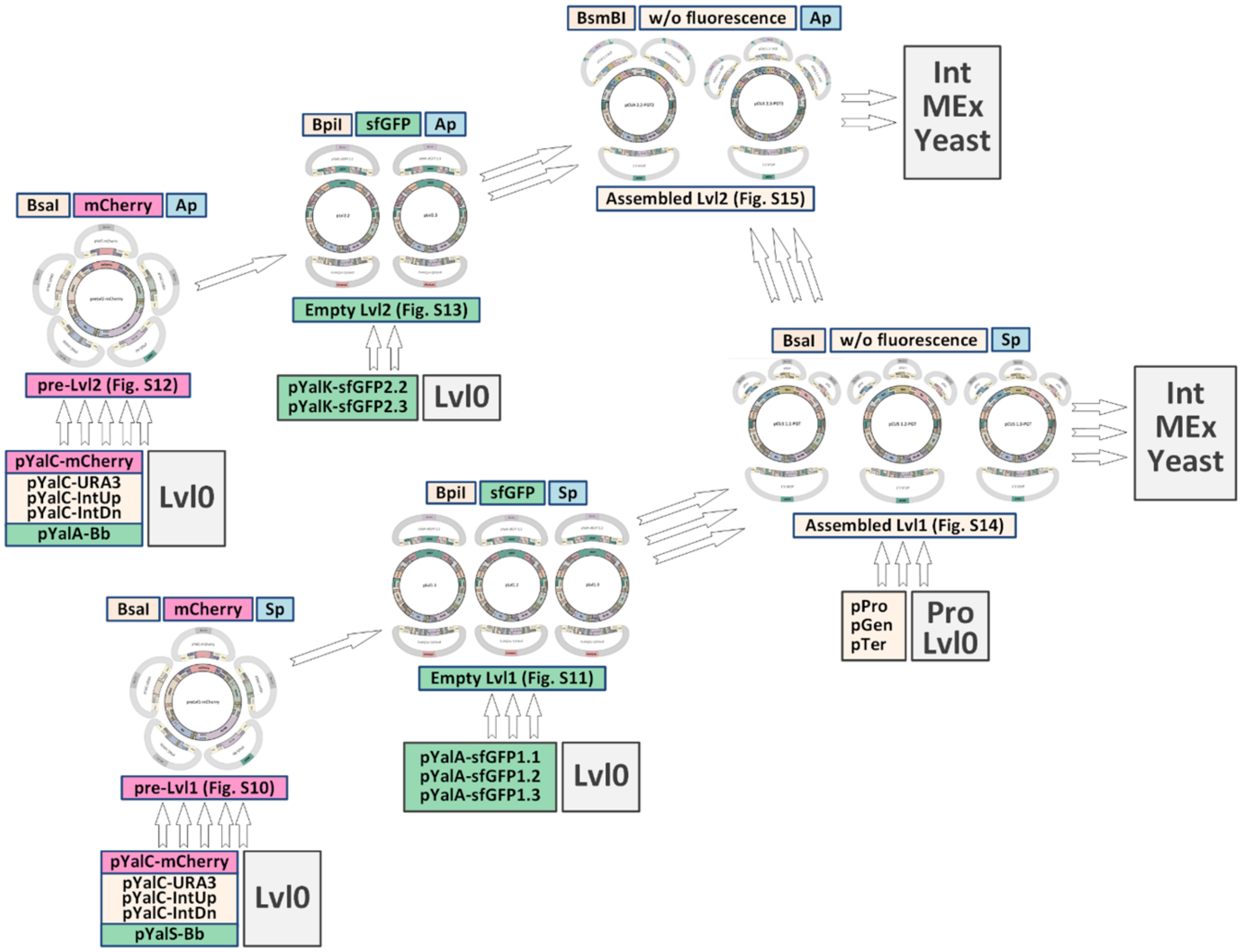

Supplementary Figure 8 General assembly scheme of empty vectors and overexpression constructs in the Exp Module. Each assembly is shown as a small pictogram. The plasmid series is shown below the picture together with reference for full-size figures in brackets. The name of the enzyme used for assembly, fluorescent reporter, and antibiotic resistance are specified above the picture. Arrows show the direction of workflow for assembly. Plasmid names participating in each assembly are specified together with module names.

pB8US1.1
pF11UA2.3

Integration Region
First letter of yeast selectable marker
First letter of *E. coli* antibiotic resistance
Plasmid level Plasmid sublevel

Supplementary Figure 9 The nomenclature of Lvl1 and Lvl2 plasmids. A colour code is provided below the plasmid names.

1.3.1 Assembly of empty Lvl1 vector

To assemble a preLvl1 vector, five Lvl0 plasmids are combined: pYalC-mCherry, pYalC-URA3, pYalS-Bb, and two plasmids carrying homology arms corresponding to the selected integration locus. For the example in Supplementary Figure 10, pYalC-IntC2Up and pYalC-IntC2Dn are required to construct a preLvl1-mCherry plasmid for integration into the IntC2 locus. For GG assembly, Protocol 3.1 with BsaI enzyme and thermal inactivation is required. Correct clones are red fluorescent when selected for on LB-Sp. These clones can be visually identified on the Petri dish using a 530nm excitation light source and a 620nm filter.

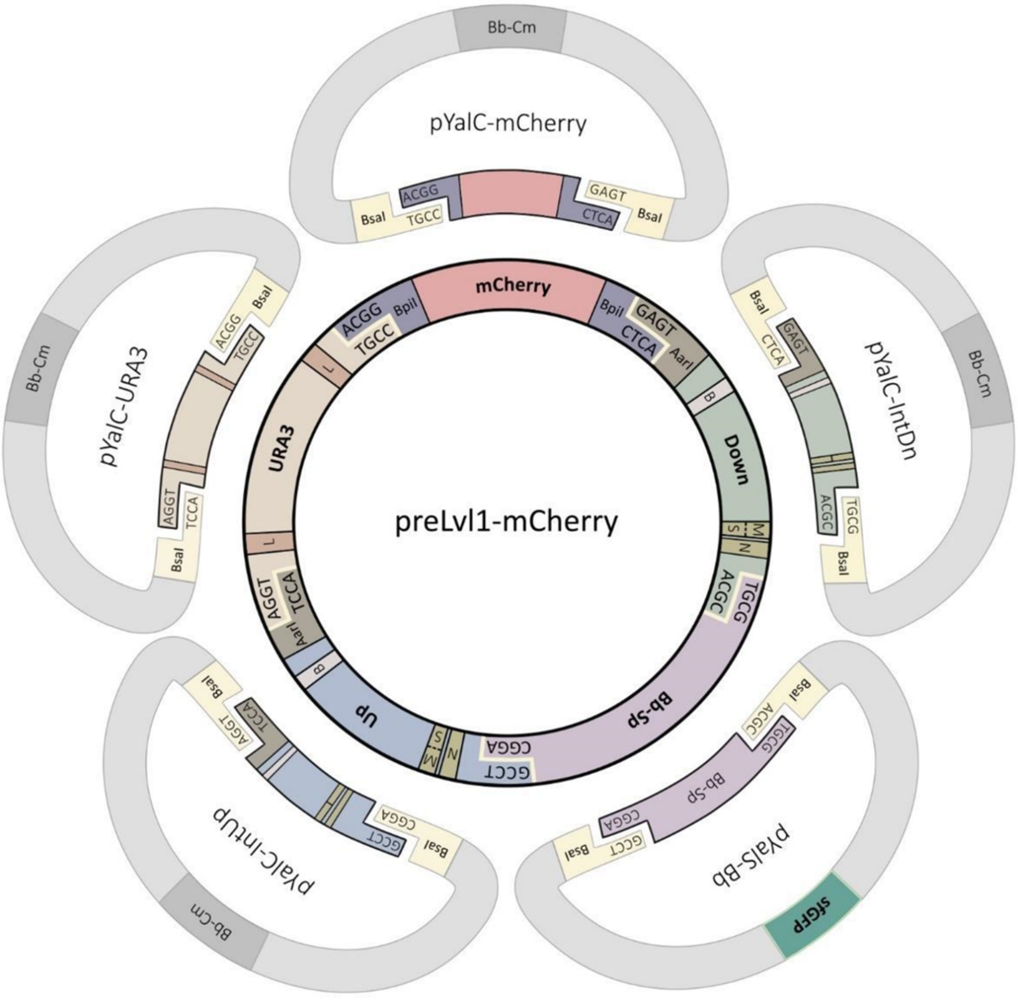

Supplementary Figure 10 Assembly of a preLvl1 plasmid. Overhangs are shown for each part. Yellow indicates enzyme cut sites. Abbreviations: B – barcode regions; Bb-Sp, Bb-Cm – backbones with different antibiotic resistances; L – Lox sites; M – MssI sites; N – NotI sites; S – SmiI sites. Up, Down – upstream and downstream homology arms.

Following preLvl-mCherry construction, empty pLvl1 plasmids with different sublevels are assembled. For this purpose, preLvl1-mCherry needs to be combined with one of three alternative plasmids: pYalA-sfGFP1.1, pYalA-sfGFP1.2 or pYalA-sfGFP1.3. This results in Lvl1.1, Lvl1.2 or Lvl1.3 plasmids (Supplementary Figure 11). For this reaction, use Protocol 3.1 with BpiI enzyme and thermal inactivation. Correct colonies can be selected for by green fluorescence (470nm excitation/580nm emission) when plated on LB-Sp.

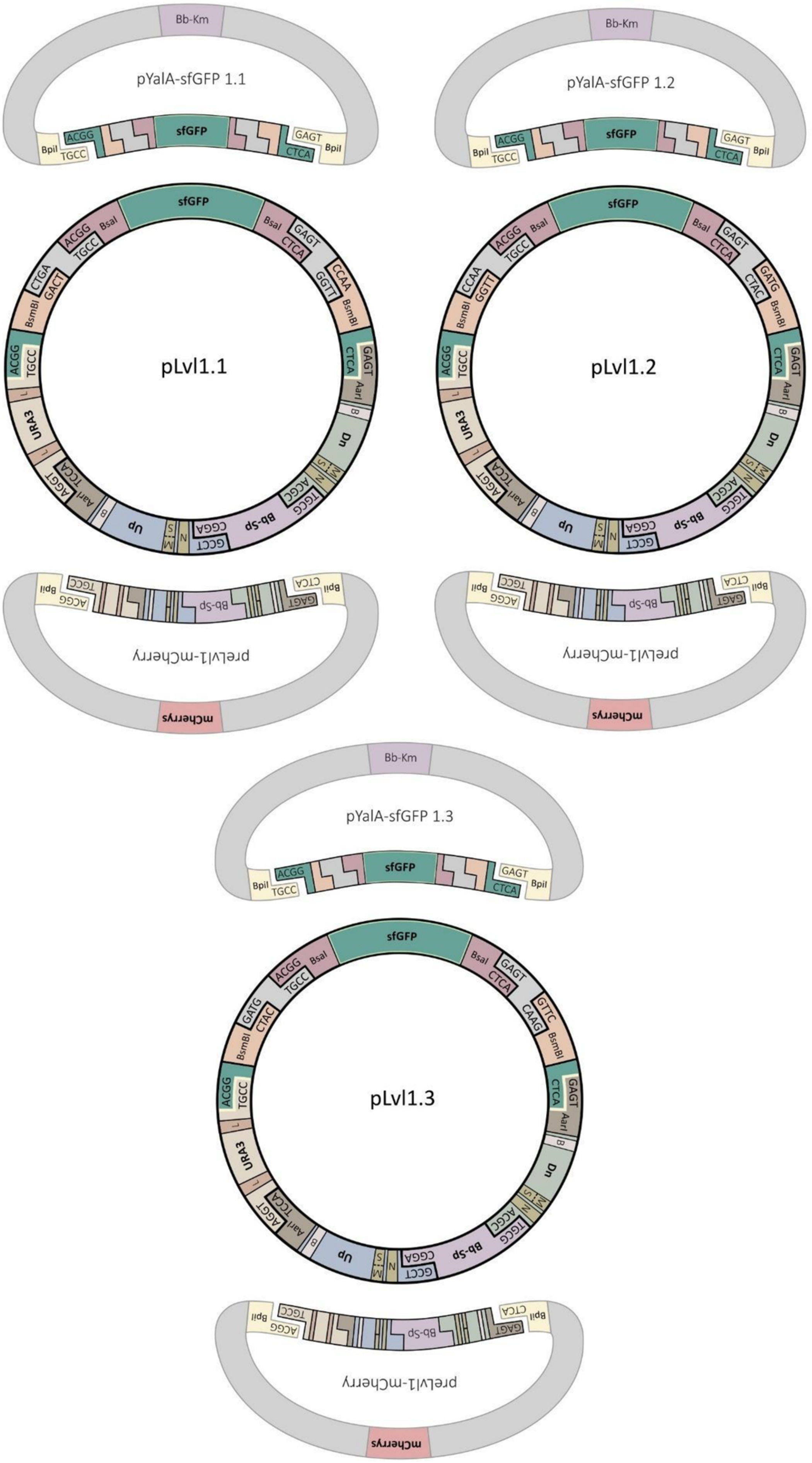
Bb-Km
pYalA-sfGFP 1.1
pYalA-sfGFP 1.2
pYalA-sfGFP 1.3
sfGFP
BpiI
ACGG
TGCC
GAGT
CTCA
BsaI
BsmBI
CTGA
GACT
CCAA
GGTT
GATG
CTAC
GTTC
CAAG
AarI
URA3
Dn
Up
Bb-Sp
TCCA
AGGT
ACGC
TGCG
CGGA
GCCT
pLvl1.1
pLvl1.2
pLvl1.3
prelvl1-mCherry
mCherry

Supplementary Figure 11 Assembly of empty pLvl1.1, pLvl1.2, and pLvl1.3 vectors. Overhangs are shown for each part. Yellow indicates enzyme cut sites. Abbreviations: B – barcode region; Bb-Sp, Bb-Ap – backbones with different antibiotic resistances; L – Lox sites; M – MssI sites; N – NotI sites; S – SmiI sites; Up, Dn – upstream and downstream homology arms.

1.3.2 Assembly of empty Lvl2 vector

Next, to assemble preLvl2 vector - preLvl2-mCherry - five Lvl0 plasmids are combined. This includes: pYalC-mCherry, pYalC-URA3, pYalA-Bb and two plasmids carrying homology arms corresponding to the selected integration locus (Supplementary Figure 12). For GG assembly of this plasmid use Protocol 3.1 with BsaI and thermal inactivation. Correct clones are selected by red fluorescence on LB-Ap plates.

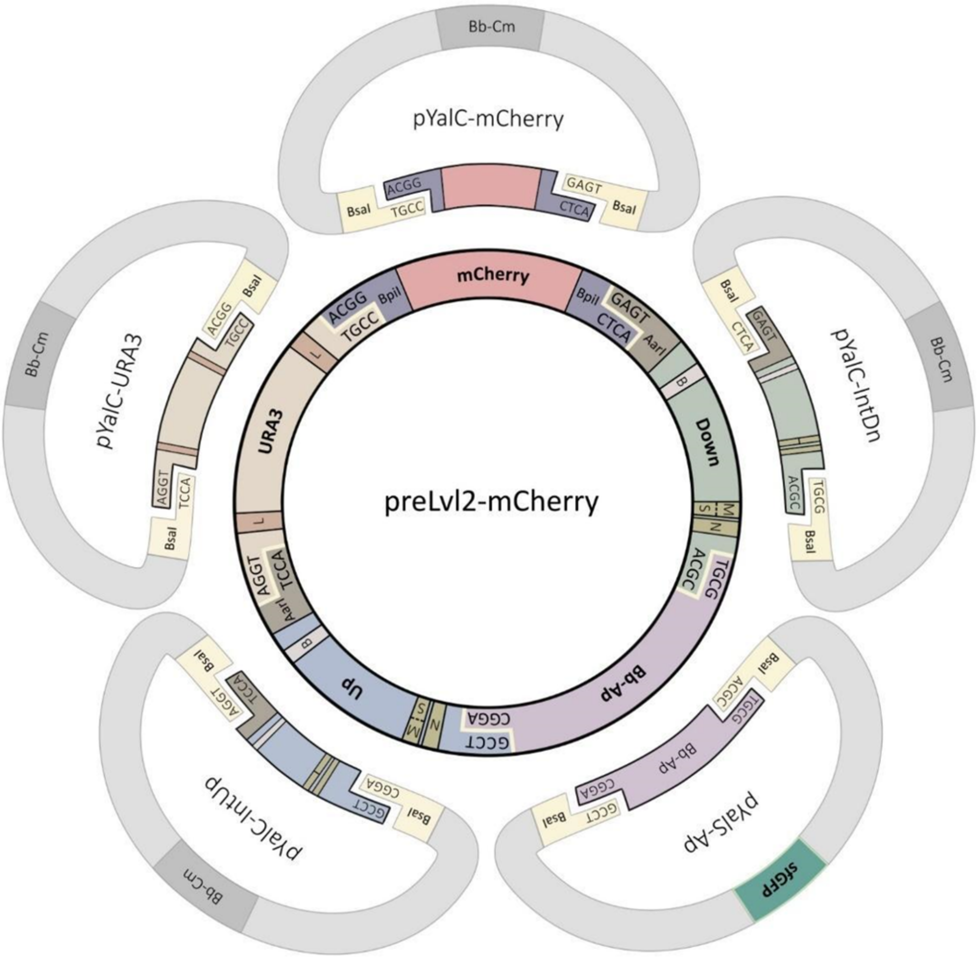

Supplementary Figure 12 Assembly of preLvl2 plasmid. Overhangs are shown for each part. Yellow indicates enzyme cut sites. Abbreviations: B – barcode regions; Bb-Ap, Bb-Cm – backbones with different antibiotic resistances; L – Lox sites; M – MssI sites; N – NotI sites; S – SmiI sites; Up, Down – upstream and downstream homology arms.

At the second stage, by combining the preLvl2-mCherry plasmid with one of two alternative plasmids pYalK-sfGFP2.2 or pYalK-sfGFP2.3, empty plasmids pLvl2.2 or pLvl2.3 can be obtained, respectively (Supplementary Figure 13). For these GG assemblies use Protocol 3.1 with BpiI enzyme and thermal inactivation. Correct clones are selected for by green fluorescence on LB-Ap.

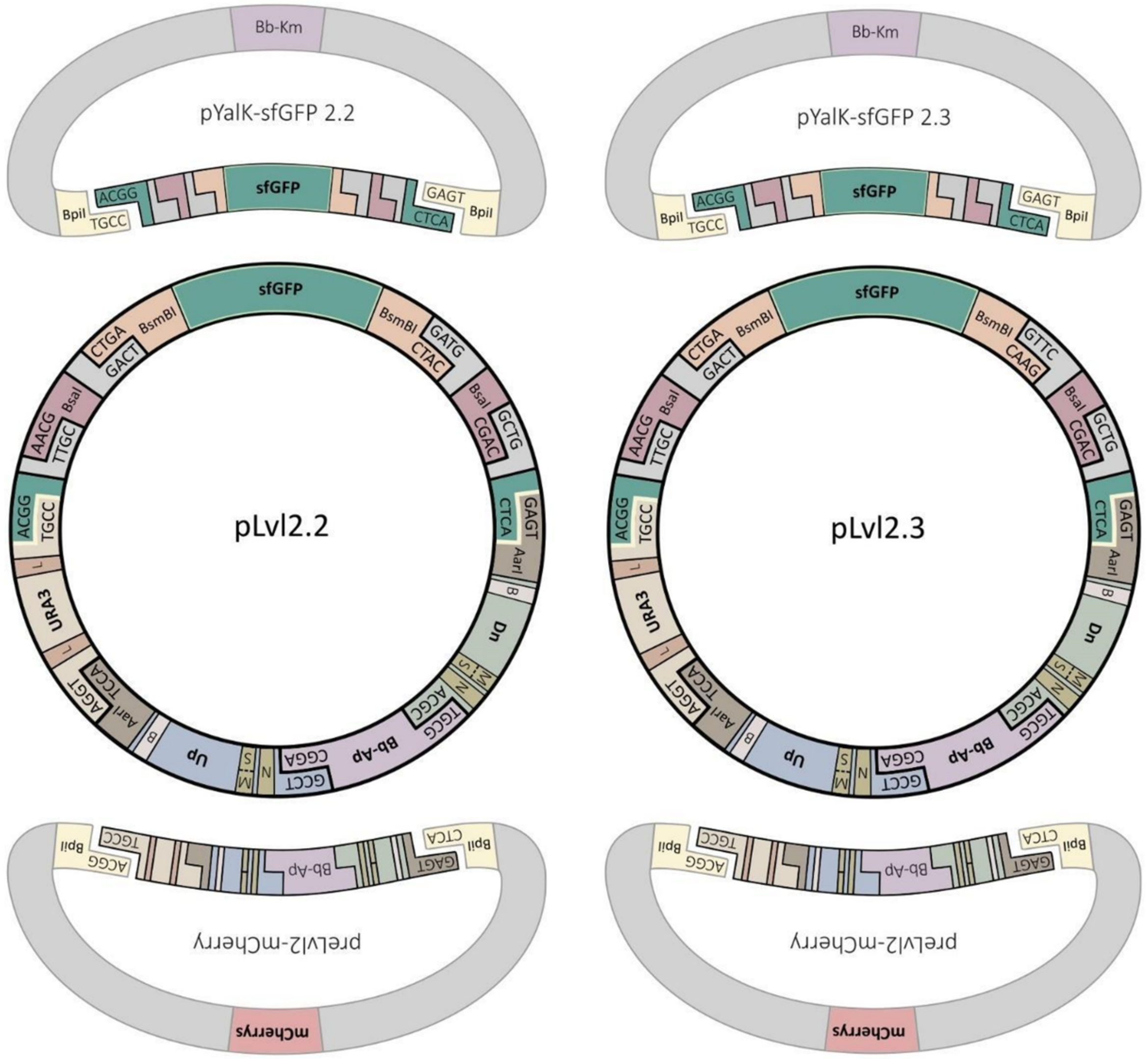

Supplementary Figure 13 Assembly of empty pLvl2.2 and pLvl2.3 vectors. Overhangs are shown for each part. Yellow indicates enzyme cut sites. Abbreviations: B – barcode region; Bb-Ap, Bb-Km – backbones with different antibiotic resistances; L – Lox sites; M – MssI sites; N – NotI sites; S – SmiI sites; Up, Dn – upstream and downstream homology arms.

1.3.3 Assembly of single TU on the Lvl1

For assembly of a single TU, an empty Lvl1 plasmid needs to be used. The choice of selected empty vector will determine the locus of integration of the resulting construct. The selected empty Lvl1 vector needs to be combined with three Lvl0 plasmids each with either a promoter (pPro-series), gene (pGen-series) or terminator (pTer-series) to make plasmids that can be used for Lvl2 assembly (Supplementary Figure 14). For GG assembly, use Protocol 3.1 with BsaI enzyme and thermal inactivation. Correct clones are selected for by lack of green fluorescence on LB-Sp.

Lvl1 plasmids also contain sublevels - 1.1, 1.2 or 1.3. The only difference between different sublevels is the overhangs produced by BsmBI. These overhangs are then used to determine the order in which TUs will be assembled on the Lvl2 plasmid. For example, to insert two TUs into a Lvl2 vector, you will need the Lvl2.2 backbone with pLvl1.1 containing the TU you want in the first position, and pLvl1.2 containing the TU you want in the second position (Supplementary Figure 15). Therefore, it is important to choose the correct Lvl1 sublevel for downstream Lvl2 assemblies.

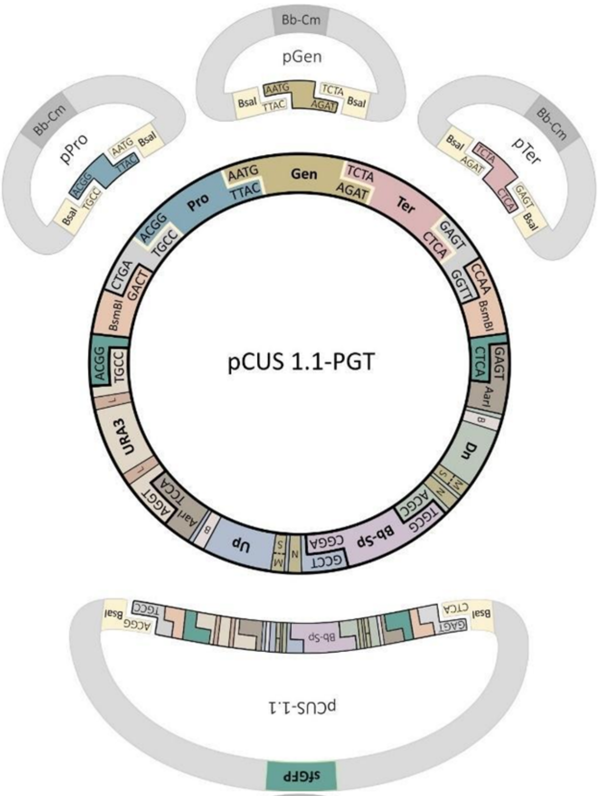
Bb-Cm
pGen
pPro
pTer
BsaI
AATG
TTAC
TCTA
AGAT
ACGG
TGCC
GAGT
CTCA
Pro
Gen
Ter
CTGA
GACT
CCAA
GGTT
BsmBI
pCUS 1.1-PGT
URA3
AarI
Up
Dn
Bb-Sp
CGGA
GCCT
TCCA
AGGT
pCUS-1.1
sfGFP

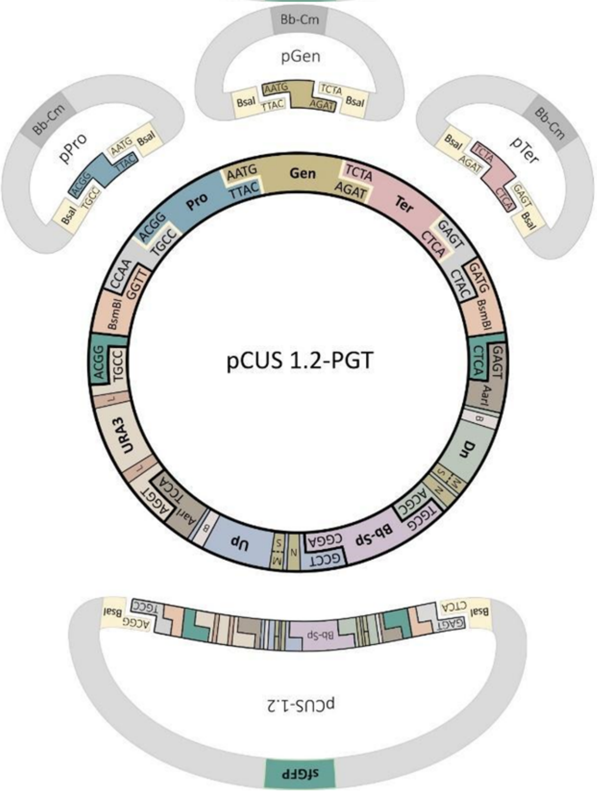
Bb-Cm
pGen
pPro
pTer
BsaI
AATG
TTAC
TCTA
AGAT
ACGG
TGCC
GAGT
CTCA
Pro
Gen
Ter
CCAA
GGTT
GATG
CTAC
BsmBI
pCUS 1.2-PGT
URA3
AarI
Up
Dn
Bb-Sp
CGGA
GCCT
TCCA
AGGT
pCUS-1.2
sfGFP

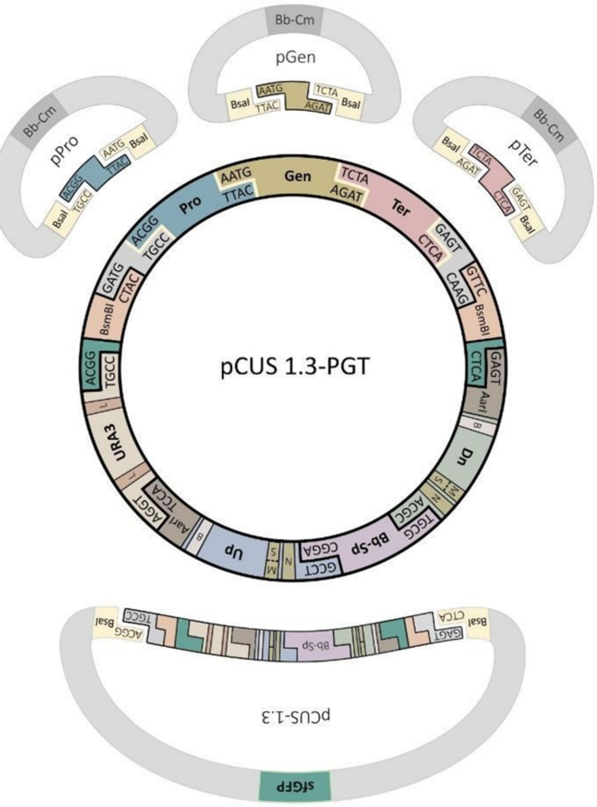

Supplementary Figure 14 Assembly of a single TU on the pLvl1.1, pLvl1.2, and pLvl1.3. The only difference between different sublevels is overhangs produced by BsmBI that determine the order in which TUs will be assembled on the Lvl2. Overhangs are shown for each part. Yellow indicates enzyme cut sites. Abbreviations: Pro – promoter, Gen – gene, Ter – terminator; B – barcode region; Bb-Sp, Bb-Cm – backbones with different antibiotic resistances; L – Lox sites; M – MssI sites; N – NotI sites; S – SmiI sites; Up, Dn – upstream and downstream homology arms.

1.3.4 Assembly of several TUs on the Lvl2

Empty Lvl2 vectors are used for the assembly of two or three TUs. The sublevel (2.2 or 2.3) will specify the number of TUs (two for 2.2 or three for 2.3) that can be assembled. Furthermore, the selected empty vector will determine the locus of integration of the resulting construct. Empty Lvl2.2 vectors need to be combined with two plasmids (Lvl1.1 and Lvl1.2) whereas empty Lvl2.3 vectors need to be combined with three Lvl1 plasmids (Lvl1.1, Lvl1.2 and Lvl1.3) (Supplementary Figure 15). As a result, the integrative vector can be assembled carrying TUs in the order determined by sublevels of the selected Lvl1 plasmids. For GG assembly, use Protocol 3.1 with BsmBI enzyme and thermal inactivation. Correct clones are selected for by lack of green fluorescence on LB-Ap.

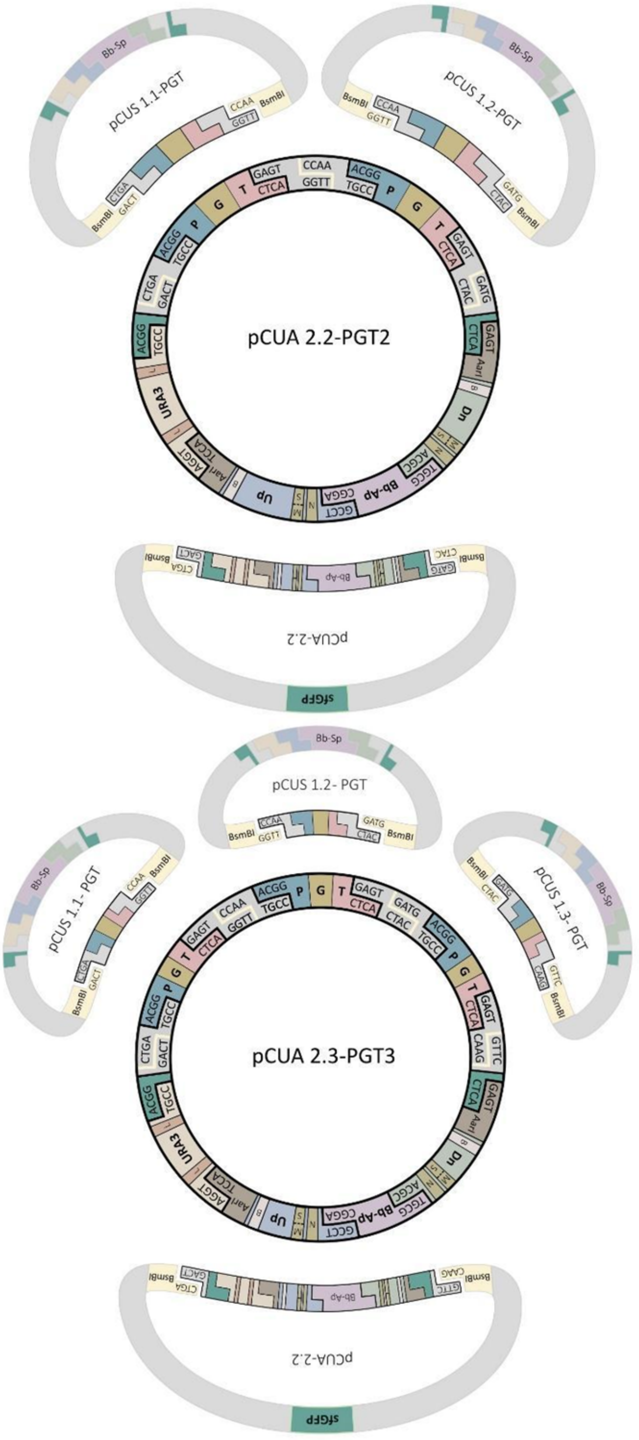

Supplementary Figure 15 Assembly of two TUs on the Lvl2.2 and three TUs on the Lvl2.3. Overhangs are shown for each part. Yellow indicates enzyme cut sites. Abbreviations: P – promoter, G – gene, T – terminator; B – barcode region; Bb-Sp, Bb-Ap – backbones with different antibiotic resistances; L – Lox sites; M – MssI sites; N – NotI sites; S – SmiI sites; Up, Dn – upstream and downstream homology arms.

1.4 (Del Module) Assembly of gene disruption construct

The Del Module utilises one-pot GG assembly and results in a vector that can be used to inactivate a gene in the *Y. lipolytica* genome. For this assembly, the empty pDelUK-RG vector is required which contains a *Y. lipolytica URA3* marker flanked by two *E. coli* reporter genes - *mScarlet* (RFP) and *sfGFP*. During the GG reaction, both reporters are replaced by PCR products amplified

from the *Y. lipolytica* genome, which contain the sequences for upstream and downstream homology arms (IntUp and IntDn) (Supplementary Figure 16). For GG assembly, use Protocol 3.3 with LguI enzyme and thermal inactivation. Correct clones are selected by lack of both green and red fluorescence on LB-Km (Supplementary Figure 17). The resulting pDel-series vector is a ready-to-use construct for inactivation of any selected gene. Importantly, due to the burden caused by the expression of two fluorescent proteins, the empty vector pDelUK-RG has a low copy number. Therefore, a separate plasmid isolation protocol is provided (Protocol 3.4). Afterwards, assembled pDel-series plasmids can be isolated using the general miniprep protocols (*e.g.* Qiagen #27104).

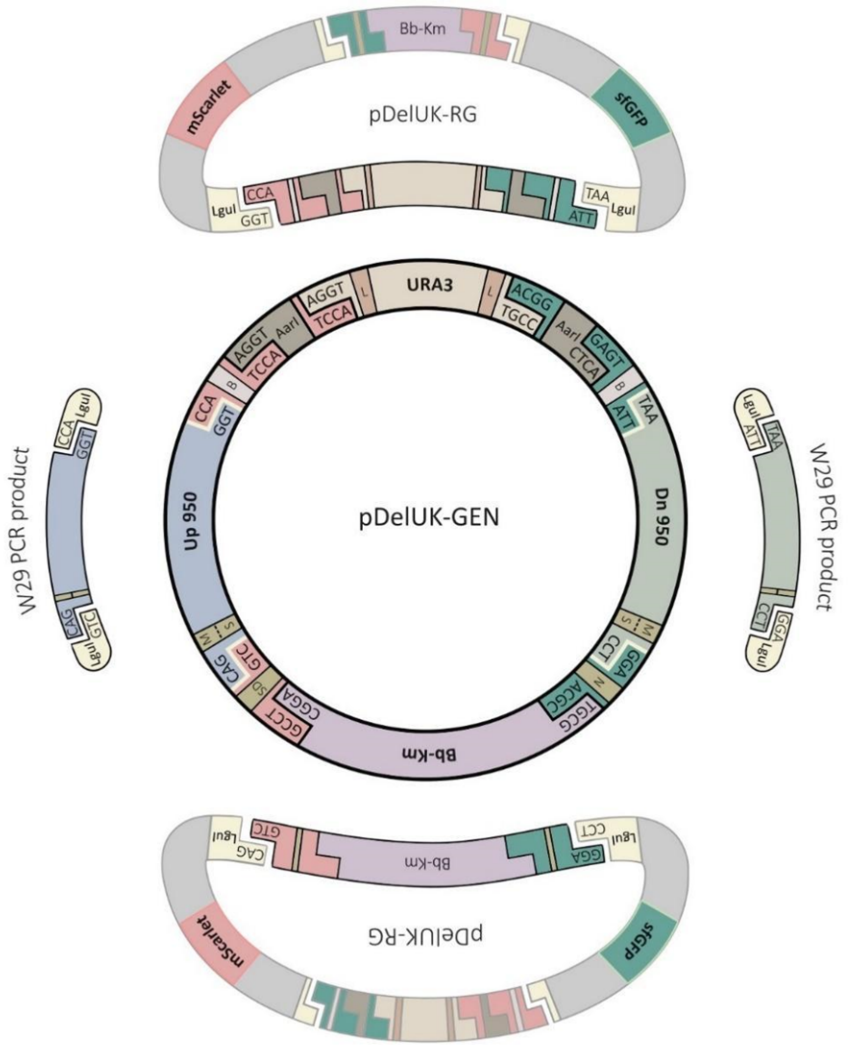

Supplementary Figure 16 Assembly of pDel-series plasmid. The same empty pDelUK-RG vector is shown at the top and the bottom. PCR products containing the homology regions from wild type *Y. lipolytica* strain are shown on the left and right. Overhangs are shown for each part. Yellow indicates enzyme cut sites. Abbreviations: B – barcode region; Bb-Km – backbones with Km-resistance gene; L – Lox sites; M – MssI sites; N – NotI site; SD – SgrDI site; S – SmiI sites; Up, Dn – upstream and downstream homology arms.

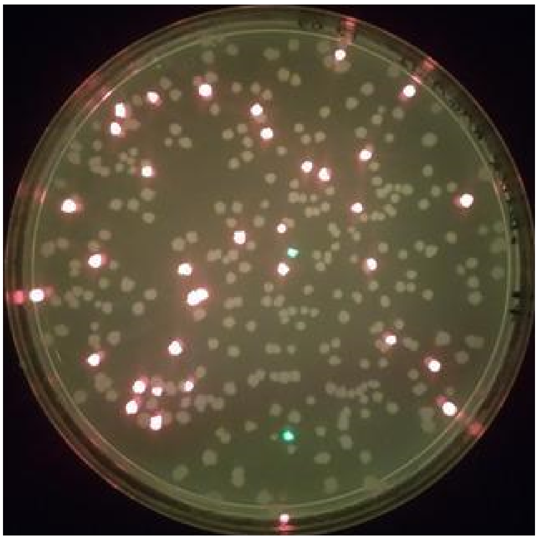

Supplementary Figure 17 Appearance of LB-Km plate containing *E. coli* transformed with GG assembly of pDel-series. Colonies that have been correctly assembled are not fluorescent under blue light (470nm excitation/580nm emission).

Lengths of homology arms may range from 400 - 1000 bp. Longer flanks are preferable for higher efficiency of homologous recombination, especially if marker-based integration is used without Cas9-helper application. However, the lengths of the selected homology arms may be limited by the presence of recognition sites (Supplementary Table 3) used for their assembly and downstream applications. This issue is partially solved by the use of LguI and AarI, which recognize rarer 7-bp sites unlike most other Type IIs endonucleases which recognise 6 bp. LguI is required for assembly of pDel-series (Supplementary Figure 17), while AarI is utilised for homology arm exchange mechanism (Supplementary Figure 20). One more restriction enzyme is required for the linearization of final construction before *Y. lipolytica* transformation. Therefore, selected homology arms should not contain the recognition sites of at least one of the following enzymes: NotI, SgrDI, MssI or SmiI. All of them recognize rare 8-bp sites that greatly simplifies the task. The vector part of pDel-series contains standard SgrDI and NotI sites that flank the upstream and downstream homology arms respectively and can be used for linearization. Note that such linearization always leads to short non-homologous extensions (5 bp or more) from the both sides. Although, such extensions do not halt homologous integration, their effects on preciseness and efficiency of *Y. lipolytica* recombination still need to be clarified. Alternatively, homology arms can be designed in such way that halves of recognition sites are naturally present at the required positions and, therefore produced ends will show a perfect match with chromosomal sequences. For this purpose, MssI or SmiI sites are recommended. Both enzymes recognize 8 bp and produce blunt ends. Therefore, selected homology arms on the corresponding ends should contain either AAAC (half of MssI site) or AAAT (half of SmiI site). As an example of such 'scarless linearization' technique, we provide primers for *AAT1* gene disruption (Supplementary Figure 18). Note that homology arms have been chosen in such way that both of them contain halves of MssI sites as part of the natural sequences. Following this rule, we also designed most of the standard homology arms used for overexpression vectors of Lvl1 and Lvl2 (Supplementary Table 5).

AAT1-Up-F	atgcGCTCTTCACAGGTTTAAACGCGTCTTTAACAGGCGAAAAAC
AAT1-Up-R	gataGCTCTTCTTGGCCTCCTTCTCGGCCTCTC
AAT1-Dn-F	atgcGCTCTTCATAATGTCGAGCAGGGCCACGAG
AAT1-Dn-R	gataGCTCTTCTTCCGTTTAAACTGTCCTACAGTTTACACAC
LguI MssI	

Supplementary Figure 18 Example primers used for cloning upstream and downstream homology arms of *AAT1*. This structure of primers can be used for the assembly of a disruption cassette using empty pDelUK-RG vector. Blue bases are MssI sites, while yellow bases are LguI sites. Lowercase bases are junk DNA to allow LguI to cut. Underlined are 3'-terminal annealing regions that need to be designed specifically for the gene of interest.

1.5 (Int Module) Exchange of homology arms

In comparison with other multigene overexpression toolkits, *YaliCraft* allows one-pot exchange of homology arms directly on overexpression constructs with one, two or three TUs. In this reaction, assembled Lvl1/Lvl2 overexpression constructs can receive homology arms from an empty Lvl1/Lvl2 vector or any disruption construct of the pDel-series. Homology arm exchange may be necessary to avoid the reassembly of TUs if multiple copies of the same overexpression construct need to be integrated, in case when an integration locus is already used or several alternative constructs were assembled for integration at the same locus. Besides, homology arm exchange enables quick and easy way to substitute one construction in the yeast genome with another. The one-pot GG protocols require only two days to get a redirected construct ready for yeast transformation. At the same time, the Int Module together with Cas Module (Section 1.7) represents the system for quick redirection of both components of Cas9-based integration system, *i.e.* donor and guide.

1.5.1 Exchange of homology arms between Lvl1 and Lvl2

An assembled Lvl2 plasmid with several TUs can be recombined with any available empty Lvl1 plasmid (Supplementary Figure 19). For the GG reaction, use Protocol 3.2 with AarI. Since the final construct also contains functional AarI sites, no thermal inactivation is applied after the final ligation step. The resultant plasmid with overexpression TUs and renewed homology arms (IntUp and IntDn) can be selected by lack of green fluorescence on LB-Sp plates.

A similar approach can be used in the opposite direction to transfer homology arms from any empty Lvl2 plasmid to an assembled Lvl1 plasmid with a single TU. The recombinant plasmid can be selected by lack of green fluorescence on LB-Ap.

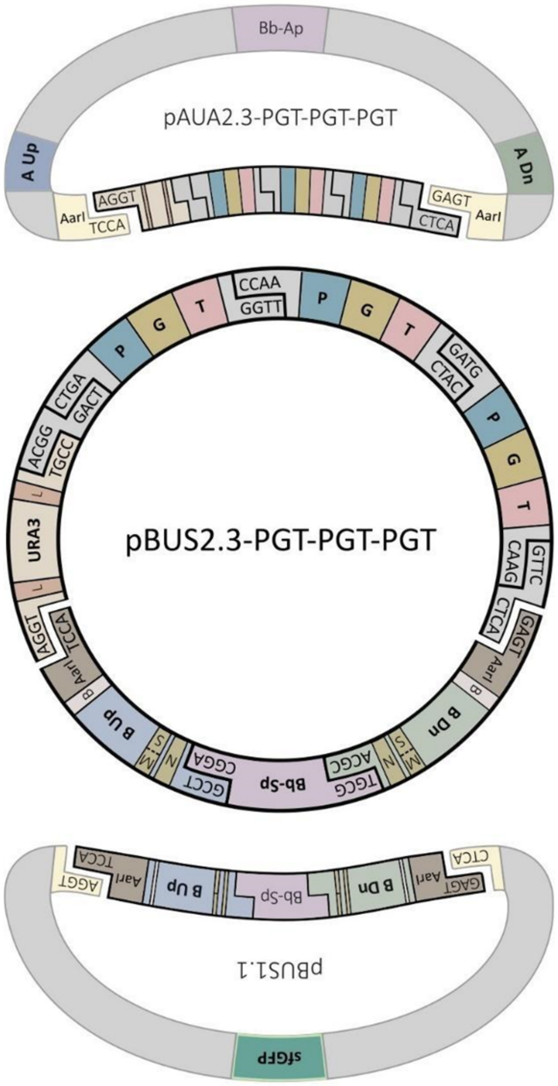

Supplementary Figure 19 Homology arms can be transferred from an empty Lvl1 vector (*e.g.* pBUS1.1) to an assembled Lvl2 plasmid (*e.g.* pAUA2.3-PGT-PGT-PGT) resulted in redirected vector (*e.g.* pBUS2.3-PGT-PGT-PGT). Abbreviations: P – promoter, G – gene, T – terminator; B – barcode region; Bb-Ap, Bb-Sp – backbones with different antibiotic resistances; L – Lox sites; M – MssI sites; N – NotI sites; S – SmiI sites; Up, Dn – upstream and downstream homology arms.

1.5.2 Transfer of homology arms from pDel-series to Lvl1 and Lvl2

Any disruption vector of the pDel-series can also be used as a donor of homology arms for overexpression constructs of Lvl1 or Lvl2 (Supplementary Figure 20). The orientation of AarI sites on the pDel-series makes this assembly irreversible because the new plasmid, when correctly assembled, does not contain any active AarI sites. To transfer homology arms between the pDel-series and Lvl1/2 plasmids, use GG reaction Protocol 3.1 with AarI enzyme and terminal inactivation. The recombinant plasmid with the overexpression construct (TUs) and renewed homology arms (IntUp and IntDn) can be selected for on LB-Km plates. Note that the resultant construct can be integrated in genomic loci with the already disrupted gene or in wild type gene, therefore, enabling simultaneous gene disruption and overexpression.

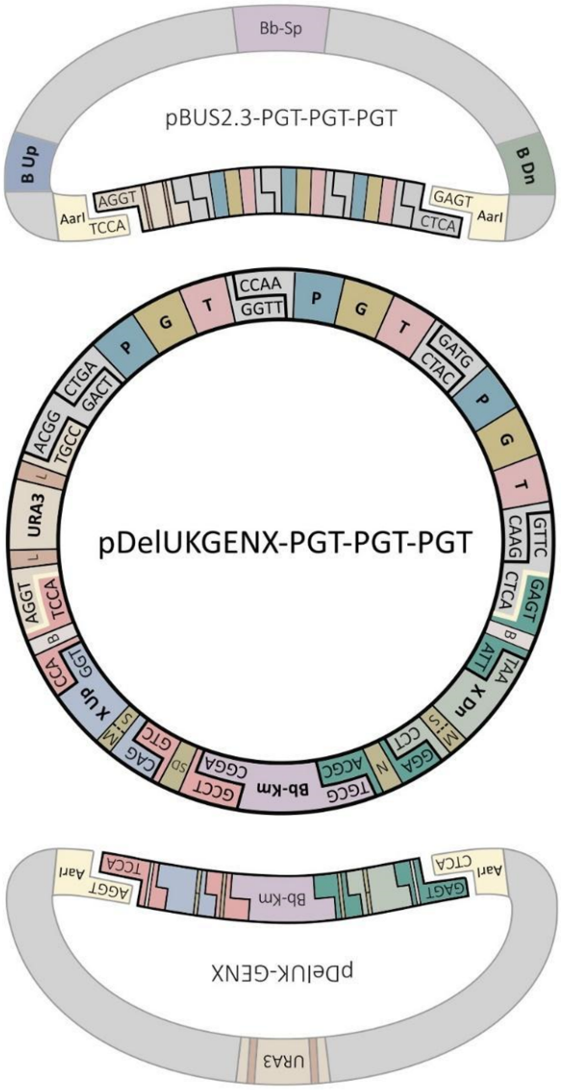

Supplementary Figure 20 Homology arms can be transferred from pDel-series plasmid (*e.g.* pDelUK-GENX) to an assembled Lvl2 plasmid (*e.g.* pBUS2.3-PGT-PGT-PGT) resulted in redirected vector (*e.g.* pDelUKGENX-PGT-PGT-PGT). Abbreviations: P – promoter, G – gene, T – terminator; B – barcode region; Bb-Km, Bb-Sp – backbones with different antibiotic resistances; L – Lox sites; M – MssI sites; N – NotI sites; SD – SgrDI site; S – SmiI sites; Up, Dn – upstream and downstream homology arms.

1.6 (MEx Module) Excision of yeast selectable marker

The MEx Module is designed for instant switching from a marker-free construction to a marker-based one. This becomes possible with construction of vector versions with and without a marker using the same GG assembly reaction mixture. For any assembly derived from the Exp, Del or Int Modules we recommend transforming GG reaction in two independent *E. coli* strains. One of them is a regular transformation host (such as DH5alpha, XL1-Blue *etc.*), while the other is *EcoCre* which under induction conditions (37 ^{o}C) overexpresses Cre recombinase. This leads to instant *URA3* marker excision that is flanked by Lox66 and Lox71 sites in all constructs (Supplementary Figure 21). Transformants can be selected for on LB plates with an appropriate antibiotic depending on the plasmid backbone used. *EcoCre* is provided as part of the toolkit (Supplementary Table 9). The protocol for preparing *EcoCre* competent cells is also provided (Protocol 3.6). Efficiency of the

marker excision was 100% as verified by restriction analysis and, therefore, the number of correct clones entirely depends on the GG reaction. As a result, the MEx Module saves on the DNA re-assemblies several working days every time when a marker-based construct is required for difficult modifications.

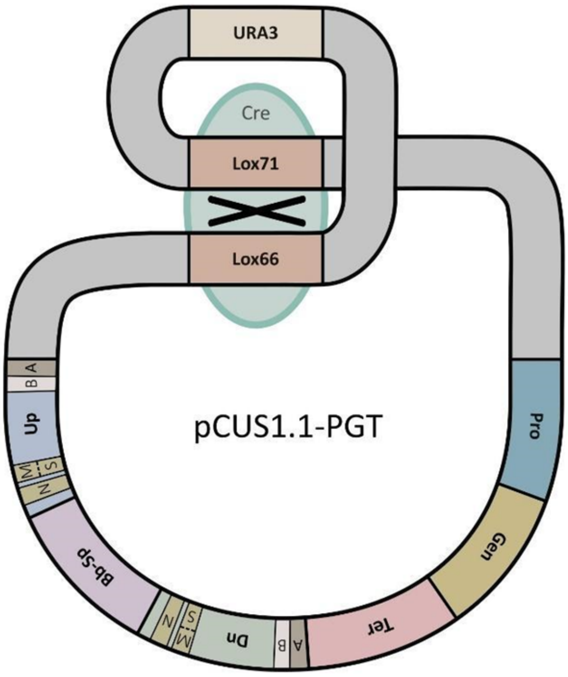

Supplementary Figure 21 Excision of *URA3* marker by *EcoCre* strain. Lvl1.1 plasmid is used here as an example to show how Cre loops-out the marker by site-specific recombination of the Lox66 and Lox71 sites. Abbreviations: Pro – promoter, Gen – gene, Ter – terminator; B – barcode region; Bb-Sp – backbones with Sp-resistances; A – AarI sites; M – MssI sites; N – NotI sites; S – SmiI sites; Up, Dn – upstream and downstream homology arms.

1.7 (Cas Module) Re-encoding Cas9-helper

The Cas Module allows redirection of episomal Cas9-helper plasmid to introduce double-strand breaks at any desired loci of the *Y. lipolytica* genome. An assembled Cas9-helper plasmid contains all the required genetic elements supporting this function *i.e.* a *Y. lipolytica* autonomously replicating sequence (ARS), nourseothricin (Nat) resistance marker, *Streptococcus pyogenes cas9* gene with nuclear localization signal (NLS), and Pol III-driven TU expressing guide (sgRNA) that includes a variable 20 bp recognition sequence. Only this short guide sequence needs to be changed to redirect Cas9-helper to new genomic loci.

The empty pCasNA-RK vector for this sequence contains a two-marker cassette with Km- resistance and streptomycin (St) susceptibility. The latter is an *rpsL* gene which enables counterselection in media with St. This counterselection is used to substitute the cassette with a 20 bp recognition sequence using oligonucleotide (oligo) recombineering (Supplementary Figure 22). To assemble new Cas9-helpers a 90-base oligo which contains these 20 nucleotides (nt) flanked by 35 nt homology regions on either side which recombines with the empty pCasNA-RK plasmid (Supplementary Figure 23) is required.

Specific 20 nt corresponding to the locus of interest can be designed using the ChopChop tool (http://chopchop.cbu.uib.no/). It is important to mention that for the purpose of marker-free

integration, Cas9 should not recognize and cleave the donor construct, and therefore, these 20 bp need to be selected from the region between the upstream and downstream homology arms.

The assembly of a Cas9-helper plasmid requires mixing 70 ng of pCasNA-RK with 1.0 ug (35 pmol) of the 90 bp oligo followed by transformation of *EcoRed*. Recombination takes place inside the transformed strain which under induction conditions (37 ^{o}C) overexpresses three phage-derived lambda Red proteins: Gam, Exo, and Beta. *EcoRed* is provided as part of the toolkit (Supplementary Table 9). The protocol for *EcoRed* competent cell preparation is also provided (Protocol 3.5). Transformants can be selected for at 30 ^{o}C on LB-Ap-St plate, verified using restriction digestion (*e.g.* with HindIII). If sequencing is required, primers sgRNA-seq-F and sgRNA-seq-R can be used (Supplementary Table 10).

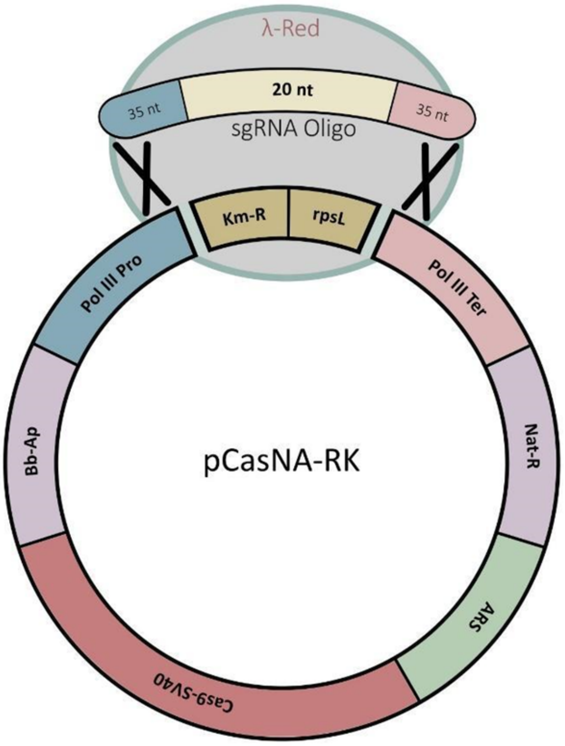

Supplementary Figure 22 Re-encoding of the guide sequence by oligo recombineering with empty Cas9-helper plasmid pCasNA-RK. The single-stranded oligonucleotide is 90 bp long and contains a 20 bp recognition sequence. Recombination occurs in the *EcoRed* strain. Transformed colonies are selected based on Sp-resistance which is a result of *rpsL* marker elimination. Abbreviations: Cas9-SV40 - *cas9* gene with SV40 NLS; Km-R - Km-resistance gene; Nat-R – Nat-resistance gene; Pol III Pro - hybrid promoter SCR1'- tRNA^{Gly} of RNA polymerase III; Pol III Ter - terminator of RNA polymerase III.

Recognition sequence in the genome		(**CACGAGCTTGAGCACTCGAGCGG**)
TCGATGGGCCCCCGGTTCGATTCCGGGTCGGCGCA		**CACGAGCTTGAGCACTCGAG**GTTTTAGAGCTAGAAATAGCAAGTTAAAATAAGGC

Supplementary Figure 23 Structure of 90-base single-stranded oligo used for assembly of pCas-series plasmids. An example for assembling a pCasNA-URA3 helper plasmid that cuts inside the *URA3* gene is shown. Green, 35-base homology regions to plasmid sequence; these are kept the same for all targets. Blue, conservative NGG sequence of protospacer adjacent motif (PAM) that

should not be included in the sequence of the oligo. Yellow, 20 bases of the recognition sequence - the only part that need to be changed during design of a new oligo.

Several alternative approaches have been used to change the 20 bp recognition sequence on episomal Cas9-helpers, including USER cloning[9] and GG assembly[10] as the most reliable and fast. For all methods involving propagation in *E. coli*, a CRISPR/Cas9 modification cannot be introduced in less than four days due to the limitation of the growth rates, *i.e.* one day (from day 1 to day 2) for cultivation of the bacteria and two days (from day 2 to day 4) for the yeast. Cloning-free assembly, which skips the E. coli step by using the gap repair mechanism in *S. cerevisiae,* enables yeast transformant isolation on day 3[11, 12]. However, this method is not applicable for most non-*Saccharomyces* yeasts and therefore is not considered here. Alternatively, GG reaction mixture can be directly transformed in yeast[13]. However, this protocol still takes four days, since the transformed yeast requires a longer recovery time. The undeniable advantage of the cloning-based techniques is that assembled plasmids can be used repeatedly many times without extra manipulations and costs such as PCR, restriction or GG reaction. Considering all the above, we have developed a recombineering-based approach, which is easier in implementation than other cloning-based methods. This cloning-free technique requires less effort and time, while producing ready-to-use plasmids, and it is suitable for all yeast species regardless of specific peculiar recombination machinery (Supplementary Table 4).

Supplementary Table 4 Comparison of alternative approaches of Cas9-helpers assembly

<table>
<tr><th>Assembly method</th><th>Day 1</th><th>Day 2</th><th>Day 3</th><th>Day 4</th><th>Total time spent</th><th>Reference</th></tr>
<tr><td colspan="6">Plasmid-required systems</td><td></td></tr>
<tr><td>GG assembly (with cloning)</td><td>● Phosphorylating oligos
● Annealing oligos
● GG assembly
● E. coli transformation</td><td>● Colony PCR
● Culturing and miniprep
● Yeast transformation</td><td rowspan="3">Yeast colony growth</td><td rowspan="3">Yeast colon y PCR</td><td rowspan="3">4 days</td><td>[10]</td></tr>
<tr><td>USER cloning</td><td>● Annealing oligos
● USER reaction
● E. coli transformation</td><td>● Colony PCR
● Culturing and miniprep
● Yeast transformation</td><td>[9]</td></tr>
<tr><td>Recombineering</td><td>● E. coli transformation</td><td>● Culturing and miniprep
● Restriction analysis
● Yeast transformation</td><td>this study</td></tr>
<tr><td colspan="6">Cloning-free system</td><td></td></tr>
<tr><td>GG assembly (cloning-free)</td><td>● PCR and purification
● GG assembly
● Yeast transformation</td><td>● Plating after recovery</td><td>Yeast colony growth</td><td>Yeast colon y PCR</td><td>4 days*</td><td>[13]</td></tr>
</table>

* - the time mentioned in original paper was 3 days, which was the time interval (from day 1 to day 4) and did not include the first experimental day

1.8 (Pro Module) Introduction of new promoters

The Pro Module is designed to build and test new *Y. lipolytica* promoters. The vector pProUA-mScarlet is required for GG assembly of new promoters (Supplementary Figure 24). In the same way as other genetic elements from the Lvl0 Module, promoters assembled on the pProUA-series can be used to assemble TUs on Lvl1 plasmids using BsaI (Supplementary Figure 14). However, in contrast to plasmids in the Lvl0 Module, the pProUA-series contains humanized *Renilla* green fluorescent protein gene (*hrGFP*) which is codon-optimized for expression in *Y. lipolytica* and regulated by the promoter of interest. Such plasmids can be integrated into the standard IntC2 locus of the *Y. lipolytica* genome using specific Cas9-helper (pCasNA-IntC2) and the integration confirmed using *URA3* marker. Therefore, assembled pPro-series could be used for both purposes: for assembly of TUs and for promoter characterisation assays in the yeast (Section 2.6). As an example,

the sequences of primers used for cloning the *Y. lipolytica ALK1* promoter are shown in Supplementary Figure 25. For GG assembly, use Protocol 3.1 with BpiI enzyme and thermal inactivation. Clones are selected for on LB-Ap by lack of red fluorescence.

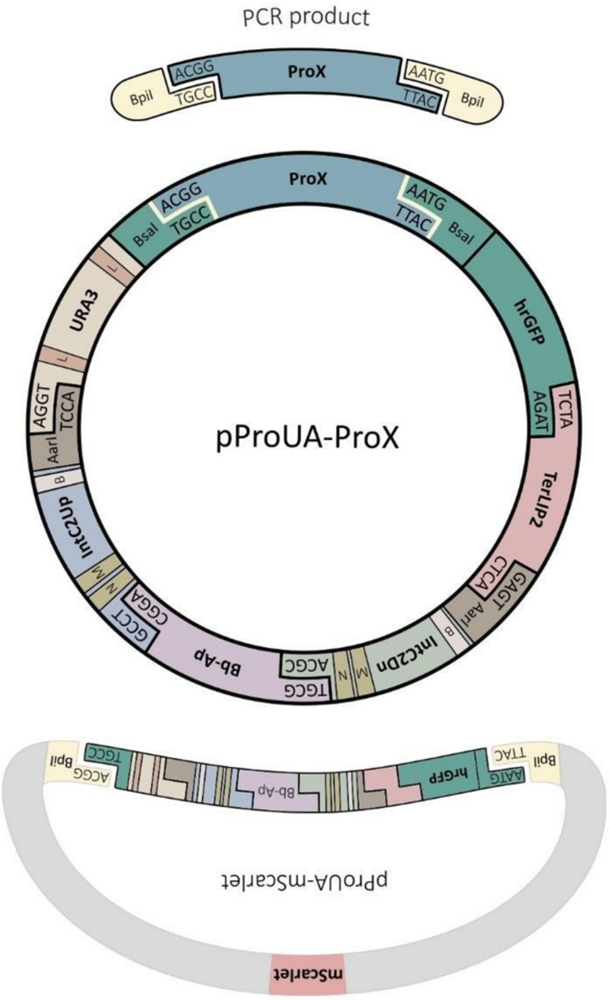

Supplementary Figure 24 Assembly of pProUA-series with new promoter. Note that the resultant plasmid contains two BsaI sites which enable assembly on a Lvl1 vector. Yellow indicates enzyme cut sites. Abbreviations: ProX – promoter to be screened; TerLIP2 – terminator of *LIP2* gene; IntC2Up, IntC2Dn – IntC2 upstream and downstream homology arms; B – barcode region; Bb-Ap – backbones with Ap-resistances; L – Lox sites; M – MssI sites; N – NotI sites.

ALK1-F	gcatGAAGACTCACGGACTGACTTGATACGCAACTG
ALK1-R	atgcGAAGACAGCATTGTGCAGGAGTATTCTGGGGAG
BpiI Start codon	

Supplementary Figure 25 Structure of primers for assembly of pProUA-series. Example here shows primers for cloning the *ALK1* promoter of *Y. lipolytica*. Lowercase are junk DNA to allow the Type IIs enzyme to cut. Underlined are 3'-terminal annealing regions that need to be redesigned when adding a new promoter.

Supplementary Note 2. YARROWIA ENGINEERING

YaliCraft provides the user with a high degree of freedom in the order of assembling genetic elements and eliminates the need to rebuild plasmids from scratch when switching between different integration approaches or alternative loci. A schematic representation of potential

iterations of marker-free and marker-based approaches is proposed in Supplementary Figure 26. As it can be seen from the estimated time periods, the marker-free technology presented here improves the rate of metabolic engineering in *Y. lipolytica* at least twofold when compared to approaches using integration of a single selectable marker, while at the same time allowing the user to return to a marker-based approach without extra assembly steps.

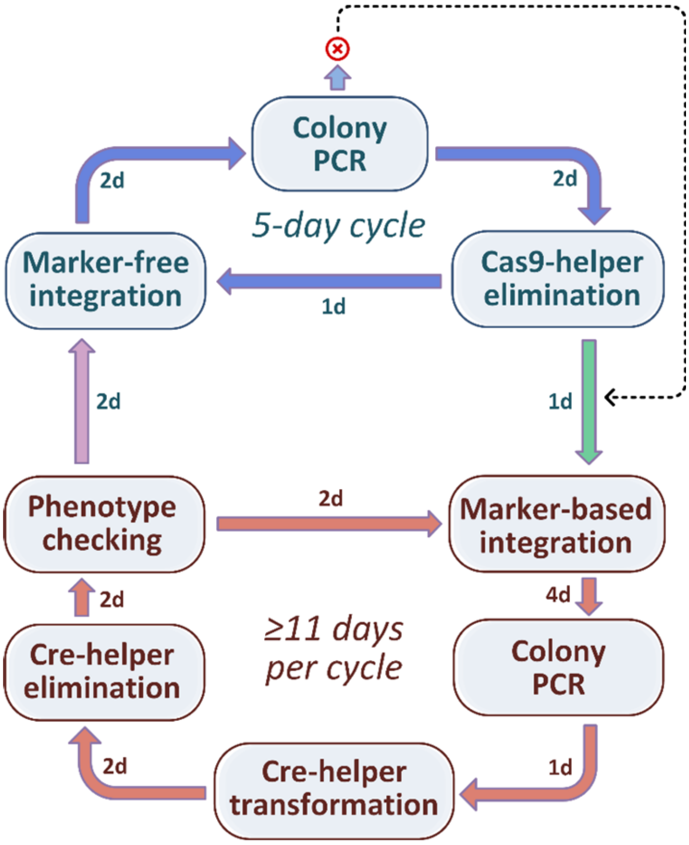

Supplementary Figure 26 Alternation of marker-free and marker-based integration techniques during *Y. lipolytica* engineering. Blue arrows, marker-free integration cycle. Red arrows, marker-based integration cycle. The marker-free cycle is less than half in duration and can be applied repeatedly as the main strategy. However, if no correct integrative transformants were isolated using this approach (red cross), then (dash line) marker-based construction needs to be transformed (green arrow). After the marker recovery procedure has been completed, the marker-free integration cycle can be restarted (purple arrow). The number of days required for each step and whole cycles are based on experimental procedures (Sections 2.2, 2.3 and 2.5).

2.1 Functional characterization of standard integration loci

Initially, 16 intergenic regions in *Y. lipolytica* genome were selected based on the following criteria: 1) located at least 1000 bp from the start codon and 500 bp from the stop codon of the nearest annotated open-reading frames, and 2) separated from the chromosome ends and from each other at least by 200 kb. The loci on each chromosome have been numbered and named accordingly as IntA1, IntA2, etc. They were chosen for designing homology arms (HA) 500-bp up and down the integration site. One extra set of integration flanks was designed with Zeta sequences instead of HAs. Zeta sequences do not show any homology to *Y. lipolytica* W29 and are usually used for random genomic integration.

For each integration locus, a set of five assembled empty vectors (Lvl1.1, Lvl1.2, Lvl1.3, Lvl2.2, and Lvl2.3) and Cas9-helper have been provided (Supplementary Table 8). To characterise each

locus, Lvl1.1 plasmid was assembled with a *hrGFP* reporter gene under the control of the *TEF1* promoter and *LIP2* terminator. Obtained plasmids were integrated as described in Section 2.6. Efficiency of Cas9-mediated integration together with other essential data for standard loci are summarized in Supplementary Table 5. The relative expression levels in different loci are provided on the Supplementary Figure 27. Notably, in our case, the 'position effect' changes gene expression levels less than two times, which is very similar to previously published data for *Y. lipolytica*[9].

Supplementary Table 5 Summarized data about 16 standard integration loci and corresponding homology arms

Integration locus	Chromosomal sequence GenBank#	IntUp homology arm position on the chromosome	IntDn homology arm position on the chromosome	Left flank integration primer (product with second primer Barcode1-R)	Right flank integration primer (product with second primer Barcode2-F)	20bp of sgRNA used on Cas9-helper	Enzyme for vector linearization	Integration efficiency % (number of correct clones among tested)
**IntB8**	CP017554.1	1280877..1281379	1281771..1282286	IntB8-chr-F (716bp)	IntB8-chr-R (756bp)	AAGTTCAAGATACTATACCC	MssI/NotI	75% (3/4)
**IntB11**	CP017554.1	2408220..2408818	2409833..2410434	IntB11-chr-F (813bp)	IntB11-chr-R (918bp)	AATGTTTGAGAGACAACGAC	SmiI/NotI	63% (5/8)
**IntC2**	CP017555.1	161071..161570	162857..163356	IntC2-chr-F (594bp)	IntC2-chr-R (601bp)	TCAAAAGTCAGTGTGAGGGG	MssI/NotI	75% (6/8)
**IntC7**	CP017555.1	1095374..1095882	1096160..1096676	IntC7-chr-F (685bp)	IntC7-chr-R (665bp)	TTATGAGACCCGATACAAGG	SmiI/NotI	50% (3/6)
**IntC13**	CP017555.1	2646315..2646874	2647563..2648077	IntC13-chr-F (713bp)	IntC13-chr-R (826bp)	AGACATGATGCATATACACA	SmiI/NotI	63% (5/8)
**IntC14**	CP017555.1	3133312..3133958	3134647..3135244	IntC14-chr-F (852bp)	IntC14-chr-R (858bp)	TCTCCATAGGTGTAGAACCA	SmiI/NotI	38% (3/8)
**IntD6**	CP017556.1	1494593..1495114	1495784..1496287	IntD6-chr-F (776bp)	IntD6-chr-R (696bp)	AAGCTTAATAAACAGCAAAG	NotI	75% (6/8)
**IntD12**	CP017556.1	2194396..2194895	2195646..2196145	IntD12-chr-F (595bp)	IntD12-chr-R (607bp)	GGTGGTGGTTATAATCGCTG	MssI/NotI	63% (5/8)
**IntE6**	CP017557.1	895599..896101	896482..896990	IntE6-chr-F (690bp)	IntE6-chr-R (698bp)	TACGAGTAGAGACGTAAACG	SmiI/NotI	67% (2/3)
**IntE8**	CP017557.1	1739277..1739776	1740717..1741216	IntE8-chr-F (589bp)	IntE8-chr-R (597bp)	AGGATAGTGCTTGTGTCCAG	SmiI/NotI	75% (3/4)
**IntE12**	CP017557.1	2831926..2832470	2832716..2833196	IntE12-chr-F (881bp)	IntE12-chr-R (1033bp)	ACCCCTATAGCCCAAACTGT	SmiI/NotI	63% (5/8)
**IntE15**	CP017557.1	3368514..3369040	3369472..3369975	IntE15-chr-F (715bp)	IntE15-chr-R (712bp)	TGCACTCTACAGGAACACTC	SmiI/NotI	75% (6/8)
**IntE16**	CP017557.1	3975493..3976035	3976233..3976833	IntE16-chr-F (817bp)	IntE16-chr-R (815bp)	TACTACTTGTAGTAGGACCA	SmiI and MssI/NotI	100% (8/8)
**IntF8**	CP017558.1	2626127..2626644	2627341..2627851	IntF8-chr-F (743bp)	IntF8-chr-R (720bp)	AGAGATCTATATGGTTAACG	MssI/NotI	63% (5/8)
**IntF9**	CP017558.1	2834729..2835240	2836124..2836625	IntF9-chr-F (814bp)	IntF9-chr-R (628bp)	CACGAAGAGAGGTAAAACAG	SmiI/NotI	100% (8/8)
**IntF11**	CP017558.1	3458258..3458790	3459091..3459592	IntF11-chr-F (799bp)	IntF11-chr-R (711bp)	AGTGGTATGGCTCTTCACAC	MssI/NotI	50% (2/4)

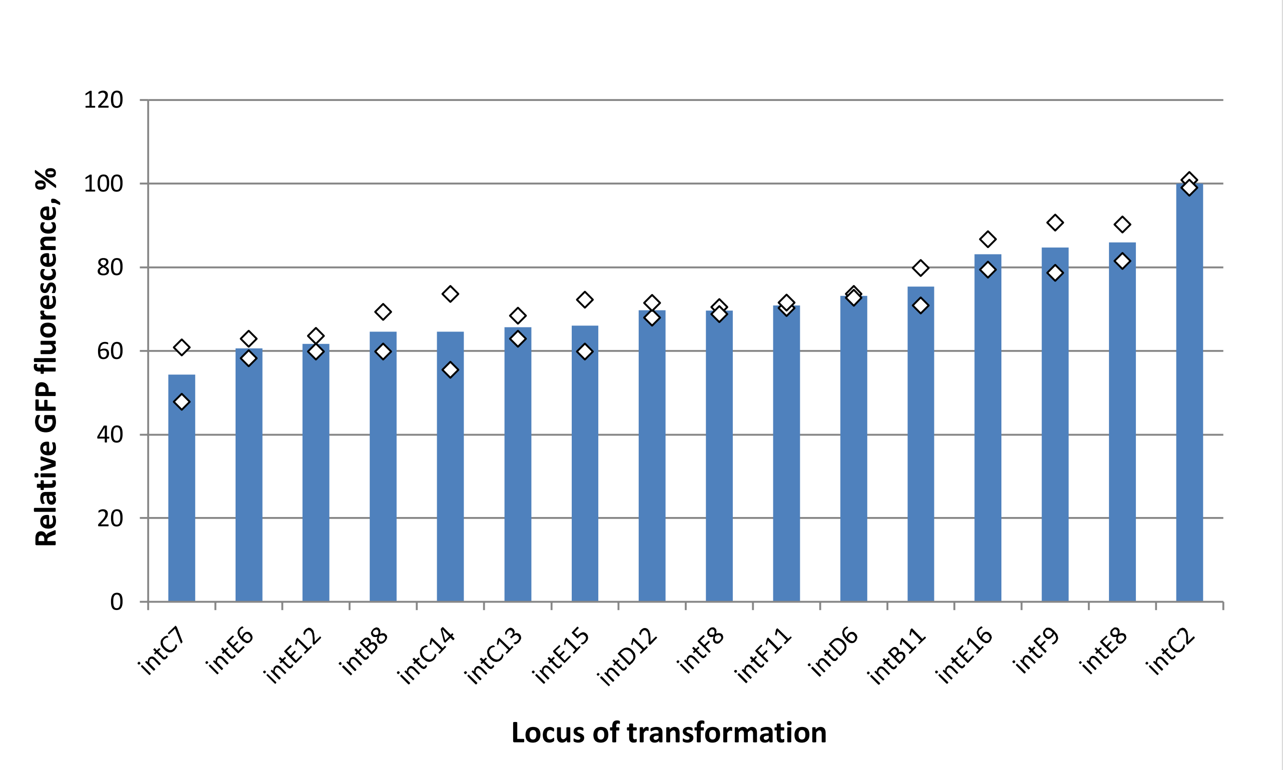

Supplementary Figure 27 Comparison of *hrGFP* expression under the control of *TEF1* promoter and *LIP2* terminator in 16 standard integration loci. The fluorescence (528 nm) was measured for cells growing in YPD medium in a plate reader after excitation at 485 nm. The values were normalized on biomass (OD_{600}) and auto-fluorescence of the parental strain S234 was extracted. Source data are provided as Supplementary Data 1.

2.2 Marker-free CRISPR/Cas9-mediated integration

Once a vector with the appropriate integrating HA has been chosen, the user can proceed to the plasmid construction. It is recommended to construct both marker-based and marker-free vectors. Using the marker-free construct is suggested to be attempted first given its shorter transformation cycle. The Cas9-helper (encoding Cas9 and guide) and integration construct (donor) need to be isolated in amounts sufficient for transformation. As an aside, we recommend verifying the plasmid concentrations based on gel electrophoresis, rather than solely on spectrophotometry. Spectrometry allows estimate total amount of DNA along with other impurities, while the fragment of interest may comprise a small portion in the mix or even be destroyed by nucleases, such as endonuclease A. The donor plasmid needs to be linearized using a restriction enzyme (Supplementary Tables 3 and 5), followed by purification and concentration using a gel purification kit (*e.g.* Zymoclean D4002, Cambridge Bioscience). The gel purification of integrating fragment is not required.

A single transformation requires 500ng of undigested Cas9-helper plasmid and between 500ng and 2000ng (larger constructs require more DNA) of integrative fragment that need to be mixed together in a total volume of 10-15 µL. The *Δku70* strain should be used for marker-free *Y. lipolytica* transformation. Examples of appropriate strains include W29ΔuraΔku70 or W29Δku70ura+ (Supplementary Table 9). The standard *Y. lipolytica* protocol needs to be applied for the transformation (Protocol 3.8). After heat shock and the single wash step, cells need to be plated on YPD with Nat (Section 4.1). Cells should be left to incubate for two days, after which several (3 to 8) large colonies are chosen and verified using colony PCR (Protocol 3.9). Some background growth of non-transformant clones can be also observed especially after prolonged incubation, however this mock colonies usually smaller than true transformants and can be distinguished based

on colony sizes. Standard primers for colony PCR are suggested for both left and right flanks for all sixteen integration loci (Supplementary Tables 5 and 10). If pDel-series was used, two new primers need to be designed corresponding to chromosomal regions flanking the insertion.

Successfully verified colonies then need to be streaked to single colonies and further incubated for 2 days on YPD plates. It is important to use medium without antibiotic at this step, because longer exposure of culture to Nat increases the chance of selecting a resistant clone, as described below. In the majority of cases, this procedure leads to elimination of unstable episomal Cas9-helper. From this point, the whole marker-free integration procedure can be repeated with a new construct. Nonetheless, we recommend using two independent transformants for the next transformation round. This might be helpful in the case that a Nat-resistant mutant was isolated or transformant which carries the helper plasmid integrated in the genome. If this is the case, after two days' incubation, there would be a lawn instead of individual clones on the transformation plate. Overall, a single round of marker-free integration (from one transformation experiment to another) takes five days.

2.3 Marker-based integration

The marker-based integration approach needs to be applied if marker-free integration did not lead to isolation of correct transformants. In that case, the marker-based version of the same integrative construct can be used. First, the assembled overexpression or deletion plasmid needs to be linearized using a restriction enzyme (Supplementary Tables 3 and 5) followed by column purification. Then, the concentration of the target fragment needs to be verified, preferably by electrophoresis - a single transformation requires 500 ng. For this, use the standard *Y. lipolytica* protocol for transformation (Protocol 3.8). Transformed cells need to be plated on YNBDcas (Section 4.1) and incubated for two days at 30°C. YNBDcas, is minimal media with glucose supplemented with casamino acids which allows URA+ transformants to grow faster as well as support growth of transformants with detrimental phenotypes that cannot be isolated on media with glucose as the single source of carbon.

Next, to select successful transformants, single colony isolation needs to be applied using the same YNBDcas medium that requiring another two days of incubation. Correct integration of isolated transformants needs to be verified by colony PCR (Section 2.2). Overall, the marker-based integration procedure takes at least five days (from one transformation experiment to another). However, the resulting strain is prototrophic for uracil, so the *URA3* marker needs be removed before the next round of marker-based integration. The suggested marker recovery procedure is provided in Section 2.5.

2.4 Combining marker-based and Cas9-mediated integration

To stimulate homologous recombination, reduce the fraction of transformants with off-target integration and improve the strength of selection, marker-based integration can be combined with CRISPR/Cas9. This approach might be useful for introducing difficult modifications that cannot be isolated using other techniques due to detrimental phenotypes.

Briefly, both DNAs, Cas9-helper and integration construct (donor), need to be prepared and transformed in *Y. lipolytica* as described in Section 2.2, with the only difference that donor fragment needs to include *URA3* marker. After the heat shock and a single washing step, transformed cells need to be incubated overnight in 2 mL of liquid YPD-Nat. The cell culture then needs to be washed with saline solution and plated on YNBDcas in serial dilutions. Transformants need to be verified by colony PCR (Section 2.2) and single colonies need to be isolated on YPD. Elimination of the Cas9-helper can be confirmed by comparing the growth on YPD medium with and without Nat. For this purpose, several colonies (3 to 8) along with the parental strain as control need to be restreaked to single colonies on YPD plates without Nat to promote elimination of the Cas9-helper. After two days,

clones need to be picked and resuspended in 25 µL of sterile saline solution. 3 µL should be plated on each of the plates: YPD and YPD-Nat. Growth needs to be screened after two days. Strains of interest (without Cas9-helper) should grow only on YPD plate without Nat, in the same way the control strain does. Finally, the marker needs to be recovered using procedure described in Section 2.5.

It is noteworthy to mention that selection on minimal media by complementation of uracil auxotrophy does not work well immediately after co-transformation of the Cas9-helper and donor with the *URA3* marker (data not shown). In order to obtain *URA3* functional expression prolonged (*e.g.* overnight) incubation in rich media is required.

2.5 Selectable marker recovery

Lvl0 plasmid pYalC-URA3 contains URA3 flanked by Lox sites - Lox66 and Lox71. The Lox sites enable permanent removal of *URA3* from any derivative construct whether it is present in *Y. lipolytica* or bacteria overexpressing Cre recombinase. In the MEx module, excision in *EcoCre* is used for the isolation of marker-free constructs (Section 1.6). In yeast, episomal Cre-helper plasmid (pCreHA) is used for removing the *URA3* marker from constructs integrated into the *Y. lipolytica* genome. To achieve this, *Y. lipolytica* needs to be transformed with 500 ng of undigested pCreHA. Transformants need to be selected on YPD-Hyg-Urd (hygromycin and uridine). After two or three days of incubation, several colonies need to be restreaked to single colonies on YPD plates without Hyg to promote elimination of the Cre-helper. After another two days, clones need to be picked and resuspended in 25 µL of sterile saline solution. 3 µL should be plated on each of the plates containing different medium compositions (Supplementary Table 6). Growth needs to be screened after two days. Strains of interest should contain neither *URA3* marker (no growth without Urd) and pCreHA helper (no growth with Hyg). The same suspensions of cells can be used as seeding for next round of integrative transformation. Therefore, single procedure of marker elimination (from Cre-helper transformation to the next transformation experiment) takes at least six days.

Supplementary Table 6 Identification of *URA3* excision and pCreHA elimination based on phenotypic tests

**Genotype of the strain**		**Observed phenotype of the strain**			
URA3	pCreHA	YNBDcas	YNBDcas with Urd	YPD with Hyg	YPD
+	+	+	+	+	+
+	-	+	+	-	+
-	+	-	+	+	+
-	-	-	+	-	+

In some cases, longer incubation is required to assure both marker and helper have been eliminated. For this purpose, below we describe a two-step procedure to get rid of *URA3* marker first and then the pCreHA helper. That makes the protocol longer, but much more efficient. In this case the 3 transformants selected above on YPD-Hyg-Urd media should be picked into separate tubes with 2 mL of liquid YPD-Hyg-Urd. One loop of the overnight culture from each tube needs to be streaked to single colony on YPD plate. A single isolated colony needs to be picked by toothpick and streaked onto two different plates: YNBDcas first, then using the same toothpick on YNBDCas with Urd. Among each progeny of the 3 independent transformants described above, single clones, which shows growth on YNBDcas with Urd, but does not on YNBDcas, need to be selected. These 3 clones need to be seeded in tubes with 2 mL of liquid YPD. One loop of the overnight culture from each tube needs to be streaked to single colony on YPD plate. A single isolated colony needs to be

picked by toothpick and streaked onto two plates: YPD first, then using the same toothpick on YPD-Hyg. Clones that show no growth on YPD with Hyg are free of both *URA3* marker and pCreHA helper and can be used for the next integration round.

2.6 Promoter library screening

Any promoter assembled on the pProUA-mScarlet vector in the Pro Module (Section 1.8) can be functionally tested by *hrGFP* expression in IntC2 locus. This locus was selected because it allows high expression levels, allowing novel promoters to be compared in the same genetic environment (Supplementary Figure 27). Alternatively, any promoter with compatible overhangs on the Lvl0 plasmid (Supplementary Table 9) can be assembled on the empty pC2US1.1 vector with *hrGFP* gene (pGenC-hrGFP) and *LIP2* terminator (pTerC-LIP2). Both types of assembled plasmids can be used for *in vivo* promoter assay using following procedures.

For integration into the genome, the assembled pProUA-series plasmid needs to be linearized with MssI, purified and co-transformed together with episomal helper pCasNA-IntC2 (Supplementary Table 8) into the W29ΔuraΔku70 strain (Supplementary Table 9). All procedures should be performed as has been described for marker-free integration (Section 2.2). Once colonies have been formed on YPD-Nat, large transformants need to be picked and streaked on YNBDcas plate. In our hands, prototrophic clones isolated through such two-step selection procedure always contained marker-based construction into the correct IntC2 locus. The integration can be further confirmed by colony PCR (Protocol 3.9) using either IntC2-chr-F/Barcode1-R or Barcode2-F/IntC2-chr-R pairs of primers (Supplementary Table 10).

For promoter activity assay two independent transformants with each promoter need to be grown along with positive control, a strain containing the *TEF1* promoter, and negative control, W29Δku70ura+. Cultures should be grown in 2 mL of liquid YPD medium for 16 hours. The biomass needs to be precipitated, washed once with sterile saline solution and adjusted to OD_{600} 5.0. At this step, if identification of only strongest promoters is required, they can be selected visually using blue LED transillumination (*e.g.* DR46B, Clare Chemical Research) with orange filter (Supplementary Figure 28). If large library of promoters is analysed these seeding cultures can be stored at -80 ^{o}C using either 25% (v/v) glycerol or 8% (v/v) dimethylsulfoxide (DMSO) as a cryoprotectant. Note that glycerol induces the strongest catabolic repression in *Y. lipolytica*, therefore DMSO is preferred if the effects of other carbon sources than glycerol need to be studied[14]. Next, depending on the condition required these seedings need to be grown until the appropriate growth phase, following by green fluorescence measurement (*e.g.* excitation, 495 nm; emission, 535 nm) using either plate reader or flow cytometer. Using both controls, promoter strengths can be expressed in the scale where the parental strain W29Δku70ura+ (without GFP) corresponds to 0%, while the transformant with *TEF1* promoter is accepted as 100% of the activity.

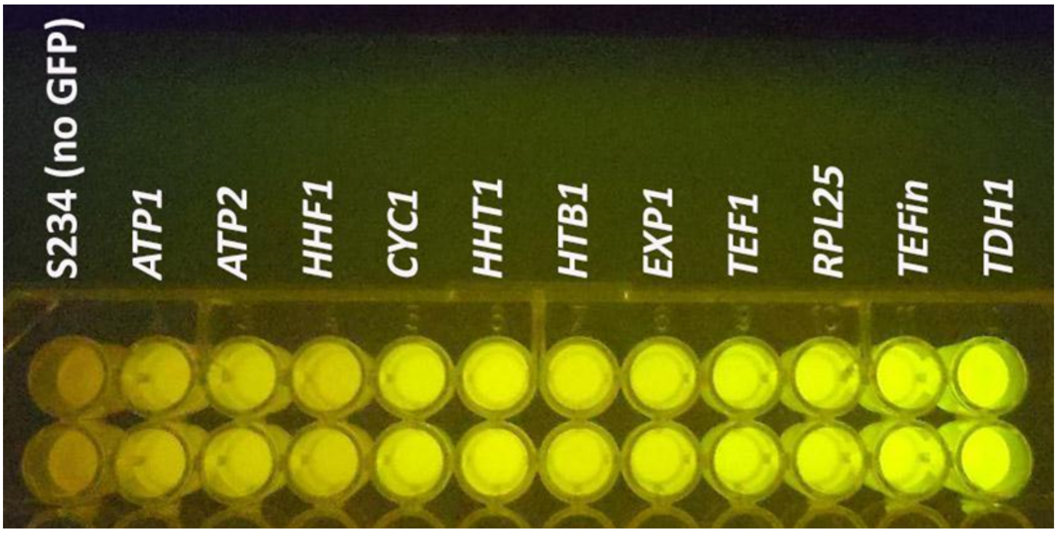

Supplementary Figure 28 Visual screening of promoter activities by hrGFP fluorescence in *Y. lipolytica* using pProUA-series. Comparison of different strength promoters is shown along with the parent strain S234 (W29Δku70ura+). Names of *Y. lipolytica* genes are shown that were used as the source of promoter sequences.

Supplementary Note 3. PROTOCOLS

3.1 GG reaction using BsaI/BsmBI/BpiI/AarI with thermal inactivation

1. Dilute all uncut plasmids to 50 nM.
2. Mix all other components, and then add enzymes as follows:

Component	For 10 µL Reaction
50 nM empty vector plasmid (backbone for assembly)	0.15 µL
50 nM other plasmids	0.5 µL
T4 DNA ligase buffer	1.0 µL
T7 DNA ligase	0.5 µL
Type IIs restriction enzyme (usually supplied as 10 U/µL)	0.5 µL
Nuclease-free water	to 10 µL

3. Place tube in thermocycler and run the following programme:

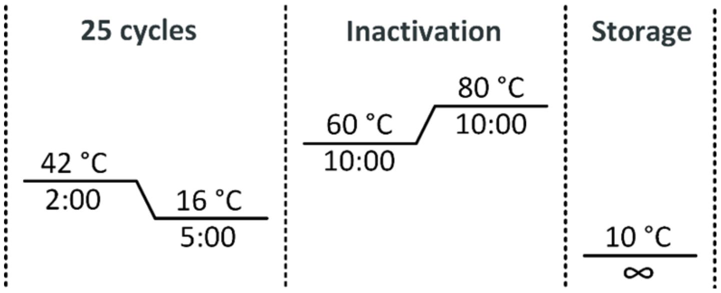

4. Add 20 ml of Nuclease-free water into a Petri dish and float a 0.025 µm MCE membrane (VSWP01300, Millipore) on the surface.
5. Drop 10 µL of GG reaction mixture on the membrane and let it dialyse for 15 min or more.
6. Collect dialyzed GG reaction mixture and electroporate in *E. coli* (Protocol 3.7).

(*for some GG reactions the efficiency of assembly and colony yield can be improved if concentration of plasmid and/or restriction enzyme are proportionally increased up to two times*)

3.2 GG reaction using AarI without thermal inactivation

1. Follow Protocol 3.1 until Step 3.
2. At the Step 3 use alternative thermocycler programme:

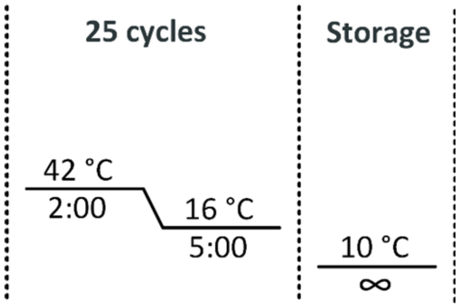

3.3 GG reaction using LguI with thermal inactivation

1. Follow Protocol 3.1 until Step 3.
2. At the Step 3 use alternative thermocycler programme:

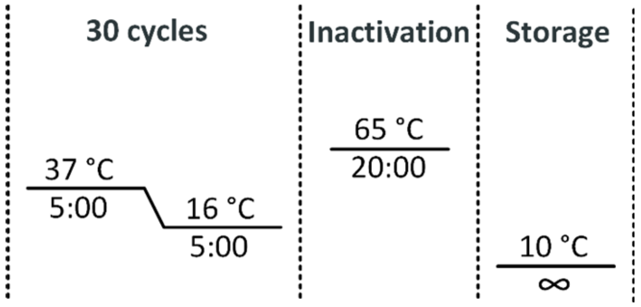

3.4 pDelUK-RG plasmid isolation

1. Seed 5 ml of LB-Km with a colony of transformed *E. coli* containing pDelUK-RG.
2. Use 500 µL of overnight culture to inoculate flask with 100mL of LB medium with Km.
3. Incubate at 37 ^{0}C with shaking (250 rpm) until the culture reaches OD_{600} 0.8
(the general rule for plasmid isolation from culture overexpressing fluorescent protein is collecting the biomass before the time point when bright colour is observed)
4. Collect the cells into a 50-mL tube by centrifuge at 10,000 x g for 6 min and remove the supernatant.
5. Refill the same tube again with leftover culture and repeat Step 4.
6. Add 2.5mL of Buffer P1 (#19051, Qiagen) into the tube and resuspend the cell pellet by vortexing.
(Ensure that RNase A has been added to Buffer P1)
7. Add 2.5mL Buffer P2 (#19052, Qiagen) and mix thoroughly by inverting the tube 4–6 times until the solution becomes viscous and slightly clear.
(Do not vortex, as this will result in shearing of genomic DNA)
8. Add 3.5mL Buffer N3 (#19052, Qiagen) and mix by inverting the tube 4–6 times.
9. Centrifuge for 10 min at 16,000 x *g* and transfer the supernatant to a fresh 50-mL tube with a pipette.
(try not to transfer white pellet and floating flakes)
10. Add 2mL of saturated solution of lithium chloride and vortex.
11. Add 14mL of isopropanol and vortex.
12. Centrifuge for 10 min at 16,000 x *g* and remove supernatant.
13. Spin shortly and remove the rest of the liquid using a pipette.
(Do not dry out the pellet)
14. Resuspend the pellet in small volume of water and transfer to 2-mL tube.
(use as little volume as possible)
15. Add 0.5 volumes of saturated lithium chloride solution and vortex.
16. Leave the tube at -20 ^{0}C for 20 min or longer.
17. Centrifuge the tube for 5 min at 13,000 x *g*.
18. Transfer supernatant by pipette to a new 2-mL tube.
19. Add 2.5 volumes of absolute ethanol and vortex.
20. Centrifuge for 7 min at 13,000 x *g* and remove the supernatant.
21. Spin shortly and remove the rest of the liquid using a pipette.
22. Dry the pellet at 37 ^{0}C for 15 min.
23. Dissolve the pellet in 50 µL of Buffer EB (#19086, Qiagen).
24. Check concentration by gel electrophoresis after restriction digest with NotI or SgrDI.
(usual concentration is 200 ng/µL)

3.5 *EcoRed* competent culture preparation

1. Seed *EcoRed* in a 250-mL flask containing 60mL of LB and grow overnight at 250 rpm and 30 ^{o}C. *(NB: EcoRed does not grow at 37 ^{o}C)*
2. Add 250 mL SOB medium to a 2L conical flask and inoculate with 25 mL of overnight *EcoRed* culture.
3. Grow with shaking (250 rpm, 30 ^{o}C) until the culture reaches an OD_{600} of 0.4-0.5.
4. Quickly transfer flasks into a pre-warmed shaking water bath (*e.g.* C76 NEWBM1248-0003, VWR) and incubate for 15 min at 42 ^{o}C with vigorous shaking.
5. Put flasks into an ice-bath, shake it gently to cool it down, and leave for 10 min. *(hereinafter, all steps should be done in the ice-bath)*
6. Pre-chill a large centrifuge (*e.g.* 5910R, Eppendorf) with an angle rotor and 50 mL tubes to 4 ^{o}C.
7. Centrifuge cells in 50-mL tubes for 10 min at 5,000 x *g* at 4°C and discard supernatant by pouring.
8. Refill the same tubes again and repeat the previous step.
9. Centrifuge tubes with the biomass for another 1 min at 5,000 x *g*.
10. Discard the rest of the supernatant by pipetting.
11. Add 1mL of ice-cold sterile 10% glycerol and gently resuspend the cell pellet by pipetting.
12. Add 30mL of ice-cold 10% glycerol and mix by inverting the tubes several times.
13. Centrifuge for 6 minutes at 5,000 x *g*.
14. Discard the supernatant by pouring.
15. Resuspend cells by pipetting in 500 µL of ice-cold 10% glycerol and transfer the suspension into a pre-chilled 1.5-mL tube.
16. Centrifuge the tube at 4°C in a pre-chilled centrifuge (*e.g.* Micro 17R, Thermo Fisher) for 1 min at 8,000 x *g*.
17. Pipette out the supernatant without disrupting the pellet.
18. Adjust cells to 500 µL by gradually adding 10% glycerol. *(the final volume of competent culture should be 1% of the original culture in SOB medium)*
19. Resuspend cells gently by pipetting.
20. Make 25 µL aliquots of competent *EcoRed* cells in pre-chilled tubes placed on ice.
21. Store the aliquots at -80 ^{o}C until use for electroporation.

3.6 *EcoCre* competent culture preparation

1. Add 60 ml of LB into a 250 ml flask and inoculate with *EcoCre*.
2. Grow overnight at 250 rpm and 37 ^{o}C.
3. Add 250 ml of SOB into two 2L flasks and inoculate each flask with 25 mL of overnight *EcoCre* culture.
4. Grow with shaking at 37 ^{o}C (250 rpm) until culture reaches OD_{600} 0.6.
5. Continue using Protocol 3.5 starting from the Step 5.

3.7 *EcoRed* and *EcoCre* electroporation

1. Take a 25-µL aliquot of competent cells from -80 ^{o}C and defrost on ice for 10 mins.
2. Cool a 1-mm electroporation cuvette on ice for 10 minutes.
3. Add DNA of interest directly to the tube containing competent cells and mix by pipetting gently.
4. Transfer mixture into the electroporation cuvette placing it between the electrodes.
5. Wipe the sides of the cuvette with a clean paper towel to avoid arcing.
6. Electroporate at 1800V in Eppendorf Eporator or equivalent under the setting recommended by manufacturer for bacteria.
(the time constant should be between 3-6 ms)
7. Immediately after the pulse, add 1 mL of SOB into the cuvette.
8. Mix once only by pipetting and transfer to 1.5-mL tube.
9. Incubate with shaking for 1 hour at 30 ^{o}C.
10. Plate on LB agar with appropriate antibiotics and grow at 30 ^{o}C overnight.
(for EcoCre culture different dilutions need to be plated depending on the assembly type. For EcoRed all transformed cells need to be plated)
11. All following cultivation of transformed cells need to be performed at 30 ^{o}C to prevent induction of recombination proteins.

3.8 *Y. lipolytica* transformation

(adapted from Chen et al.[15])

1. Streak *Y. lipolytica* strain to single colony from glycerol stock onto a YPD plate until single colonies can be seen.
2. Resuspend a single colony in 100 µL of sterile saline solution and spread it on YPD plate to grow overnight.
(if the strain is uracil auxotroph, supplying YPD with Urd)
3. Prepare transformation mixture in a 2-mL tube by combining:

Component	For 100 µL of Transformation Mixture
50% PEG 4000	90 µL
2M lithium acetate (pH 6.0)	5 µL
2M DTT	5 µL

and mix by pipetting.
4. From the overnight plate, pick a small loop of biomass and resuspend it in the transformation mixture.
(this amount of biomass visually correspond to the size of a match head and for the W29 strain it is equal to 1 mL of cell suspension with an OD_{600} of 10)
5. Boil 5 µL of salmon sperm DNA (#15632011, Thermo Fisher) for 8 mins at 95 ^{o}C, add to the mixture and mix with the loop.
6. Add ≤15 µL of transforming DNA to the mixture and mix with the loop. The preferred concentration of DNA varies with the protocol and has been described elsewhere. When the DNA must be digested or linearized (e.g. NotI), this should have been done before this step.
7. Incubate 30 min at 30 ^{o}C with moderate shaking.
8. Then heat shock for 30 min at 39 ^{o}C in a water bath.
9. Dilute the transformation mix with 1 mL of sterile saline solution and gently vortex.
10. Centrifuge the biomass for 2 min at 6,000 x *g*.
11. Remove the supernatant by pipette.
12. Carefully resuspend in 100 µL of saline solution by gently pipetting with a 1000-µL tip.
13. Plate on appropriate media.

3.9 *Y. lipolytica* colony PCR

(this protocol is appropriate for PCR products between 200-900 bp. Amplification of bigger fragments requires isolation of genomic DNA before colony PCR)

1. Pick a single colony of *Y. lipolytica* from freshly grown plate using a pipette tip and resuspend in 25 µL of sterile water.
2. Prepare a 1.1X PCR master mix using 2X Phire Plant Direct PCR Master Mix (#F-160, Thermo Fisher) and both primers with 0.5 µM final concentration.

For example, for 100 µL of PCR prepare 90 µL of 1.1X PCR master mix as follow:

Component	For 90 µL of 1.1X PCR master mix
2X Phire Plant Direct PCR Master Mix	50 µL
100 µM forward primer	0.5 µL
100 µM reverse primer	0.5 µL
Nuclease-free water	39 µL

3. Add 9 µL of 1.1X PCR master mix into a PCR tube.
4. Add 1 µL of cell suspension to the reaction mix for a total reaction volume of 10 µL.
5. Place tubes in a thermocycler and run the following programme:

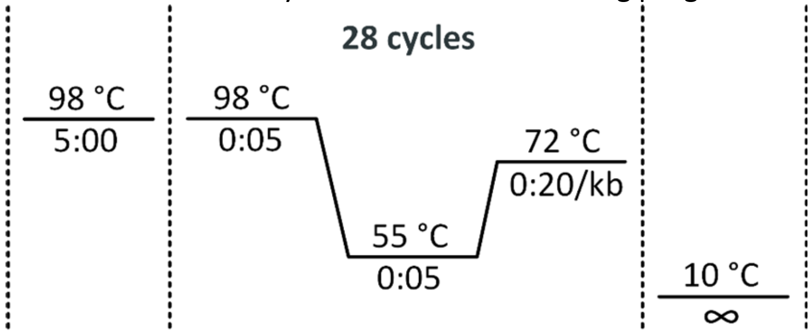

6. Run all of the reaction mixes in a 1% agarose gel.

Supplementary Note 4. APPENDIX
4.1 Media recipes

Supplementary Table 7 List of media supplements

Abbreviated name	Full name	Final concentration (µg/mL)
Ap	Ampicillin	150
Km	Kanamycin	100
Cm	Chloramphenicol	10
St	Streptomycin	1000
Sp	Spectinomycin	75
Nat	Nourseothricin	250
Hyg	Hygromycin	450
Urd	Uridine*	300

* – Uridine is preferred to uracil, because of its better solubility.

Saline solution
Prepare 0.85% sodium chloride solution and autoclave.

SOB
1. Mix and autoclave the components below to make SOB(Mg-):
    20 g/L tryptone from casein
    5 g/L yeast extract
    0.6 g/L NaCl (sodium chloride)
    0.2 g/L KCl (potassium chloride)
2. Mix and autoclave the components below to make the Magnesium Mix:
    0.5M $MgCl_2$ (magnesium chloride)
    0.5M $MgSO_4$ (magnesium sulfate)
3. For 1L of SOB(Mg-) from Step 1, add 20mL of Magnesium Mix from step 2 under sterile conditions.

YPD
1. Mix and autoclave the components below:
    20 g/L tryptone from casein
    10 g/L yeast extract
2. Filter sterilize 400 g/L glucose solution.
3. Add glucose to final concentration 20 g/L under sterile conditions.
(other carbon sources can be applied instead of glucose).

YNBDcas
1. Mix and autoclave the components below:
    6.7g/L yeast nitrogen base without amino acids (Y0626, Sigma)
    4g/L casamino acids
2. Filter sterilize 400 g/L glucose solution.
3. Add glucose to final concentration 20 g/L under sterile conditions.
(other carbon sources can be applied instead of glucose).

4.2 List of plasmids

Supplementary Table 8 List of *YaliCraft* plasmids (*continued on the next page*)

Addgene Number	Module	Plasmid Name	Plasmid Type	*E. coli* Marker	*E. coli* Fluorescence	Used for assembly	*Y. lipolytica* Marker	Application in Yali Module
175709	Lvl0	pYTK001	empty Lvl0	Cm	GFP	Lvl0		
175690	Lvl0	pYalA-Bb	Lvl0, Backbone	Ap	GFP	preLvl		
175711	Lvl0	pYalC-Bb	Lvl0, Backbone	Cm	GFP	preLvl		
175727	Lvl0	pYalK-Bb	Lvl0, Backbone	Km	GFP	preLvl		
175731	Lvl0	pYalS-Bb	Lvl0, Backbone	Sp	GFP	preLvl		
175712	Lvl0	pYalC-URA3	Lvl0, Selectable Marker	Cm		preLvl	URA3	
175710	Lvl0	pYalC-mCherry	Lvl0, *E.coli* RFP	Cm	RFP	preLvl		
175639	Lvl0	pYalC-IntB11Up	Lvl0, Homology Arm	Cm		preLvl		
175640	Lvl0	pYalC-IntB11Dn	Lvl0, Homology Arm	Cm		preLvl		
175715	Lvl0	pYalC-IntC2Up	Lvl0, Homology Arm	Cm		preLvl		
175716	Lvl0	pYalC-IntC2Dn	Lvl0, Homology Arm	Cm		preLvl		
175641	Lvl0	pYalC-IntC13Up	Lvl0, Homology Arm	Cm		preLvl		
175642	Lvl0	pYalC-IntC13Dn	Lvl0, Homology Arm	Cm		preLvl		
175717	Lvl0	pYalC-IntD12Up	Lvl0, Homology Arm	Cm		preLvl		
175718	Lvl0	pYalC-IntD12Dn	Lvl0, Homology Arm	Cm		preLvl		
175643	Lvl0	pYalC-IntE6Up	Lvl0, Homology Arm	Cm		preLvl		
175644	Lvl0	pYalC-IntE6Dn	Lvl0, Homology Arm	Cm		preLvl		
175719	Lvl0	pYalC-IntE8Up	Lvl0, Homology Arm	Cm		preLvl		
175720	Lvl0	pYalC-IntE8Dn	Lvl0, Homology Arm	Cm		preLvl		
175645	Lvl0	pYalC-IntE15Up	Lvl0, Homology Arm	Cm		preLvl		
175646	Lvl0	pYalC-IntE15Dn	Lvl0, Homology Arm	Cm		preLvl		
175647	Lvl0	pYalC-IntF8Up	Lvl0, Homology Arm	Cm		preLvl		
175648	Lvl0	pYalC-IntF8Dn	Lvl0, Homology Arm	Cm		preLvl		
175649	Lvl0	pYalC-IntF11Up	Lvl0, Homology Arm	Cm		preLvl		
175650	Lvl0	pYalC-IntF11Dn	Lvl0, Homology Arm	Cm		preLvl		
175713	Lvl0	pYalC-ZetaUp	Lvl0, Homology Arm	Cm		preLvl		
175714	Lvl0	pYalC-ZetaDn	Lvl0, Homology Arm	Cm		preLvl		
175691	Lvl0	pYalA-sfGFP1.1	Lvl0, *E.coli* GFP	Ap	GFP	empty Lvl1		
175692	Lvl0	pYalA-sfGFP1.2	Lvl0, *E.coli* GFP	Ap	GFP	empty Lvl1		
175693	Lvl0	pYalA-sfGFP1.3	Lvl0, *E.coli* GFP	Ap	GFP	empty Lvl1		
175728	Lvl0	pYalK-sfGFP2.2	Lvl0, *E.coli* GFP	Km	GFP	empty Lvl2		
175729	Lvl0	pYalK-sfGFP2.3	Lvl0, *E.coli* GFP	Km	GFP	empty Lvl2		
175705	Pro	pProUA-mScarlet	empty ProUA-vector	Ap	RFP	Pro-series	URA3	
175721	Lvl0	pProC-TEF1	Lvl0, Pro-series	Cm		Lvl1		
175623	Pro	pProUA-EXP1	ProUA-series	Ap		Lvl1	URA3	
175624	Pro	pProUA-TDH1	ProUA-series	Ap		Lvl1	URA3	
175625	Pro	pProUA-FBA1	ProUA-series	Ap		Lvl1	URA3	
175722	Lvl0	pGenC-hrGFP	Lvl0, Gen-series	Cm		Lvl1		
175723	Lvl0	pTerC-LIP2	Lvl0, Ter-series	Cm		Lvl1		
175724	Lvl0	pTerC-ScADH1	Lvl0, Ter-series	Cm		Lvl1		
175725	Lvl0	pTerC-ScPGK1	Lvl0, Ter-series	Cm		Lvl1		
175726	Lvl0	pTerC-ScENO2	Lvl0, Ter-series	Cm		Lvl1		
175669	Exp	pB8US1.1	empty Lvl1.1	Sp	GFP	Lvl1	URA3	Integration IntB8
175670	Exp	pB8US1.2	empty Lvl1.2	Sp	GFP	Lvl1	URA3	Integration IntB8
175671	Exp	pB8US1.3	empty Lvl1.3	Sp	GFP	Lvl1	URA3	Integration IntB8
175651	Exp	pB11US1.1	empty Lvl1.1	Sp	GFP	Lvl1	URA3	Integration IntB11
175652	Exp	pB11US1.2	empty Lvl1.2	Sp	GFP	Lvl1	URA3	Integration IntB11
175653	Exp	pB11US1.3	empty Lvl1.3	Sp	GFP	Lvl1	URA3	Integration IntB11
175735	Exp	pC2US1.1	empty Lvl1.1	Sp	GFP	Lvl1	URA3	Integration IntC2
175736	Exp	pC2US1.2	empty Lvl1.2	Sp	GFP	Lvl1	URA3	Integration IntC2
175737	Exp	pC2US1.3	empty Lvl1.3	Sp	GFP	Lvl1	URA3	Integration IntC2
175672	Exp	pC7US1.1	empty Lvl1.1	Sp	GFP	Lvl1	URA3	Integration IntC7
175673	Exp	pC7US1.2	empty Lvl1.2	Sp	GFP	Lvl1	URA3	Integration IntC7
175674	Exp	pC7US1.3	empty Lvl1.3	Sp	GFP	Lvl1	URA3	Integration IntC7
175654	Exp	pC13US1.1	empty Lvl1.1	Sp	GFP	Lvl1	URA3	Integration IntC13
175655	Exp	pC13US1.2	empty Lvl1.2	Sp	GFP	Lvl1	URA3	Integration IntC13
175656	Exp	pC13US1.3	empty Lvl1.3	Sp	GFP	Lvl1	URA3	Integration IntC13
175675	Exp	pC14US1.1	empty Lvl1.1	Sp	GFP	Lvl1	URA3	Integration IntC14
175676	Exp	pC14US1.2	empty Lvl1.2	Sp	GFP	Lvl1	URA3	Integration IntC14
175677	Exp	pC14US1.3	empty Lvl1.3	Sp	GFP	Lvl1	URA3	Integration IntC14
175678	Exp	pD6US1.1	empty Lvl1.1	Sp	GFP	Lvl1	URA3	Integration IntD6
175679	Exp	pD6US1.2	empty Lvl1.2	Sp	GFP	Lvl1	URA3	Integration IntD6
175680	Exp	pD6US1.3	empty Lvl1.3	Sp	GFP	Lvl1	URA3	Integration IntD6
175738	Exp	pD12US1.1	empty Lvl1.1	Sp	GFP	Lvl1	URA3	Integration IntD12
175739	Exp	pD12US1.2	empty Lvl1.2	Sp	GFP	Lvl1	URA3	Integration IntD12
175740	Exp	pD12US1.3	empty Lvl1.3	Sp	GFP	Lvl1	URA3	Integration IntD12
175657	Exp	pE6US1.1	empty Lvl1.1	Sp	GFP	Lvl1	URA3	Integration IntE6
175658	Exp	pE6US1.2	empty Lvl1.2	Sp	GFP	Lvl1	URA3	Integration IntE6
175659	Exp	pE6US1.3	empty Lvl1.3	Sp	GFP	Lvl1	URA3	Integration IntE6
175741	Exp	pE8US1.1	empty Lvl1.1	Sp	GFP	Lvl1	URA3	Integration IntE8
175742	Exp	pE8US1.2	empty Lvl1.2	Sp	GFP	Lvl1	URA3	Integration IntE8
175743	Exp	pE8US1.3	empty Lvl1.3	Sp	GFP	Lvl1	URA3	Integration IntE8
175681	Exp	pE12US1.1	empty Lvl1.1	Sp	GFP	Lvl1	URA3	Integration IntE12

Addgene Number	Module	Plasmid Name	Plasmid Type	*E. coli* Marker	*E. coli* Fluorescence	Used for assembly	*Y. lipolytica* Marker	Application in Yali Module
175682	Exp	pE12US1.2	empty Lvl1.2	Sp	GFP	Lvl1	URA3	Integration IntE12
175683	Exp	pE12US1.3	empty Lvl1.3	Sp	GFP	Lvl1	URA3	Integration IntE12
175660	Exp	pE15US1.1	empty Lvl1.1	Sp	GFP	Lvl1	URA3	Integration IntE15
175661	Exp	pE15US1.2	empty Lvl1.2	Sp	GFP	Lvl1	URA3	Integration IntE15
175662	Exp	pE15US1.3	empty Lvl1.3	Sp	GFP	Lvl1	URA3	Integration IntE15
175684	Exp	pE16US1.1	empty Lvl1.1	Sp	GFP	Lvl1	URA3	Integration IntE16
175685	Exp	pE16US1.2	empty Lvl1.2	Sp	GFP	Lvl1	URA3	Integration IntE16
175686	Exp	pE16US1.3	empty Lvl1.3	Sp	GFP	Lvl1	URA3	Integration IntE16
175663	Exp	pF8US1.1	empty Lvl1.1	Sp	GFP	Lvl1	URA3	Integration IntF8
175664	Exp	pF8US1.2	empty Lvl1.2	Sp	GFP	Lvl1	URA3	Integration IntF8
175665	Exp	pF8US1.3	empty Lvl1.3	Sp	GFP	Lvl1	URA3	Integration IntF8
175687	Exp	pF9US1.1	empty Lvl1.1	Sp	GFP	Lvl1	URA3	Integration IntF9
175688	Exp	pF9US1.2	empty Lvl1.2	Sp	GFP	Lvl1	URA3	Integration IntF9
175689	Exp	pF9US1.3	empty Lvl1.3	Sp	GFP	Lvl1	URA3	Integration IntF9
175666	Exp	pF11US1.1	empty Lvl1.1	Sp	GFP	Lvl1	URA3	Integration IntF11
175667	Exp	pF11US1.2	empty Lvl1.2	Sp	GFP	Lvl1	URA3	Integration IntF11
175668	Exp	pF11US1.3	empty Lvl1.3	Sp	GFP	Lvl1	URA3	Integration IntF11
175732	Exp	pZUS1.1	empty Lvl1.1	Sp	GFP	Lvl1	URA3	Integration random
175733	Exp	pZUS1.2	empty Lvl1.2	Sp	GFP	Lvl1	URA3	Integration random
175734	Exp	pZUS1.3	empty Lvl1.3	Sp	GFP	Lvl1	URA3	Integration random
175609	Exp	pB8UA2.2	empty Lvl2.2	Ap	GFP	Lvl2	URA3	Integration IntB8
175610	Exp	pB8UA2.3	empty Lvl2.3	Ap	GFP	Lvl2	URA3	Integration IntB8
175597	Exp	pB11UA2.2	empty Lvl2.2	Ap	GFP	Lvl2	URA3	Integration IntB11
175598	Exp	pB11UA2.3	empty Lvl2.3	Ap	GFP	Lvl2	URA3	Integration IntB11
175696	Exp	pC2UA2.2	empty Lvl2.2	Ap	GFP	Lvl2	URA3	Integration IntC2
175697	Exp	pC2UA2.3	empty Lvl2.3	Ap	GFP	Lvl2	URA3	Integration IntC2
175611	Exp	pC7UA2.2	empty Lvl2.2	Ap	GFP	Lvl2	URA3	Integration IntC7
175612	Exp	pC7UA2.3	empty Lvl2.3	Ap	GFP	Lvl2	URA3	Integration IntC7
175599	Exp	pC13UA2.2	empty Lvl2.2	Ap	GFP	Lvl2	URA3	Integration IntC13
175600	Exp	pC13UA2.3	empty Lvl2.3	Ap	GFP	Lvl2	URA3	Integration IntC13
175613	Exp	pC14UA2.2	empty Lvl2.2	Ap	GFP	Lvl2	URA3	Integration IntC14
175614	Exp	pC14UA2.3	empty Lvl2.3	Ap	GFP	Lvl2	URA3	Integration IntC14
175615	Exp	pD6UA2.2	empty Lvl2.2	Ap	GFP	Lvl2	URA3	Integration IntD6
175616	Exp	pD6UA2.3	empty Lvl2.3	Ap	GFP	Lvl2	URA3	Integration IntD6
175698	Exp	pD12UA2.2	empty Lvl2.2	Ap	GFP	Lvl2	URA3	Integration IntD12
175699	Exp	pD12UA2.3	empty Lvl2.3	Ap	GFP	Lvl2	URA3	Integration IntD12
175601	Exp	pE6UA2.2	empty Lvl2.2	Ap	GFP	Lvl2	URA3	Integration IntE6
175602	Exp	pE6UA2.3	empty Lvl2.3	Ap	GFP	Lvl2	URA3	Integration IntE6
175700	Exp	pE8UA2.2	empty Lvl2.2	Ap	GFP	Lvl2	URA3	Integration IntE8
175701	Exp	pE8UA2.3	empty Lvl2.3	Ap	GFP	Lvl2	URA3	Integration IntE8
175617	Exp	pE12UA2.2	empty Lvl2.2	Ap	GFP	Lvl2	URA3	Integration IntE12
175618	Exp	pE12UA2.3	empty Lvl2.3	Ap	GFP	Lvl2	URA3	Integration IntE12
175603	Exp	pE15UA2.2	empty Lvl2.2	Ap	GFP	Lvl2	URA3	Integration IntE15
175604	Exp	pE15UA2.3	empty Lvl2.3	Ap	GFP	Lvl2	URA3	Integration IntE15
175619	Exp	pE16UA2.2	empty Lvl2.2	Ap	GFP	Lvl2	URA3	Integration IntE16
175620	Exp	pE16UA2.3	empty Lvl2.3	Ap	GFP	Lvl2	URA3	Integration IntE16
175605	Exp	pF8UA2.2	empty Lvl2.2	Ap	GFP	Lvl2	URA3	Integration IntF8
175606	Exp	pF8UA2.3	empty Lvl2.3	Ap	GFP	Lvl2	URA3	Integration IntF8
175621	Exp	pF9UA2.2	empty Lvl2.2	Ap	GFP	Lvl2	URA3	Integration IntF9
175622	Exp	pF9UA2.3	empty Lvl2.3	Ap	GFP	Lvl2	URA3	Integration IntF9
175607	Exp	pF11UA2.2	empty Lvl2.2	Ap	GFP	Lvl2	URA3	Integration IntF11
175608	Exp	pF11UA2.3	empty Lvl2.3	Ap	GFP	Lvl2	URA3	Integration IntF11
175694	Exp	pZUA2.2	empty Lvl2.2	Ap	GFP	Lvl2	URA3	Integration random
175695	Exp	pZUA2.3	empty Lvl2.3	Ap	GFP	Lvl2	URA3	Integration random
175730	Del	pDelUK-RG	empty Del-vector	Km	GFP/RFP	Del-series	URA3	
175706	Del	pDelUA-KU70	Del-series	Ap			URA3	Disruption Ku70
175708	Cas	pCasNA-RK	empty Cas9-helper	Ap Km		Cas-series	Nat	
175632	Cas	pCasNA-IntB8	Cas9-helper	Ap			Nat	Integration IntB8
175626	Cas	pCasNA-IntB11	Cas9-helper	Ap			Nat	Integration IntB11
175702	Cas	pCasNA-IntC2	Cas9-helper	Ap			Nat	Integration IntC2
175633	Cas	pCasNA-IntC7	Cas9-helper	Ap			Nat	Integration IntC7
175627	Cas	pCasNA-IntC13	Cas9-helper	Ap			Nat	Integration IntC13
175634	Cas	pCasNA-IntC14	Cas9-helper	Ap			Nat	Integration IntC14
175635	Cas	pCasNA-IntD6	Cas9-helper	Ap			Nat	Integration IntD6
175703	Cas	pCasNA-IntD12	Cas9-helper	Ap			Nat	Integration IntD12
175628	Cas	pCasNA-IntE6	Cas9-helper	Ap			Nat	Integration IntE6
175704	Cas	pCasNA-IntE8	Cas9-helper	Ap			Nat	Integration IntE8
175636	Cas	pCasNA-IntE12	Cas9-helper	Ap			Nat	Integration IntE12
175629	Cas	pCasNA-IntE15	Cas9-helper	Ap			Nat	Integration IntE15
175637	Cas	pCasNA-IntE16	Cas9-helper	Ap			Nat	Integration IntE16
175630	Cas	pCasNA-IntF8	Cas9-helper	Ap			Nat	Integration IntF8
175638	Cas	pCasNA-IntF9	Cas9-helper	Ap			Nat	Integration IntF9
175631	Cas	pCasNA-IntF11	Cas9-helper	Ap			Nat	Integration IntF11
175707	Yali	pCreHA	Cre-helper	Ap			Hph	Marker recovery

Complete set of 147 *YaliCraft* plasmids may be ordered as two 96 well plates. Deposit 80045 with plasmids #175597-175689 and deposit 80046 with plasmids #175690-175743.

4.3 List of strains

Supplementary Table 9 List of *YaliCraft* strains

VKPM Number	Strain name	Species (parent wt strain)	Genotype	Growth temperature
**B-14086**	*EcoRed*	*E. coli* (MG1655)	F^- *ilvG*$^-$ *rfb-50 rph-1* λ [*cI*^{857}*Δ(cro-attR)*] *rpsL*^{K43R}	30 ^oC
**B-14087**	*EcoCre*	*E. coli* (MG1655)	F^- *ilvG*$^-$ *rfb-50 rph-1* λ [*cI*^{857} *Δ(cro-attR) Δ(N-attL)::P1cre)*] *rpsL*^{K43R}	30 ^oC
**Y-4971**	W29Δura	*Y. lipolytica* (W29)	*Δura3*	30 ^oC
**Y-4972**	W29Δku70ura+	*Y. lipolytica* (W29)	*Δku70::URA3*	30 ^oC
**Y-4973**	W29ΔuraΔku70	*Y. lipolytica* (W29)	*Δura3 Δku70*	30 ^oC

VKPM - Russian National Collection of Industrial Microorganisms.
Modified lambda prophage sequences of both *EcoRed* and *EcoCre* strains are provided in GenBank format.

4.4 List of primers

Supplementary Table 10 List of standard *YaliCraft* primers (*continued on the next page*)

Number	Name	Sequence	Application
**3158**	sgRNA-seq-R	ATCTGGGCCTCGTGATACG	sgRNA sequencing on pCas-series
**3159**	sgRNA-seq-F	GAGTGGTAAATCGCCTTCTTG	sgRNA sequencing on pCas-series
**3142**	Barcode1-R	ACTATCGGTAGAGCCAATTAG	Left flank checking from any construct
**3143**	Barcode2-F	GAAGTTGGTGAAAACATTTACG	Right flank checking from any construct
**4256**	IntB8-chr-F	TGCAACACACGACAGCTATC	Left flank checking from locus IntB8
**4257**	IntB8-chr-R	AAATCTCTAGCATCTCCAACG	Right flank checking from locus IntB8
**4640**	IntB11-chr-F	ATCGGTTGCAAAACGCTGAC	Left flank checking from locus IntB11
**4641**	IntB11-chr-R	TGACCCTTCGTTACGGACC	Right flank checking from locus IntB11
**3176**	IntC2-chr-F	GTGTGTCCAACCAAAGTGAG	Left flank checking from locus IntC2
**3177**	IntC2-chr-R	GTCAATCACAACAATGTCTCTC	Right flank checking from locus IntC2
**3178**	IntC7-chr-F	ATATCACATTGCAGTCACTCC	Left flank checking from locus IntC7
**3179**	IntC7-chr-R	TACTCCTCTGCGACCCTAC	Right flank checking from locus IntC7
**4642**	IntC13-chr-F	CCATGTGTCTATAAGTACAGC	Left flank checking from locus IntC13
**4643**	IntC13-chr-R	TAACCCTGACAGTTTGATAGC	Right flank checking from locus IntC13
**4250**	IntC14-chr-F	AACTTTCTGAAACTTGCTTTCC	Left flank checking from locus IntC14
**4251**	IntC14-chr-R	AACCAAACACGTGATCAAAGG	Right flank checking from locus IntC14
**5005**	IntD6-chr-F	AGGTGGTCAATGAGTTGTCG	Left flank checking from locus IntD6
**5006**	IntD6-chr-R	TACGTGTGTTTGGCTGTCTC	Right flank checking from locus IntD6
**3164**	IntD12-chr-F	CTGGCAACCACACCGACTC	Left flank checking from locus IntD12
**3165**	IntD12-chr-R	TTTCAGATGTGGACAATTCGG	Right flank checking from locus IntD12
**4454**	IntE6-chr-F	ACTCAACTGTGAATACTTCGG	Left flank checking from locus IntE6
**4455**	IntE6-chr-R	TCCTGGTTAAAGGGGGTTTC	Right flank checking from locus IntE6
**3170**	IntE8-chr-F	TGGGGTGTGTACTGTACTTG	Left flank checking from locus IntE8
**3171**	IntE8-chr-R	ATCAGATCATGATTGGAGAGC	Right flank checking from locus IntE8
**4307**	IntE12-chr-F	CTGGATCTTCCCTCATGGC	Left flank checking from locus IntE12
**4308**	IntE12-chr-R	TCCCCGACGCGAGATTTTC	Right flank checking from locus IntE12
**4456**	IntE15-chr-F	ACCTACCTCCTTCTATCTCC	Left flank checking from locus IntE15
**4457**	IntE15-chr-R	TCTGTAAGCTGAAGCACATTC	Right flank checking from locus IntE15
**4254**	IntE16-chr-F	TCGTGCATTTCGAAACACAAG	Left flank checking from locus IntE16
**4255**	IntE16-chr-R	ATGTAACACGTTCAGACCAGT	Right flank checking from locus IntE16

Number	Name	Sequence	Application
**4850**	IntF8-chr-F	CCAAGAGTTGAATAATGTAACC	Left flank checking from locus IntF8
**4851**	IntF8-chr-R	ACCTAGACTACCGCACCAC	Right flank checking from locus IntF8
**4258**	IntF9-chr-F	TGCCGTCGAGCCACTGTC	Left flank checking from locus IntF9
**4958**	IntF9-chr-R	AGTTACTACAAGTAACCTGAAG	Right flank checking from locus IntF9
**4460**	IntF11-chr-F	ACCTGACTGTTTACATCCGC	Left flank checking from locus IntF11
**4461**	IntF11-chr-R	TCAAGTTCTTGCACTCTGTTG	Right flank checking from locus IntF11

5. SUPPLEMENTARY REFERENCES

1. Juretzek T, Le Dall M, Mauersberger S, Gaillardin C, Barth G, Nicaud J. Vectors for gene expression and amplification in the yeast *Yarrowia lipolytica*. *Yeast* **18**, 97-113 (2001).
2. Celinska E, Ledesma-Amaro R, Larroude M, Rossignol T, Pauthenier C, Nicaud JM. Golden Gate Assembly system dedicated to complex pathway manipulation in *Yarrowia lipolytica*. *Microbial biotechnology* **10**, 450-455 (2017).
3. Larroude M, Park YK, Soudier P, Kubiak M, Nicaud JM, Rossignol T. A modular Golden Gate toolkit for *Yarrowia lipolytica* synthetic biology. *Microbial biotechnology* **12**, 1249-1259 (2019).
4. He Q, Szczepanska P, Yuzbashev T, Lazar Z, Ledesma-Amaro R. *De novo* production of resveratrol from glycerol by engineering different metabolic pathways in *Yarrowia lipolytica*. *Metabolic engineering communications* **11**, e00146 (2020).
5. Horton RM, Hunt HD, Ho SN, Pullen JK, Pease LR. Engineering hybrid genes without the use of restriction enzymes: gene splicing by overlap extension. *Gene* **77**, 61-68 (1989).
6. Lee ME, DeLoache WC, Cervantes B, Dueber JE. A highly characterized yeast toolkit for modular, multipart assembly. *ACS synthetic biology* **4**, 975-986 (2015).
7. Damude HG*, et al.* High eicosapentaenoic acid producing strains of *Yarrowia lipolytica*. Patet Application US 2006115881 (2006).
8. Nicaud JM*, et al.* Protein expression and secretion in the yeast *Yarrowia lipolytica*. *FEMS yeast research* **2**, 371-379 (2002).
9. Holkenbrink C*, et al.* EasyCloneYALI: CRISPR/Cas9-based synthetic toolbox for engineering of the yeast *Yarrowia lipolytica*. *Biotechnology journal* **13**, e1700543 (2018).
10. Gowers GF*, et al.* Improved betulinic acid biosynthesis using synthetic yeast chromosome recombination and semi-automated rapid LC-MS screening. *Nature communications* **11**, 868 (2020).
11. Generoso WC, Gottardi M, Oreb M, Boles E. Simplified CRISPR-Cas genome editing for *Saccharomyces cerevisiae*. *Journal of microbiological methods* **127**, 203-205 (2016).
12. Horwitz AA*, et al.* Efficient multiplexed integration of synergistic alleles and metabolic pathways in yeasts via CRISPR-Cas. *Cell systems* **1**, 88-96 (2015).
13. Zhang Y*, et al.* A gRNA-tRNA array for CRISPR-Cas9 based rapid multiplexed genome editing in *Saccharomyces cerevisiae*. *Nature communications* **10**, 1053 (2019).
14. Yuzbasheva EY*, et al.* A metabolic engineering strategy for producing free fatty acids by the *Yarrowia lipolytica* yeast based on impairment of glycerol metabolism. *Biotechnology and bioengineering* **115**, 433-443 (2018).
15. Chen DC, Beckerich JM, Gaillardin C. One-step transformation of the dimorphic yeast *Yarrowia lipolytica*. *Applied microbiology and biotechnology* **48**, 232-235 (1997).

(This is an information page separating Supplementary Manual and Supplementary Experiments. The figure, table, and section numbering are continuous)

6. ASSESSING THE EFFICIENCY OF THE TOOLKIT

6.1 Pro Module

The efficiency of DNA assembly of new promoters was tested using *ALK1* promoter of *Y. lipolytica* as an example. The assembly was done following procedures described in the Sections 1.8 of Supplementary Manual. The DNA parts involved in the assembly are summarised in Supplementary Table 17. Plasmid DNA was isolated from 18 clones and restriction analysis was performed using BsaI enzyme (Supplementary Figure 29). The observed assembly efficiency of pProUA-ALK1 plasmid was 100% (18/18).

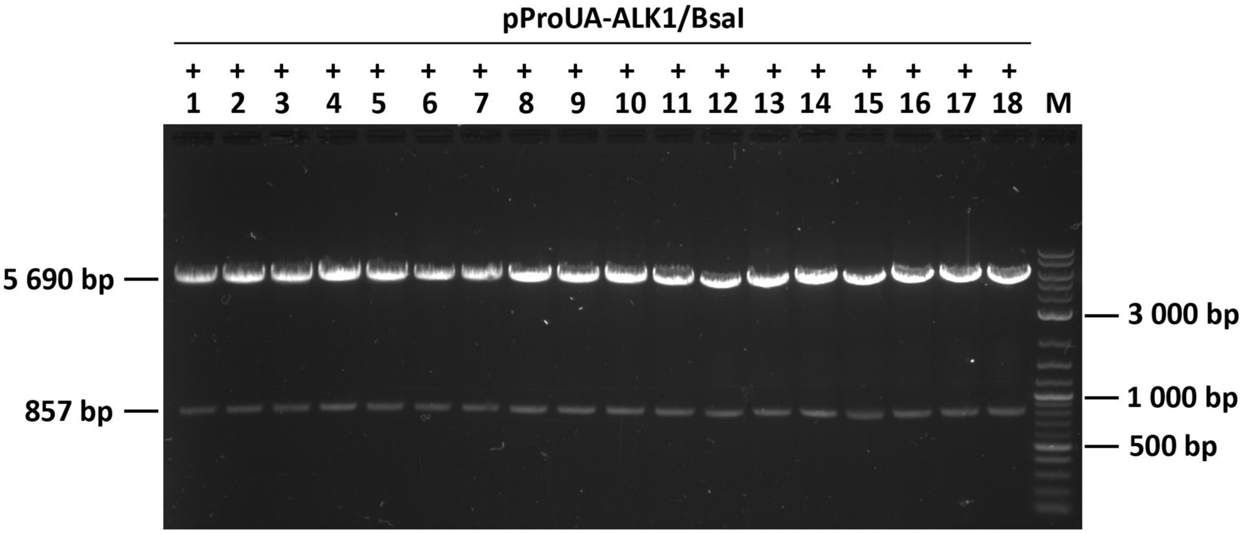

Supplementary Figure 29 The restriction analysis of clones isolated from pProUA-ALK1 assembly using BsaI. The restriction fragments corresponding to pProUA-ALK1 are shown on the left side. The major bands of the molecular weight marker NEB #B7025 (M) are shown on the right side. Correctly assembled clones are designated with pluses above the clone numbers.

6.2 Del Module

The DNA assembly efficiency of of pDel-series plasmids was tested using *AAT1* gene of *Y. lipolytica* as an example. The assembly was done following procedures described in the Sections 1.4 of Supplementary Manual. DNA parts involved in the assembly were summarised in Supplementary Table 17. Plasmid DNA was isolated from 18 clones and restriction analysis was performed using MssI enzyme (Supplementary Figure 30). The observed assembly efficiency of pDelUK-AAT1 plasmid was 50% (9/18).

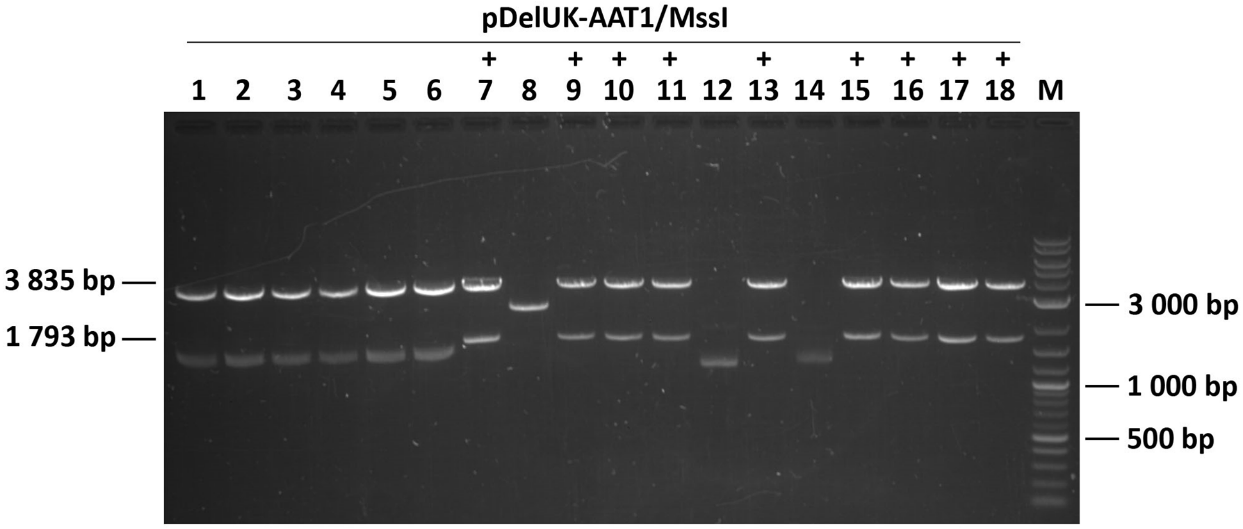

Supplementary Figure 30 The restriction analysis of clones isolated from pDelUK-AAT1 assembly using MssI. Restriction fragments corresponding to pDelUK-AAT1 are shown on the left side. Major bands of the molecular weight marker NEB #B7025 (M) are shown on the right side. The correctly assembled clones are designated by pluses above the clone numbers.

6.3 Int Module - exchange of homology arms between Lvl1 and Lvl2

The efficiency of homology arms exchange between Lvl1 and Lvl2 was tested using plasmids pE8US1.1 and pZUA2.3-HPD1-ARO4-ARO7 as an example (Supplementary Table 17). The assembly was done following procedures described in Sections 1.5.1 of Supplementary Manual. Plasmid DNA was isolated from 8 clones and restriction analysis was performed using PaqI enzyme (Supplementary Figure 31). The observed assembly efficiency of pE8US-HPD1-ARO4-ARO7 plasmid was 100% (8/8).

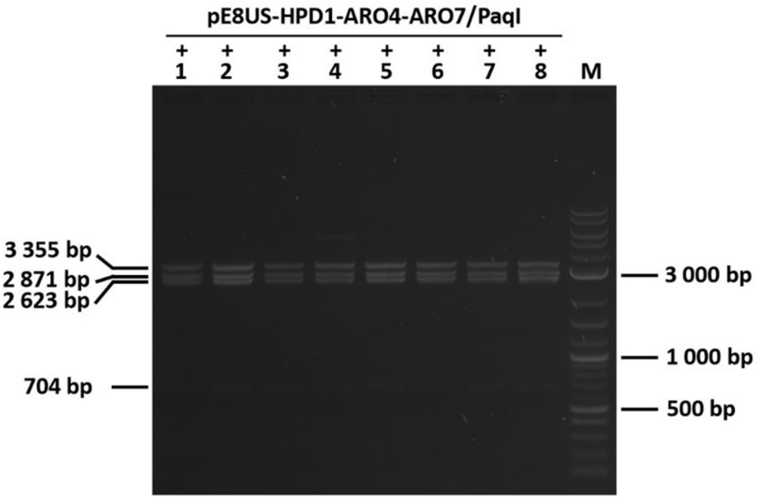

Supplementary Figure 31 The restriction analysis of clones isolated from pE8US-HPD1-ARO4-ARO7 assembly using PaqI. The restriction fragments corresponding to pE8US-HPD1-ARO4-ARO7 are shown on the left side. The major bands of the molecular weight marker NEB #B7025 (M) are shown on the right side. The correctly assembled clones are selected by pluses above the clone numbers.

6.4 Int Module - Transfer of homology arms from pDel-series to Lvl1 and Lvl2

The efficiency of homology arms transfer from pDel-series to Lvl1 and Lvl2 was tested using plasmids pE8US-HPD1-ARO4-ARO7 and pDelUK-AAT1 as an example (Supplementary Table 17). The assembly was done following procedures described in Sections 1.5.2 of Supplementary Manual. Plasmid DNA was isolated from 12 clones and restriction analysis was performed using MssI enzyme

(Supplementary Figure 32). The observed assembly efficiency of pDelUK-AAT1::HPD1-ARO4-ARO7 plasmid was 58.3% (7/12).

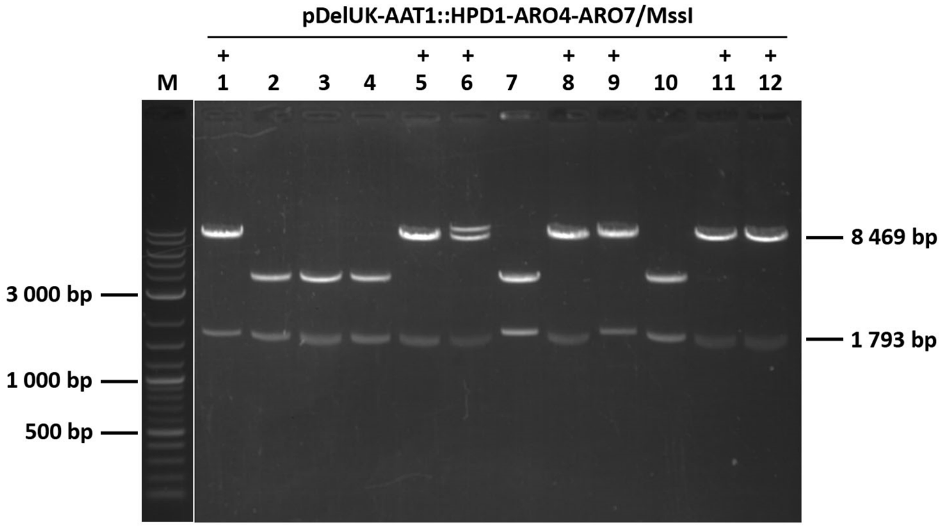

Supplementary Figure 32 The restriction analysis of clones isolated from pDelUK-AAT1::HPD1-ARO4-ARO7 assembly using MssI. Restriction fragments corresponding to pDelUK-AAT1::HPD1-ARO4-ARO7 are shown on the right side. Major bands of the molecular weight marker NEB #B7025 (M) are shown on the left side. The extra band of clone 6 above 8 469 bp supposed to be a fragment with size 10 262 bp corresponding to incomplete restriction of the plasmid pDelUK-AAT1::HPD1-ARO4-ARO7. Correctly assembled clones are selected by pluses above the clone numbers.

6.5 MEx Module

The efficiency of the yeast selectable marker excision was tested using plasmids pC2US1.1-hrGFP, pD12US1.1-hrGFP, and pE8US1.1-hrGFP as examples (Supplementary Table 17). The marker excision was done following procedures described in Sections 1.6 of Supplementary Manual. Plasmid DNA was isolated from 8 clones from each experiment and restriction analysis was performed using NotI enzyme (Supplementary Figure 33). The observed marker excision efficiency was 100% (24/24).

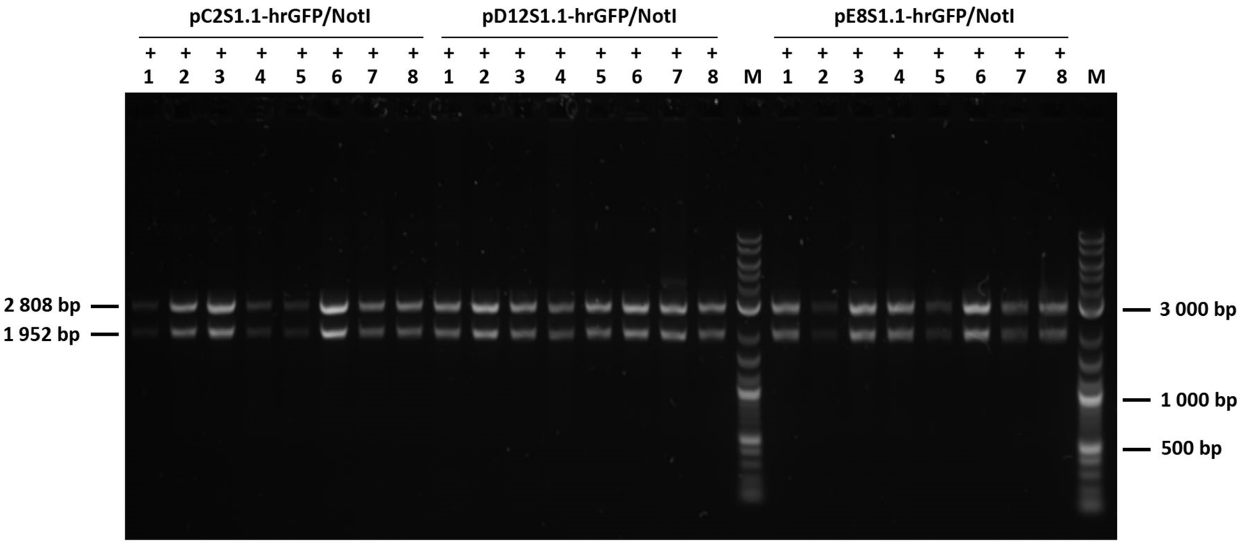

Supplementary Figure 33 The restriction analysis of clones isolated after marker excision from plasmids pC2US1.1-hrGFP, pD12US1.1-hrGFP, and pE8US1.1-hrGFP. The fragments corresponding NotI restriction of markerless plasmids pC2S1.1-hrGFP, pD12S1.1-hrGFP, and pE8S1.1-hrGFP are

shown on the left side. The major bands of the molecular weight marker NEB #B7025 (M) are shown on the right side. The correctly assembled clones are designated by pluses above the clone numbers.

6.6 Cas Module

The efficiency of DNA assembly of Cas9-helper plasmids was tested using three randomly generated 20-base recognition sequences as examples. The assemblies were done following procedures described in Sections 1.7 of Supplementary Manual. DNA parts involved in the assembly were summarised in Supplementary Table 17. Plasmid DNA was isolated from 34 clones and restriction analysis was performed using HindIII enzyme (Supplementary Figure 34). The observed assembly efficiency of plasmids pCasNA-Rdm1, pCasNA-Rdm2 and pCasNA-Rdm3 was 79.4% (27/34).

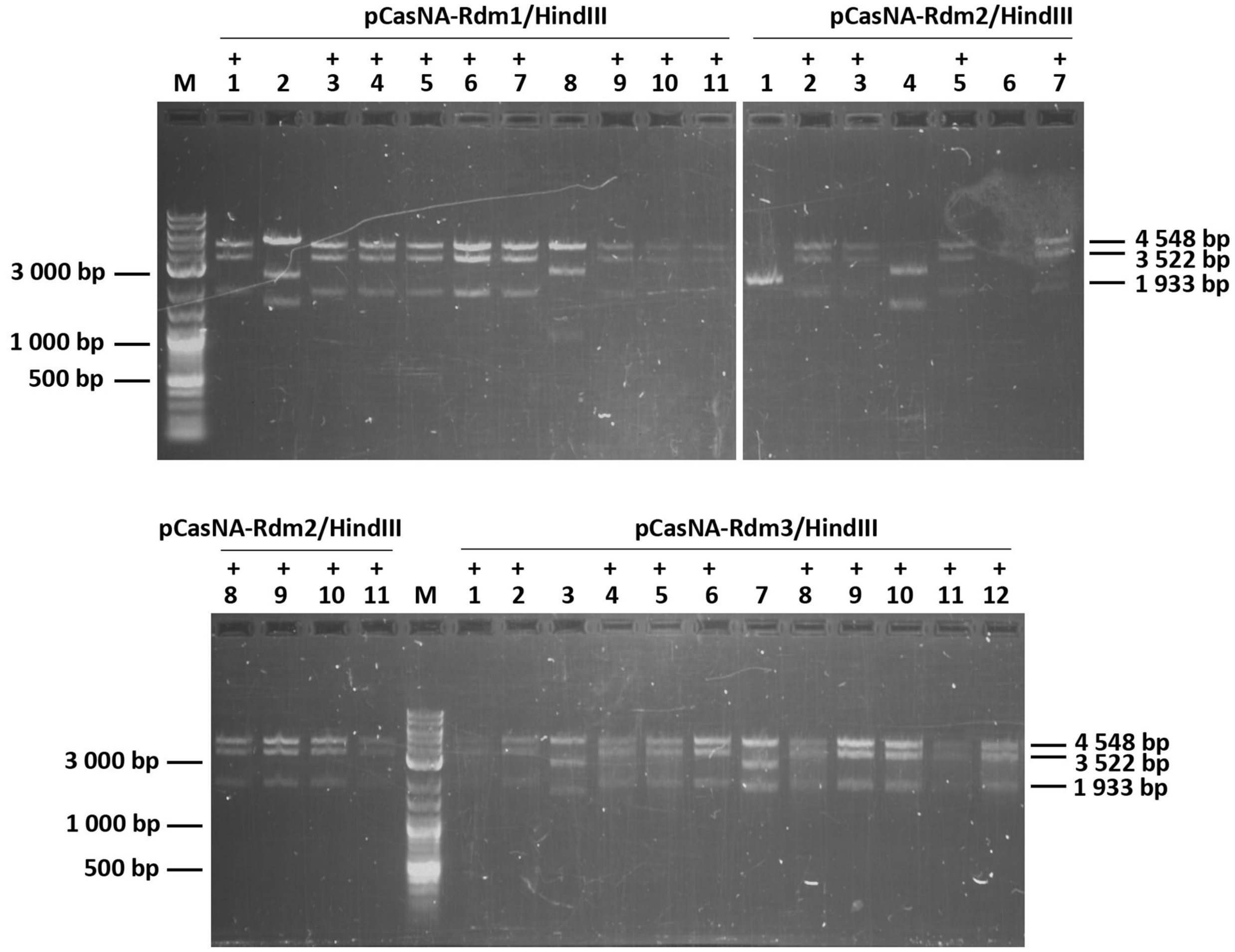

Supplementary Figure 34 The restriction analysis of clones isolated from pCasNA-Rdm1, pCasNA-Rdm2 and pCasNA-Rdm3 assemblies using HindIII. The restriction fragments corresponding to pCasNA-Rdm1, pCasNA-Rdm2 and pCasNA-Rdm3 are shown on the right side. The major bands of the molecular weight marker NEB #B7025 (M) are shown on the left side. The correct assemblies are designated by pluses above the clone numbers.

6.7 Assessing marker-free gene disruptions in *Y. lipolytica*

For assessing the efficiency of marker-free gene disruption we selected ARO8 and ARO9 as examples. For each gene, three alternative Cas9-helpers with different 20-base pair recognition sequences were constructed: pCasNA-ARO8a, pCasNA-ARO8c, pCasNA-ARO8i and pCasNA-ARO9a, pCasNA-ARO9i, pCasNA-ARO9k, respectively (Supplementary Table 17). Two marker-free constructs for *ARO8* and *ARO9* gene disruption were assembled using overlap extension PCR (OE-PCR) with

W29 *Y. lipolytica* genomic DNA as the template[1]. For *ARO8* gene, primers were 3204, 3205, 3206, and 3207 (Supplementary Table 16). For *ARO9* gene primers were 3208, 3209, 3210, and 3211 (Supplementary Table 16). Each construct represented a fusion of 500 bp of upstream and 500 bp of downstream homology regions flanking the corresponding gene. *Y. lipolytica* W29ΔuraΔku70 strain was co-transformed with each combination of marker-free construct (donor) and Cas9-helper (Cas9 and guide) according to procedures described in Section 2.2 of Supplementary Manual. Six large colonies from each transformation experiment were analysed by colony-PCR using primers pars 3212 and 3213 (for *ARO8* disruption) or 3214 and 3215 (for *ARO9* disruption). The fraction of clones with gene deletion was estimated for each Cas9-helper (Supplementary Figure 35), including pCasNA-ARO8a (4/6), pCasNA-ARO8c (6/6), pCasNA-ARO8i (5/6) and pCasNA-ARO9a (6/6), pCasNA-ARO9i (4/6), pCasNA-ARO9k (3/6), respectively, with overall efficiency of marker-free gene disruption 77,8% (28/36).

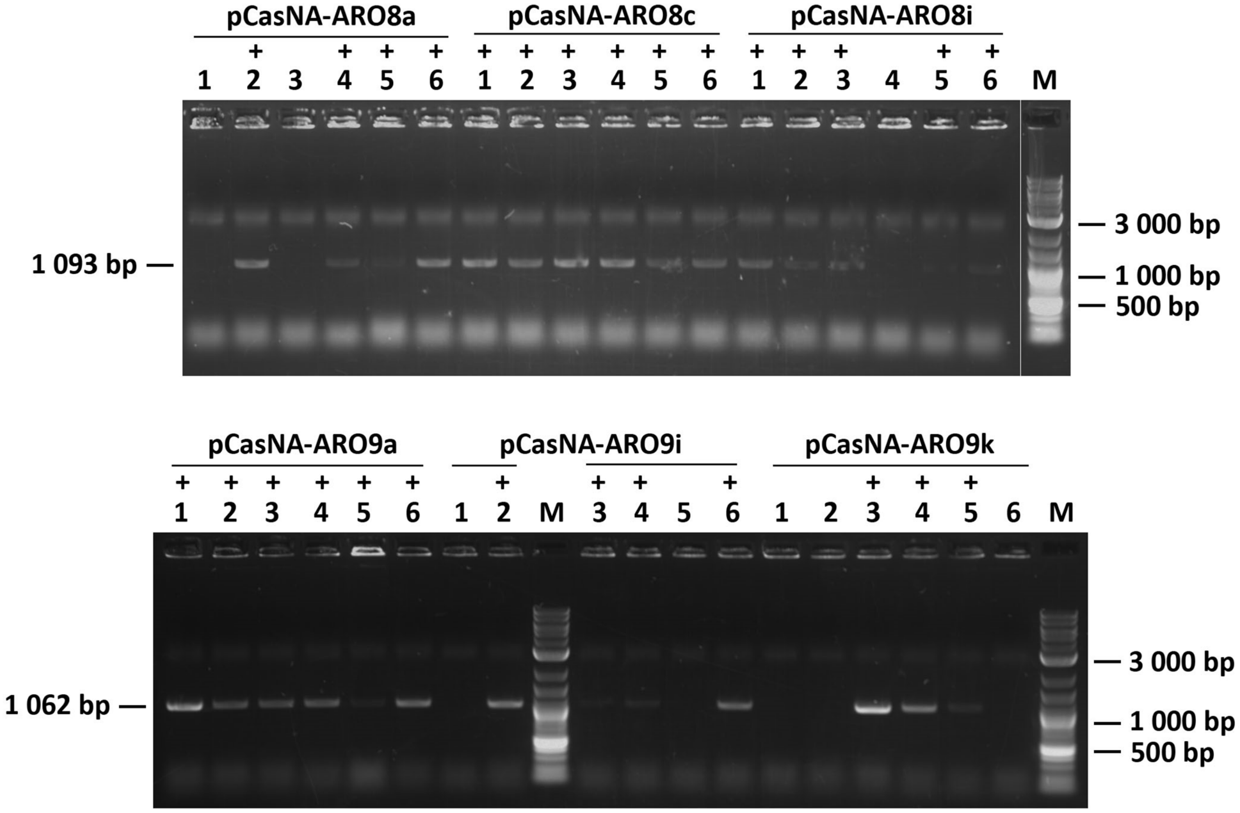

Supplementary Figure 35 The colony-PCR of *Y. lipolytica* clones isolated after marker-free disruption of *ARO8* and *ARO9* genes. PCR fragments corresponding to deletions are shown on the left side. The major bands of the molecular weight marker NEB #B7025 (M) are shown on the right side. The correct transformants bearing required deletions are designated by pluses above the clone numbers.

6.8 Assessing marker-free integration of overexpressing constructs in *Y. lipolytica*

For assessing marker free integration efficiency hrGFP was used as an example. Three marker-free constructs, pC2S1.1-hrGFP, pD12S1.1-hrGFP, and pE8S1.1-hrGFP, overexpressing *hrGFP* gene under control of *TEF1* promoter, were integrated into the genome of *Y. lipolytica* W29ΔuraΔku70 strain as described in Section 2.2 of Supplementary Manual. Among the selected clones, transformants were identified based on green fluorescence (Section 2.6 of Supplementary Manual). Correct integration among transformants with GFP-positive phenotype was confirmed using several clones with each construct (Supplementary Figure 36). As a result, the efficiency of

marker-free vector integrations into IntC2, IntD12, and IntE8 loci was estimated as 44.4% (8/18), 88.9% (16/18), and 57.1% (8/14), respectively, with the overall value equal to 64% (32/50).

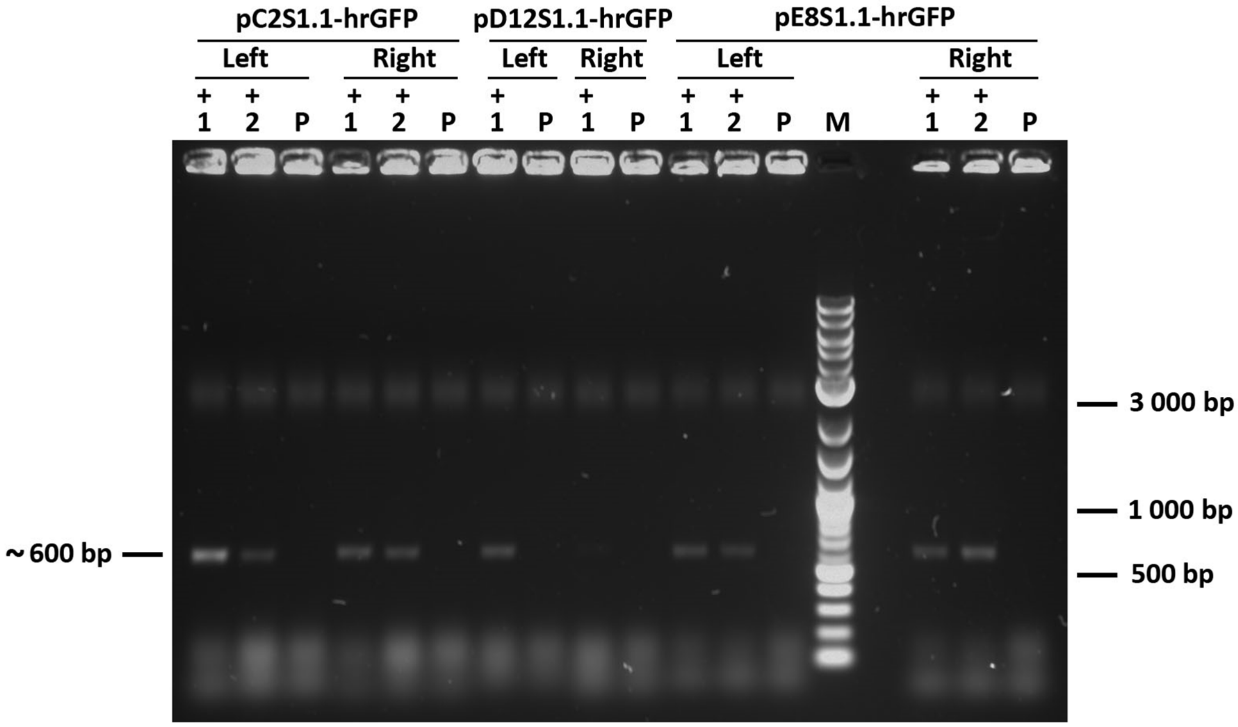

Supplementary Figure 36 The colony-PCR of *Y. lipolytica* clones isolated after marker-free integration of pC2S1.1-hrGFP, pD12S1.1-hrGFP, and pE8S1.1-hrGFP constructs. Standard primers were used specific to both integration flanks (Supplementary Tables 5 and 10). The PCR fragments corresponding to integration are as follows: IntC2 locus – 594 bp (left flank) and 601bp (right flank), IntD12 locus – 595bp (left flank) and 607bp (right flank), IntE8 locus – 589bp (left flank) and 597bp (right flank). Parent strain (P) was used as the negative control. The major bands of the molecular weight marker NEB #B7025 (M) are shown on the right side. The transformants with confirmed integration are designated with pluses above the clone numbers.

7. METABOLIC ENGINEERING

7.1 Engineering strains of *Y. lipolytica* to produce HGA

DNA assemblies of plasmid constructs required for metabolic engineering experiments are summarized in Supplementary Table 17. A pedigree of strains constructed during the metabolic engineering experiment are provided in the flowchart in Supplementary Figure 37.

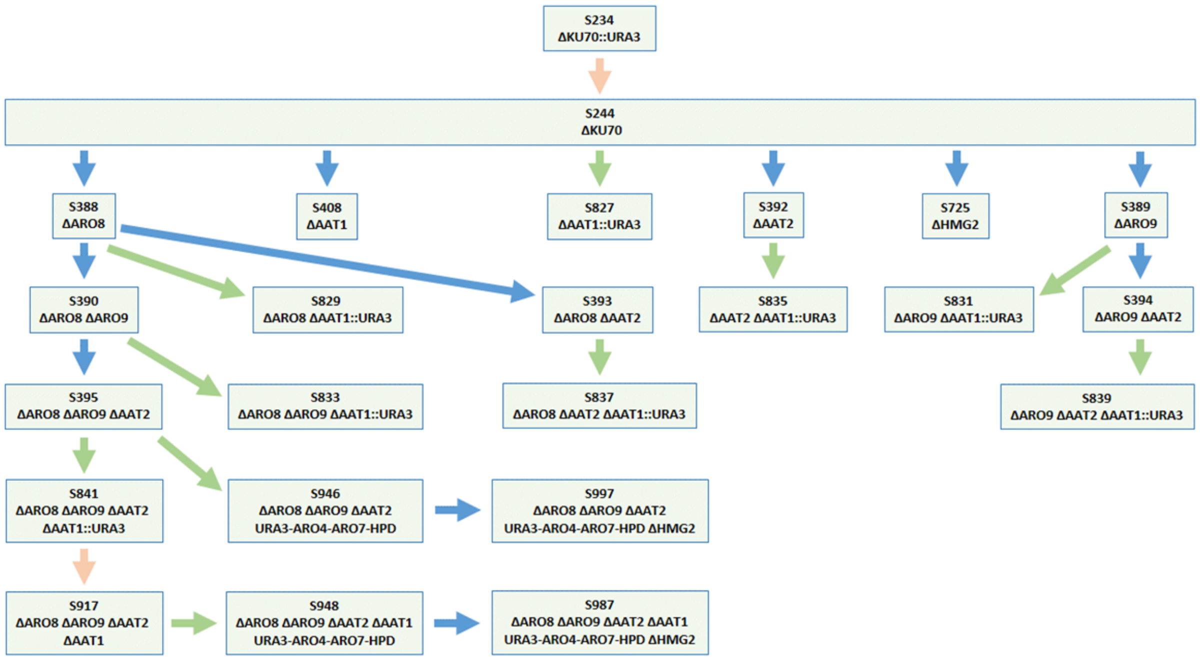

Supplementary Figure 37 Pedigree flowchart of the metabolic engineering of HGA producing *Y. lipolytica* strains by combining marker-free and marker-based integration approaches. Blue arrows, marker-free integration steps. Green arrows, marker-based integration steps. Pink arrows, marker recovery steps. All strains contained disruption of *KU70* gene.

Marker-free disruption of *ARO8* and *ARO9* genes was performed as described in Section 6.7. For *AAT2* gene deletion, the helper plasmid pCasNA-AAT2 was co-transformed with a marker-free disruption construct assembled by OE-PCR using primer set 3296, 3297, 3298, and 3299. Disruption of *AAT2* was verified by colony PCR using primers 3300 and 3301.

AAT1 gene was disrupted using both marker-free and marker-based approaches. For the marker-free approach pCasNA-AAT1 helper was co-transformed with a marker-free disruption construct assembled by OE-PCR using primers set 3308, 3309, 3310, and 3311. The deletion was verified by colony PCR using primers 3312 and 3313. Since initial attempts to disrupt *AAT1* gene in S395 (*Δaro8Δaro9Δaat2*) using the marker-free approach were not successful, we directly compared integration efficiency between S395 and the parental strain S234. Both S395 and S234 were transformed in parallel with the same marker-free construct and pCasNA-AAT1 helper. 8 big colonies were tested from each experiment by colony PCR. None of the tested transformants of strain S395 showed *AAT1* deletions (0/8), while all derivatives of S234 contained the knockout (8/8).

A similar comparison was done using a marker-based approach. For marker-based *AAT1* gene disruption the construct pDelUK-AAT1 was integrated using procedure described in Section 2.3 of Supplementary Manual. Correct disruptions were verified by colony PCR using primers 3373 and Barcode1-R (Supplementary Tables 10 and 16). Using this approach, we have isolated *AAT1* gene deletions in S395 with high efficiency (6/12). This suggested the convenience of using a selectable marker for the introduction of difficult modifications. Moreover, the appearance of transformants (Supplementary Figure 38) clearly demonstrated the detrimental phenotype of *Δaro8Δaro9Δaat2Δaat1::URA3* strains. Further comparative analysis using marker-based *AAT1* disruption in strains with different combinations of inactivated aminotransferases indicated that such a detrimental phenotype appeared in all experiments where the deletions of *AAT1* and *AAT2* genes were combined (Supplementary Figure 39).

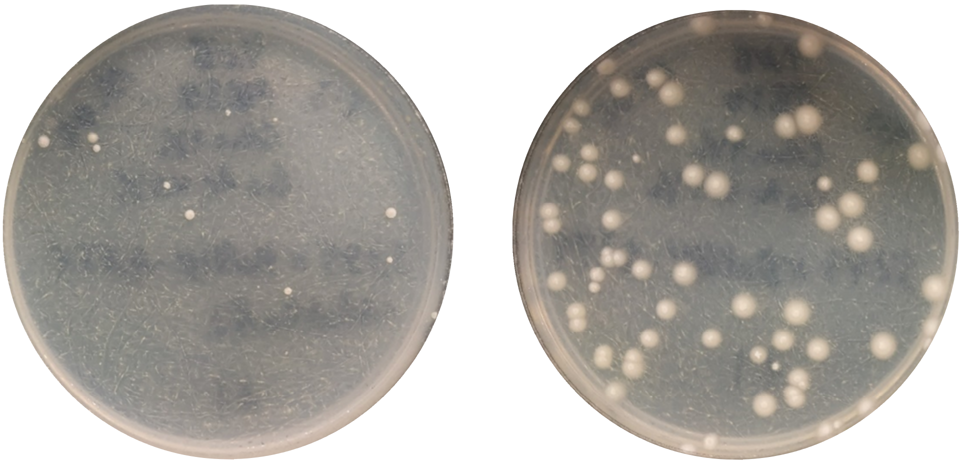

Supplementary Figure 38 Detrimental phenotype of *AAT1* gene disruption in strain S395. Both S395 (left) and parental strain W29ΔuraΔku70 (right) were transformed with 500 ng of pDelUK-AAT1 construct under the similar conditions. Transformants were selected on synthetic complete medium (YNB with 1% glucose, 0.4% casamino acids, and 50mM phosphate buffer, pH6.8). Pictures were taken on 9th day of incubation at 30 ^{o}C.

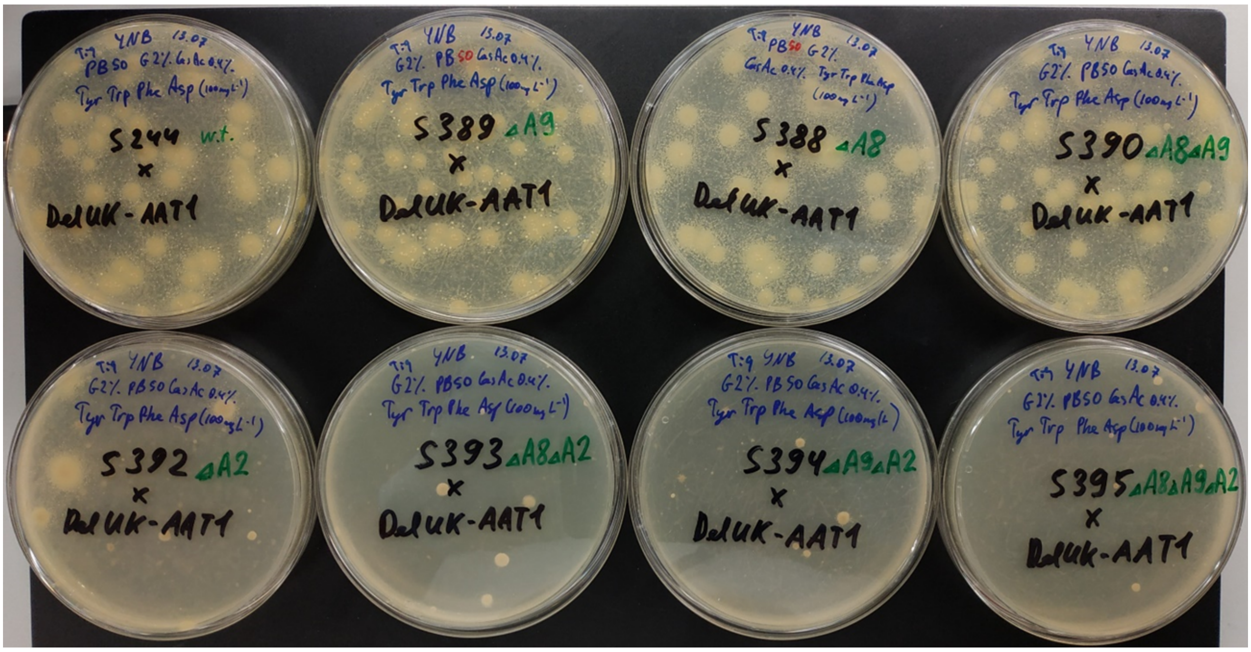

Supplementary Figure 39 Detrimental phenotype of combined deletions of *AAT1* and *AAT2* genes. Seven strains with all possible combinations of three aminotransferases gene deletions (*i.e. ARO8, ARO9* and *AAT2*) and parental strain S244 (W29ΔuraΔku70) were transformed with 500 ng of linearized pDelUK-AAT1 under the similar conditions. Transformants were selected on synthetic complete medium (YNB with 2% glucose, 0.4% casamino acids, and 50mM phosphate buffer, pH6.8) with further addition of 100 mg/L of each L-tyrosine, L-tryptophan, L-phenylalanine, and L-aspartate. Plates were imaged after 11 days of incubation at 30 ^{o}C. Detrimental phenotypes were observed in all experiments where recipients with *AAT2* gene disruption were used (bottom row of plates). To check genotypes, please see the pedigree flowchart (Supplementary Figure 37).

Then, the marker recovery protocol was applied to remove *URA3* gene from S841 resulting in strain S917 (Section 2.5 of Supplementary Manual). Attempts to transform both lineages (S395 and S917) with a marker-free construct overexpressing three genes (pE8S2.3-HPD-ARO4-ARO7)

using pCasNA-IntE8 helper did not lead to observed integration, assessed via colony PCR with primers 3143 and 3171 on 15 transformants from each experiment. Similarly, no integration with the same constructs was observed when the parental strain S244 was used (0/15). To verify which gene among those overexpressed lead to the toxic effects halting transformant selection, three transcriptional units were transformed in S244 separately using marker-free constructs (pE8A1.1-HPD1, pE8A1.2-ARO4 and pE8A1.3-ARO7) targeted to the same IntE8 locus using pCasNA-IntE8 helper. Colony PCR revealed integration of two genes, *HPD1* (5/15) and *ARO7*^{G141S} (7/15), while no correct integrations were observed for *ARO4*^{K229L} (0/15).

To further assess the toxic effect of *ARO4*^{K229L} allele, the random integration vector pZUS1.2-ARO4 was assembled and transformed in the recipient stain W29Δura (Supplementary Table 9), which retained functional NHEJ (Section 2.5 of Supplementary Manual). Random integration approaches frequently lead to integration of the vector parts instead of the whole sequence. Therefore, the obtained transformants could be a mixture of clones with either integration of the full-size vector overexpressing *ARO4*^{K229L} or a fragment with the *URA3* marker only. Accordingly, after 11 days of incubation, two clearly distinguishable colony types of different size were observed (Supplementary Figure 40). Colony PCR with primers 3064 and 3066 showed that only the small colonies contained *ARO4*^{K229L} gene, while larger colonies did not, therefore suggesting that overexpression of this gene reduced colony viability.

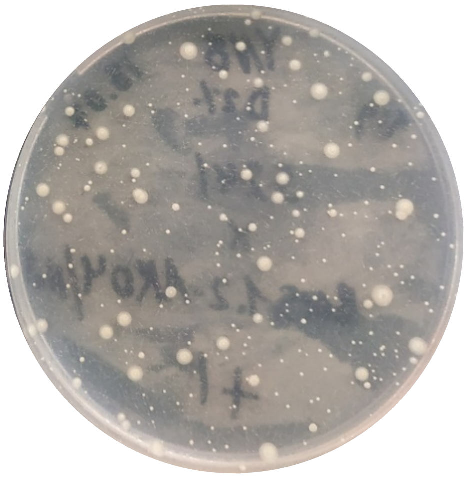

Supplementary Figure 40 Detrimental phenotype of *ARO4*^{K229L} overexpression. Plate contains transformants of W29Δura strain with random integrating construct pZUS1.2-ARO4. Picture was taken after 11 days of incubation at 30 ^{o}C on YNB medium with 2% glucose. Only small colonies contained *ARO4*^{K229L} allele.

To assemble both detrimental modifications (namely, double deletion *Δaat2Δaat1* and *ARO4*^{K229L} overexpression) in one strain we decided to combine both selection approaches, *e.g.* to stimulate integration of marker-based construct by inducing double-strand breaks in the target region via Cas9. Indeed, it has been shown that CRISPR/Cas9 assisted integration of marker-based construct yields a significantly higher integration frequency than with the same vector without Cas9 assistance[2]. Both strains, S395 with triple (*Δaro8Δaro9Δaat2*) and S917 with quadruple (*Δaro8Δaro9Δaat2Δaat1*) aminotransferase gene deletions were co-transformed by pE8US-HPD1-ARO4-ARO7 with pCasNA-IntE8 helper using a two-steps selection protocol (Section 2.4 of Supplementary Manual). Transformants were selected on YNB medium with 2% glucose, casamino acids (5 g/L) and 50mM phosphate buffer (pH6.8). After 10 days of incubation selected clones were analysed by colony PCR using standard primer sets for both flanks of IntE8 locus (Supplementary Tables 10 and 16). Correct clones were restreaked to single colony on the same medium and

presence of all three overexpressed genes, *i.e. HPD1*, *ARO4*^{K229L}, and *ARO7*^{G141S}, was confirmed by colony PCR using primer pairs 3064/3194, 3064/3066 and 3064/3390 respectively. As a result, strains S946 and S948 were obtained as derivatives of S395 and S917 (Supplementary Figure 37).

In the final step of engineering the HGA-producing strain, the gene *HMG2* was disrupted along with truncation of the telomeric region of chromosome D, as discussed below (Section 7.2). This truncation was induced by a pCasNA-HMG2 helper transformed without application of a donor molecule (Section 2.4 of Supplementary Manual). Positive transformants of strain lineages with three or four aminotransferase deletions, were recognized by a brown halo on YPD medium after 4 days of incubation resulting in derivatives designated as S997 and S987, respectively (Supplementary Figure 37).

To check HGA production, YNB media with 9% glucose was inoculated with each strain to an initial OD_{600} of 0.1 in 2.5mL of culture using 24-deepwell microplates (Enzyscreen, CR1424). Three strains were analysed, including both final strains (S997 and S987) and the wild type parent (S234) as the negative control. Cultivation was performed at high aeration rate (300 rpm) at 30° C. After 14 days biomass was separated and HGA concentration was measured in the supernatant by UPLC/MS. The accumulation of HGA by the two engineered strains is summarized in Supplementary Table 11.

Supplementary Table 11 HGA production by engineered *Y. lipolytica* strains.

Strain number	Strain genotype	HGA production (mg/L)	Standard deviation*
S234	*Δku70::URA3*	**0.0**	0.0
S987	*Δku70Δaro8Δaro9Δaat2Δaat1/↑HPD1↑ARO4↑ARO7/Δhmg2*	**339.1**	11.6
S997	*Δku70Δaro8Δaro9Δaat2/↑HPD1↑ARO4↑ARO7/Δhmg2*	**373.8**	4.1

* – standard deviation was estimated based on two biological replicates

For visual screening of pyomelanin production in liquid media similar cultivation conditions were applied, with a different carbon source and cultivation time. In that case 2% of sodium citrate (pH7.0) was used instead of glucose, with cultivation terminated after 8 days.

7.2 Discovery of *HMG2* gene and putative HGA degradation gene cluster in *Y. lipolytica*

Alignments of the amino acid sequence of homogentisate 1,2-dioxygenase of *Aspergillus fumigatus* (product of *hmgA*, GenBank XM_744461.1) revealed stretches of high similarity in a single region of chromosome D in *Y. lipolytica*. However, neither published genomic sequences of W29 strain (GenBank CP028451.1 and CP017556.1) revealed an intact open reading frame (ORF) in this region. Consequently, no locus tag was assigned in previous studies for this region during automated genome annotation[3]. Resequencing of this region in W29 strain revealed discrepancies with published sequences and allowed us to identify a new ORF that was designated as *HMG2* (GenBank accession number MZ387986). Analysis of the sequences around *HMG2* revealed two genes, which could be involved in the pathway of HGA degradation (Supplementary Figure 41). Upstream of *HMG2* lies an ORF (ORF2 on Supplementary Figure 41) encoding a protein with high level of similarity to a fumarylacetoacetate hydrolase from *A. fumigatus, fahA*. A third ORF next to these two (ORF3 on Supplementary Figure 41) encodes putative glutathione S-transferase, the enzyme family to which maleylacetoacetate isomerase encoded by *maiA* gene of *A. fumigatus* belongs. Notably, these three genes of *Y. lipolytica* are placed in the same order and orientation as the HGA-degradation cluster of *Aspergillus sp*[4].

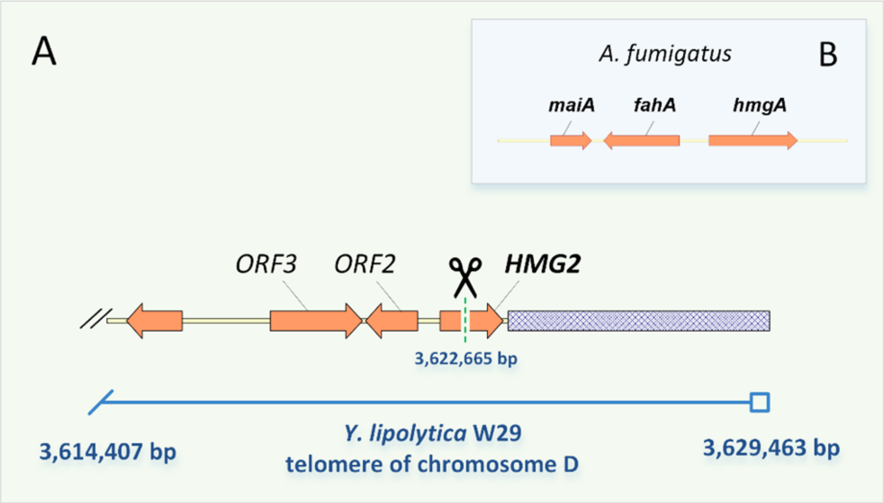

Supplementary Figure 41 Putative HGA degradation cluster of *Y. lipolytica*. **A**, structure of the telomeric region of chromosome D of *Y. lipolytica* W29. The gene cluster includes *HMG2* (homogentisate 1,2-dioxygenase), *ORF2* is YALI1_D36215g (putative fumarylacetoacetate hydrolase), and *ORF3* YALI1_D36182g (putative maleylacetoacetate isomerase). Hatched box is a terminal non-unique sequence repeated in the W29 genome. Nucleotide numbering is shown according to the published W29 chromosome D sequence (GenBank CP017556.1). The position of Cas9 cut site is shown that leads to chromosome truncation as described in the text. **B**, structure of similar HGA degradation cluster in *A. fumigatus* (GenBank NC 007195.1).

To test the effect of *HMG2* disruption, we assembled pCasNA-HMG2 helper cutting within the coding sequence of this gene (Supplementary Table 17). Since *HMG2* is the last unique sequence on the chromosome D, such a double-strand break will not affect the chromosome integrity if left unrepaired. Surprisingly, transformation of wild type background strain S244 with pCasNA-HMG2 resulted in numerous colonies secreting a brown pigment on YPD medium (Supplementary Figure 42). It is well known that pyomelanin is the product of self-oxidation and polymerization of HGA formed from aromatic amino acids[5]. At the same time, homogentisate 1,2-dioxygenase depletes HGA and, therefore its inactivation would facilitate pyomelanin formation. Therefore, this disruption enabled phenotype-based characterization of *HMG2* via visualisation of pyomelanin.

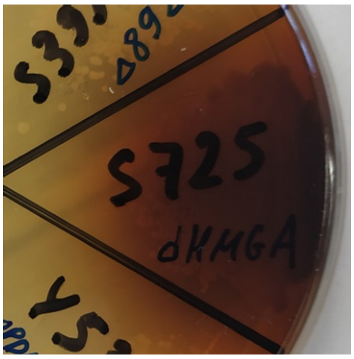

Supplementary Figure 42 Bioconversion of aromatic amino acids from rich medium into pyomelanin by *Y. lipolytica* strain with disrupted *HMG2* gene. The strain S725 is S244 derivative with truncation of the telomere region of chromosome D induced by Cas9-helper cutting inside the *HMG2* gene.

We can speculate that the truncation of this telomeric region could occur spontaneously in nature. Any double-strand break arising in or upstream of *HMG2* would lead to the characteristic 'brown' phenotype. Due to its chromosomal location, the cell cycle can proceed without reparation of these double-strand breaks, which otherwise requires searching the broken DNA ends, bringing them together, and fixing the gap by one of the available reparation mechanisms. This would maintain viability of spontaneous *HMG2* mutants, increasing the frequency of their appearance. Indeed, researchers who work with *Y. lipolytica* often observe mutants accumulating brown pigment on rich media[6, 7]. It is noteworthy to mention that the HGA degradation cluster is absent in sequenced genomes of *Y. lipolytica* strains CLIB122 and WSH-Z06, while present in H222 and IBT446. Interestingly, spontaneous pyomelanin accumulation by *Y. lipolytica* induces deep brown coloration of some ripened cheeses (*e.g.* Gorgonzola and Camembert) which leads to significant losses in manufacture[8, 9]. Therefore, a potential application of this finding might be the creation of *Y. lipolytica* strains with a more stable chromosomal location of the HGA degradation gene cluster.

8. PROMOTER LIBRARY SCREENING IN *Y. LIPOLYTICA*

8.1 Natural promoter libraries

Ideally, toolkits intended for the assembly of multiple transcription units should avoiding using repetitive sequences. Application of the same promoter in different positions leads to formation of direct repeats. Recombination between such promoters can induce of the removal of coding sequences locating between them and lead to instability of integrated constructs. To make systems more robust; which is essential for industrial applications; different promoters should be preferentially be made available. This is especially true for strong constitutive promoters, which are often limited, while also used very frequently, such as promoters of *Y. lipolytica* gene *TEF1*.

As a potential source of promoters with strong constitutive expression we selected genes encoding proteins of ribosomes. Studies on *S. cerevisiae* have shown that 50% of the transcripts produced by RNA polymerase II are devoted to the pool of ribosomal proteins[10], while their promoters are among the strongest during exponential growth phase[11]. Therefore, as the source of promoters for the libraries, we selected *Y. lipolytica* genes encoding proteins of small (40S) and large (60S) ribosomal subunits. Commercial synthesis of seven (namely, promoters *RPL5, RPL16, RPL28, RPS5, RPS7, RPS14,* and *RPS30*) was not possible due to complexity of secondary structure and widely distributed homopolymers. As a result, promoters of 26 genes encoding proteins of the small and 38 genes of the large subunit were chosen to test. Besides, we have selected 29 promoters of different origins including one from human cytomegalovirus (*hCMV*) and 28 from *Y. lipolytica*. Among those were genes encoding key metabolic reactions and different house-keeping genes supposed to be highly active in various media and growth conditions. Altogether, three libraries comprised 93 natural promoters that were synthesized and assembled on an empty pProUA-mScarlet vector (Supplementary GenBank files) (Section 1.8 of Supplementary Manual). To make promoters compatible with other modules of the toolkit, we introduced a limited number of point mutations (no more than 4 per promoter) in order to eliminate forbidden recognition sequences (Supplementary Table 3 of Supplementary Manual).

All obtained pProUA-series plasmids along with *TEF1* promoter (pProUA-TEF1) as a positive control were linearized by MssI and co-transformed with pCasNA-IntC2 helper into the strain S244 (W29ΔuraΔku70) using the procedure described in Section 2.6 of Supplementary Manual. For each construction two independent transformants were isolated and analysed for GFP activity. The parental strain S234 (W29Δku70ura+) was used as a negative control. The selected strains were first cultivated overnight (16 h) in 2 mL YPD. The biomass was precipitated at 4 ^{o}C, washed with saline solution and resuspended to get OD_{600} 10.0. At this step, the activities of the different promoters could be visually compared using blue LED transilluminator (*e.g.* DR46B, Clare Chemical Research) with orange filter (Supplementary Figure 43).

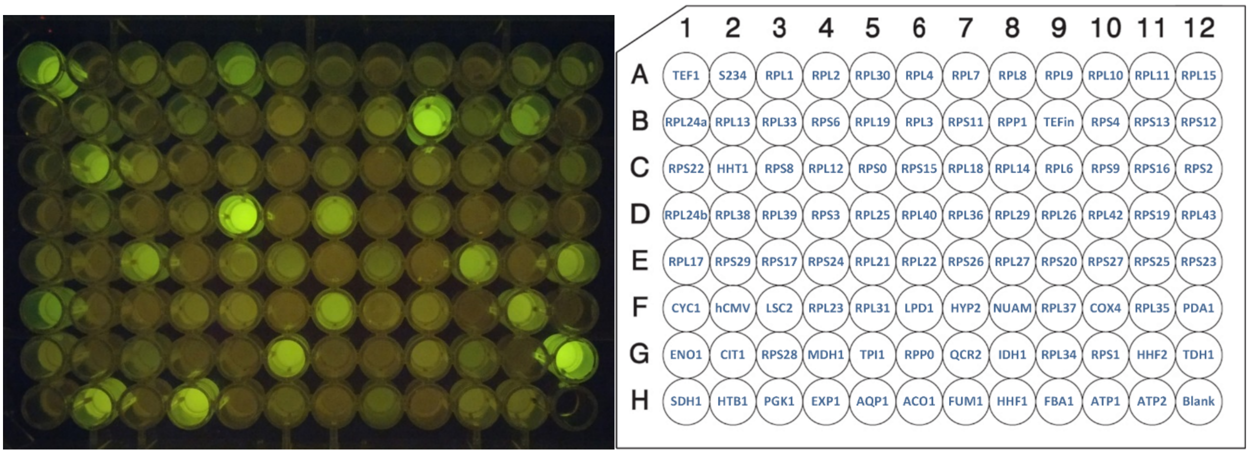

Supplementary Figure 43 Visual screening of promoter libraries integrated in *Y. lipolytica* genome using CRISPR/Cas9. Left, three libraries of native promoters were combined in 96-well plate. Single transformant represents each of 96 tested promoters. Right, plate map shown with gene names that were used as the source of the promoters.

Such suspensions were diluted with the same volume of 16% DMSO solution to an OD_{600} 5.0. Then, samples were aliquoted by 10 µL and stored at -80 ^{o}C. For fluorescence measurement these aliquots were defrosted and used to inoculate 2 mL of YPD and YNB media with 2% glucose to a starting OD_{600} of 0.025. Cells were grown at 30 ^{o}C with shaking (250 rpm) until exponential growth phase, which corresponded to 16 and 24 hours of incubation for YPD and YNBD, respectively. Obtained cultures were washed and resuspended in the same volume of saline solution. 200 µl of each suspension were transferred into black-walled 96-well plates with flat transparent bottom (665096 Greiner Bio-One) and green fluorescence (excitation, 495 nm; emission, 535 nm) along with OD_{600} were measured using luminometer CLARIOstar Plus (BMG Labtech). The gain of the photomultiplier was automatically controlled by enhanced dynamic range function. Measured values were normalized for 1 s accumulation time. Acquired data were analysed using MARS software (BMG Labtech). All fluorescence and OD_{600} data were automatically blanked using the reference well with the saline solution. Relative fluorescence (*RF*) for each well was calculated by dividing fluorescence on OD_{600}. Promoter strength (*Px*) was calculated using the following equation:

$$(1)\ P_X(\%) = \frac{RF_{Px} - \bar{X}(RF_{parent})}{\bar{X}(RF_{TEF1} - \bar{X}(RF_{parent}))} \times 100$$

, where RF_{Px}, RF_{TEF1}, and RF_{Parent} are relative fluorescences of strains with promoters *Px*, *TEF1* and the parent strain S234, respectively.

As a result, the autofluorescence of the parent strain S234 (without GFP) was taken as the baseline (0%), while the strength of all promoters was normalized on the *TEF1* activity corresponding to 100%. Overall, during three independent experiments 93 native promoters were functionally characterized (Supplementary Tables 12, 13, and 14).

Supplementary Table 12 Measured strength of 26 promoters of *Y. lipolytica* genes encoding 40S ribosomal proteins

Promoter	Source gene	Locus tag	Supposed function of gene product	Donor plasmid	Promoter strength (%)*			
					YPD mean	YPD SD	YNBD mean	YNBD SD
**Parent S234**					0,0	0,11	0,0	0,54
**RPS15**	*Y. lipolytica RPS15*	YALI1_F08635g	Ribosomal 40S subunit protein S15	pProUA-RPS15	0,7	0,51	1,32	0,02
**RPS26**	*Y. lipolytica RPS26*	YALI1_E23610g	Ribosomal 40S subunit protein S26	pProUA-RPS26	1,3	0,35	1,79	0,44
**RPS24**	*Y. lipolytica RPS24*	YALI1_F32659g	Ribosomal 40S subunit protein S24B	PproUA-RPS24	1,2	0,44	1,96	0,65
**RPS20**	*Y. lipolytica RPS20*	YALI1_E24442g	Ribosomal 40S subunit protein S20	pProUA-RPS20	2,1	0,37	2,38	1,02
**RPS25**	*Y. lipolytica RPS25*	YALI1_C05133g	Ribosomal 40S subunit protein S25	pProUA-RPS25	1,7	0,64	3,59	0,98
**RPS29**	*Y. lipolytica RPS29*	YALI1_B11788g	Ribosomal 40S subunit protein S29	pProUA-RPS29	2,6	1,31	3,81	0,95
**RPS16**	*Y. lipolytica RPS16*	YALI1_B17008g	Ribosomal 40S subunit protein S16	pProUA-RPS16	4,8	2,2	5,1	0,2
**RPS12**	*Y. lipolytica RPS12*	YALI1_F09230g	Ribosomal 40S subunit protein S12	pProUA-RPS12	2,1	0,12	6,28	0,98
**RPS22**	*Y. lipolytica RPS22*	YALI1_D07348g	Ribosomal 40S subunit protein S24A	PproUA-RPS22	7,02	2,3	8,04	0,71
**RPS11**	*Y. lipolytica RPS11*	YALI1_F27175g	Ribosomal 40S subunit protein S11	pProUA-RPS11	9,7	1,23	12,47	5,54
**RPS3**	*Y. lipolytica RPS3*	YALI1_E28033g	Ribosomal 40S subunit protein S3	pProUA-RPS3	16,4	0,1	13,14	3,08
**RPS8**	*Y. lipolytica RPS8*	YALI1_F32305g	Ribosomal 40S subunit protein S8	pProUA-RPS8	22,5	1,3	19,39	1,21
**RPS9**	*Y. lipolytica RPS9*	YALI1_F08284g	Ribosomal 40S subunit protein S9	pProUA-RPS9	26,6	1,72	19,4	2,61
**RPS0**	*Y. lipolytica RPS0*	YALI1_A18557g	Ribosomal 40S subunit protein S0	pProUA-RPS0	15,4	0,6	20,76	0,73
**RPS4**	*Y. lipolytica RPS4*	YALI1_D16029g	Ribosomal 40S subunit protein S4	pProUA-RPS4	28,5	0,3	21,16	4,33
**RPS2**	*Y. lipolytica RPS2*	YALI1_E17466g	Ribosomal 40S subunit protein S2	pProUA-RPS2	30,4	0,2	22,05	3,63
**RPS19**	*Y. lipolytica RPS19*	YALI1_B26808g	Ribosomal 40S subunit protein S19	pProUA-RPS19	27,2	0,31	22,96	1,2
**RPS28**	*Y. lipolytica RPS28*	YALI1_C06597g	Ribosomal 40S subunit protein S28	pProUA-RPS28	28,4	0,14	23,01	4,26
**RPP1**	*Y. lipolytica RPP1*	YALI1_B16968g	60S acidic ribosomal protein P1	pProUA-RPP1	25,9	0,5	23,3	4,89
**RPS1**	*Y. lipolytica RPS1*	YALI1_F08431g	Ribosomal 40S subunit protein S1	pProUA-RPS1	25,8	2,7	29,68	2,18
**RPS6**	*Y. lipolytica RPS6*	YALI1_F25001g	Ribosomal 40S subunit protein S6	pProUA-RPS6	32,1	0,6	30,09	0,24
**RPS13**	*Y. lipolytica RPS13*	YALI1_F14712g	Ribosomal 40S subunit protein S13	pProUA-RPS13	47,4	4,6	33,8	3,9
**RPS17**	*Y. lipolytica RPS17*	YALI1_F19249g	Ribosomal 40S subunit protein S17	pProUA-RPS17	42,9	5,66	34,24	1,53
**RPS23**	*Y. lipolytica RPS23*	YALI1_A21775g	Ribosomal 40S subunit protein S23	pProUA-RPS23	45,4	6,56	35,06	1,58
**RPS27**	*Y. lipolytica RPS27*	YALI1_E41367g	Ribosomal 40S subunit protein S27	pProUA-RPS27	45,1	5	39,07	2,31
**RPP0**	*Y. lipolytica RPP0*	YALI1_B18707g	Ribosomal protein P0	pProUA-RPP0	40,6	5,6	41,85	4,68
**TEF1**	*Y. lipolytica TEF1*	YALI1_C12642g	Translational elongation factor EF-1 alpha	pProUA-TEF1	100	2,33	100	2,89

* - Standard deviation was calculated based on two replicates using independent transformants. Promoter strengths were sorted from weak to strong based on the results obtained in the minimal YNBD medium.

Supplementary Table 13 Measured strength of 38 promoters of *Y. lipolytica* genes encoding 60S ribosomal proteins

Promoter	Source gene	Locus tag	Supposed function of gene product	Donor plasmid	Promoter strength (%)			
					YPD mean	YPD SD	YNBD mean	YNBD SD
**Parent S234**					0,0	0,4	0,0	0,33
**RPL42**	*Y. lipolytica RPL42*	YALI1_B05646g	Ribosomal 60S subunit protein L42	pProUA-RPL42	1,3	1,3	1,95	0,16
**RPL10**	*Y. lipolytica RPL10*	YALI1_D16249g	Ribosomal 60S subunit protein L10	pProUA-RPL10	3,5	0	2,05	0,27
**RPL8**	*Y. lipolytica RPL8*	YALI1_F32043g	Ribosomal 60S subunit protein L8	pProUA-RPL8	2,4	0,9	3,37	0,45
**RPL39**	*Y. lipolytica RPL39*	YALI1_D07338g	Ribosomal 60S subunit protein L39	pProUA-RPL39	1,1	0,3	3,85	0,21
**RPL43**	*Y. lipolytica RPL43*	YALI1_E41061g	Ribosomal 60S subunit protein L43	pProUA-RPL43	3,8	1,7	5,29	0,75
**RPL40**	*Y. lipolytica RPL40*	YALI1_F12190g	Ribosomal 60S subunit protein L40	pProUA-RPL40	7,7	3,4	5,52	1,36
**RPL24A**	*Y. lipolytica RPL24A*	YALI1_E27880g	Ribosomal 60S subunit protein L24A	pProUA-RPL24A	4	2,1	6,07	0,51
**RPL19**	*Y. lipolytica RPL19*	YALI1_E29930g	Ribosomal 60S subunit protein L19	pProUA-RPL19	5,2	0,5	6,17	0,47
**RPL14**	*Y. lipolytica RPL14*	YALI1_E00928g	Ribosomal 60S subunit protein L14	pProUA-RPL14	4,1	0,9	6,49	1,02
**RPL24B**	*Y. lipolytica RPL24B*	YALI1_B13137g	Ribosomal 60S subunit protein L24B	pProUA-RPL24B	4,4	0,4	6,83	2,19
**RPL33**	*Y. lipolytica RPL33*	YALI1_E37930g	Ribosomal 60S subunit protein L33	pProUA-RPL33	8,8	3,6	7,7	1,43
**RPL29**	*Y. lipolytica RPL29*	YALI1_F11855g	Ribosomal 60S subunit protein L29	pProUA-RPL29	12,5	0,2	12,94	1,47
**RPL9**	*Y. lipolytica RPL9*	YALI1_E34344g	Ribosomal 60S subunit protein L9	pProUA-RPL9	19	3,8	12,47	0,6
**RPL6**	*Y. lipolytica RPL6*	YALI1_E32842g	Ribosomal 60S subunit protein L6	pProUA-RPL6	21,3	1,8	15,85	2,84
**RPL11**	*Y. lipolytica RPL11*	YALI1_B19897g	Ribosomal 60S subunit protein L11	pProUA-RPL11	24	3,4	16,38	4,77
**RPL4**	*Y. lipolytica RPL4*	YALI1_C09094g	Ribosomal 60S subunit protein L4	pProUA-RPL4	27,3	0,2	17,08	0,33
**RPL17**	*Y. lipolytica RPL17*	YALI1_C22922g	Ribosomal 60S subunit protein L17	pProUA-RPL17	22,7	2,7	17,1	3,9
**RPL31**	*Y. lipolytica RPL31*	YALI1_E29150g	Ribosomal 60S subunit protein L31	pProUA-RPL31	21,4	3,6	17,11	7,36
**RPL13**	*Y. lipolytica RPL13*	YALI1_B16996g	Ribosomal 60S subunit protein L13	pProUA-RPL13	26,3	1,7	18,07	3,45
**RPL2**	*Y. lipolytica RPL2*	YALI1_F32090g	Ribosomal 60S subunit protein L2	pProUA-RPL2	21,4	4,1	19,64	2,67
**RPL1**	*Y. lipolytica RPL1*	YALI1_E37886g	Ribosomal 60S subunit protein L1	pProUA-RPL1	28,9	3,2	20,05	3,93
**RPL15**	*Y. lipolytica RPL15*	YALI1_D32153g	Ribosomal 60S subunit protein L15	pProUA-RPL15	29,5	2,4	20,37	2
**RPL22**	*Y. lipolytica RPL22*	YALI1_E38178g	Ribosomal 60S subunit protein L22	pProUA-RPL22	32,6	3,8	20,42	4,34
**RPL37**	*Y. lipolytica RPL37*	YALI1_F39051g	Ribosomal 60S subunit protein L37	pProUA-RPL37	20,9	1,6	21	2,26
**RPL34**	*Y. lipolytica RPL34*	YALI1_C06511g	Ribosomal 60S subunit protein L34	pProUA-RPL34	23,6	7,3	21,34	5,2
**RPL18**	*Y. lipolytica RPL18*	YALI1_B11934g	Ribosomal 60S subunit protein L18	pProUA-RPL18	30,8	1,5	21,59	1
**RPL23**	*Y. lipolytica RPL23*	YALI1_D12930g	Ribosomal 60S subunit protein L23	pProUA-RPL23	20,1	0,1	22,22	2,38
**RPL3**	*Y. lipolytica RPL3*	YALI1_C29810g	Ribosomal 60S subunit protein L3	pProUA-RPL3	21,8	1,6	22,46	2,1
**RPL27**	*Y. lipolytica RPL27*	YALI1_B07937g	Ribosomal 60S subunit protein L27	pProUA-RPL27	35,6	2,8	22,59	0,75
**RPL21**	*Y. lipolytica RPL21*	YALI1_F08260g	Ribosomal 60S subunit protein L21	pProUA-RPL21	34	0,1	25,77	1,46
**RPL7**	*Y. lipolytica RPL7*	YALI1_E16649g	Ribosomal 60S subunit protein L7	pProUA-RPL7	18,5	2,6	27,23	0,1
**RPL38**	*Y. lipolytica RPL38*	YALI1_B14993g	Ribosomal 60S subunit protein L38	pProUA-RPL38	28,8	0,3	26,94	1,64
**RPL35**	*Y. lipolytica RPL35*	YALI1_A09798g	Ribosomal 60S subunit protein L35	pProUA-RPL35	37,7	1,4	28,38	1,87
**RPL12**	*Y. lipolytica RPL12*	YALI1_D17206g	Ribosomal 60S subunit protein L12	pProUA-RPL12	34,4	2,9	30	0,06
**RPL26**	*Y. lipolytica RPL26*	YALI1_E25317g	Ribosomal 60S subunit protein L26	pProUA-RPL26	29,7	2,1	31,23	4,39
**RPL36**	*Y. lipolytica RPL36*	YALI1_E35963g	Ribosomal 60S subunit protein L36	pProUA-RPL36	36,5	6,2	37,28	1,04
**RPL30**	*Y. lipolytica RPL30*	YALI1_E27871g	Ribosomal 60S subunit protein L30	pProUA-RPL30	56,3	6,1	48,14	2,49
**TEF1**	*Y. lipolytica TEF1*	YALI1_C12642g	Translational elongation factor EF-1 alpha	pProUA-TEF1	100	2,33	100	3,73
**RPL25**	*Y. lipolytica RPL25*	YALI1_F32792g	Ribosomal 60S subunit protein L25	pProUA-RPL25	130,9	9,17	125,66	3,38

Supplementary Table 14 Measured strength of 29 natural promoters of different sources

Promoter	Source gene	Locus tag	Supposed function of gene product	Donor plasmid	Promoter strength (%)			
					YPD mean	YPD SD	YNBD mean	YNBD SD
**Parent S234**					0,0	0,33	0,0	0,66
**FUM1**	*Y. lipolytica FUM1*	YALI0C06776g	Fumarate hydrotase mitochondrial	pProUA-FUM1	0,82	0,52	0,64	0,36
**PGK1**	*Y. lipolytica PGK1*	YALI1_D15424g	Phosphoglycerate kinase	pProUA-PGK1	0,62	0,27	0,75	0,5
**SDH1**	*Y. lipolytica SDH1*	YALI1_D14260g	Flavoprotein subunit of succinate dehydrogenase	pProUA-SDH1	0,34	0,31	0,87	0,65
**AQP**	*Y. lipolytica AQP*	YALI1_F00616g	Protein similar to aquaporin 9 (Small solute channel 1)	pProUA-AQP	0,8	0,7	0,91	0,34
**hCMV**	*Human Cytomegalovirus*		Human cytomegalovirus (CMV) immediate early promoter	pProUA-hCMV	2,62	0,5	1,72	0,84
**LPD1**	*Y. lipolytica LPD1*	YALI1_D26360g	Dihydrolipoyl dehydrogenase	pProUA-LPD1	4,08	0,62	2,26	1,38
**FBA1**	*Y. lipolytica FBA1*	YALI0E26004g	Fructose 1,6-bisphosphate aldolase	pProUA-FBA1	1,36	0,2	2,6	0,33
**LSC2**	*Y. lipolytica LSC2*	YALI1_D05892g	Beta subunit of mitochondrial succinyl-CoA ligase	pProUA-LSC2	4	0,8	3,95	2,23
**NUAM**	*Y. lipolytica NUAM*	YALI1_D07089g	Subunit NUAM of NADH:Ubiquinone Oxidoreductase (Complex I)	pProUA-NUAM	4,65	0,32	4,33	1,52
**TPI1**	*Y. lipolytica TPI1*	YALI1_F07847g	Triosephosphate isomerase	pProUA-TPI1	9,1	1,03	5,94	2,15
**COX4**	*Y. lipolytica COX4*	YALI1_E23635g	Subunit IV of cytochrome c oxidase	pProUA-COX4	7,4	0,3	6,16	1,79
**PDA1**	*Y. lipolytica PDA1*	YALI1_F27556g	E1α subunit of the pyruvate dehydrogenase	pProUA-PDA1	6,9	0,27	7,23	2,38
**IDH1**	*Y. lipolytica IDH1*	YALI1_E06015g	Subunit of mitochondrial NAD(+)-dependent isocitrate dehydrogenase	pProUA-IDH1	11,4	0,81	10,09	3,14
**MDH1**	*Y. lipolytica MDH1*	YALI1_D20676g	Mitochondrial malate dehydrogenase	pProUA-MDH1	8,98	1,1	10,67	1,09
**QCR2**	*Y. lipolytica QCR2*	YALI1_F12033g	Subunit 2 of ubiquinol cytochrome-c reductase (Complex III)	pProUA-QCR2	10,55	0,4	10,44	2,48
**HHF2**	*Y. lipolytica HHF2*	YALI1_F33331g	Histone H4	pProUA-HHF2	8,18	1,5	12,63	0,67
**ATP1**	*Y. lipolytica ATP1*	YALI1_F04455g	Alpha subunit of the F1 sector of mitochondrial ATP synthase	pProUA-ATP1	19,22	1,24	13,54	3,47
**ACO1**	*Y. lipolytica ACO1*	YALI1_D11984g	Gene encoding a mitochondrial aconitate hydratase	pProUA-ACO1	18,87	0,6	13,6	1,44
**CIT1**	*Y. lipolytica CIT1*	YALI1_E03300g	Citrate synthase mitochondrial	pProUA-CIT1	16,33	0,59	17,76	0,56
**CYC1**	*Y. lipolytica CYC1*	YALI1_D11769g	Cytochrome c	pProUA-CYC1	25,58	3,71	20,06	3,13
**ENO1**	*Y. lipolytica ENO2*	YALI1_F22436g	Enolase 1	pProUA-ENO1	21,24	1,41	20,11	0,13
**HYP2**	*Y. lipolytica HYP2*	YALI1_C09230g	Translation elongation factor eIF-5A	pProUA-HYP2	37,24	7,1	27,07	2,84
**ATP2**	*Y. lipolytica ATP2*	YALI1_B05364g	Beta subunit of the F1 sector of mitochondrial ATP synthase	pProUA-ATP2	22,3	4,4	27,43	2,82
**HHF1**	*Y. lipolytica HHF1*	YALI1_C15964g	Histone H4	PproUA-HHF1	31,78	0,2	31,49	0,53
**HTB1**	*Y. lipolytica HTB1*	YALI1_E31389g	Histone H2B	pProUA-HTB1	62,35	1,4	53,85	1,63
**HHT1**	*Y. lipolytica HHT1*	YALI1_F33345g	Histone H3	pProUA-HHT1	68,9	1,4	61,29	5,75
**TEF1**	*Y. lipolytica TEF1*	YALI1_C12642g	Translational elongation factor EF-1 alpha	pProUA-TEF1	100	3,7	100	3,33
**EXP1**	*Y. lipolytica EXP1*	YALI1_C16851g	Cargo receptor protein for Pma1p	pProUA-EXP1	84,76	1,47	103,55	7,02
**TEFIn**	*Y. lipolytica TEFIn*	YALI1_C12642g	Translational elongation factor EF-1 alpha;	pProUA-TEFIn	117,3	4,8	115	1,81
**TDH1**	*Y. lipolytica TDH1*	YALI1_C08262g	Glyceraldehyde-3-phosphate dehydrogenase	pProUA-TDH1	122,25	13,38	166,1	12,45

8.2 Hybrid promoter library

The number of natural promoters available for a single organism is limited and their number and properties can be significantly expanded by combining regulatory sequences. Such sequences can be of different origins, including genes derived from other species. Therefore, we decided to use new screening system (*i.e.* the Pro Module) to test another library consisting of hybrid promoters. Initially, we designed 105 promoters. However, only 43 of them were possible to synthesize due to wildly distributed homopolymer sequences. As a result, these 43 hybrid promoters were synthesized and assembled as pProUA-series using commercial service (Supplementary GenBank files). Each promoter was 800 bp in length and combined three to four sequences from seven different yeast species, including *Candida hispaniensis*, *Kluyveromyces lactis*, *Kluyveromyces marxianus*, *Komagataella phaffii*, *Ogataea polymorpha, S. cerevisiae,* and *Y. lipolytica* (Section 10). All sequences were selected from upstream regions of genes expected to be highly expressed due to their roles in highly demanded cellular functions, including glycolysis, TCA cycle, pentose phosphate pathway, substrate utilization, cytosolic membrane transport, electron transfer and translation machinery (Supplementary Table 15).

All pProUA-series plasmids along with a positive control *TEF1* promoter were linearized using MssI and co-transformed with pCasNA-IntC2 into S244 strain (W29ΔuraΔku70) (Section S2.6 of Supplementary Manual). Two independent transformants with each construct were analysed. As the negative control, parental strain S234 (W29Δku70ura+) was used. First, cultures were grown overnight in 2.5 mL of YPD. The biomass was washed with saline solution and diluted in 10% glycerol up to an OD_{600} 1.0. Such seeding material was aliquoted by 10 µL in 96-well flat-bottom plates and stored at -80 ^{o}C. To measure promoter activities at single-cell level, flow cytometry was used. For this purpose, each aliquot was mixed with 90 µL of either YPG or YNBG, both supplemented with 1.1% glycerol. Therefore, each well contained 100 µL of culture with initial OD_{600} 0.1 and glycerol concentration adjusted to 2%. Microplates were grown in plate reader Synergy HT (BioTek). To increase the aeration, a plastic lid was used and maximal shaking rate was applied. OD_{600} was measured every 20 min and monitored using Gen5 software (BioTek). Using trial experiments the mid-exponential growth phase was observed at 9th or 15th hours of the incubation in YPG or YNBG media, respectively. At that time point cultivations were stopped and microplates were transferred to flow cytometer Attune NxT (Thermo Fisher Scientific). The fluorescence data were collected from 10,000 cells for each sample and GFP fluorescence was measured using excitation with a 488 nm laser and a 510/10 nm emission filter. The data were analysed using FlowJo software (Ashland, OR). As a result, the median fluorescence intensity (*MFI*) was calculated for each sample based on fluorescence of individual cells. Promoter strength (*Px*) was estimated using the following equation:

$$(2)\ P_X(\%) = \frac{MFI_{Px} - \underline{X}(MFI_{parent})}{\underline{X}(MFI_{TEF1} - \underline{X}(MFI_{parent}))} \times 100$$

, where MFI_{Px}, MFI_{TEF1}, and MFI_{Parent} are median fluorescence intensity of strains with promoters *Px*, *TEF1* and the parent strain S234, respectively. Resulting strengths of 43 hybrid promoters are summarized in Supplementary Table 15.

Supplementary Table 15 Measured strength and composition of 43 hybrid promoters

Promoter	Structure of a hybrid promote				Relative Promoter Strength (%)			
	Source 1 (species gene)	Source 2 (species gene)	Source 3 (species gene)	Source 4 (species gene)	YPG mean	YPG SD	YNBG mean	YNBG SD
**Parent S234**					0,0	0,01	0,0	0,15
**P78**	*S. cerevisiae TEF1*	*K. phaffii TEF1*	*S. cerevisiae HXT7*		0,5	0,10	-0,1	0,05
**P98**	*Y. lipolytica PGK1*	*O. polymorpha CYC1*	*O. polymorpha PSH1*		0,7	0,19	0,1	0,05
**P24**	*Y. lipolytica GPDH*	*Y. lipolytica ENO2*	*K. marxianus TAL1*		0,4	0,14	0,2	0,30
**P1**	*S. cerevisiae PFK2*	*O. polymorpha PYK1*	*K. phaffii GND2*		0,0	0,17	0,2	0,50
**P36**	*S. cerevisiae ZWF1*	*K. phaffii PGK1*	*Y. lipolytica ADH1*		0,5	0,04	0,2	0,05
**P55**	*Y. lipolytica ENO2*	*O. polymorpha PYK1*	*S. cerevisiae PGK1*		0,8	0,13	0,3	0,10
**P11**	*S. cerevisiae ENO1*	*K. phaffii GPM1*	*Y. lipolytica GPDH*		0,2	0,08	0,4	0,00
**P43**	*Y. lipolytica PGK1*	*K. marxianus TAL1*	*K. lactis GPDH*		0,8	0,09	0,4	0,10
**P23**	*Y. lipolytica TEF1*	*Y. lipolytica ZWF1*	*K. marxianus CYC1*		0,5	0,02	0,4	0,20
**P95**	*S. cerevisiae TEF1*	*Y. lipolytica ZWF1*	*K. phaffii PYK1*		1,0	0,12	0,4	0,05
**P8**	*O. polymorpha TPI1*	*O. polymorpha PYK1*	*K. phaffii ADH1*		0,4	0,08	0,9	0,35
**P99**	*Y. lipolytica GPDH*	*O. polymorpha PFK2*	*K. phaffii AOX1*		0,9	0,03	1,2	0,30
**P94**	*Y. lipolytica GPDH*	*O. polymorpha PGI1*	*O. polymorpha TAL1*		1,2	0,01	1,4	0,00
**P3**	*K. phaffii MDH1*	*K. phaffii PFK1*	*K. phaffii GND2*		0,4	0,35	1,4	0,05
**P10**	*K. phaffii MDH1*	*O. polymorpha ENO2*	*O. polymorpha TAL1*		0,8	0,04	1,4	1,40
**P92**	*O. polymorpha ZWF1*	*Y. lipolytica PFK1*	*O. polymorpha TAL1*		1,2	0,00	1,5	0,05
**P32**	*S. cerevisiae ENO1*	*S. cerevisiae ZWF1*	*Y. lipolytica PFK1*		1,2	0,10	1,6	0,05
**P13**	*Y. lipolytica PGK1*	*K. marxianus MDH1*	*K. phaffii AOX1*		0,5	0,27	1,8	0,00
**P37**	*Y. lipolytica PGK1*	*Y. lipolytica FBA1*	*Y. lipolytica ENO2*		1,5	0,13	2,1	0,35
**P14**	*Y. lipolytica GPDH*	*O. polymorpha ENO2*	*K. phaffii AOX1*		1,0	0,03	2,3	0,74
**P5**	*O. polymorpha ACO1*	*K. phaffii PGK1*	*K. phaffii GND2*		0,5	0,25	2,5	0,05
**P50**	*Y. lipolytica MDH1*	*K. lactis PFK2*	*K. phaffii AOX*		2,3	0,03	2,5	0,15
**P4**	*K. phaffii TPI1*	*O. polymorpha ENO2*	*K. phaffii GND2*		0,3	0,26	2,8	0,15
**P12**	*K. phaffii TPI1*	*S. cerevisiae ADH1*	*Y. lipolytica PFK1*		1,0	0,23	3,2	0,75
**P38**	*O. polymorpha SDH1*	*O. polymorpha PGI1*	*Y. lipolytica ENO2*		1,8	0,02	3,9	0,30
**P2**	*K. phaffii ACO1*	*K. phaffii IDP2*	*K. phaffii GND2*		0,4	0,14	4,1	1,20
**P73**	*S. cerevisiae TEF1*	*K. lactis TEF1*	*K. phaffii AOX1*		5,3	0,17	4,1	0,90
**P26**	*Y. lipolytica GPDH*	*O. polymorpha PSH1*	*O. polymorpha IDH2*	*Y. lipolytica GPDH*	1,0	0,16	4,9	0,25
**P28**	*Y. lipolytica TEF1*	*Y. lipolytica ENO2*	*Y. lipolytica PFK1*		2,7	0,13	5,1	0,00
**P47**	*S. cerevisiae PGK1*	*Y. lipolytica GPDH*	*S. cerevisiae PGK1*	*K. phaffii ADH1*	0,9	0,08	5,8	1,00
**P103**	*K. lactis ENO1*	*Y. lipolytica ENO2*	*Y. lipolytica GPDH*		4,9	0,03	5,9	0,55
**P101**	*Y. lipolytica PGK1*	*Y. lipolytica ZWF1*	*Y. lipolytica GND2*		4,7	0,36	6,8	0,20
**P96**	*K. phaffii TPI1*	*Y. lipolytica TPI1*	*Y. lipolytica GND2*		5,1	0,40	8,1	0,51
**P34**	*Y. lipolytica STL1*	*K. marxianus MDH1*	*Y. lipolytica GND2*		5,5	0,46	12,9	0,67
**P25**	*S. cerevisiae PFK2*	*S. cerevisiae ZWF1*	*Y. lipolytica FBA1*		3,9	0,28	13,8	0,78
**P97**	*K. phaffii PMA1*	*K. phaffii PGK1*	*Y. lipolytica FBA1*		5,8	0,51	14,3	0,25
**P45**	*Y. lipolytica ENO2*	*Y. lipolytica GPDH*	*Y. lipolytica ACO1*	*Y. lipolytica ENO2*	6,1	0,59	15,2	0,72
**P46**	*K. phaffii CYC1*	*Y. lipolytica GPDH*	*Y. lipolytica ZWF1*	*K. phaffii CYC1*	7,9	0,93	18,2	0,10
**TEF1**					100,0	6,32	100,0	23,14
**P68**	*S. cerevisiae TEF1*	*C. hispaniensis TEF1*	*Y. lipolytica TEF1*		133,5	24,43	137,2	2,94
**P67**	*Y. lipolytica TEF1*	*Y. lipolytica TEF1*	*C. hispaniensis TEF1*	*Y. lipolytica TEF1*	106,1	2,32	143,0	8,03
**P88**	*Y. lipolytica TEF1*	*K. marxianus TEF1*	*Y. lipolytica TEF1*		119,0	18,97	156,2	0,91
**P89**	*S. cerevisiae TEF1*	*K. marxianus TEF1*	*Y. lipolytica TEF1*		134,5	13,66	168,0	1,99
**P62**	*S. cerevisiae TEF1*	*K. lactis TEF1*	*Y. lipolytica TEF1*		130,1	3,78	174,7	0,78

9. CHARACTERISATION OF STANDARD INTEGRATION LOCI

For each of the 16 standard integration loci described in the main text of the article a Lvl1.1 plasmid was constructed with promoter *TEF1*, gene *hrGFP*, and terminator *LIP2* (Supplementary Table 17). The obtained overexpression constructs (pB8US1.1-hrGFP, pB11US1.1-hrGFP, pC2US1.1-hrGFP, pC7US1.1-hrGFP, pC13US1.1-hrGFP, pC14US1.1-hrGFP, pD6US1.1-hrGFP, pD12US1.1-hrGFP, pE6US1.1-hrGFP, pE8US1.1-hrGFP, pE12US1.1-hrGFP, pE15US1.1-hrGFP, pE16US1.1-hrGFP, pF8US1.1-hrGFP, pF9US1.1-hrGFP, and pF11US1.1-hrGFP) were linearized and co-transformed together with the corresponding standard Cas9-helpers (pCasNA-IntB8, pCasNA-IntB11, pCasNA-IntC2, pCasNA-IntC7, pCasNA-IntC13, pCasNA-IntC14, pCasNA-IntD6, pCasNA-IntD12, pCasNA-IntE6, pCasNA-IntE8, pCasNA-IntE12, pCasNA-IntE15, pCasNA-IntE16, pCasNA-IntF8, pCasNA-IntF9, and pCasNA-IntF11) into the S244 (W29ΔuraΔku70) strain of *Y. lipolytica* (Section 2.6 of Supplementary Manual). Successful integration for each locus was confirmed using colony PCR with standard primer sets (Supplementary Tables 5 and 10 of Supplementary Manual). The efficiency of Cas9-mediated integration for the selected 16 standard loci are summarized in Supplementary Table 5 of Supplementary Manual. Two independent transformants were isolated for each locus and hrGFP fluorescence was assayed in a plate reader as described in Section 8.1. The parental strain S234 (W29Δku70ura+) was used as the negative control. The relative expression levels in the 16 loci are compared in Supplementary Figure 27 and discussed in Section 2.1 of Supplementary Manual.

10. APPENDIX

Supplementary Table 16 Oligonucleotides used in this study (*continued on the next page*)

#	Name	Sequence*
3193	HPD-F	gcatCGTCTCATCGGGGTCTCAAATGTCACCTTCCGTCGAAGTC
3194	HPD-OE-R	CATGGGTGGTCACATCTCGAGAGCCAGTCTCAAG
3195	HPD-OE-F	GCTCTCGAGATGTGACCACCCATGTCGTGGGC
3196	HPD-R	ctgaCGTCTCTGGTCGGTCTCATAGATTAAAGGTTGCCTCGCTTGGCCTG
3459	ALK1-F	gcatGAAGACTCACGGACTGACTTGATACGCAACTG
3460	ALK1-R	atgcGAAGACAGCATTGTGCAGGAGTATTCTGGGGAG
3369	AAT1-Up950-F	atgcGCTCTTCACAGGTTTAAACGCGTCTTTAACAGGCGAAAAAC
3370	AAT1-Up950-R	gataGCTCTTCTTGGCCTCCTTCTCGGCCTCTC
3371	AAT1-Dn950-F	atgcGCTCTTCATAATGTCGAGCAGGGCCACGAG
3372	AAT1-Dn950-R	gataGCTCTTCTTCCGTTTAAACTGTCCTACAGTTTACACAC
3373	AAT1-chr-F2	GTGCGCACTCTCTCACACC
3204	ARO8-Up500-F	CCAGATTTATATACCGAACACC
3205	ARO8-Up500-R	ACTTTTCCGCCTCTTTGTAGTCGTGCTTTTGG
3206	ARO8-Dn500-F	ACGACTACAAAGAGGCGGAAAAGTCTCTGCTC
3207	ARO8-Dn500-R	TCTCTCTCCCACATGTATGG
3212	ARO8-chr-F	CATACAAACATTCCATGTCGC
3213	ARO8-chr-R	GGCCTGTTTCTTGCATTACTC
3208	ARO9-Up500-F	TTTGGTGACGGAATAAGTCTC
3209	ARO9-Up500-R	TACAGAAAACACCGCTGGAAACAGTGATGATATAG
3210	ARO9-Dn500-F	CTGTTTCCAGCGGTGTTTTCTGTATAGTACAAG
3211	ARO9-Dn500-R	ATTGAGTGGCAATTCTGAACC
3214	ARO9-chr-F	GGTTACCTTATCATGCATGTG
3215	ARO9-chr-R	ACGTCTCAAAAGTGTCCAAAC
3308	AAT1-Up500-F	AACCAACCAAATAACCAAATAAC
3309	AAT1-Up500-R	AATCTGAGAGGTGTTTTGAGAGTCTGGTGGAG
3310	AAT1-Dn500-F	AGACTCTCAAAACACCTCTCAGATTGGTATGTTC
3311	AAT1-Dn500-R	GATGCTCGGTTACAGTCTAC
3312	AAT1-chr-F	CTAGTGGTCGACGACAACC
3313	AAT1-chr-R	GTAGTAAACAATAGCTAGTCAG
3296	AAT2-Up500-F	CTCCACCATCTAGTCATCTC
3297	AAT2-Up500-R	CGCATCTCGCAAGGCCGAAAAGGGCATCTG
3298	AAT2-Dn500-F	CTTTTCGGCCTTGCGAGATGCGCTCAAGAG
3299	AAT2-Dn500-R	TCGTGTCTACAAGTTGCTGC
3300	AAT2-chr-F	CCACGGATCCGCTGAAGC
3301	AAT2-chr-R	CCATACTAACAACTAAGCTCTC

3064	TEF-seq-F1	CTGCAGTCTGGAATCTACGC
3066	ARO4-seq-R	GGTTTCTCCAGATAAGCTCTC
3390	ARO7-seq-R	CTCGTCGGGAGACTCGAAG
3149	Rdm1-20bp	TCGATGGGCCCCCGGTTCGATTCCGGGTCGGCGCA**ATATTATTGTACACCTACCG**GTTTTAGAGCTAGAAATAGCAAGTTAAAATAAGGC
3150	Rdm2-20bp	TCGATGGGCCCCCGGTTCGATTCCGGGTCGGCGCA**GCATCAGGTGGACTAGCATG**GTTTTAGAGCTAGAAATAGCAAGTTAAAATAAGGC
3151	Rdm3-20bp	TCGATGGGCCCCCGGTTCGATTCCGGGTCGGCGCA**ATGGACGAAATGCTTCACCA**GTTTTAGAGCTAGAAATAGCAAGTTAAAATAAGGC
3216	ARO8a-20bp	TCGATGGGCCCCCGGTTCGATTCCGGGTCGGCGCA**GCACGACATTCTCATCATCG**GTTTTAGAGCTAGAAATAGCAAGTTAAAATAAGGC
3217	ARO8c-20bp	TCGATGGGCCCCCGGTTCGATTCCGGGTCGGCGCA**GAACAGCTCAACAACTGGGT**GTTTTAGAGCTAGAAATAGCAAGTTAAAATAAGGC
3218	ARO8i-20bp	TCGATGGGCCCCCGGTTCGATTCCGGGTCGGCGCA**GTCCGTCGGTATCAAAGTTG**GTTTTAGAGCTAGAAATAGCAAGTTAAAATAAGGC
3219	ARO9a-20bp	TCGATGGGCCCCCGGTTCGATTCCGGGTCGGCGCA**AGAAGCAGTACTCCTCAACA**GTTTTAGAGCTAGAAATAGCAAGTTAAAATAAGGC
3220	ARO9i-20bp	TCGATGGGCCCCCGGTTCGATTCCGGGTCGGCGCA**GACAACATTAAATCTGTCGG**GTTTTAGAGCTAGAAATAGCAAGTTAAAATAAGGC
3221	ARO9k-20bp	TCGATGGGCCCCCGGTTCGATTCCGGGTCGGCGCA**GTGGACCTGCTCATTAACCC**GTTTTAGAGCTAGAAATAGCAAGTTAAAATAAGGC
3306	AAT1-20bp	TCGATGGGCCCCCGGTTCGATTCCGGGTCGGCGCA**GCAGTGGCGAAAGATTGAGG**GTTTTAGAGCTAGAAATAGCAAGTTAAAATAAGGC
3302	AAT2-20bp	TCGATGGGCCCCCGGTTCGATTCCGGGTCGGCGCA**AAGCAGATCTTCGAGAACGT**GTTTTAGAGCTAGAAATAGCAAGTTAAAATAAGGC
3006	URA3-20bp	TCGATGGGCCCCCGGTTCGATTCCGGGTCGGCGCA**CACGAGCTTGAGCACTCGAG**GTTTTAGAGCTAGAAATAGCAAGTTAAAATAAGGC
3295	HMG2-20bp	TCGATGGGCCCCCGGTTCGATTCCGGGTCGGCGCA**CACTTCGATATTCCTGACCG**GTTTTAGAGCTAGAAATAGCAAGTTAAAATAAGGC

* – The variative region of recombineering oligonucleotides is underlined.

Supplementary Table 17 Assembly of plasmid constructs (*continued on the next page*)

Module (Lvl)	Assembled plasmid	Backbone plasmid	Part 1	Part 2	Part 3	Manual section (or GenBank provided)
Exp (Lvl0)	pGenC-YlHPD1	pYTK001	OE-PCR from W29 primers 3193, 3194, 3195, 3196			1.2
Exp (Lvl0)	pGenA-ScARO4^{K229L}	Gene synthesis				(GenBank)
Exp (Lvl0)	pGenK-ScARO7^{G141S}	Gene synthesis				(GenBank)
Exp (Lvl1)	pZUS1.1-HPD1	pZUS1.1	pProC-TEF1	pGenC-YlHPD1	pTerC-LIP2	1.3.3
Exp (Lvl1)	pZUS1.2-ARO4	pZUS1.2	pProC-TEF1	pGenA-ScARO4^{K229L}	pTerC-LIP2	1.3.3
Exp (Lvl1)	pZUS1.3-ARO7	pZUS1.3	pProC-TEF1	pGenK-ScARO7^{G141S}	pTerC-LIP2	1.3.3
Exp (Lvl1)	pB8US1.1-hrGFP	pB8US1.1	pProC-TEF1	pGenC-hrGFP	pTerC-LIP2	1.3.3
Exp (Lvl1)	pB11US1.1-hrGFP	pB11US1.1	pProC-TEF1	pGenC-hrGFP	pTerC-LIP2	1.3.3
Exp (Lvl1)	pC2US1.1-hrGFP	pC2US1.1	pProC-TEF1	pGenC-hrGFP	pTerC-LIP2	1.3.3
Exp (Lvl1)	pC7US1.1-hrGFP	pC7US1.1	pProC-TEF1	pGenC-hrGFP	pTerC-LIP2	1.3.3
Exp (Lvl1)	pC13US1.1-hrGFP	pC13US1.1	pProC-TEF1	pGenC-hrGFP	pTerC-LIP2	1.3.3
Exp (Lvl1)	pC14US1.1-hrGFP	pC14US1.1	pProC-TEF1	pGenC-hrGFP	pTerC-LIP2	1.3.3
Exp (Lvl1)	pD6US1.1-hrGFP	pD6US1.1	pProC-TEF1	pGenC-hrGFP	pTerC-LIP2	1.3.3
Exp (Lvl1)	pD12US1.1-hrGFP	pD12US1.1	pProC-TEF1	pGenC-hrGFP	pTerC-LIP2	1.3.3
Exp (Lvl1)	pE6US1.1-hrGFP	pE6US1.1	pProC-TEF1	pGenC-hrGFP	pTerC-LIP2	1.3.3
Exp (Lvl1)	pE8US1.1-hrGFP	pE8US1.1	pProC-TEF1	pGenC-hrGFP	pTerC-LIP2	1.3.3
Exp (Lvl1)	pE12US1.1-hrGFP	pE12US1.1	pProC-TEF1	pGenC-hrGFP	pTerC-LIP2	1.3.3
Exp (Lvl1)	pE15US1.1-hrGFP	pE15US1.1	pProC-TEF1	pGenC-hrGFP	pTerC-LIP2	1.3.3
Exp (Lvl1)	pE16US1.1-hrGFP	pE16US1.1	pProC-TEF1	pGenC-hrGFP	pTerC-LIP2	1.3.3
Exp (Lvl1)	pF8US1.1-hrGFP	pF8US1.1	pProC-TEF1	pGenC-hrGFP	pTerC-LIP2	1.3.3
Exp (Lvl1)	pF9US1.1-hrGFP	pF9US1.1	pProC-TEF1	pGenC-hrGFP	pTerC-LIP2	1.3.3
Exp (Lvl1)	pF11US1.1-hrGFP	pF11US1.1	pProC-TEF1	pGenC-hrGFP	pTerC-LIP2	1.3.3
Exp (Lvl2)	pZUA2.3-HPD1-ARO4-ARO7	pZUA2.3	pZUS1.1-HPD1	pZUS1.1-ARO4	pZUS1.1-ARO7	1.3.4
Pro	pProUA-ALK1	pProUA-mScarlet	PCR from W29 primers 3459 & 3460			1.8
Del	pDelUK-AAT1	pDelUK-RG	PCR from W29 primers 3369 & 3370	PCR from W29 primers 3371&3372		1.4
Int	pE8US-HPD1-ARO4-ARO7	pE8US1.1	pZUA2.3-HPD1-ARO4-ARO7			1.5.1
Int	pDelUK-AAT1::HPD1-ARO4-ARO7	pDelUK-AAT1	pE8US-HPD1-ARO4-ARO7			1.5.2
Int/MEx	pE8A1.1-HPD1	pE8UA2.2	pZUS1.1-HPD1			1.5.1/1.6
Int/MEx	pE8A1.2-ARO4	pE8UA2.2	pZUS1.2-ARO4			1.5.1/1.6
Int/MEx	pE8A1.3-ARO7	pE8UA2.2	pZUS1.3-ARO7			1.5.1/1.6
Int/MEx	pE8S-HPD1-ARO4-ARO7	pE8US1.1	pZUA2.3-HPD1-ARO4-ARO7			1.5.1/1.6
MEx	pC2S1.1-hrGFP	pC2US1.1-hrGFP				1.6
MEx	pD12S1.1-hrGFP	pD12US1.1-hrGFP				1.6
MEx	pE8S1.1-hrGFP	pE8US1.1-hrGFP				1.6
Cas	pCasNA-Rdm1	pCasNA-RK	Oligo 3149			1.7
Cas	pCasNA-Rdm2	pCasNA-RK	Oligo 3150			1.7
Cas	pCasNA-Rdm3	pCasNA-RK	Oligo 3151			1.7
Cas	pCasNA-ARO8a	pCasNA-RK	Oligo 3216			1.7
Cas	pCasNA-ARO8c	pCasNA-RK	Oligo 3217			1.7
Cas	pCasNA-ARO8i	pCasNA-RK	Oligo 3218			1.7
Cas	pCasNA-ARO9a	pCasNA-RK	Oligo 3219			1.7
Cas	pCasNA-ARO9i	pCasNA-RK	Oligo 3220			1.7
Cas	pCasNA-ARO9k	pCasNA-RK	Oligo 3221			1.7
Cas	pCasNA-AAT1	pCasNA-RK	Oligo 3306			1.7
Cas	pCasNA-AAT2	pCasNA-RK	Oligo 3302			1.7
Cas	pCasNA-URA3	pCasNA-RK	Oligo 3006			1.7
Cas	pCasNA-HMG2	pCasNA-RK	Oligo 3295			1.7

Structure of hybrid promoters

Sequence and structure of 43 hybrid promoters tested in the study are provided. Each promoter consists of several fragments which are parts of natural promoters derived from seven different yeast genomes. The source of each fragment (yeast species and gene name) is specified above the sequence panel. Corresponding DNA fragments and sources are highlighted using the same colours.

Hybrid promoter P1

S. cerevisiae PFK2 *O. polymorpha PYK1* *K. phaffii GND2*

AAAACGAAGATTAAGATAAAGTTGGGTAAAATCCGGGGTAAGAGGCAAGGGGGTAGAGAAAAAAAAACC
GGAGTCATTATATACGATACCGTCCAGGGTAAGACAGTGATTTCTAGCTTCCACTTTTTTCAATTTCTTTTTTT
CGTTCCAAATGGCGTCCACCCGTACATCCGGAATCTGACGGCACAAGAGCCGATTAGTGGAAGCCACGGTT
ACGTGATTGCGGTTTCGCGGCGACCGTCGCACTATGTCCCGCACGCGCCGCTTGGTAGACTGGCCAGCGTC
GTCCTGGCTGTGTGCATCGCTTTTGTTGAGCTCGTGTTGGAATTTCAGTATAAGCGATTCCGTCAGACTCACT
GCATCTGGGTCAGAGGGAGGCTTTAGGACAGGAGCCATCTGTACAGAGGCTACGGAGTGTGGTGGCGGG
TTCATGGGTGGCTCAAGCGGTCTAAAAGCAAAGGTGCGCGGCCGTACGTTATTGTTTGTGTGGGTACGCGA
TAAATAAAAAATCAGACAATCGGCTATGGGGGTGACATAAGCGATGAGCAAACTCTATAGCCTCGGCAAAC
AGTCTCTTCCCTTGTCCCTCATTGATACCTCTTTATTCTCCCCCACCACCATACACTACCTTCCTCGCACCCCTG
TCATCACAACCGCAATATAATTGATGCGCGGTTTCTTGCCTAATCCATCGTCCAACAGAGAGGTCGCTCTCCT
TATATATATAGTTGATCCCCCTTTTTTTCTACCCTTGCAATTTTTTTTTGGGACCAAAGAAAAGAAACAAGACT
GATACAAAA

Hybrid promoter P2

K. phaffii ACO1 *K. phaffii IDP2* *K. phaffii GND2*

GGTAAATACTTTTCTAGTGCACCCCGACATTCGACTCAAAAGGCTTAAACTAAACTCCTAAAATGTCCGTGG
TTGACCAATAGAAAGTATCACTCAGCTCCCTGATTGTTCATAGCCTAACTGTTTCTGAATCTCTCCAAGTTTAT
TGCTGTCGGGTGAGCCTATGATTATCCCCTTTCACAATAGGCTCATTGTGTCTTAGGAAGTACCTGCCCACTT
CCCCCTGATAAACTTTCCACCATCCCCGGTCATTCGCTCATGACCTTGTTATTACCATGCCAAAACATCCCATA
ATGAAAGGGTATCGGCAACATGGGAGCTAAATTTCAGACCCTCGAGATGGAGTCGGTAATCGTTCGAGAA
CAATTTAACAGGTACAATGGTTATATGCAAGGAGCCGAGAAATTAACTTGGAGTTTTGGTTCTGTTTGGAGT
TGTCTCCGTTGGAGGAACAGAATACTAAACTTACTTGCAACAATTAATGAATAGGGTGTAGGTTTTGGACAT
GAATACTATTGGTAGATGATAAAGAGTGAGGTCGTAGCTTGTAGTAACTGTAATCGTTAAGGGGTGTTCTT
GATGATCTTGTATTGATACCTCTTTATTCTCCCCCACCACCATACACTACCTTCCTCGCACCCCTGTCATCACA
ACCGCAATATAATTGATGCGCGGTTTCTTGCCTAATCCATCGTCCAACAGAGAGGTCGCTCTCCTTATATATA
TAGTTGATCCCCCTTTTTTTCTACCCTTGCAATTTTTTTTTGGGACCAAAGAAAAGAAACAAGACTGATACAA
AA

Hybrid promoter P3

K. phaffii MDH1 *K. phaffii PFK1* *K. phaffii GND2*

TGAGGTGAAATATTCTTTAGTAATCTAGCAGGGAATCCTTGGAAGGGTAATTTAGACGGAAGACGTCATCT
TGCGGAAGGGTGAAACATTCCAAGCAGAGTGCTGGTTCCGAGATTCTCCGCTCCTGTAGAAACCTTCAACC
TTCCAACATTAGAACCTTCGCGAATAGACTCCCTATCCAATCACGTTTCTCCGTTTCAAGGAAGTAGCCTGGG
TGTCTATGGTTAATATGTTCCTGCAGCGCGACTAATACTACGAATTTGCGCGCCTGCAACTCTCCTCTCTCAG
TCTGGTTCTTGAAGATGTGTATAAGTCTCGCAATGAAACAAATGTTCTGGGAGATGGATCGTTGCTCAATAT
GAATATCGGATTAGTAAAGTATAAACTTATAAGACTTTCAGTGGTTTTCACATTGACCTAGGCTTCTTATAAC
AAGGCGACTCCGAGAACAAAAGAAAAATAGAATGGCCCTCAAAGTAGCTTATGTGAGATCTGGGCTTGATT
CCAATCATAGTCCCTAGTTGATTGCTTGTAGCAAATGCCACAACAGTAGGCATTTACGTCCTCACAGTCTCTT

CCCTTGTCCCTCATTGATACCTCTTTATTCTCCCCCACCACCATACACTACCTTCCTCGCACCCCTGTCATCACA
ACCGCAATATAATTGATGCGCGGTTTCTTGCCTAATCCATCGTCCAACAGAGAGGTCGCTCTCCTTATATATA
TAGTTGATCCCCCTTTTTTTCTACCCTTGCAATTTTTTTTTGGGACCAAAGAAAAGAAACAAGACTGATACAA
AA

Hybrid promoter P4
K. phaffii TPI1 *O. polymorpha ENO2* *K. phaffii GND2*

GAAATATACCACATTGCCAGTTTATACAGATGGTTAAGGGTGAAAATCAACGTTACACCTTGACGACCCCAT
TATTACGATGGCGTGAAGGAGATGAAGACCGGGTAGAAGAAATAAGAAAAGCGGTACAGTTTAGGTCCG
GAGATCTAGGGAAGGAGGCCTTAGCTTATATTGTAGCTGCTGAGAGAGAGGCAGCTGCTGGAAGATCTGA
AGGCCCTATCAACCTCCTTTGAAAGGTCCCCTCTGGCAACAATAGTCAAATTTGTGCCGCGTTCTGGAGGGT
TAGGCGCAACATCCACATGAGTCAGTTTGAGGAGTTGAGAAGACGAGGAGTCGCATAGAAGAAGGGGAG
AGTCGCCCGGAACAGGCTTGGTGTCCTGGCCTGGAAAAGCAAACGGGTTGAGCGACAGACCGTTGACAAG
AGAGAGCAAAAACACTAAAGGCACAGGGAAAAGCATTAGGAAGCAAGCACTGAAATTGTTGGCCTTAAAA
GTCAATTGAGTTCCAATCATAGTCCCTAGTTGATTGCTTGTAGCAAATGCCACAACAGTAGGCATTTACGTC
CTCACAGTCTCTTCCCTTGTCCCTCATTGATACCTCTTTATTCTCCCCCACCACCATACACTACCTTCCTCGCAC
CCCTGTCATCACAACCGCAATATAATTGATGCGCGGTTTCTTGCCTAATCCATCGTCCAACAGAGAGGTCGC
TCTCCTTATATATATAGTTGATCCCCCTTTTTTTCTACCCTTGCAATTTTTTTTTGGGACCAAAGAAAAGAAAC
AAGACTGATACAAAA

Hybrid promoter P5
O. polymorpha ACO1 *K. phaffii PGK1* *K. phaffii GND2*

GGTCACAATGTTTAAAGCTCTGTCGGTAAATTGTGATTGATCCATGATTAGCAAAATTGCAGTTGTAGGGAA
AGCAATTTATACAGCGTACAGACATCAGACGCTTGTGATGGTCTGGTGATCTAGAGCCGCACAGAAACTTC
TAGCAATATCTGGTGTGTCTGGGTGGTTGCTGGTGTCTGGTCTGCATAACTTGGCCTGAGGATGCTTATGTA
ATATGGCCTTCCTGGTGCATGGAAGTGGCTGGTGGCAAAAAAAAGTCTGTGAGCCTCCCAAGCAATCTACT
AATGTTTATTTTTCGTCCAACCTAATTGTGGTTTCAAAGCGCTATCAGGTGGGGGGTAAGAGGAATGTGAG
TGGAAAGCGAAAATAACTGGCAGCTGGGGTCAGATCCCGTGATGCCACCTCTTGTGGTATTTTGAAACGCG
TGTTGCGATTGGCCGCGAGAACGGAAAGGAATATATTTACTGCCGATCGCATTTTGGCCTCAAATAAATCTT
GAGCTTTTGGACATAGATTATATGTTCTTTCTTGGAAGCTCTTTCAGCTAATAGTGAAGTGTTTCCTACTAAG
GATCGCCTCCAAACGTTCCAACTACGGGCGGAGGTTGCAAAGAAAACGGATCTCTCAGCGAATTGTTCTCA
TCCATGAGTGAGTCCTCTCCGTCCTTTCCTCGCGCCTGCCTAATCCATCGTCCAACAGAGAGGTCGCTCTCCT
TATATATATAGTTGATCCCCCTTTTTTTCTACCCTTGCAATTTTTTTTTGGGACCAAAGAAAAGAAACAAGACT
GATACAAAA

Hybrid promoter P8
O. polymorpha TPI1 *O. polymorpha PYK1* *K. phaffii ADH1*

TGTGTTGGTGCTGGTGACTCATGGAATATGGGCGCGTGTCTTCCTGACCAAATGGTTTAGATGGAGCGTAG
AAAAGTTCGAAGACCTGCAAAACGTGCCTAACTGTCGCTGGATAGTGATGGAAAAGGACGACACGACGCA
GCGTTACGTCCTTCGCACGCATCTGAGCACGTGGTCAGAGCTTGAACAGACAAAAAGAGAGGAAGAAGTC
AAAAGAGATGCCGGGAGAGAGTTTACATTCAACAGTACAGTGCCTCTCACAGACGACGAGGTAAGACAGG
TAGCCGACGCGGAGGCGCGAGCGAAAAACGACCAGGTGCAAAAATCGCTAAAAATAAGCGATTCCGTCAG
ACTCACTGCATCTGGGTCAGAGGGAGGCTTTAGGACAGGAGCCATCTGTACAGAGGCTACGGAGTGTGGT
GGCGGGTTCATGGGTGGCTCAAGCGGTCTAAAAGCAAAGGTGCGCGGCCGTACGTTATTGTTTGTGTGGG
TACGCGATAAATAAAAAATCAGACAATCGGCTATGGGGGTGACATAAGCGATGAGCAAACTCTATAGCCTC
GGCAAACCTGGGCGTGCACGGATGTCGTCGGAGTGCAATTTTCCAGCGGACCAAAGTTCACCAGGAAAAA

AATGACCCAATATGGGCGGTGCCCAATGATCACACCAACAATTGGTCCACCCCTCCCCAATCTCTAATATTC
ACAATTCACCTCACTATAAATACCCCTGTCCTGCTCCCAAATTCTTTTTTCCTTCTTCCATCAGCTACTAGCTTT
TATCTTATTTACTTTACGAAA

Hybrid promoter P10

K. phaffii MDH1 *O. polymorpha ENO2* *O. polymorpha TAL1*

TGAGGTGAAATATTCTTTAGTAATCTAGCAGGGAACTCTTGGAAGGGTAATTTAGACGGAAGACGTCATCT
TGCGGAAGGGTGAAACATTCCAAGCAGAGTGCTGGTTCCGAGATTCTCCGCTCCTGTAGAAACCTTCAACC
TTCCAACATTAGAACCTTCGCGAATAGACTCCCTATCCAATCACGTTTCTCCGTTTCAAGGAAGTAGCCTGGG
TGTCCGGTACGCCAAAAAACCTCCTTTGAAAGGTCCCCTCTGGCAACAATAGTCAAATTTGTGCCGCGTTCT
GGAGGGTTAGGCGCAACATCCACATGAGTCAGTTTGAGGAGTTGAGAAGACGAGGAGTCGCATAGAAGA
AGGGGAGAGTCGCCCGGAACAGGCTTGGTGTCCTGGCCTGGAAAAGCAAACGGGTTGAGCGAGACACCG
TTGACAAGAGAGAGCAAAAACACTAAAGGCACAGGGAAAAGCATTAGGAAGCAAGCACTGAAATTAGAA
AGGAGGCAAATAATTTGTCACCCAGCTGGCTCCTAAAATGGAAATGCATGTGGCAAAGTAATGGCTCCTAG
GGTGGACTTCAAGGATACACTCACATTGATTGGTTGACAAGGTTACGTAAACTTGTCTCCGAAGACGAATTT
GTTTACGAAGATAGCATTTATTTGTAGGTTATTGATTAGGTAAGATTTATTCAACGCAAACTGGGTGATGAA
TATAAATAAGCTCGGGTATTCCTCCGCAACCAGAACTTGAATTATATACGTTTCGATTGATTTCCATCAAATT
ATAAATTCAACAATTGCA

Hybrid promoter P11

S. cerevisiae ENO1 *K. phaffii GPM1* *Y. lipolytica GPDH*

AGTTAGTAGCAACCTGAACTCGGTCATTGATGCATGCATGTGCCGTGAAGCGGGACAACCAGAAAAGTCGT
CTATAAATGCCGGCACGTGCGATCATCGTGGCGGGGTTTTAAGAGTGCATATCACAAATTGTCGCATTACC
GCGGAACCGCCAGATATTCATTACTTGACGCAAAAGCGTTTGAAATAATGACGAAAAAGAAGGAAGAAAA
AAAAAGAAAAATACCGCTTCTAGGCGGGTTATCTACTGATCCGAGCTTCCACTAGGATAGCACCCAAACAG
CTGCATATTTGGACGACCTTTACCAAATGAGAACCCTGTATTTCCTTGGCCAGAGCCTGAGAAACTGAACCC
TGATCCAGACATTATCAATACCTTGGGTTATTAGTAGTGTCCGTTATTTTTCTGTTTAGGTTACGATTTTGCCA
GATTTTTTGGGAGGAGGGAAACAAAAGAACCAGTGCTACACGACCTTTAAGTGCCATCAGGCATCCTGTTT
TCTCGACCTCATCTCATCACATCCGTCAGTCTGAGCTTTCAGTTCTCAGTTTTCGATTGACTCTTGCCCTGCTG
CGCGCACACCATACCCTGGCTCCCTCTCATGCTTCTGGCGTTACCCCGGAATCGTACATCCATGCCGCGAATC
CCGGACAGGACTCAGACGGATTTCACTATTTGGGCGGGCTTGCTCCGTCTGTCCAAGGCAACATTTATATAA
GGGTCTGCATCGCCGGCTCAATTGAATCTTTTTTCTTCTTCTCTTCTCTATATTCATTCTTGAATTAAACACAC
ATCAACA

Hybrid promoter P12

K. phaffii TPI1 *S. cerevisiae ADH1* *Y. lipolytica PFK1*

GAAATATACCACATTGCCAGTTTATACAGATGGTTAAGGGTGAAAATCAACGTTACACCTTGACGACCCCAT
TATTACGATGGCGTGAAGGAGATGAAGACCGGGTAGAAGAAATAAGAAAAGCGGTACAGTTTAGGTCCG
GAGATCTAGGGAAGGAGGCCTTAGCTTATATTGTAGCTGCTGAGAGAGAGGCAGCTGCTGGAAGATCTGA
AGGCCCTATCACGTATGATGATGGTGATGACCATTAGAGAACGCCCAGAGATTGATAGCCAGTTCTTGGAC
AACAATTCGGAACTTTATTCACGGTGCAAGTTTCACTACCCTTTTTCCATTTGCCATCTATTGAAGTAATAATA
GGCGCATGCAACTTCTTTTCTTTTTTTTTCTTTTCTCTCTCCCCCGTTGTTGTCTCACCATATCCGCAATGACAA
AAAAATGATGGAAGACACTAAAGGAAAAAATTAACGACAAAGACAGCACCAACAGATGTCGTTGTTCCAG
AGCTGATGAGGGGTATCTCGAAGCACACGAAACTTTTTCCTTCCTTCATTCACGCACACTACTCTCTAATGAG
CAACGGTATACGGCCTTCCTTCCAGTTACTTGAATTTGAAATAAAAAAAAGTTTGCTGTCTTGCTATCAAGTA
TAAATAGACCTGCAATTATTAATCTTTTGGCCGGCGTAACTTTGAGTGTCGTCAAACTCTGATATATATATAG

AGAGAGGTATCCCAACAGTTGATAGTCGACAAACGCAAAACAGACGGACACTGAACCCCCCGCGCTTCAAA
ACACCGACA

Hybrid promoter P13
Y. lipolytica PGK1 *K. marxianus MDH1* *K. phaffii AOX1*

CAGACAGTGACGAGTCATACATTCTCCGTATAATATCGTGTATGTCCAGACGATAGTCGTACTCGTACTCGT
TACTGTAACTACTGTGCGAGTACTCGTGCATGTATCGTAGGTATTGTATGTTCGAGTACATACACATACGAT
ACCAAACACTGCCCACTGTTCTGTCATGTTAGATCATGGCCAATCCACGTGACTTGCATGCAGGTTTGGCAT
TGAATATTCAGCGTGGCTACTACAAGTAGTACATACTGTATCAATACGATTGTACATACGGTACTCACCCTTT
GCTACAGTATGTACATACAAGGGCGCACATGGACCTTGCCCTTTTGACTAGGGTACTACTCTGGGCAGGGT
ACTTACTAAACTGGGCCCAGACAGACAGAGGCCCAGACAGAGGCCCCAAAGAGAAGCCGCCAGGTTCCCT
GCCTAGGCCTTCCTCTGGTTGCCCCCCAGAGTACCCTCCGGAACGAAACGAAACGAAAACGCACATCGTGA
TGCGCGCTATTATAATTGCGTCTTGCGAATTCCATACGCCATGCGCTTAGTTAGCACATGAAGATTCTGGTG
GGAATACTGCTGATAGCCTAACGTTCATGATCAAAATTTAACTGTTCTAACCCCTACTTGACAGCAATATATA
AACAGAAGGAAGCTGCCCTGTCTTAAACCTTTTTTTATCATCATTATTAGCTTACTTTCATAATTGCGACTG
GTTCCAATTGACAAGCTTTTGATTTTAACGACTTTTAACGACAACTTGAGAAGATCAAAAAACAACTAATTAT
TCGAAACA

Hybrid promoter P14
Y. lipolytica GPDH *O. polymorpha ENO2* *K. phaffii AOX1*

GTAGGTTGGGTTGGGTGGGAGCACCCCTCCACAGAGTAGAGTCAAACAGCAGCAGCAACATGATAGTTGG
GGGTGTGCGTGTTAAAGGAAAAAAAAAAGAAGCTTGGGTTATATTCCCGCTCTATTTAGAGGTTGCGGGATA
GACGCCGACGGAGGGCAATGGCGCCATGGAACCTTGCGGATATGGGGACGCCGCGGCGGACTGCGTCCG
AACCAGCTCCAGCAGCGTTTTTTCCGGGCCATTGAGCCGACTGCGACCCCGCCAACGTGTCTTGGCCCACGC
ACTCATGTCATGTTGGTGTTGGGAGGCCACTTTTTAAGTAGCACAAGGCACCTAGCTCGCAGAAGGGGAGA
GTCGCCCGGAACAGGCTTGGTGTCCTGGCCTGGAAAAGCAAACGGGTTGAGCGAGAGAGCGTTGACAAG
AGAGAGCAAAAACACTAAAGGCACAGGGAAAAGCATTAGGAAGCAAGCACTGAAATTAGAAAGGAGGCA
AATAATTTTTGAAAGCGACAATGCGTCGACAGAATCTGCCGACAGAAAGATTGTCGACAAGCGACGATAAC
GCAATAGTACATGGTGTTATTACGCAGTGTAACGTTCATGATCAAAATTTAACTGTTCTAACCCCTACTTGAC
AGCAATATATAAACAGAAGGAAGCTGCCCTGTCTTAAACCTTTTTTTATCATCATTATTAGCTTACTTTCATA
ATTGCGACTGGTTCCAATTGACAAGCTTTTGATTTTAACGACTTTTAACGACAACTTGAGAAGATCAAAAAA
CAACTAATTATTCGAAACA

Hybrid promoter P23
Y. lipolytica TEF1 *Y. lipolytica ZWF1* *K. marxianus CYC1*

GGTATTTTCACAATTGCACCCCAGCCAGACCGATAGCCGGCCGCAATCCGCCACCCACAACCGTCTACCTCC
CACAAAAAGAAGTCACTTCCACCCTTTTCCACCAGATCATATGTCCCAACTTGCCAAATTAAAACCGTGCGAA
TTTTCAAAATAAACTTTGGCAGGAAGGCTGCAAAGGAGGGGCTGGTGAGGGCGTCTGGAAGTCGACCACA
CACCGGGTTGGCGGCGTATTTGTGTCCCAAAAAACAGCCCCAATTGCCCCAATTGACCCCAAATTGACCCAG
TAGCGGGCCCAACCCCGGCGAGAGCCCCCTTCACCCCACATATCAAACCTCCCCCGGTTCCCACACTTGCCG
TTAAGGGCGTAGGGTACTGCAGTCTGGATAGTTCACCAGTCACGCTGGTGGGCCATTCTCTTAGCACATTTC
CCCCCTCCCAAGTCCCCTCAACCCCCAATGTAACCCTCAACCTCCCACAGCTCAGTAGCACACGTGCAACTAG
TTAGTAACAACCCCCCTCCGTCCAGCTTCTCTTTCACACTGCTTAGAGTTCGGGTGCTACGAAATAGAACGCT
CATAGTAGATGAAAAATTTCGTCAAAGCTAAAGCATTAATGCAGCATTTTGATATATATAAGATGCTTCAAG
AAGAAAAAAAAAGTCTTGAACTTTTATTCTTTACACTTGAGTGTGTTTTAACATTCAAGCTAATCTGTTTAGT

CAGTTTATAAACATCAAGCAACTGTTTGAATTTTATCATACGCAGCTAAACCAAACACACATTATTTATAGAT
AAGAA

Hybrid promoter P24
Y. lipolytica GPDH *Y. lipolytica ENO2* *K. marxianus TAL1*

GTAGGTTGGGTTGGGTGGGAGCACCCCTCCACAGAGTAGAGTCAAACAGCAGCAGCAACATGATAGTTGG
GGGTGTGCGTGTTAAAGGAAAAAAAAAGAAGCTTGGGTTATATTCCCGCTCTATTTAGAGGTTGCGGGATA
GACGCCGACGGAGGGCAATGGCGCCATGGAACCTTGCGGATATCCATACGCCGCGGCGGACTGCGTCCGA
ACCAGCTCCAGCAGCGTTTTTTCCGGGCCATTGAGCCGACTGCGACCCCGCCAACGTGTCTTGGCCCACGCA
CTCATGTCATGTTGGTGTTGGGAGGCCACTTTTTAAGTAGCACAAGGCACCTAGCTCGCAGCAAGGTGTCC
GAACCAAAGAAGCGGTCCCATCGGTAATCACGTGTGTGCCGATTTGCAAGACGAAAAGCCACGAGAATAA
ACCGGGAGAGGGGATGGAAGTCCCCGAACAGCAACCAGCCCTTGCCCTCGTGGACATAACCTTTCACTTGC
CAGAACTCTAAGCGTCACCACGGTATACAAGCGCACGTAGAAGATTGTGGAAGTCGTGTTGGAGACTGTTG
ATTTGGCTCTTGCCCATCCATTTTACTCATTATATAATCTAGCTTTTGAAATATAGGAAATAGGTATATAAAG
GATAGGAAAAAAAATTGTAATTGCAGAAAAAGAGGTGAACACGGTTAAAAACAGGTGAAAAACAGTTGTC
AATTGTTGATGGATTGTTAGTAATTGATAGAATTTTCGATTAGAAATTGTCTATTACCAATTGAGAATAAACA
CCACCAAACGATACAAGA

Hybrid promoter P25
S. cerevisiae PFK2 *S. cerevisiae ZWF1* *Y. lipolytica FBA1*

AAAACGAAGATTAAGATAAAGTTGGGTAAAATCCGGGGTAAGAGGCAAGGGGGTAGAGAAAAAAAAACC
GGAGTCATTATATACGATACCGTCCAGGGTAAGACAGTGATTTCTAGCTTCCACTTTTTTCAATTTCTTTTTTT
CGTTCCAAATGGCGTCCACCCGTACATCCGGAATCTGACGGCACAAGAGCCGATTAGTGGAAGCCACGGTT
ACGTGATTGCGGTTTTTTTTTCCTACGTATAACGCTATGACGGTAGTTGAATGTTAAAAACGAAAACAGAGA
TATTGAATTGATCAATTTGATCAGTACGTATGTAATCTTTTGTTTGCCATGTTAAATCGGCCTGAAATCACCA
CAAACTGTGTGTATCAAGTACATAGTGACATTTATATAATAGCAAGAACAACAATAATAGTAGCGCTACTGG
AAGCACCACGTAATAGTGGAAAAGAACTGGAAAAACCGCTATAAGATGCATACTCCGGCGGTCTTACGCG
GAGATACAAGCTTCCAACGGTGCTAAAAGCCCGGTAGTGTACTTCAATCGCCCCCTGGATATAGCCCCGAC
AATAGGCCGTGGCCTCATTTTTTTGCCTTCCGCACATTTCCATTGCTCGGTACCCACACCTTGCTTCTCCTGCA
CTTGCCAACCTTAATACTGGTTTACATTGACCAACATCTTACAAGCGGGGGGCTTGTCTAGGGTATATATAA
ACAGTGGCTCTCCCAATCGGTTGCCAGTCTCTTTTTTCCTTTCTTTCCCCACAGATTCGAAATCTAAACTACAC
ATCACACA

Hybrid promoter P26
Y. lipolytica GPDH *O. polymorpha PSH1* *O. polymorpha IDH2* *Y. lipolytica GPDH*

GTTGGGGTTACGTAATTGCGGCATTTGGGTCCTGCGCGCATGTCCCATTGGTCAGAATTAGTCCGGATAGG
AGACTTATCAGCCAATCACAGCGCCGAATCCACCTGTAGGTTGGGTTGGGTGGGAGCACCCCTCCACAGAG
TAGAGTCAAACAGCAGCTCGCGACTCTCTGTCCGTTTCAGACTACACGAACATGCTTCTGGATGCCGTCAAG
TTCCATAACCATGAGTTTGGCATGGTAGTTTTGGACAGAATGGCAAAGGAATCGCCGCGCAGCAACACCGA
ACTTCTCAGGGCTGTGTACGCCATAATCAAGTTTTACCTTGAGCTAGGGCGTCTGGACCTTGCGATGCATAT
ACTAGAGCAGGTCAAGAAGGAAAGTATGGGGATGTGGCAAACGATTCCATCGTTCTTACCAGCTGACAGG
TCACTGGAAGAGCAATAGGTCGCAAAAGAACTGCCAAATCCCGCCGGACTTTGACTACACTCCGATTAAGT

TGTCGCGCGATACGTAGACTTTAGATGCCAATACGATGTGTGCTATGTTTATGTAAGCGGGTCGATCGACCC
CTGTCTCGTTGTATGCTCGCCAACGCCCGGTCTTTTGCACCACATCAGGTTACCCCAAGCCAAACCTTTGTGT
TAAAAAGCTTAACATATTATACCGAACGTAGGTTTGGGCGGGCTTGCTCCGTCTGTCCAAGGCAACATTTAT
ATAAGGGTCTGCATCGCCGGCTCAATTGAATCTTTTTTCTTCTTCTCTTCTCTATATTCATTCTTGAATTAAAC
ACACATCAACA

Hybrid promoter P28
Y. lipolytica TEF1 *Y. lipolytica ENO2* *Y. lipolytica PFK1*

GGTATTTTCACAATTGCACCCCAGCCAGACCGATAGCCGGCCGCAATCCGCCACCCACAACCGTCTACCTCC
CACAGAACCCCGTCACTTCCACCCTTTTCCACCAGATCATATGTCCCAACTTGCCAAATTAAAACCGTGCGAA
TTTTCAAAATAAACTTTGGCAAAGAGGCTGCAAAGGAGGGGCTGGTGAGGGCGTCTGGAAGTCGACCACA
GAGCGGGTTGGCGGCGTGGCACCCCGGAAAAAAACAGCCCCAATTGCCCCAATTGACCCCAAATTGACCCA
GTAGCGGGCCCAACCCCGGCGAGAGCCCCCTTCACCCCACATATCAAATGGGCGAGAAGGCGCGTAGATG
TAGTCTTCCTCGGTCCCATCGGTAATCACGTGTGTGCCGATTTGCAAGACGAAAAGCCACGAGAATAAACC
GGGAGAGGGGATGGAAGTCCCCGAACAGCAACCAGCCCTTGCCCTCGTGGACATAACCTTTCACTTGCCAG
AACTCTAAGCGTCACCACGGTATACAAGCGCACGTAGAAGATTGTGGAAGTCGTGTTGGAGACTGTTGATT
TGGGCGGTGGAGGGGGGTATTTGAGAGCAATCCTTAACTGTTGGTCGGTTGCCGTTTTTGCCTGCTCCTAA
CAGCTCGCACCGACTCTAAAAAACCTATACGACCTGGCCGGCGTAACTTTGAGTGTCGTCAAACTCTGATAT
ATATATAGACACACGTATCCCAACAGTTGATAGTCGACAAACGCAAAACAGACGGACACTGAACCCCCCGC
GCTTCAAAACACCGACA

Hybrid promoter P32
S. cerevisiae ENO1 *S. cerevisiae ZWF1* *Y. lipolytica PFK1*

ATCGCGTTACCATATCGCCAAAAACTGATATACGCCGCGGAAACCAGGCAAACAATTGAAAAGAAAAATTT
TGAGGAACTCTCTGCATCGAAGCCGTCTAGAGTTACCACTAGTCAGATGCCGCGGGCACTTGAGCACCTCA
TGCACAGCAATAACACAACACAATGGTTAGTAGCAACCTGAATTCGGTCATTGATGCATGCATGTGCCGTG
AAGCGGGACAACCAGAAAAGTCGTCTATAAATGCCGGCACGTGCGATCATCGTGGCGGGGTTTTAAGAAA
AGGATAACCCCTGTGTTGGGAGCACCTGGTAAGTAAGGTGTAGTTTTGCACCCGTGTACATAAGCGTGAAA
TCACCACAAACTGTGTGTATCAAGTACATAGTGACATTTAATTAATAGCAAGAACAACAATAATAGTAGCGC
TACTGGAAGCACCACGTAATAGTGGAAAAGAACTGGAAAAACCGCTATAAGATGCATACTCCGGCGGTCTT
ACGCGGAGATACAAGCTTCCAACGGTGCTAAAAGCCCGGTTTCGGCTCGGCCGGAGGCGGTGGTTCAAAC
ACCTCAGATCAGCTCACTATCGGCTGAGACAATCCTTAACTGTTGGTCGGTTGCCGTTTTTGCCTGCTCCTAA
CAGCTCGCACCGACTATAAAAAACCTATACGACCTGGCCGGCGTAACTTTGAGTGTCGTCAAACTCTGATAT
ATATATAGACAGAGGTATCCCAACAGTTGATAGTCGACAAACGCAAAACAGACGGACACTGAACCCCCCGC
GCTTCAAAACACCGACA

Hybrid promoter P34
Y. lipolytica STL1 *K. marxianus MDH1* *Y. lipolytica GND2*

TAAGTAGGCTTTTGAGTTTCCGTCTTTTTCGCAAAGTGAAAAAAGGTGGGATAATCCGGGGTGCCGTACATT
TGGAGAAGGTGGTAATATAACCGAATAGTTGGCCAATATGAGCCGCGAAAATCGGAATCAAGTCGCCAAC
GTCTACTCATTTTATTCCAGATCTCAGATTCTTTCTAGCACATCTATAGGCCCTAATAACTGCGTCCCCTTCGA
GCTATAACATTTAAGGTGCAAAGAGAGGTTGGGAAGACAGCCTAAGGATTAGGGAGAGGTTTTGCTGCGT
GGGGGAACCGAGGGAGGGAATCCTCTGGAAGCCGGACCTTGCCCTTTTGACTAGGGTACTACTCTGGGCA
GGGTACTTACTAAACTGGGCCCAGACAGACAGAGGCCCAGACAGAGGCCCCAAAGAGAAGCCGCCAGGTT
CCCTGCCTAGGCCTTCCTCTGGTTGCCCCCCAGAGTACCCTCCGGAACGAAACGAAACGAAAACGCACATC
GTGATGCGCGCTATTATAATTGCGTCTTGCGAATTCCATACGCCATGCGCTTAGTTAGCACATGAAATCCTG

CCAATATTGACAATCCAACTTCCCAATTCCATTCCGATGACTAAAGTTTGAGCGTTTAATCCGCCAAATGACC
ACACTGTATATAAACTCGATTGCCAGCGAAATAGAGTTGCTTTACTAAGCACAAAGTCTGTTGAGTTGGCTG
AGACTTGGATTTATAAAAGGCTGCAGCGTCCCTCTCCAGACCTTTTCTGCAACTTGACATTTTCTTGTTAACG
ACACCATCACACA

Hybrid promoter P36
S. cerevisiae ZWF1 *K. phaffii PGK1* *Y. lipolytica ADH1*

CCCGCCCGCTTTCGCTAGAGTACGTCTTCGCGCCCGTCAAGCAGGCCGTCGAAAAGGATCTAGCCCCTGTTG
GGAGCACCTGGTAAGTAAGGTGTAGTTTTGCACCCGTGTACATAAGCGTGAAATCACCACAAACTGTGTGT
ATCAAGTACATAGTGACTTTTAAATAATAGCAAGAACAACAATAATAGTAGCGCTACTGGAAGCACCACGT
AATAGTGGAAAAGAACTGGAAAAACCGCTATAAGATGCATACTCCGGCGGTCTTACGCGGAGATACAAGC
TATGTTTATTTTTCGTCCAACCTAATTGTGGTTTCAAAGCGCTATCAGGTGGGGGGTAAGAGGAATGTGAGT
GGAAAGCGAAAATAACTGGCAGCTGGGGTCAGATCCCGTGATGCCACCTCTTGTGGTATTTTGAAACGCGT
GTTGCGATTGGCCGCGAGAACGGAAAGGAATATATTTACTGCCGATCGCATTTTGGCCTCAAATAAATCTTG
AGCTTTTGGACATAGATTATATGTTCTTTCTGCAATTGCACAATCGCAGACGGTGGAGATTGTCGGGTGACG
CACGTACACATACAGTATCGCCTGTACCACCCTACCCAGCACGTCAACTGCAGCTGTTCAACAGACGTCGAA
CCCCCACCATCTCAAAGATGACAGGAGACAAGGAGACAGACTTTCTATCGTTTTTGAACCCTATTATTTCTGC
CTCTTGCCTTATATAAAGGTCGGTCAGAGCGCCCTCTTATCATGGTCCCCACTTCCAATCCCTACTTCTACACC
AGCATCAAAA

Hybrid promoter P37
Y. lipolytica PGK1 *Y. lipolytica FBA1* *Y. lipolytica ENO2*

CAGACAGTGACGAGTCATACATTCTCCGTATAATATCGTGTATGTCCAGACGAAATGGTATGGATATGCAC
GGGGCGTAACTACTGTGCGAGTACTCGTGCATGTATCGTAGGTATTGTATGTTCGAGTACATACACATACG
ATACCAAACACTGCCCACTGTTCTGTCATGTTAGATCATGGCCAATCCACGTGACTTGCATGCAGGTTTGGC
ATTGAATATTCAGCGTGGCTACTACAAGTAGTACATACTGTATCAATACGATTGTACATACGGTACTCACCCT
TTGCTACATCACGTGTGTACAAGGGCGCACATGGCAGGCGCTGAGGTCGAGCAGGGTGGTGTGACTTGTT
ATAGCCTTTAGAGCTGCGAAAGCGCGTATGGATTTGGCTCATCAGGCCAGATTGAGGGTCTGTGGACACAT
GTCATGTTAGTGTACTTCAATCGCCCCCTGGATATAGCCCCGACAATAGGCCGTGGCCTCATTTTTTTGCCTT
CCGCACATTTCCATTTGAGATTTGTGCCATTGAGGGGGAGGTTATTGTGGCCATGCAGTCGGATTTGCCGTC
ACGGGACCGCAACATGCTTTTCATTGCAGTCCTTCAACTATCCATCTCACCTCCCCCAATGGCTTTTAACTATA
TAAAGACGAAAGCACCCCCCTTTGTACAGATGACTATTTGGGACCAATCCAATAGCGCAATTGGGTTTGCAT
CATGTATAAAAGGAGCAATCCCCCACTAGTTATAAAGTCACAAGTATCTCAGTATACCCGTCTAACCACACA
TTTATCACA

Hybrid promoter P38
O. polymorpha SDH1 *O. polymorpha PGI1* *Y. lipolytica ENO2*

CTCGCGACGGCGGACATTGGTGTCGCCATGGGCTCGGGGTCGGATCTGGCCTTGAGCTCGTGCGATTTTGT
CCTTTTGTCGCAGAAACAGCCACTCAAGCAGCTGCAAATCCTGCTCCAGCTGTCGCACAAAGTGATCAACCG
TGTCAAATTCAACTTTGGCTGGGCACTGGTCTACAATGTGATTGGAATCCCGATCGCTGCAGGGGTTATTTA
TCCGTACCATAACTCGCGTCTGAGTCCTACGTGGTCGAGTTTAGCCATGGCTTGTTCGAGCGTGAGTGTTTT
ATGCAGCAGTCTGGCACTCAGATGGGATAATCAGCAGTCTTTTTCGGAATTTGAAAATGTTTGGACGGACT
ACGAAAATAAGGAGTTTGATATTAATGAGAGTAAGTTTTTCAATGCAATGGAAGAGAAGAACGAAGCTGC
AGAAGAGCAAATAGACGCTTTGAAACAGCAGATCTTGAACATGGATGCATACACCAAACAGCTCTGGGAG
CAAGCAAAGTCGTCCTGGAAGTCCGGAAAATAGCCGCACAGCCCGGACGGTTTCTTGCGGAGAACAGTTG
CCGTCACGGGACCGCAACATGCTTTTCATTGCAGTCCTTCAACTATCCATCTCACCTCCCCCAATGGCTATAA

AAGTTCGAATGACGAAAGCACCCCCCTTTGTACAGATGACTATTTGGGACCAATCCAATAGCGCAATTGGG
TTTGCATCATGTATAAAAGGAGCAATCCCCCACTAGTTATAAAGTCACAAGTATCTCAGTATACCCGTCTAAC
CACACATTTATCACA

Hybrid promoter P43
Y. lipolytica PGK1 K. marxianus TAL1 K. lactis GPDH

CAGACAGTGACGAGTCATACATTCTCCGTATAATATCGTGTATGTCCAGACGATAGTCGTACTCGTACTCGT
TACTGTAACTACTGTGCGAGTACTCGTGCATGTATCGTAGGTATTGTATGTTCGAGTACATACACATACGAT
ACCAAACACTGCCCACTGTTCTGTCATGTTAGATCATGGCGGGGCCACGTGACTTGCATGCAGGTTTGGCAT
TGAATATTCAGCGTGGCTACTACAAGTAGTACATACTGTATCAATACGATTGTATTTCCAATCCAGATTCTAC
GGGACATAACAACAGCAACACCCACGATCCCACGCATATTTATTTCTTCTGTATCAGCTAGACTTTTTGTTCT
CCTCACTGCTTGCTAGAGGCAGACAGACGTGTACAGCATTTGTAATCATTTATGACCTACTAGAAACAAAAA
ATAAAAAAAGGCATCAAAAAGCACACTACATTACATACACATGTATATACTACGAGCCAAGCCAAGGCAAA
GTAGGCCAGGGTAAGGTATGGTAAGGTAAGCGGCGGTATGCCCCACAGCCCTTAGCAATTCCAGAAGTAT
CTGCACATAATGTGGTTATGTTCCAATATAGGTACCACCTTTGTTCAAGATTTAGTTTTCTAATTGAATATAA
ATACAGAGGTTATTTCAACCTAATTGAGATTAAGGAGAGACTTATTTTACTATAGTATATATTTATATATAAT
TACTTATTGTTACTCCAATCCCCAAGTAGATTAGATTTAATCAATCACACACAAACATCAAAACAACAAATTA
ACAAAA

Hybrid promoter P45
Y. lipolytica ENO2 Y. lipolytica GPDH Y. lipolytica ACO1 Y. lipolytica ENO2

TTCAGGCGGCTACTTGTATGTAGCATCCACGTTCATGTTTTGTGGATCAGATTAATGGTATGGATATGCACG
GTTGAAATGAATCGGCCGACGCTCGGTAGTCGGAAAGAGCCGGGACCGGCCGGCGAGCATAAACCGGAC
GCAGTAGGATGTCCTGCACGGGTCTTTTTGTGGGGTGTGGAGAGGGGGGTGCTTGGAGATGGAAGCCGG
TAGAACCGGGCTGCTTGGGGGGATTTGGGGCCGCTGGGCTCCAAAGAGGGGTAGGCATTTCGTTGGGGTT
ACGTAATCCGCACCGAAGTGACAACATGGACAATGTGACACGTAGATACACGCAGGAAGCAGCTGTCCAC
ACACATTTATCCCGAAAAATAGCCCGCATCACATGCACGACTCGTAAAAAGAAAAGAGCTGCGGGCCAAAG
GACCAATAAGTGCCGAGGAATGTTAAGCCAAAAGAACAACGACGATCGCCAGACAGGTTTAGTGGGAGCA
GCAGCAGCAGAGGCCGTGCAACGGCAGGAGAGAGAGGTCTGGCGAAAAGGAGGACAGGGGCCATGCAG
TCGGATTTGCCGTCACGGGACCGCAACATGCTTTTCATTGCAGTCCTTCAACTATCCATCTCACCTCCCCCAA
TGGCTTTTAACTTTCGAATGACGAAAGCACCCCCCTTTGTACAGATGACTATTTGGGACCAATCCAATAGCG
CAATTGGGTTTGCATCATGTATAAAAGGAGCAATCCCCCACTAGTTATAAAGTCACAAGTATCTCAGTATAC
CCGTCTAACCACACATTTATCACA

Hybrid promoter P46
K. phaffii CYC1 Y. lipolytica GPDH Y. lipolytica ZWF1 K. phaffii CYC1

TCAATAAGGTTTTTGACAGTCTAAAACTTTCTATAACGAGTATGCCTTACATCTTGAAACGATGGCTTGGACC
AGACCGAGGAGTAGCCGACGCTCGGTAGTCGGAAAGAGCCGGGACCGGCCGGCGAGCATAAACCGGACG
CAGTAGGATGTCCTGCACGGGTCTTTTTGTGGGGTGTGGAGAGGGGGGTGCTTGGAGATGGAAGCCGGTA
GAACCGGGCTGCTTGGGGGGATTTGGGGCCGCTGGGCTCCAAAGAGGGGTAGGCATTTCGTTGGGGTTAC
GTAATTGCGGCATTTGGGTCCTGCGCGCATGTCCCATTGGTCAGAATTAGTCCGGATAGGAGACTTATCAG
CCAATCACAGCCTGCGTACCACCTGTAGGTGCGATAGTTCACCAGTCACGCTGGTGGGCCATTCTCTTAGCA
CATTTCCCCCCTCCCAAGTCCCCTCAACCCCCAATGTAACCCTCAACCTCCCACAGCTCAGTAGCACACGTGC
AACTAGTTAGTAACAACCCCCCTCCGTCCAGCTTCTCTTTCACACTGCTTAGAGTTCGGGTGCCAACGTTGCC
AAACTACCCTGACGTGCTAACAAAGGAAAGCGTTTTAACTGTAAGCTATCAGATTACATAAGACCTGTCCGC
GATCGCCCGATCTCAGAGTTCCCACAAGAGCCAATAAAATCGCGCGAATTTGAAATCGGAAAATGTAGGAA

CTCAATTGTCCTATATAAATAATTCCCTTCCCGTGGAAACTGAAAAAAAAGTTCACCCCGACTTATTTATTCA
ATTCAACACAAAA

Hybrid promoter P47
S. cerevisiae PGK1 *Y. lipolytica GPDH* *S. cerevisiae PGK1* *K. phaffii ADH1*

GCAAGAATTACTCGTGAGTAAGGAAAGAGTGAGGAACTATCGCATACCTGCATTTAAAGATGCCGATTTGG
GCGCGAAATGAATCGGCCGACGCTCGGTAGTCGGAAAGAGCCGGGACCGGCCGGCGAGCATAAACCGGA
CGCAGTAGGATGTCCTGCACGGGTCTTTTTGTGGGGTGTGGAGAGGGGGGTGCTTGGAGATGGAAGCCG
GTAGAACCGGGCTGCTTGGGGGGATTTGGGGCCGCTGGGCTCCAAAGAGGGGTAGGCATTTCGTTGGGG
TTACGTAATTGCGGCATTTGGGTCCTGCGCGCATGTCCCATTGGTCAGAATTAGTCCGGATAGGAGACTTAT
CAGCCAATGGCGGGAAAGGGTTTAGTACCACATGCTATGATGCCCACTGTGATCTCCAGAGCAAAGTTCGT
TCGATCGTACTGTTACTCTCTCTCTTTCAAACAGAATTGTCCGAATCGTGTGACAACAACAGCCTGTTCTCAC
ACACTCTTTTCTTCTAACCAAGGGGGTGGTTTAGTTTAGTAGAACCTCGTGAAACTTACATTTACATATATAT
AAACTTGCATAAATTGGTCAATGCAAGAAATACATATTTTGCCTATTGTAGACGTCAACCCGCATCTGGTGC
GAATATAGCGCACCCCCAATGATCACACCAACAATTGGTCCACCCCTCCCCAATCTCTAATATTCACAATTCA
CCTCACTATAAATACCCCTGTCCTGCTCCCAAATTCTTTTTTCCTTCTTCCATCAGCTACTAGCTTTTATCTTATT
TACTTTACGAAA

Hybrid promoter P50
Y. lipolytica MDH1 *K. lactis PFK2* *K. phaffii AOX1*

GTTCCGGCTCCGATTTCTCGGCCAAGTGCCGACTCCACAAGACCGAGGAAGGTAAACTCTCTGTTCACGTG
GGGCTGAAGAAAGGCCAGCTCACGGTGGTGGAGTTGCAATGGAGGCGGCAGCTGAATAGACCTTGCAAG
TAAGGGGTACCTGACGGGAATGTGGGAATATGGGACACAAATTGGGGGGCAAACTTGCAACATGCGTCCA
GTGTACCCCGGATAACACCATTAGTGTGGCTAATAGCGAGGATGGGGACTTGGAGGGATGGGTTCGCAGG
GATGGACTCGGAGGGATGGACGCGAATGGCGTGGAGGGCTCGGATGGCGCGGAGGGTTCGGAGATGGG
TCCAGGGCCAAAAATCCGGTTTAAAAAGGTGGATATGGTCGATTGTAGTGGAAGCTCGTGATCCTACATGC
TCACGAAACCCATCATCATGCAATCCACATTAAAGGAAGGGAAAAGGATATTGAACTTTTGACTATTTAGTA
TAAATGAAAACTACTTTGTAAGCTTGGACAGAGGAATAATTTCTGATTCGTGCTTCTGCTTCTACTGACTTGC
AAACCAGTAAACAAGTTACTGCATTAATCAGTAATAATCTGCGTATAGCGAAACTTGTAACCCTACTTGACA
GCAATATATAAACAGAAGGAAGCTGCCCTGTCTTAAACCTTTTTTTTATCATCATTATTAGCTTACTTTCATAA
TTGCGACTGGTTCCAATTGACAAGCTTTTGATTTTAACGACTTTTAACGACAACTTGAGAAGATCAAAAAAC
AACTAATTATTCGAAACA

Hybrid promoter P55
Y. lipolytica ENO2 *O. polymorpha PYK1* *S. cerevisiae PGK1*

ATCACGCTACACTTAGCTACAGAATAAAGCTCGGTAGCGCCAACAGCGTTGACAAATAGCTCAAGGGCGTG
GAGCACAGGGTTTAGGAGGTTTTAATGGGCGAGAAGGCGCGTAGATGTAGTCTTCCTCGGTCCCATCGGTA
ATCACGTGTGTGCCGATTTGCAAGACGAAAAGCCACGAGAATGGGGCGGGAGAGGGGATGGAAGTCCCC
GAACAGCAACCAGCCCTTGCCCTCGTGGACATAACCTTTCACTTGCCAGAACTCTAAGCGTCACCACGGTAT
ACAAGCGCACGTAGAAGATTGTGGAAGTTGAGCTCGTGTTGGAATTTCAGTATAAGCGATTCCGTCAGACT
CACTGCATCTGGGTCAGAGGGAGGCTTTAGGACAGGAGCCATCTGTACAGAGGCTACGGAGTGTGGTGGC
GGGTTCATGGGTGGCTCAAGCGGTCTAAAAGCAAAGGTGCGCGGCCGTACGTTATTGTTTGTGTGGGTAC
GCGATAAATAAAAAATCAGACAATCGGCTATGGGGGTGACATAAGCGATGAGCAAACTCTATAGCCTCGG
CAAACCTGGGCGTGCACACACTCTTTTCTTCTAACCAAGGGGGTGGTTTAGTTTAGTAGAACCTCGTGAAAC
TTACATTTACATATATATAAACTTGCATAAATTGGTCAATGCAAGAAATACATATTTGGTCTTTTCTAATTCGT

AGTTTTTCAAGTTCTTAGATGCTTTCTTTTTCTCTTTTTTACAGATCATCAAGGAAGTAATTATCTACTTTTTAC
AACAAATATAAAACA

Hybrid promoter P62
S. cerevisiae TEF1 *K. lactis TEF1* *Y. lipolytica TEF1*

AGGTCTTTAGTCAGAGGCAGGAACAGCCGTCAAGGGGGCATAAGACTACGGTCATCCCCATCTGCCTCTTC
GTCCAGCCTTGCCAACAGGGAGTTCTTCAGAGACATGGAGGCTCAAAACGAAATTATTGACAGCCTAGACA
TCAATAGTCATACAACAGAAAGCGACCACCCAACTTTGGCTGGGGGTAGCGTATAAACAATGCATACTTTG
TACGTTCAAAATACAATGCAGTAGATATATTTATGCATATTACATATAATACATATCACATAGGAATGGACA
GACAACTATACCAGCATGGATCTCTTGTATCGGTTCTTTTCTCCCGCTCTCTCGCAATAACAATGAACACTGG
GTCAATCATAGCCTACACAGGTGAACAGAGTAGCGTTTATACAGGGTTTATACGGTGATTCCTACGGCAAA
AATTTTTCATTTCTAAAAAGAAAAAGAAAAATTTTTCTTTCCAACGCTAGAAGGAAAAGAAAAATCTAATTA
AATTGATTTGGTGATTTTCTGAGAGTTCCCACACTTGCCGTTAAGGGCGTAGGGTACTGCAGTCTGGAATCT
ACGCTTGTTCAGACTTTGTACTAGTTTCTTTGTCTGGCCATCCGGGTAACCCATGCCGGACGCTATATAAGCT
ACTGAAAATTTTTTTGCTTTGTGGTTGGGACTTTAGCCAAGGGTATAAAAGACCACCGTCCCCGAATTACCTT
TCCTCTTCTTTTCTCTCTCTCCTTGTCAACTCACACCCGAAATCGTTAAGCATTTCCTTCTGAGTATAAGAATC
ATTCAAA

Hybrid promoter P67
Y. lipolytica TEF1 *Y. lipolytica TEF1* *C. hispaniensis TEF1* *Y. lipolytica TEF1*

TTCGCTCCCACACTACACCGTAATACCACGTCACTCTCATTGCAGGTTACCCTGCCCGTAGTCGCTCGATCCA
CCTCCTCCTTCTCTCGTGTGTGCAGCAAAGAGGCAGAGATGGAGCCCGTATGGTGAATTAAAACCGTGCGA
ATTTTCAAAATAAACTTTGGCAAAGAGGCTGCAAAGGAGGGGGGGTGAGGGCGTCTGGAAGTCGACCA
GACAGCGGGTTGGCGGCGTATTTGTGTCCCAAAAAACAGCCCCAATTGCCCCAAGTCCACCACCACTGGAC
ACTTGATTTACAAGTGCGGTGGTATGGATAAGCGAACCATTGAAAAGTTTGAGAAGGAAGCCGATGAGCTT
GGAAAGGGTTCTTTCAAGTACGCTTGGGTTCTTGACAAGTTGAAGGCTGAGCGAGAGCGAGGTATCACCAT
TGATATTGCTCTCTGGAAGTTCGACACGCCCAAGTACTACGTTACCATTATTGATGCTCCCGGTCACCGAGA
TTTCATCAAGAATATGATTACCGGTACTTCCCCACACTTGCCGTTAAGGGCGTAGGGTACTGCAGTCTGGAA
TCTACGCTTGTTCAGACTTTGTACTAGTTTCTTTGTCTGGCCATCCGGGTAACCCATGCCGGACGCTATATAA
GCTACTGAAAATTTTTTTGCTTTGTGGTTGGGACTTTAGCCAAGGGTATAAAAGACCACCGTCCCCGAATTA
CCTTTCCTCTTCTTTTCTCTCTCTCCTTGTCAACTCACACCCGAAATCGTTAAGCATTTCCTTCTGAGTATAAGA
ATCATTCAAA

Hybrid promoter P68
S. cerevisiae TEF1 *C. hispaniensis TEF1* *Y. lipolytica TEF1*

AGGTCTTTAGTCAGAGGCAGGAACAGCCGTCAAGGGGGCATAAGACTACGGTCATCCCCATCTGCCTCTTC
GTCCAGCCTTGCCAACAGGGAGTTCTTCAGAGACATGGAGGCTCAAAACGAAATTATTGACAGCCTAGACA
TCAATAGTCATACAACAGAAAGCGACCACCCAACTTTGGCTGGGGGTAGCGTATAAACAATGCATACTTTG
TACGTTCAAAATACAATGCAGTAGATATATTTATGCATATTACATATAATACATATCACATAGGAAGCAACA
CTTGATTTACAAGTGCGGTGGTATGGATAAGCGAACCATTGAAAAGTTTGAGAAGGAAGCCGATGAGCTT
GGAAAGGGTTCTTTCAAGTACGCTTGGGTTCTTGACAAGTTGAAGGCTGAGCGAGAGCGAGGTATCACCAT
TGATATTGCTCTCTGGAAGTTCGAGAGGCCCAAGTACTACGTTACCATTATTGATGCTCCCGGTCACCGAGA
TTTCATCAAGAATATGATTACCGGTACTTCCCCACACTTGCCGTTAAGGGCGTAGGGTACTGCAGTCTGGAA
TCTACGCTTGTTCAGACTTTGTACTAGTTTCTTTGTCTGGCCATCCGGGTAACCCATGCCGGACGCTATATAA
GCTACTGAAAATTTTTTTGCTTTGTGGTTGGGACTTTAGCCAAGGGTATAAAAGACCACCGTCCCCGAATTA

CCTTTCCTCTTCTTTTCTCTCTCTCCTTGTCAACTCACACCCGAAATCGTTAAGCATTTCCTTCTGAGTATAAGA
ATCATTCAAA

Hybrid promoter P73
S. cerevisiae TEF1 *K. lactis TEF1* *K. phaffii AOX1*

AGGTCTTTAGTCAGAGGCAGGAACAGCCGTCAAGGGGGCATAAGACTACGGTCATCCCCATCTGCCTCTTC
GTCCAGCCTTGCCAACAGGGAGTTCTTCAGAGACATGGAGGCTCAAAACGAAATTATTGACAGCCTAGACA
TCAATAGTCATACAACAGAAAGCGACCACCCAACTTTGGCTGGGGGTAGCGTATAAACAATGCATACTTTG
TACGTTCAAAATACAATGCAGTAGATATATTTATGCATATTACATATAATACATATCACATAGGAAGCAACA
GACAACTATACCAGCATGGATCTCTTGTATCGGTTCTTTTCTCCCGCTCTCTCGCAATAACAATGAACACTGG
GTCAATCATAGCCTACACAGGTGAACAGAGTAGCGTTTATACAGGGTTTATACGGTGATTCCTACGGCAAA
AATTTTTCATTTCTAAAAGAAAAAAGAAAAATTTTTCTTTCCAACGCTAGAAGGAAAAGAAAAATCTAATTA
AATTGATTTGGTGATTTTCTGAGAGTTCCCTTTTTCATATATCGAATTTTGAATATAAAAGGAGATCGAAAAA
ATTTTTCTATTCAATCTGTTTTCTGGTTTTATTTGATAGTTTTTTCTAACCCCTACTTGACAGCAATATATAAAC
AGAAGGAAGCTGCCCTGTCTTAAACCTTTTTTTTATCATCATTATTAGCTTACTTTCATAATTGCGACTGGTTC
CAATTGACAAGCTTTTGATTTTAACGACTTTTAACGACAACTTGAGAAGATCAAAAAACAACTAATTATTCG
AAACA

Hybrid promoter P78
S. cerevisiae TEF1 *K. phaffii TEF1* *S. cerevisiae HXT7*

TGAGGTCTTTAGTCAGAGGCAGGAACAGCCGTCAAGGGGGCATAAGACTACGGTCATCCCCATCTGCCTCT
TCGTCCAGCCTTGCCAACAGGGAGTTCTTCAGAGACATGGAGGCTCAAAACGAAATTATTGACAGCCTAGA
CATCAATAGTCATACAACAGAAAGCGACCGCCCAACTTTGGCGGGGAATAGCGTATAAACAATGCATACTT
TGTACGTTCAAAATACAATGCAGTAGATATATTTATGCATATTACATATAATACATATCACATAGGAAGCAA
CAGGCGCGTTGGCCTCCTTCACCTTTTAACCATCTTGCCCATTCCAACTCGTGTCAGATTGCGTATCAAGTGA
AAAAGAAAAAATTTTAAATCTTTAACCCAATCAGGTAATAACTGTCGCCTCTTTTATCTGCCGCACTGCATGA
GGTGTCCCCTTAGTGGAAAAGAATACTGAGCCAACCCTGGAGGACAGCAAGGGAAAAATACCTACAACTT
GCTTCATAATGGTCGTAAAAACAATCCTTGTCGGATATAAGTGTTGTAGACTGTCCCTTATCCTCTGCGATGT
TCTTCCTCTCAAAGTTTGCGATTTCTCTCTATCAGAATTGCCATCAAGAGAAAAAAAAGTATAAATAGACACC
ATATATGCCAATACTTCACAATGTTCGAATCTATTCTTCATTTGCAGCTATTGTAAAATAATAAAACATCAAG
AACAAACAAGCTCAACTTGTCTTTTCTAAGAACAAAGAATAAACACAAAAACAAAAAGTTTTTTTAATTTTAA
TCAAAAA

Hybrid promoter P88
Y. lipolytica TEF1 *K. marxianus TEF1* *Y. lipolytica TEF1*

GGTATTTTCACAATTGCACCCCAGCCAGACCGATAGCCGGCCGCAATCCGCCACCCACAACCGTCTACCTCC
CACAGAACCCCGTCACTTCCACCCTTTTCCACCAGATCATATGTCCCAACTTGCCAAATTAAAACCGTGCGAA
TTTTCAAAATAAACTTTGGCAAAGAGGCTGCAAAGGAGGGGGGGGTGAGGGCGTCTGGAAGTCGACCACA
CAGCGGGTTGGCGGCGTATTTGTGTCCCAAAAAACAGCCCCAATTGCCCCAATTGACCCCAAATTGACCCA
GTAGCGGGCCAGCGTATCCCAGCCTAGTGTATCCCAGCCTAGCCTAGCCTAGGCCAAACCTAGCCCTCTCTA
GCCTAGCGCCCAGCAGAAACACCGATGAAGCAAAGAAGTAACAGCAGGAAAGAAAAACAAACACAACAA
AAAAAAACAAGCAGCATAGCATCAACAGAAATTTCTAAAGAGAACCAAATTCACCCCAGAAACAACCGCAC
AAATACGACATCCATCCACCTTTCTTTTATCCCACACTTGCCGTTAAGGGCGTAGGGTACTGCAGTCTGGAAT
CTACGCTTGTTCAGACTTTGTACTAGTTTCTTTGTCTGGCCATCCGGGTAACCCATGCCGGACGCTATATAAG
CTACTGAAAATTTTTTTGCTTTGTGGTTGGGACTTTAGCCAAGGGTATAAAAGACCACCGTCCCCGAATTAC

CTTTCCTCTTCTTTTCTCTCTCTCCTTGTCAACTCACACCCGAAATCGTTAAGCATTTCCTTCTGAGTATAAGAA
TCATTCAAA

Hybrid promoter P89

S. cerevisiae TEF1 *K. marxianus TEF1* *Y. lipolytica TEF1*

AGGTCTTTAGTCAGAGGCAGGAACAGCCGTCAAGGGGGCATAAGACTACGGTCATCCCCATCTGCCTCTTC
GTCCAGCCTTGCCAACAGGGAGTTCTTCAGAGACATGGAGGCTCAAAACGAAATTATTGACAGCCTAGACA
TCAATAGTCATACAACAGAAAGCGACCACCCAACTTTGGCTGGGGGTAGCGTATAAACAATGCATACTTTG
TACGTTCAAAATACAATGCAGTAGATATATTTATGCATATTACATATAATACATATCACATAGGAAGCAACA
GGCGCCATCCCAGCGTATCCCAGCCTAGTGTATCCCAGCCTAGCCTAGCCTAGGCCAAACCTAGCCCTCTCT
AGCCTAGCGCCCAGCAGAAACACCGATGAAGCAAAGAAGTAACAGCAGGAAAGAAAAACAAACACAACA
AAAAAAAACAAGCAGCATAGCATCAACAGAAATTTCTAAAGAGAACCAAATTCACCCCAGAAACAACCGCA
CAAATACGACATCCATCCACCTTTCTTTTATCCCACACTTGCCGTTAAGGGCGTAGGGTACTGCAGTCTGGA
ATCTACGCTTGTTCAGACTTTGTACTAGTTTCTTTGTCTGGCCATCCGGGTAACCCATGCCGGACGCTATATA
AGCTACTGAAAATTTTTTTGCTTTGTGGTTGGGACTTTAGCCAAGGGTATAAAAGACCACCGTCCCCGAATT
ACCTTTCCTCTTCTTTTCTCTCTCTCCTTGTCAACTCACACCCGAAATCGTTAAGCATTTCCTTCTGAGTATAAG
AATCATTCAAA

Hybrid promoter P92

O. polymorpha ZWF1 *Y. lipolytica PFK1* *O. polymorpha TAL1*

GTTGAGCACGACGCGACCCCGATCTCGCACATCTATGAGCAGTATGCGCCCCGGGACTGGCCCGATGCGGA
GGCGGCATGGCTCAACTGCGAAGGCTCGTCGTCAGGAGGGTATGCGCTCCAGTATTTATTGCACTTGCAAA
AAAAGCACGAAAACACAGAGATCAGGACGATAAACGGCAGAAGGGGGGACATTTACGAGCTGCGGCTTG
TGAGCGGGTCCGAGGTGTCTACTTGGTCCTGGTACTACACTCGTACCGGTACCTTTTGATCCGATGGTTACT
TTTTATGTTCTATTTTACATTAGCGTGGAAATAGACCATGCCATCTTTGGCACCCCGGAAAAACTTGATCCAA
TAGAGTTGTTGGGTGGAGCTAGTGACTGGCGGCAATTGGAGAGCTTCTAGAAGACGAACCAGGAGCCCGA
TAGGAACTCCGTTGGCGTGAGTCGGCCCCCCAGTAGCAATTCGAATCACGTGACGTGGAGTTTTCCCTCGCC
CGCGTTCCTGGATTGTCCCAGCTGGCTCCTAAAATGGAAATGCATGTGGCAAAGTAATGGCTCCTAGGGTG
GACTTCAAGGATACACTCACATTGATTGGTTGACAAGGTTACGTAAACTTGTCTCCGAAGACGAATTTGTTT
ACGAAGATAGCATTTATTTGTAGGTTATTGATTAGGTAAGATTTATTCAACGCAAACTGGGTGATGAATATA
AATAAGCTCGGGTATTCCTCCGCAACCAGAACTTGAATTATATACGTTTCGATTGATTTCCATCAAATTATAA
ATTCAACAATTGCA

Hybrid promoter P94

Y. lipolytica GPDH *O. polymorpha PGI1* *O. polymorpha TAL1*

GTAGGTTGGGTTGGGTGGGAGCACCCCTCCACAGAGTAGAGTCAAACAGCAGCAGCAACATGATAGTTGG
GGGTGTGCGTGTTAAAGGAAAAAAAAAGAAGCTTGGGTTATATTCCCGCTCTATTTAGAGGTTGCGGGATA
GACGCCGACGGAGGGCAATGGCGCCATGGAACCTTGCGGATATGGGGACGCCGCGGCGGACTGCGTCCG
AACCAGCTCCAGCAGCGTTTTTTCCGGGCCATTGAGCCGACTGCGACCCCGCCAACGTGTCTTGGCCCACGC
ACTCATGTCATGTTGGTGCGGCTTGCACGGGATAATCAGCAGTCTTTTTCGGAATTTGAAAATGTTTGGACG
GACTACGAAAATAAGGAGTTTGATATTAATGAGAGTAAGTTTTTCAATGCAATGGAAGAGAAGAACGAAG
CTGCAGAAGAGCAAATAGACGCTTTGAAACAGCAGATCTTGAACATGGATGCATACACCAAACAGCTCTGG
GAGCAAGCAAAGTCGTCCTGGAAGCTGGCTCCTAAAATGGAAATGCATGTGGCAAAGTAATGGCTCCTAG
GGTGGACTTCAAGGATACACTCACATTGATTGGTTGACAAGGTTACGTAAACTTGTCTCCGAAGACGAATTT
GTTTACGAAGATAGCATTTATTTGTAGGTTATTGATTAGGTAAGATTTATTCAACGCAAACTGGGTGATGAA

TATAAATAAGCTCGGGTATTCCTCCGCAACCAGAACTTGAATTATATACGTTTCGATTGATTTCCATCAAATT
ATAAATTCAACAATTGCA

Hybrid promoter P95

S. cerevisiae TEF1 *Y. lipolytica ZWF1* *K. phaffii PYK1*

TATTTTCACAATTGCACCCCAGCCAGACCGATAGCCGGCCGCAATCCGCCACCCACAACCGTCTACCTCCCA
CAGAACCCCGTCACTTCCACCCTTTTCCACCAGATCATATGTCCCAACTTGCCAAATTAAAACCGTGCGAATT
TTCAAAATAAACTTTGGCAAAGAGGATGATAAGGAGGGGGGGTGAGGGCGTCTGGAAGTCGACCACAC
ACGGGGTTGGCGGCGTATTTGTGTCCCAAAAAGCAGCCCCAATTGCCCCAATTGACCCCAAAGCCCTACGT
CTGGACCGCTACATTATTTCATAGCACAGCAACAGAACCGAGTTTAATGGTTTGAAACCTAGGTGGAAGAG
GGGCGGGCGAGGTATCGTACTGTGGGTGCGATAGTTCACCAGTCACGCTGGTGGGCCATTCTCTTAGCACA
TTTCCCCCCTCCCAAGTCCCCTCAACCCCCAATGTAACCCTCAACCTCCCACAGCTCAGTAGCACACGTGCAA
CTAGTTAGTAACAACCCCCCACCGTCCAGCTTCCGTACGTTTTTCACATTCAAGGATGAGGGTTTTCCACGAG
TGAACTATTACTCCGGACCCCCACCATCATTTGCGGAATGAAACCTTTTGTGCTGAGATTATATAAGGCGTG
GGGACGGACGCTTCTTAACCGTTCCCCTAGAATGTCGTCCCCTGATCAAAATTTAATGGCATCCAACTTTGCT
GTAATAGGTATATATAACCTAGCAGGCGACCGTTCATGTACAGTAAATTGTTTTAGACTTTTTTTTAACTGAA
ATCAATCCA

Hybrid promoter P96

K. phaffii TPI1 *Y. lipolytica TPI1* *Y. lipolytica GND2*

CGAAATATACCACATTGCCAGTTTATACAGATGGTTAAGGGTGAAAATCAACGTTACACCTTGACGACCCCA
TTATTACGATGGCGTGAAGGAGATGAAGACCGGGTAGAAGAAATAAGAAAAGCGGTACAGTTTAGGTCCG
GAGATCTAGGGAAGGAGGCCTTAGCTTATATTGTAGCTGCTGGGGGAGAGGCAGCTGCTGGAAGATCTGA
AGGCCCTATCACGTATGATGATGGTGATGACCATTAGAGAACGCCCAGAGATTGATAGCCAGGTGACTCAG
AAAGTGCATGTTGGAAATGAGCCACAGACCAAGACAAGATATGACAAAATTGCACTATTCGATGCAGAATT
CGACGGTGTTTCCATTGGTGTTATGACATTCATCTGCATTCATACAAAAAAGTCTTGGTAGTGGTACTTTTGC
GTTATTACCTCCGATATCTACGCACCCCCCAACCCCCCTGCTACAGTAAAGAGTGTGAGTCTACTGTACATGC
TTACTAACTCTCTCTCCAAGACGGCACATCTGAGCACCAGCCACAACCAACAAACGACTTCTACTTGAGTGT
GGGGAGATTGCTCACAAGTCTGGACTGCCACGGATGACTAAAGTTTGAGCGTTTAATCCGCCAAATGACCA
CACTGTGCATAAACTCGATTGCCAGCGAAATAGAGTTGCTTTACTAAGCACAAAGTCTGTTGAGTTGGCTGA
GACTTGGATATATAAAACGCTGCAGCGTCCCTCTCCAGACCTTTTCTGCAACTTGACATTTTCTTGTTAACGA
CACCATCACACA

Hybrid promoter P97

K. phaffii PMA1 *K. phaffii PGK1* *Y. lipolytica FBA1*

AGGAAACCTCGATGATTCTCCCGTTCTTCCATGGGCGGGTATCGCAAAATGAGGAATTTTTCAAATTTCTCT
ATTGTCAAGACTGTTTATTATCTAAGAAATAGCCCAATCCGAAGCTCAGTTTTGAAAAAATCACTTCCGCGTT
TCTTTTTTACAGCCCGATGAATATCCAAATTTGGAATATGGGGTACTCTATCGGGACTGCAGATAATATGAC
AACAACGCAGATTACATTTTAGGTAAGGCATAAATTCTACAGGCACGTGCGAGGCAAGCAATCTACTAATG
TTTATTTTTCGTCCAACCTAATTGTGGTTTCAAAGCGCTATCAGGTGGGGGGTAAGAGGAATGTGAGTGGA
AAGCGAAAATAACTGGCAGCTGGGGTCAGATCCCGTGATGCCACCTCTTGTGGTATTTTGAAACGCGTGTT
GCGATTGGCCGCGAGAACGGAAAGGAATATATTTACTGCCGATCGCATTTTGGCCTCAAATAAATCTTGAG
CTTTTGGACATAGGTCTGTGGACACATGTCATGTTAGTGTACTTCAATCGCCCCCTGGATATAGCCCCGACA
ATAGGCCGTGGCCTCATTTTTTTGCCTTCCGCACATTTCCATTGCTCGGTACCCACACCTTGCTTCTCCTGCAC
TTGCCAACCTTAATACTGGTTTACATTGACCAACATCTTACAAGCGGGGGGCTTGTCTAGGGTATATATAAA

CAGTGGCTCTCCCAATCGGTTGCCAGTCTCTTTTTTCCTTTCTTTCCCCACAGATTCGAAATCTAAACTACACA
TCACACA

Hybrid promoter P98
Y. lipolytica PGK1 *O. polymorpha CYC1* *O. polymorpha PSH1*

CAGACAGTGACGAGTCATACATTCTCCGTATAATATCGTGTATGTCCAGACGATAGTCGTACTCGTACTCGT
TACTGTAACTACTGTGCGAGTACTCGTGCATGTATCGTAGGTATTGTATGTTCGAGTACATACACATACGAT
ACCAAACACTGCCCACTGTTCTGTCATGTTAGATCATGGCGGGGCCACGTGACTTGCATGCAGGTTTGGCAT
TGAATATTCAGCGTGGCTACTACAAGTAGTACATACTGTATCAATACGATTGTACATACGGTACTGGGTTTC
GCAAACAACATGGTAAAATTACAAATGTGACTATTTTTCCACTTTTTATTTTGGTACACTGGCCTTTCTTTTTG
CCATCAGCGCAATTCCCGACACCCGTGCACCGCGACACCCAAAAGTTGTCATTGAAATTTTCTCGCCGTTTG
AGATCGCTTTTGGAAAAAAAGAAAAAAATTACCTGGAAGCTCGTAGAATCCAGGGAGCCGAGGAATAAAC
TGGGGGTGCACAGCCTTACACCTTGTTATATATCTCGAGCACACGATCAAAGTGCTACAAAACCAGTACGAC
CACATCAGCGCACAGCATATGCAAAATTTGGACCAGTATCTAAAGAAAATGGCCCGCTGGAGGGAAATTGT
ATAAAGATAGACAAATCGACTAAATTTCTAAAACACAAAATAATTTTTTATGATCCACATGGATGGAAGTGC
TCAAGTTATTGCAATCCTTGGGGGATGAGAACAAGAAGGACTTGATGCGACGCAGTGTCGAGGTCTTTACT
TGTACCATCA

Hybrid promoter P99
Y. lipolytica GPDH *O. polymorpha PFK2* *K. phaffii AOX1*

GTAGGTTGGGTTGGGTGGGAGCACCCCTCCACAGAGTAGAGTCAAACAGCAGCAGCAACATGATAGTTGG
GGGTGTGCGTGTTAAAGGAAAAAAAAAAGAAGCTTGGGTTATATTCCCGCTCTATTTAGAGGTTGCGGGATA
GACGCCGACGGAGGGCAATGGCGCCATGGAACCTTGCGGATATGGGGACGCCGCGGCGGACTGCGTCCG
AACCAGCTCCAGCAGCGTTTTTTCCGGGCCATTGAGCCGACTGCGACCCCGCCAACGTGTCTTGGCGCAACA
TTCGGGAGCAGCTGGAGCGTATGTGTGAGCGGATGGGGCTTTTGGACGCAAACCAGCCGGTTTTGGACCA
CGACCGACTGCTTGTGAACGTGCTGAAGAGCATTGTGGCTGGCTTTTTCGTCAATGCTGCGCAGCTGAGCC
GGTCCGGCGACTCGTACCGGTCGATGAAAAAGAACCAGGCGGTGTGGATGCATCCGTCGTCGGTGCTGTT
CGGCGTGAAGCCGCCGCCGAAGCTGGTGATTTGGATGATTATGCATTGTCTCCACATTGTATGCTTCCAAGA
TTCTGGTGGGAATACTGCTGATAGCCTAACGTTCATGATCAAAATTTAACTGTTCTAACCCCTACTTGACAGC
AATATATAAACAGAAGGAAGCTGCCCTGTCTTAAACCTTTTTTTTTATCATCATTATTAGCTTACTTTCATAATT
GCGACTGGTTCCAATTGACAAGCTTTTGATTTTAACGACTTTTAACGACAACTTGAGAAGATCAAAAAACAA
CTAATTATTCGAAACA

Hybrid promoter P101
Y. lipolytica PGK1 *Y. lipolytica ZWF1* *Y. lipolytica GND2*

CAGACAGTGACGAGTCATACATTCTCCGTATAATATCGTGTATGTCCAGACGATAGTCGTACTCGTACTCGT
TACTGTAACTACTGTGCGAGTACTCGTGCATGTATCGTAGGTATTGTATGTTCGAGTACATACACATACGAT
ACCAAACACTGCCCACTGTTCTGTCATGTTAGATCATGGCGGGGCCACGTGACTTGCATGCAGGTTTGGCAT
TGAATATTCAGCGTGGCTACTACAAGTAGTACATACTGTATCAATACGATTGTACATACGGTACTCACCCTTT
GCTACAGTATGTACATACAAGGGCGCAACAGAGCCGAGTTTAATGGTTTGAAACCTAGGTGGAAGAGGGG
CGGGCGAGGTATCGTACTGTGGGTGCGATAGTTCACCAGTCACGCTGGTGGGCCATTCTCTTAGCACATTT
CCCCCCTCCCAAGTCCCCTCAACCCCCAATGTAACCCTCAACCTCCCACAGCTCAGTAGCACACGTGCAACTA
GTTAGTAACAACCCCCCTCCGTCCAGCTTCTCTTTCACACTGCTTAGAGTTCGACTTCTACTTGAGTGTGGGG
AGATTGCTCACAAGTCTGGACTGCCACGGATGACTAAAGTTTGAGCGTTTAATCCGCCAAATGACCACACTG
TGCATAAACTCGATTGCCAGCGAAATAGAGTTGCTTTACTAAGCACAAAGTCTGTTGAGTTGGCTGAGACTT

GGATTTATAAAACGCTGCAGCGTCCCTCTCCAGACCTTTTCTGCAACTTGACATTTTCTTGTTAACGACACCA
TCACACA

Hybrid promoter P103

K. lactis ENO1 *Y. lipolytica ENO2* *Y. lipolytica GPDH*

GGGACAAAGAAGAATCTTCGTTCTTCTTTCTTGTTCTCAACTTCCCAGCTTCCGTGTGATTACCCTCCGGGAC
AACAGAAAAACTGGCATTCGGTATCCCGGGAATCTGCTGAGAAGGAAAGAAAACGAAAAAAAAATTGTAC
ATTTGTGTCACATTATGAATTACAGGAAGTCAGAAAACAGGGGGGACATGTCTCGCACATGCATGTCCATC
AGACGAGACATTATGAGACATGCACGCGTGTGAGACTTAGCTACAGAATAAAGCTCGGTAGCGCCAACAG
CGTTGACAAATAGCTCAAGGGCGTGGAGCACAGGGTTTAGGAGGTTTTAATGGGCGAGAAGGCGCGTAG
ATGTAGTCTTCCTCGGTCCCATCGGTAATCACGTGTGTGCCGATTTGCAAGACGAAAAGCCACGAGAATAA
ACCGGGAGAGGGGATGGAAGTCCCCGAACAGCAACCAGCCCTTGCCCTCGTGGACATAACCTTTCACTTGC
CAGAACTCTAAGCGTCACCACGGTATACAAGCGCACGTAGAAGATCCTCGAATTTCAGACCAGTCACGGCC
CCATTCGCCCGCGCAATGGCTCGCCAACGCCCGGTCTTTTGCACCACATCAGGTTACCCCAAGCCAAACCTT
TGTGTTAAAAAGCTTAACATATTATACCGAACGTAGGTTTGGGCGGGCTTGCTCCGTCTGTCCAAGGCAACA
TTTATATAAGGGTCTGCATCGCCGGCTCAATTGAATCTTTTTTCTTCTTCTCTTCTCTATATTCATTCTTGAATT
AAACACACATCAACA

11. SUPPLEMENTARY REFERENCES

1. Horton RM, Hunt HD, Ho SN, Pullen JK, Pease LR. Engineering hybrid genes without the use of restriction enzymes: gene splicing by overlap extension. *Gene* **77**, 61-68 (1989).
2. Hoof JB, Nodvig CS, Mortensen UH. Genome Editing: CRISPR-Cas9. *Methods in molecular biology* **1775**, 119-132 (2018).
3. Magnan C*, et al.* Sequence assembly of *Yarrowia lipolytica* strain W29/CLIB89 shows transposable element diversity. *PloS one* **11**, e0162363 (2016).
4. Fernandez-Canon JM, Penalva MA. Characterization of a fungal maleylacetoacetate isomerase gene and identification of its human homologue. *The Journal of biological chemistry* **273**, 329-337 (1998).
5. Carreira A, Ferreira LM, Loureiro V. Brown pigments produced by *Yarrowia lipolytica* result from extracellular accumulation of homogentisic acid. *Applied and environmental microbiology* **67**, 3463-3468 (2001).
6. Bassel J, Hambright P, Mortimer R, Bearden AJ. Mutant of the yeast *Saccharomycopsis lipolytica* that accumulates and excretes protorphyrin IX. *Journal of bacteriology* **123**, 118-122 (1975).
7. Barth G, Weber H. Genetic studies on the yeast *Saccharomycopsis lipolytica*. Inactivation and mutagenesis. *Zeitschrift fur allgemeine Mikrobiologie* **23**, 147-157 (1983).
8. Carreira A, Dillinger K, Eliskases-Lechner F, Loureiro V, Ginzinger W, Rohm H. Influence of selected factors on browning of Camembert cheese. *The Journal of dairy research* **69**, 281-292 (2002).
9. Carreira A, Paloma L, Loureiro V. Pigment producing yeasts involved in the brown surface discoloration of ewes' cheese. *International journal of food microbiology* **41**, 223-230 (1998).
10. Warner JR. The economics of ribosome biosynthesis in yeast. *Trends in biochemical sciences* **24**, 437-440 (1999).
11. Keren L*, et al.* Promoters maintain their relative activity levels under different growth conditions. *Molecular systems biology* **9**, 701 (2013).
