# Supplementary material for: A DNA assembly toolkit to unlock the CRISPR/Cas9 potential for metabolic engineering
Source: Commun Biol. 2023 Aug 18;6:858. doi: 10.1038/s42003-023-05202-5 (PMC10439232; doi:10.1038/s42003-023-05202-5)
Supplement: Supplementary file 3 — Description of Additional Supplementary Files [file 42003_2023_5202_MOESM3_ESM.pdf]

Description of Additional Supplementary Files

File name: Supplementary Data 1
Description: Source data for the graphs in this manuscript
