# Supplementary material for: A DNA assembly toolkit to unlock the CRISPR/Cas9 potential for metabolic engineering
Source: Commun Biol. 2023 Aug 18;6:858. doi: 10.1038/s42003-023-05202-5 (PMC10439232; doi:10.1038/s42003-023-05202-5)
Supplement: Supplementary file 6 — Reporting Summary [file 42003_2023_5202_MOESM6_ESM.pdf]

nature portfolio

Corresponding author(s): Tigran V. Yuzbashev, Rodrigo Ledesma-Amaro

Last updated by author(s): May 22, 2023

Reporting Summary

Nature Portfolio wishes to improve the reproducibility of the work that we publish. This form provides structure for consistency and transparency in reporting. For further information on Nature Portfolio policies, see our Editorial Policies and the Editorial Policy Checklist.

Statistics

For all statistical analyses, confirm that the following items are present in the figure legend, table legend, main text, or Methods section.

n/a	Confirmed	
☐	☒	The exact sample size (*n*) for each experimental group/condition, given as a discrete number and unit of measurement
☐	☒	A statement on whether measurements were taken from distinct samples or whether the same sample was measured repeatedly
☒	☐	The statistical test(s) used AND whether they are one- or two-sided<br>*Only common tests should be described solely by name; describe more complex techniques in the Methods section.*
☒	☐	A description of all covariates tested
☒	☐	A description of any assumptions or corrections, such as tests of normality and adjustment for multiple comparisons
☐	☒	A full description of the statistical parameters including central tendency (e.g. means) or other basic estimates (e.g. regression coefficient) AND variation (e.g. standard deviation) or associated estimates of uncertainty (e.g. confidence intervals)
☒	☐	For null hypothesis testing, the test statistic (e.g. *F*, *t*, *r*) with confidence intervals, effect sizes, degrees of freedom and *P* value noted<br>*Give P values as exact values whenever suitable.*
☒	☐	For Bayesian analysis, information on the choice of priors and Markov chain Monte Carlo settings
☒	☐	For hierarchical and complex designs, identification of the appropriate level for tests and full reporting of outcomes
☒	☐	Estimates of effect sizes (e.g. Cohen's *d*, Pearson's *r*), indicating how they were calculated

Our web collection on statistics for biologists contains articles on many of the points above.

Software and code

Policy information about availability of computer code

Data collection	Attune NxT Software v3.1 was used for data collection in flow cytometry experiments. CLARIOstar software v5.7 was used to collect data from plate reader.
Data analysis	MARS software v3.42 was used for analysing fluorescense and absorbance data from plate reader. The flow cytometry data were analysed using FlowJo X10.0.7r2 software (Ashland, OR).

For manuscripts utilizing custom algorithms or software that are central to the research but not yet described in published literature, software must be made available to editors and reviewers. We strongly encourage code deposition in a community repository (e.g. GitHub). See the Nature Portfolio guidelines for submitting code & software for further information.

Data

Policy information about availability of data

All manuscripts must include a data availability statement. This statement should provide the following information, where applicable:

- Accession codes, unique identifiers, or web links for publicly available datasets
- A description of any restrictions on data availability
- For clinical datasets or third party data, please ensure that the statement adheres to our policy

All plasmid sequences, including components of the toolkit and tested promoter constructions are provided as Supplementary GenBank files. Modified lambda prophage sequences of both EcoRed and EcoCre strains are provided in GenBank format. Strains EcoRed, EcoCre, W29Δura, W29Δku70ura+, W29ΔuraΔku70 are deposited in VKPM as B-14086, B-14087, Y-4971, Y-4972, and Y-4973, respectively. Plasmid components of the toolkit are available via the non-profit plasmid repository Addgene (##175597-175743). The revised sequence of HMG2 gene of Y. lipolytica W29 is available from GenBank under accession number MZ387986.

Field-specific reporting

Please select the one below that is the best fit for your research. If you are not sure, read the appropriate sections before making your selection.

☒ Life sciences ☐ Behavioural & social sciences ☐ Ecological, evolutionary & environmental sciences

For a reference copy of the document with all sections, see nature.com/documents/nr-reporting-summary-flat.pdf

Life sciences study design

All studies must disclose on these points even when the disclosure is negative.

Sample size	For GFP activity screening with different promoters and integration loci each construction was tested in duplicate with independent transformants. Efficiency of DNA assembly was assayed using at least eight transformants. Marker-free Cas9-driven deletions and overexpressions in yeast were assayed using 36 and 50 transformants, respectively.
Data exclusions	No data were excluded.
Replication	New methods that could be poorly reproducible were tested with multiple replicates, e.g. marker-free deletions or overexpressions were assayed using two or three different genomic loci, respectively.
Randomization	Selection of E. coli and yeast transformants was guided based on colony phenotype, such as their size, resistance to antibiotics or fluorescence. No randomization was required in this study.
Blinding	Blinding was not applied in this study.

Reporting for specific materials, systems and methods

We require information from authors about some types of materials, experimental systems and methods used in many studies. Here, indicate whether each material, system or method listed is relevant to your study. If you are not sure if a list item applies to your research, read the appropriate section before selecting a response.

Materials & experimental systems

n/a	Involved in the study	
☒	☐	Antibodies
☒	☐	Eukaryotic cell lines
☒	☐	Palaeontology and archaeology
☒	☐	Animals and other organisms
☒	☐	Human research participants
☒	☐	Clinical data
☒	☐	Dual use research of concern

Methods

n/a	Involved in the study	
☒	☐	ChIP-seq
☐	☒	Flow cytometry
☒	☐	MRI-based neuroimaging

Flow Cytometry

Plots

Confirm that:

☐ The axis labels state the marker and fluorochrome used (e.g. CD4-FITC).

☐ The axis scales are clearly visible. Include numbers along axes only for bottom left plot of group (a 'group' is an analysis of identical markers).

☐ All plots are contour plots with outliers or pseudocolor plots.

☐ A numerical value for number of cells or percentage (with statistics) is provided.

Methodology

Sample preparation	To measure promoter activities yeast cultures were grown in microplates with either YPG or YNBG media. At 9th or 15th hour, respectively, the microplates were transferred to flow cytometer.
Instrument	Flow cytometer Attune NxT (Thermo Fisher Scientific).
Software	Attune NxT Software v3.1 was used for data collection. The data were analysed using FlowJo X10.0.7r2 software (Ashland, OR).

Cell population abundance	Fluorescence data were collected from 10,000 cells for each sample. Yeast cell populations exhibiting GFP fluorescence were selected using SSC-H/FSC-H gating as described below. Plots showing a distribution of cell fraction versus fluorescence intensity for all 90 samples were not provided. Instead, a single characteristic value, the median, was calculated and shown in Tables S15 and Figure 2.
Gating strategy	Two population of cells were observed using SSC-H/FSC-H gating. One of those has never exhibit GFP fluorescence and suggested to be cell debris present in yest extract. A picture with gating strategy will be provided during second submission.

☐ Tick this box to confirm that a figure exemplifying the gating strategy is provided in the Supplementary Information.
