# Supplementary material for: A DNA assembly toolkit to unlock the CRISPR/Cas9 potential for metabolic engineering
Source: Commun Biol. 2023 Aug 18;6:858. doi: 10.1038/s42003-023-05202-5 (PMC10439232; doi:10.1038/s42003-023-05202-5)
Supplement: Supplementary file 5 — Supplementary GenBank files [file 42003_2023_5202_MOESM5_ESM.zip › data_set/YaliCraft Manual.pdf]

Imperial College London
BioMediCan Inc

YaliCraft
manual

- 2023 -

Table of Contents

1. ASSEMBLY OF DNA CONSTRUCTS 3
   1.1 Introduction 3
   1.2 (Lvl0 Module) Assembly of basic parts 5
   1.3 (Exp Module) Assembly of overexpression constructs 8
      1.3.1 Assembly of empty Lvl1 vector 10
      1.3.2 Assembly of empty Lvl2 vector 13
      1.3.3 Assembly of single TU on the Lvl1 14
      1.3.4 Assembly of several TUs on the Lvl2 16
   1.4 (Del Module) Assembly of gene disruption construct 17
   1.5 (Int Module) Exchange of homology arms 20
      1.5.1 Exchange of homology arms between Lvl1 and Lvl2 20
      1.5.2 Transfer of homology arms from pDel-series to Lvl1 and Lvl2 21
   1.6 (MEx Module) Excision of yeast selectable marker 22
   1.7 (Cas Module) Re-encoding Cas9-helper 23
   1.8 (Pro Module) Introduction of new promoters 25

2. YARROWIA ENGINEERING 26
   2.1 Functional characterization of standard integration loci 27
   2.2 Marker-free CRISPR/Cas9-mediated integration 29
   2.3 Marker-based integration 30
   2.4 Combining marker-based and Cas9-mediated integration 30
   2.5 Selectable marker recovery 31
   2.6 Promoter library screening 32

3. PROTOCOLS 34
   3.1 GG reaction using BsaI/BsmBI/BpiI/AarI with thermal inactivation 34
   3.2 GG reaction using AarI without thermal inactivation 34
   3.3 GG reaction using LguI with thermal inactivation 35
   3.4 pDelUK-RG plasmid isolation 35
   3.5 *EcoRed* competent culture preparation 36
   3.6 *EcoCre* competent culture preparation 36
   3.7 *EcoRed* and *EcoCre* electroporation 37
   3.8 *Y. lipolytica* transformation 37
   3.9 *Y. lipolytica* colony PCR 38

4. APPENDIX 39
   4.1 Media recipes 39
   4.2 List of plasmids 40
   4.3 List of strains 42
   4.4 List of primers 42

5. REFERENCES 44

1. ASSEMBLY OF DNA CONSTRUCTS

1.1 Introduction

YaliCraft is a multi-modular toolkit for constructing integrative vectors and Cas9-helper plasmids for metabolic engineering in *Y. lipolytica* (Fig. S1). Six modules (Lvl0, Pro, Exp, Del, Int, MEx) are intended for the assembly of integrative constructs that are targeted to the *Y. lipolytica* genome. The Cas Module is designed to assemble episomal Cas9-helpers. There is also variation in the order in which modules can be utilised, leading to different integrative constructs for both gene overexpression and inactivation or both. These two types of modifications were selected because they are the most common in metabolic engineering. Nevertheless, the toolkit can be expanded with new modules and easily adapted for tasks such as introducing point mutations, short insertions/deletions or for the substitution of promoters upstream of native genes.

Most of the modules, except the Exp Module, include single-step manipulation of the construct. Modules Lvl0, Pro, Exp, Del, and Int are based on Golden Gate (GG) reactions. Each GG step requires only two days to get constructs ready either for the next modification step or for yeast transformation (Table S1). Due to the addition of fluorescent reporters and use of alternating antibiotic resistance markers, it is possible to select transformants with high efficiency by phenotypic selection alone. Chloramphenicol (Cm), spectinomycin (Sp), ampicillin (Ap) and kanamycin (Km) resistance are reserved for Lvl0-, Lvl1-, Lvl2-, and pDel-series, respectively. Additionally, the essential advantage of GG is that it does not require sequence verification after each assembly step – DNA only needs to be sequenced once after initial cloning of a PCR amplified fragment. The sequences of all overhangs required for GG assembly in this toolkit are summarized in Table S2.

The Cas and MEx Modules are based on *EcoRed* and *EcoCre E. coli* strains respectively. Both strains contained the modified λ prophage for temperature-inducible expression of λ-Red genes (*gam, bet, exo*) or *cre* gene of phage P1, respectively. Application of *in vivo* recombination does not require any *in vitro* step, and therefore, significantly accelerates and simplifies the workflow.

As a result, all procedures in this toolkit are based on the application of uncut plasmids and do not require a laborious gel purification step. At the same time, we highly recommend carrying out restriction analysis for accurate qualitative and quantitative analysis of constructs obtained following each assembly step. It is important to mention that incorrect spectrophotometric estimation of DNA concentration is the most common cause of DNA assembly failures.

The *YaliCraft* system is capable of growth and can be extended with new modules. It can be modified for overexpression of more than 3 transcriptional units (TUs) at a single integration locus, supplied with alternative yeast selectable markers or expanded to include additional integration loci. The toolkit also contains a set of empty vectors with Zeta sequences as integration flanks, which are commonly used for random integration[1]. Random integration requires active non-homologous end-joining system, and therefore, can be preferentially applied on the strains with Ku70+ genetic background. Furthermore, the promoters, genes, terminators, yeast markers and homologous arms from this system are fully compatible with the current most widely used *Y. lipolytica* GG system[2, 3, 4].

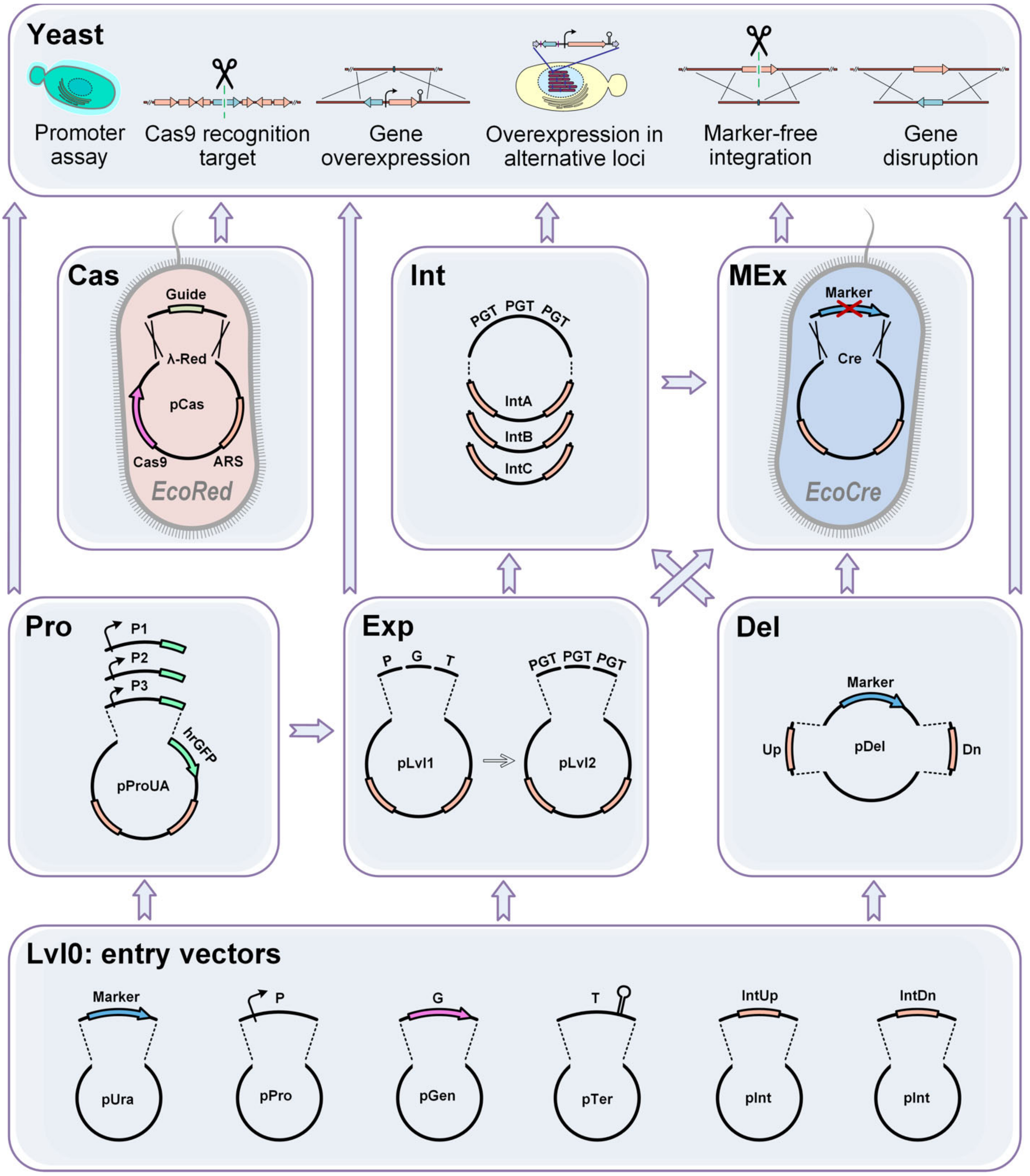

Fig. S1 Blueprint of the *YaliCraft* system. Seven modules are shown. Lvl0 Module: single parts in entry vectors. Exp Module: assembly of overexpression constructs. Pro Module: assembly and screening of new promoters. Del Module: assembly of disruption constructs. Int Module: changing integration loci by homology arms exchange. MEx Module: assembly of marker-free constructs by selectable marker excision. Cas Module: redirection of Cas9-helper to new genome loci. The arrows between modules indicate different orders in which they can be applied to enable variable genome engineering techniques as shown on the top panel - Yeast.

Table S1 Time required for single GG step (days)

Assembly method	Day 1	Day 2	Total time spent
**GG assembly**	● (opt) PCR and purification<br>● GG assembly or exchange<br>● *E. coli* transformation	● Culturing and miniprep<br>● Restriction analysis*<br>● Yeast transformation or next GG step	2 days

* - sequence verification is recommended only for DNA fragments amplified by PCR before assembling

Table S2 Overhangs used for different types of GG assemblies in *YaliCraft*

GG assembly	GGA enzyme	Bb*		IntUp		Marker		Pro (PGT)		Gen (PGT)		Ter (PGT)		IntDn		Bb
**Lvl0**	BsmBI		**TCGG**												**GACC**	
**preLvl**	BsaI		**GCCT**		**AGGT**		**ACGG**						**GAGT**		**TGCG**	
**Empty Lvl**	BpiI						**ACGG**						**GAGT**			
**Lvl1**	BsaI						**ACGG**		**AATG**		**TCTA**		**GAGT**			
**Lvl2.2**	BsmBI						**CTGA**		**CCAA**		**GATG**					
**Lvl2.3**	BsmBI						**CTGA**		**CCAA**		**GATG**		**GTTC**			
**Pro**	BpiI						**ACGG**		**AATG**							
**Del**	LguI		**CAG**		**CCA**								**TAA**		**GGA**	
**Int**	AarI				**AGGT**								**GAGT**			

* – Blue columns show the genetic elements in the order they appear in assembled Lvl1 or Lvl2 plasmids, while green columns show overhangs that are used in corresponding assembly types to combine these elements. Detailed description of each assembly type is provided in the following sections. Genetic elements are abbreviated as follows. Bb, plasmid backbone. IntUp and IntDn, upstream and downstream homologous arms, respectively. Marker, *Y. lipolytica* selectable marker. Pro, promoter. Gen, gene. Ter, terminator. PGT, transcription unit.

1.2 (Lvl0 Module) Assembly of basic parts

During the first step, genetic elements are cloned and stored separately in the Lvl0 Module. Each new element, whose sequence has been verified, can be used in subsequent assemblies without further sequencing. These genetic elements include *Y. lipolytica* parts such as promoters, genes, terminators, homologous arms, and selectable markers. The Lvl0 Module also contains *E. coli* parts like fluorescent reporters carrying overhangs for different levels and backbones with antibiotic resistance markers on the multicopy ColE1 replication origin for easy phenotypic selection (Section 4.2).

Since elements cloned in this module are used for GG assemblies, it is necessary to first remove recognition sites of certain restriction enzymes. This could be done during design by selecting appropriate regions of natural sequences or by introduction of specific codon substitutions. To remove a small number of undesirable sites, site-directed mutagenesis can be achieved using overlap extension PCR[5]. The full list of restriction sites that need to be excluded is provided in Table S3. Depending on the exact element the list can be shortened as specified in the table.

Table S3 Restriction sites that need to be excluded depending on the genetic element

Enzyme (isoschizomer)	Recognition site	Reverse complement [a]	Module [b]	IntUp and IntDn for Exp Module, and Marker [3]	Promoter Gene Terminator [c]	IntUp and IntDn for Del Module [c]
**BpiI** (BbsI)	GAAGAC	GTCTTC	Exp/Pro	-	+	+
**BsaI** (Eco31I)	GGTCTC	GAGACC	Exp	-	-	+
**BsmBI** (Esp3I)	CGTCTC	GAGACG	Exp/Lvl0	-	-	+
**AarI**	CACCTGC	GCAGGTG	Int	-	-	-
**LguI** (SapI)	GCTCTTC	GAAGAGC	Del	+	+	-
**NotI**	GCGGCCGC		Yali	-	-	+/-
**SgrDI**	CGTCGACG		Yali	-	-	+/-
**MssI** (PmeI)	GTTTAAAC		Yali	-	-	+/-
**SmiI** (SwaI)	ATTTAAAT		Yali	-	-	+/-

[a] – Recognition sites are specified in both orientation for non-palindromic sequences.
[b] – Name of the module where an enzyme is used.

[c] – Last three columns describe which recognition sites should be excluded (-) or might be retained (+) for exact genetic element lists. Please pay attention that different requirements apply to homology arms (designated as IntUp and IntDn) depending on which module they are appropriated for. To assure linearization of deletion cassette before *Y. lipolytica* transformation, at least one of the four sites (marked as +/-) need to be excluded from IntUp and IntDn sequences designed for the Del Module.

Lvl0 entry vectors can be assembled using BsmBI and the pYTK001 plasmid which contains *sfGFP*[6]. Resultant clones can be selected on LB-Cm plates (Section 4.1) by the lack of green fluorescence. An example of Lvl0 assembly for a PCR-amplified gene is shown in Fig. S2. The resultant construct represents the pGen-series in the Lvl0 module. An example of the primers used to assemble a Lvl0 plasmid with *Y. lipolytica HPD1* gene is provided in Fig. S3. Note if genes are purchased commercially, vectors containing spectinomycin resistance must be avoided as this is used in the Lvl1-series of plasmids. Examples of primer structures used for assembly of Lvl0 plasmids with terminator shown in Figure S4. Figures S5 and S6 contain primers for amplification of up and down homology arms (IntUp and IntDn), respectively. We recommend to use 500bp at least for IntUp and IntDn sequences for purpose of assembly of overexpression constructs. Such sequence length is satisfactory for efficient homologous integration while keeping the probability of including undesired restriction sites low (Table S3). To design the homology arms for linearization with SmiI or MssI, which do not produce undesirable extra base at the ends please refer to Section 1.4. Primers for assembly of pPro-series of plasmid with promoter parts are shown in description of the Pro Module (Section 1.8).

The basic set of the toolkit is provided with set Lvl0 plasmids required for assembly of empty Lvl1 and Lvl2 vectors (Table S8). It contains four previously characterized strong constitutive *Y. lipolytica* promoters, including promoters of genes *TEF1*, *EXP1*, *TDH1*, *FBA1*[7], and four characterized terminators, including terminators of *Y. lipolytica* gene *LIP2*[8] and *S. cerevisiae* gene *ADH1*, *PGK1*, and *ENO2*[6]. The nomenclature used for Lvl0 plasmids is provided in Fig. S7.

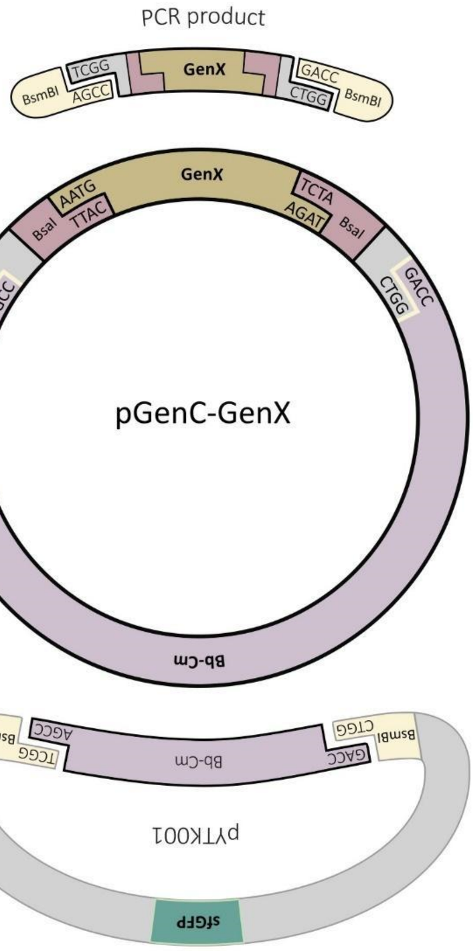

Fig. S2 Assembly of pGenC. A PCR-amplified gene is inserted into pYTK001 using GG assembly with BsmBI. The backbone contains a Cm-resistance marker (Bb-Cm).

HPD-F gcatCGTCTCATCGGGGTCTCAAATGTCACCTTCCGTCGAAGTC
HPD-R ctgaCGTCTCTGGTCGGTCTCATAGATTAAAGGTTGCCTCGCTTGGCCTG

BsmBI BsaI Start codon Stop codon

Fig. S3 pGen-series primer structure for insertion into vector. Primers shown here are an example for cloning *HPD1*. Lowercase bases are junk DNA to allow the Type IIs enzyme to cut. Underlined bases are 3'-terminal annealing regions that need to be designed specifically for the desired gene.

ScADH-F gcatCGTCTCATCGGGGTCTCTTCTATAACTCGAGGCGAATTTCTTATGA
ScADH-R atgcCGTCTCAGGTCGGTCTCTACTCGAAATGGGGAGCGATTTGCAGGCA

BsmBI BsaI

Fig. S4 pTer-series primer structure for insertion into vector. Primers shown here are an example for cloning the *ADH1* terminator. Lowercase bases are junk DNA to allow the Type IIs enzyme to cut. Underlined bases are 3'-terminal annealing regions that need to be designed specifically for the desired terminator.

IntC2Up-F
gcatCGTCTCATCGGGGTCTCTGCCTGCGGCCGCGTTTAAACACGCCAGACTTGGTTTATTAC

IntC2Up-R
gcatCGTCTCAGGTCGGTCTCTACCTACTAGCAGGTGTAGACTATCGGTAGAGCCAATTAGCTCCTCAAACGGAACTTTCCTG

BsmBI BsaI NotI MssI AarI Barcode1

Fig. S5 pYalC-IntUp-series primer structure for insertion into vector. Primers shown here are an example for cloning the upstream homology arm for integration into locus IntC2 of *Y. lipolytica*. Lowercase bases are junk DNA to allow the Type IIs enzyme to cut. Barcode1 is used as an annealing region for standard primer Barcode1-R to verify correct integration into the *Y. lipolytica* genome. This artificial sequence was also used as the first author authentic signature. Underlined bases are 3'-terminal annealing regions that need to be designed specifically for the desired upstream homology region.

IntC2Dn-F
gcatCGTCTCATCGGGGTCTCTGAGTACTAGCAGGTGTAAGAAGTTGGTGAAAACATTTACGCCTAACGCATTGCTTTTTCAGCCTT
C

IntC2Dn-R
gcatCGTCTCAGGTCGGTCTCTCGCAGCGGCCGCGTTTAAACGTGCAAAGGTGGAGAC

BsmBI BsaI NotI MssI AarI Barcode2

Fig. S6 pYalC-IntDn-series primer structure. Example primers for cloning the downstream homology arm for integration into locus IntC2 of *Y. lipolytica*. Lowercase bases are junk DNA to allow the Type IIs enzyme to cut. Barcode2 is used as an annealing region for standard primer Barcode2-F to verify correct integration into the *Y. lipolytica* genome. This artificial sequence was also used as the second author authentic signature. Underlined bases are 3'-terminal annealing regions that need to be designed specifically for the desired downstream homology region.

pYalA-Bb
pYalC-URA3
pYalC-IntB11Dn
pProC-TEF1
pGenC-hrGFP
pTerC-ScADH1

Series First letter of *E. coli* antibiotic resistance Genetic element

Fig. S7 Nomenclature of Lvl0 plasmids. pYal: plasmids suitable for assembling empty Lvl1 and empty Lvl2. pPro, pGen or pTer: plasmids suitable for assembling promoter, gene or terminator on Lvl1. Bb: plasmid backbone - includes high copy ColE1 origin of replication and antibiotic resistance.

1.3 (Exp Module) Assembly of overexpression constructs

The Exp Module is a multilevel GG platform for assembling up to three TUs together (Fig. S8). A single TU can be assembled by combining an empty Lvl1 vector with three Lvl0 plasmids each containing a promoter, gene or terminator. To construct a Lvl2.2 plasmid containing two TUs, an empty Lvl2.2 vector needs to be combined with a Lvl1.1 and Lvl1.2 plasmid. To construct a Lvl2.3 plasmid containing three TUs, a Lvl2.3 vector needs to be combined with Lvl1.1, Lvl1.2 and Lvl1.3 plasmids. In all cases, each Lvl1 plasmid contains a single TU.

With the Exp Module, a variety of empty Lvl1 and Lvl2 vectors can be assembled with Lvl0 plasmids. Lvl1 vectors contain Sp resistance gene whereas Lvl2 vectors contain Ap resistance gene. Depending on the Lvl0 plasmid set that is selected, an assembled empty vector will differ by locus of integration, levels and sublevels. The sublevel of the Lvl1 plasmids determines the position of the TU in the subsequent Lvl2 assembly. The sublevel of the Lvl2 plasmid indicates the number of TUs that might be assembled on it. The basic plasmid set (Table S8) only contains the *URA3* gene as the selectable marker as the marker-free approach is most commonly used for genomic integration.

YaliCraft parts are interchangeable with the most widely used *Y. lipolytica* GG system[2, 3]. However, the previous system utilised the same enzyme, BsaI, for the assembly of empty Lvl1 plasmids and for the subsequent assembly of a TU. As a result, an intermediate step for assembling empty vectors was introduced in the Exp Module. The intermediate plasmid set is named preLvl. A preLvl plasmid is assembled in a GG reaction using BsaI and contains the *mCherry* gene. To assemble an empty Lvl1 or Lvl2 vector from a preLvl plasmid, a GG reaction with BpiI is used instead. During this reaction, *sfGFP* replaces *mCherry* and appropriate overhangs for transcriptional unit assembly are inserted. The levels and sublevels of resultant empty vectors will depend on the selected Lvl0 plasmid with *sfGFP* which contains specific restriction sites and overhangs.

As part of the toolkit, there are 80 empty Lvl1 and Lvl2 plasmids designed for integration in 16 different genomic loci. These can be used for overexpression of up to 48 genes in the *Y. lipolytica* genome and this means that complex biosynthetic pathways can be built (Table S8). The nomenclature used for Lvl1 and Lvl2 plasmids is described in Fig. S9.

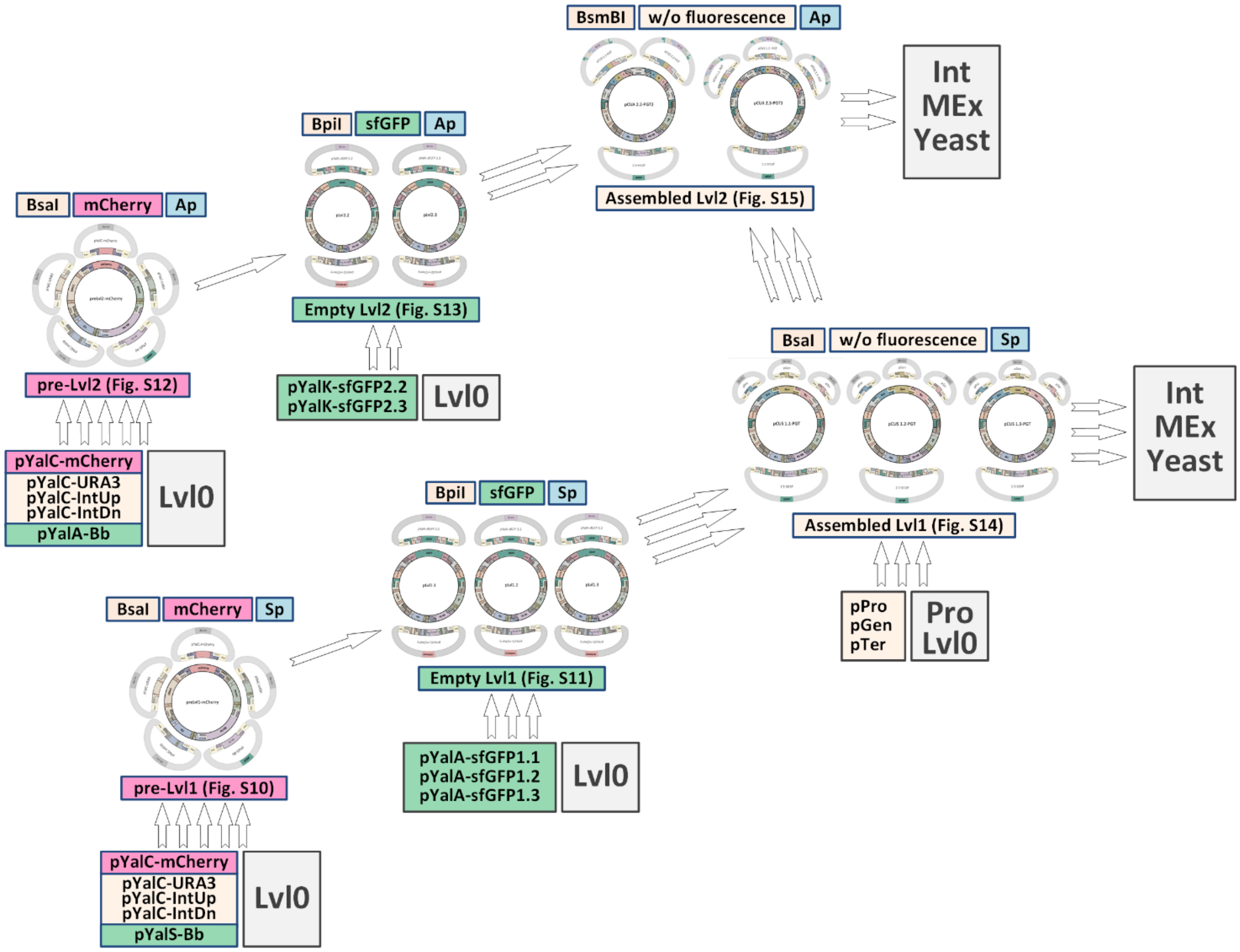

Fig. S8 General assembly scheme of empty vectors and overexpression constructs in the Exp Module. Each assembly is shown as a small pictogram. The plasmid series is shown below the picture

together with reference for full-size figures in brackets. The name of the enzyme used for assembly, fluorescent reporter, and antibiotic resistance are specified above the picture. Arrows show the direction of workflow for assembly. Plasmid names participating in each assembly are specified together with module names.

pB8US1.1
pF11UA2.3

Integration Region
First letter of yeast selectable marker
First letter of *E. coli* antibiotic resistance
Plasmid level Plasmid sublevel

Fig. S9 The nomenclature of Lvl1 and Lvl2 plasmids. A colour code is provided below the plasmid names.

1.3.1 Assembly of empty Lvl1 vector

To assemble a preLvl1 vector, five Lvl0 plasmids are combined: pYalC-mCherry, pYalC-URA3, pYalS-Bb, and two plasmids carrying homology arms corresponding to the selected integration locus. For the example in Fig. S10, pYalC-IntC2Up and pYalC-IntC2Dn are required to construct a preLvl1-mCherry plasmid for integration into the IntC2 locus. For GG assembly, Protocol 3.1 with BsaI enzyme and thermal inactivation is required. Correct clones are red fluorescent when selected for on LB-Sp. These clones can be visually identified on the Petri dish using a 530nm excitation light source and a 620nm filter.

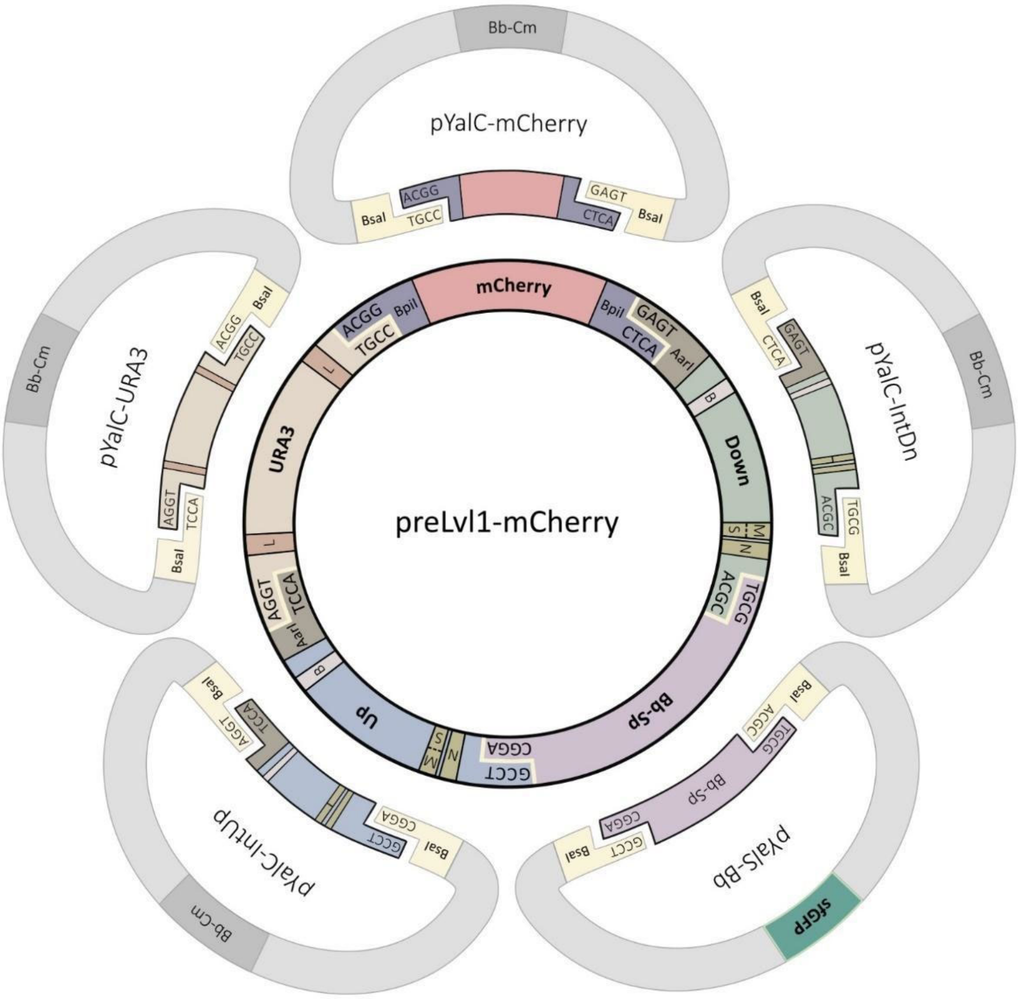

Fig. S10 Assembly of a preLvl1 plasmid. Overhangs are shown for each part. Yellow indicates enzyme cut sites. Abbreviations: B – barcode regions; Bb-Sp, Bb-Cm – backbones with different antibiotic resistances; L – Lox sites; M – MssI sites; N – NotI sites; S – SmiI sites. Up, Down – upstream and downstream homology arms.

Following preLvl-mCherry construction, empty pLvl1 plasmids with different sublevels are assembled. For this purpose, preLvl1-mCherry needs to be combined with one of three alternative plasmids: pYalA-sfGFP1.1, pYalA-sfGFP1.2 or pYalA-sfGFP1.3. This results in Lvl1.1, Lvl1.2 or Lvl1.3 plasmids (Fig. S11). For this reaction, use Protocol 3.1 with BpiI enzyme and thermal inactivation. Correct colonies can be selected for by green fluorescence (470nm excitation/580nm emission) when plated on LB-Sp.

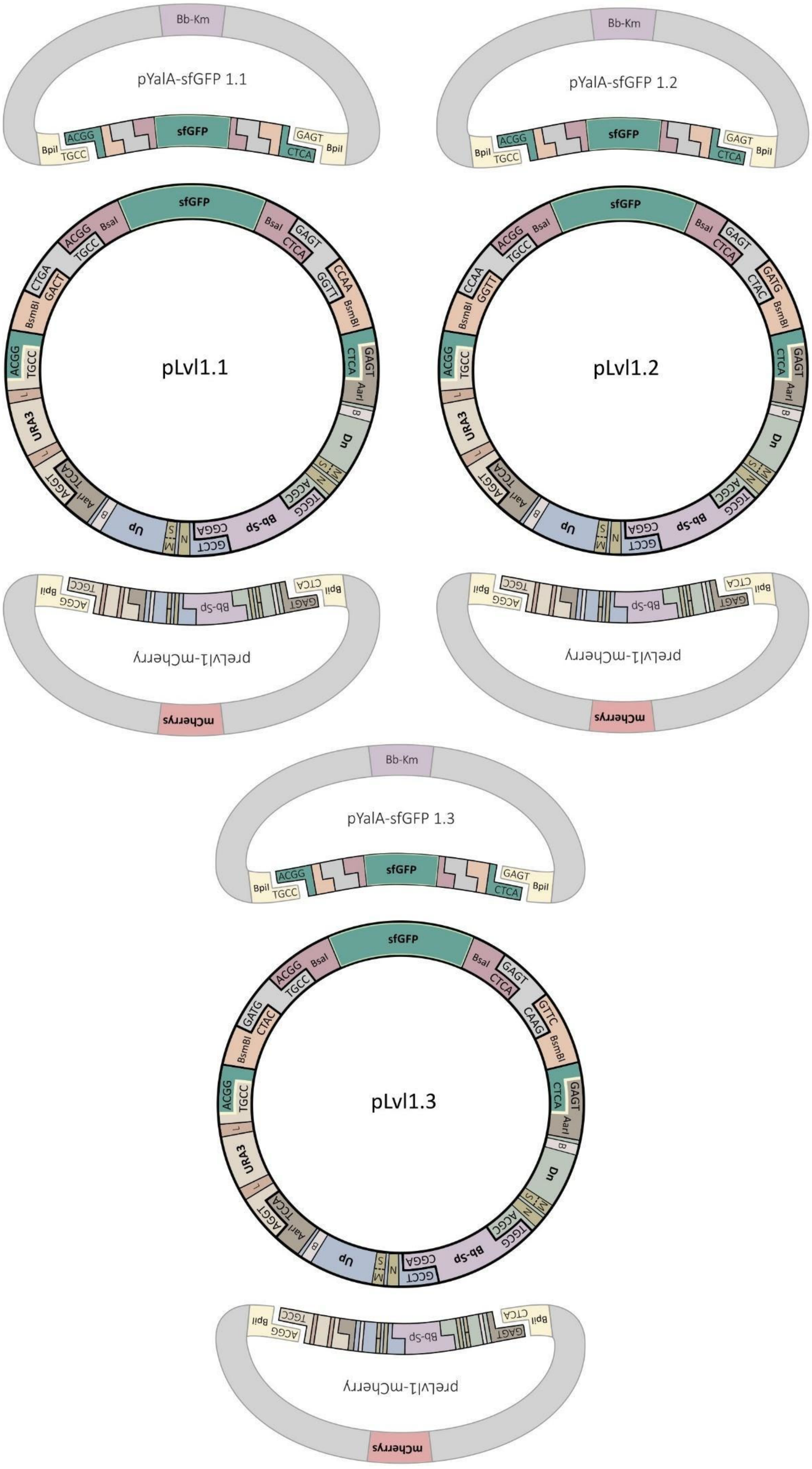

Bb-Km
pYalA-sfGFP 1.1
pYalA-sfGFP 1.2
pYalA-sfGFP 1.3
sfGFP
BpiI
ACGG
TGCC
GAGT
CTCA
BsaI
BsmBI
AarI
URA3
Up
Dn
Bb-Sp
pLvl1.1
pLvl1.2
pLvl1.3
preLvl1-mCherry
mCherry

Fig. S11 Assembly of empty pLvl1.1, pLvl1.2, and pLvl1.3 vectors. Overhangs are shown for each part. Yellow indicates enzyme cut sites. Abbreviations: B – barcode region; Bb-Sp, Bb-Ap – backbones with different antibiotic resistances; L – Lox sites; M – MssI sites; N – NotI sites; S – SmiI sites; Up, Dn – upstream and downstream homology arms.

1.3.2 Assembly of empty Lvl2 vector

Next, to assemble preLvl2 vector - preLvl2-mCherry - five Lvl0 plasmids are combined. This includes: pYalC-mCherry, pYalC-URA3, pYalA-Bb and two plasmids carrying homology arms corresponding to the selected integration locus (Fig. S12). For GG assembly of this plasmid use Protocol 3.1 with BsaI and thermal inactivation. Correct clones are selected by red fluorescence on LB-Ap plates.

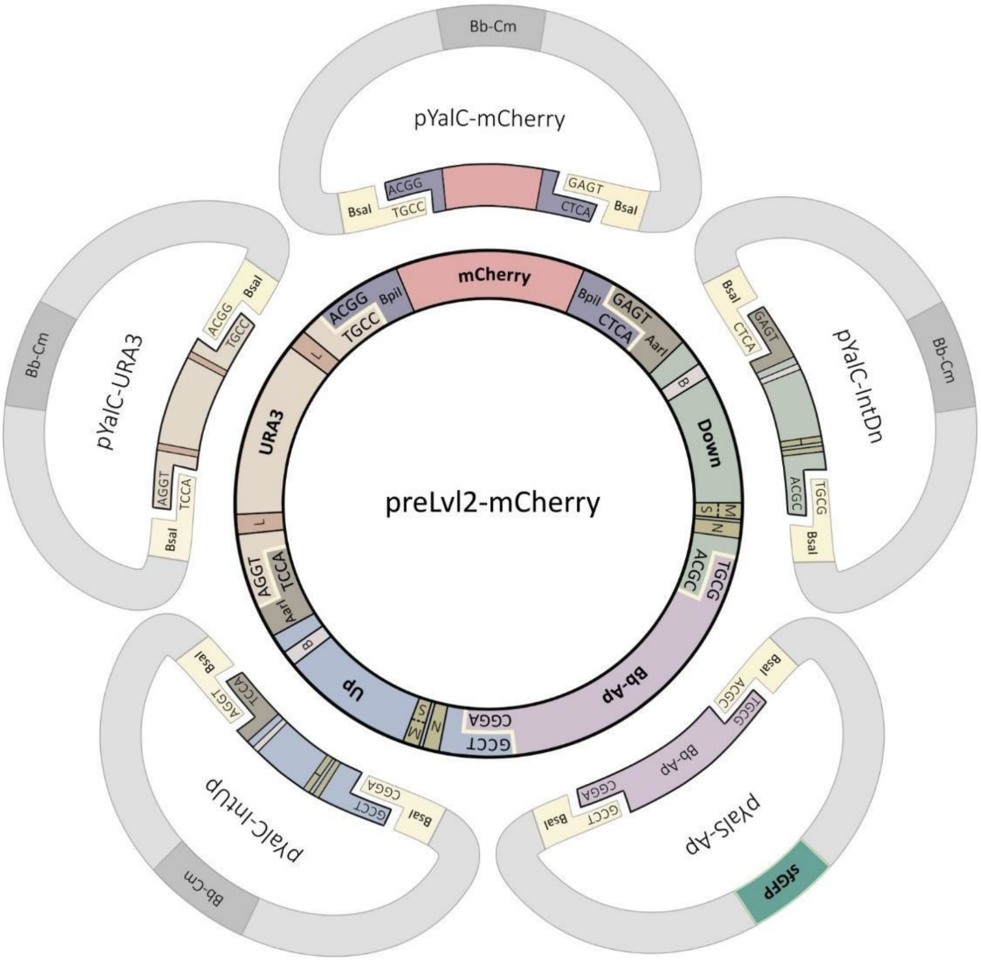

Fig. S12 Assembly of preLvl2 plasmid. Overhangs are shown for each part. Yellow indicates enzyme cut sites. Abbreviations: B – barcode regions; Bb-Ap, Bb-Cm – backbones with different antibiotic resistances; L – Lox sites; M – MssI sites; N – NotI sites; S – SmiI sites; Up, Down – upstream and downstream homology arms.

At the second stage, by combining the preLvl2-mCherry plasmid with one of two alternative plasmids pYalK-sfGFP2.2 or pYalK-sfGFP2.3, empty plasmids pLvl2.2 or pLvl2.3 can be obtained, respectively (Fig. S13). For these GG assemblies use Protocol 3.1 with BpiI enzyme and thermal inactivation. Correct clones are selected for by green fluorescence on LB-Ap.

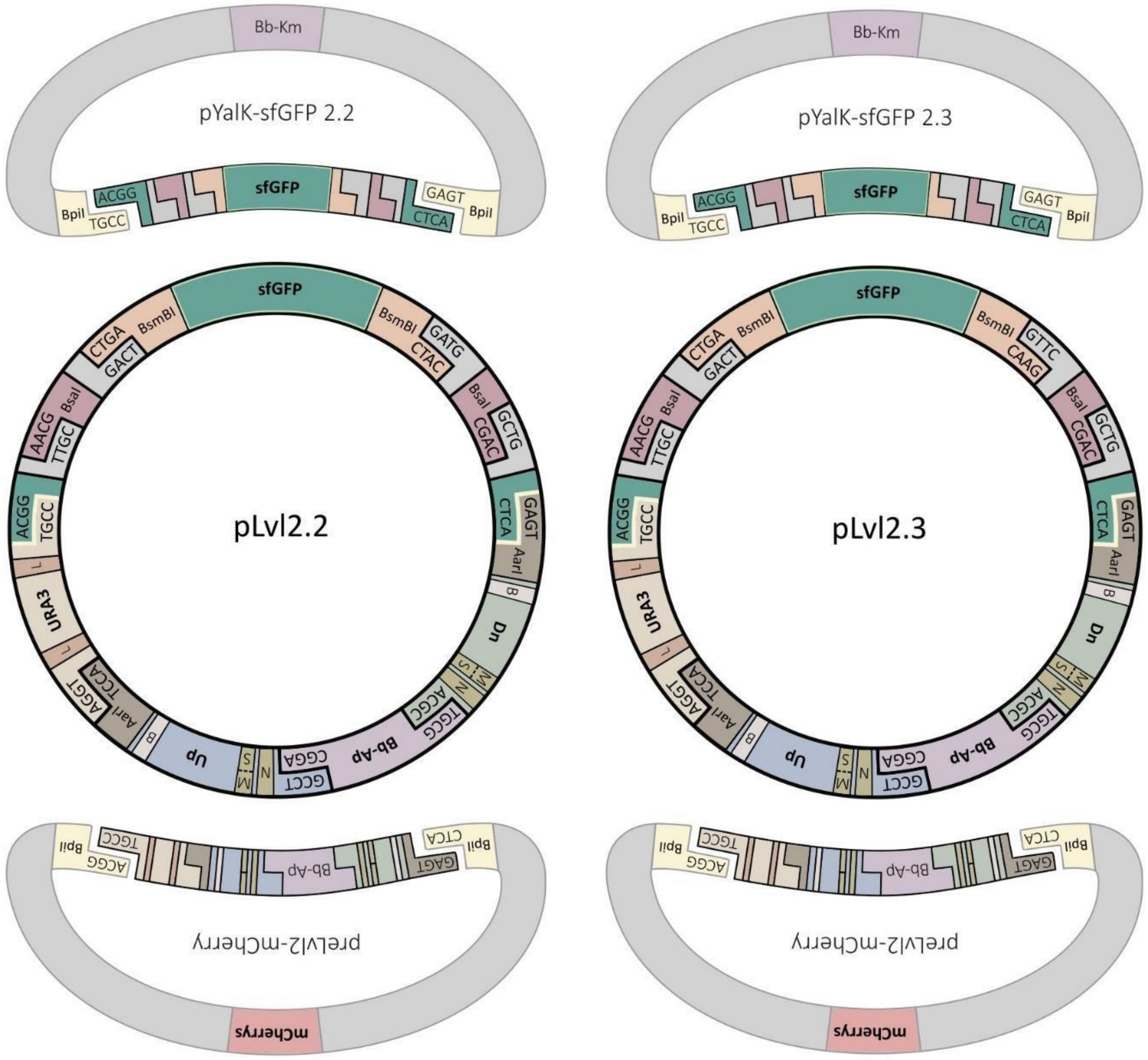

Fig. S13 Assembly of empty pLvl2.2 and pLvl2.3 vectors. Overhangs are shown for each part. Yellow indicates enzyme cut sites. Abbreviations: B – barcode region; Bb-Ap, Bb-Km – backbones with different antibiotic resistances; L – Lox sites; M – MssI sites; N – NotI sites; S – SmiI sites; Up, Dn – upstream and downstream homology arms.

1.3.3 Assembly of single TU on the Lvl1

For assembly of a single TU, an empty Lvl1 plasmid needs to be used. The choice of selected empty vector will determine the locus of integration of the resulting construct. The selected empty Lvl1 vector needs to be combined with three Lvl0 plasmids each with either a promoter (pPro-series), gene (pGen-series) or terminator (pTer-series) to make plasmids that can be used for Lvl2 assembly (Fig. S14). For GG assembly, use Protocol 3.1 with BsaI enzyme and thermal inactivation. Correct clones are selected for by lack of green fluorescence on LB-Sp.

Lvl1 plasmids also contain sublevels - 1.1, 1.2 or 1.3. The only difference between different sublevels is the overhangs produced by BsmBI. These overhangs are then used to determine the order in which TUs will be assembled on the Lvl2 plasmid. For example, to insert two TUs into a Lvl2 vector, you will need the Lvl2.2 backbone with pLvl1.1 containing the TU you want in the first position, and pLvl1.2 containing the TU you want in the second position (Fig. S15). Therefore, it is important to choose the correct Lvl1 sublevel for downstream Lvl2 assemblies.

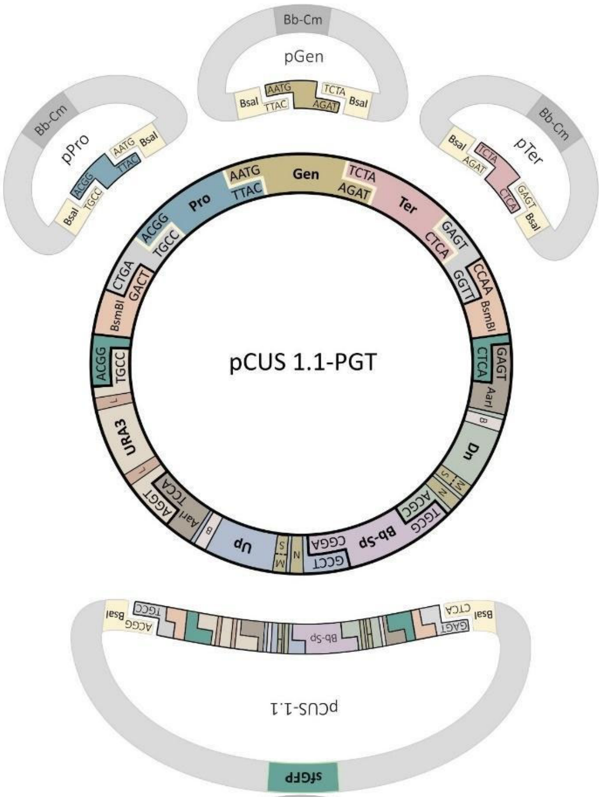

Bb-Cm
pGen
BsaI
AATG
TTAC
TCTA
AGAT
pPro
ACGG
TGCC
pTer
GAGT
CTCA
pCUS 1.1-PGT
Pro
Gen
Ter
CCAA
GGTT
CTGA
GACT
BsmBI
AarI
URA3
Up
Dn
Bb-Sp
TCCA
AGGT
ACGC
TGCG
CGGA
GCCT
pCUS-1.1
sfGFP

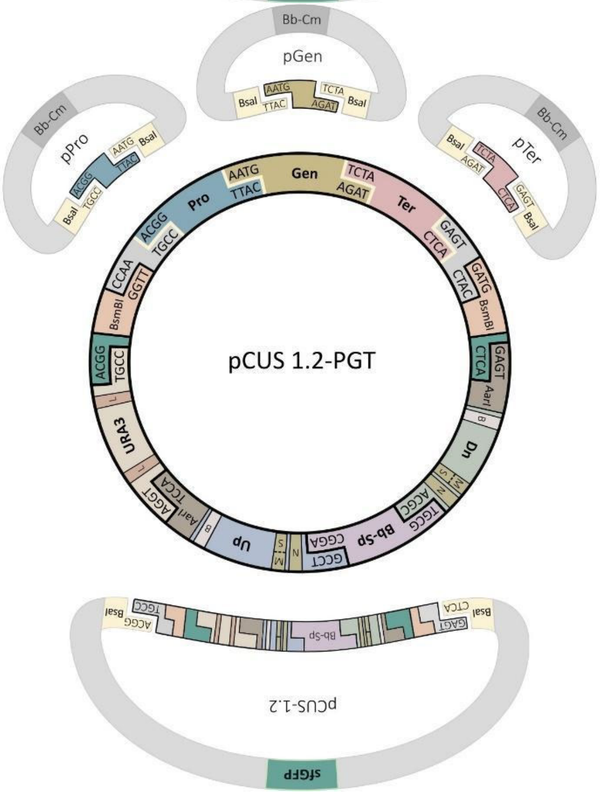

Bb-Cm
pGen
BsaI
AATG
TTAC
TCTA
AGAT
pPro
ACGG
TGCC
pTer
GAGT
CTCA
pCUS 1.2-PGT
Pro
Gen
Ter
GATG
CTAC
CCAA
GGTT
BsmBI
AarI
URA3
Up
Dn
Bb-Sp
TCCA
AGGT
ACGC
TGCG
CGGA
GCCT
pCUS-1.2
sfGFP

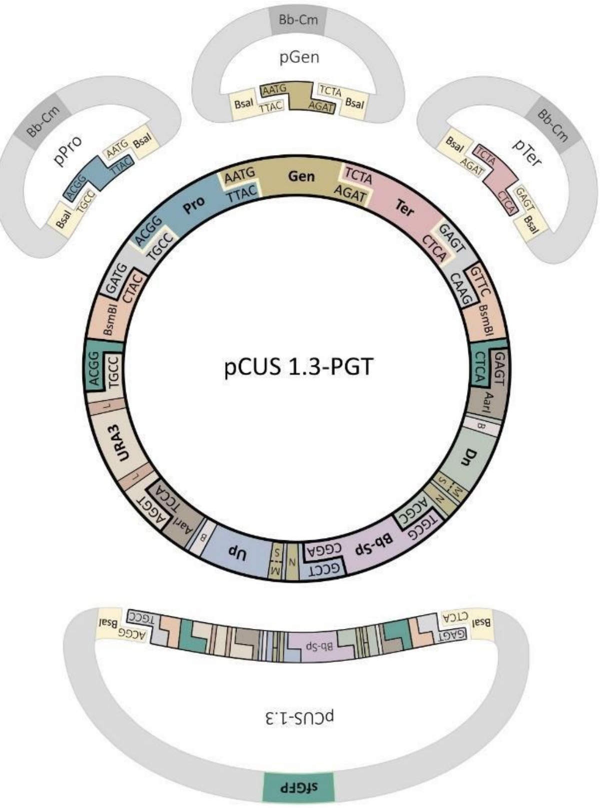

Fig. S14 Assembly of a single TU on the pLvl1.1, pLvl1.2, and pLvl1.3. The only difference between different sublevels is overhangs produced by BsmBI that determine the order in which TUs will be assembled on the Lvl2. Overhangs are shown for each part. Yellow indicates enzyme cut sites. Abbreviations: Pro – promoter, Gen – gene, Ter – terminator; B – barcode region; Bb-Sp, Bb-Cm – backbones with different antibiotic resistances; L – Lox sites; M – MssI sites; N – NotI sites; S – SmiI sites; Up, Dn – upstream and downstream homology arms.

1.3.4 Assembly of several TUs on the Lvl2

Empty Lvl2 vectors are used for the assembly of two or three TUs. The sublevel (2.2 or 2.3) will specify the number of TUs (two for 2.2 or three for 2.3) that can be assembled. Furthermore, the selected empty vector will determine the locus of integration of the resulting construct. Empty Lvl2.2 vectors need to be combined with two plasmids (Lvl1.1 and Lvl1.2) whereas empty Lvl2.3 vectors need to be combined with three Lvl1 plasmids (Lvl1.1, Lvl1.2 and Lvl1.3) (Fig. S15). As a result, the integrative vector can be assembled carrying TUs in the order determined by sublevels of the selected Lvl1 plasmids. For GG assembly, use Protocol 3.1 with BsmBI enzyme and thermal inactivation. Correct clones are selected for by lack of green fluorescence on LB-Ap.

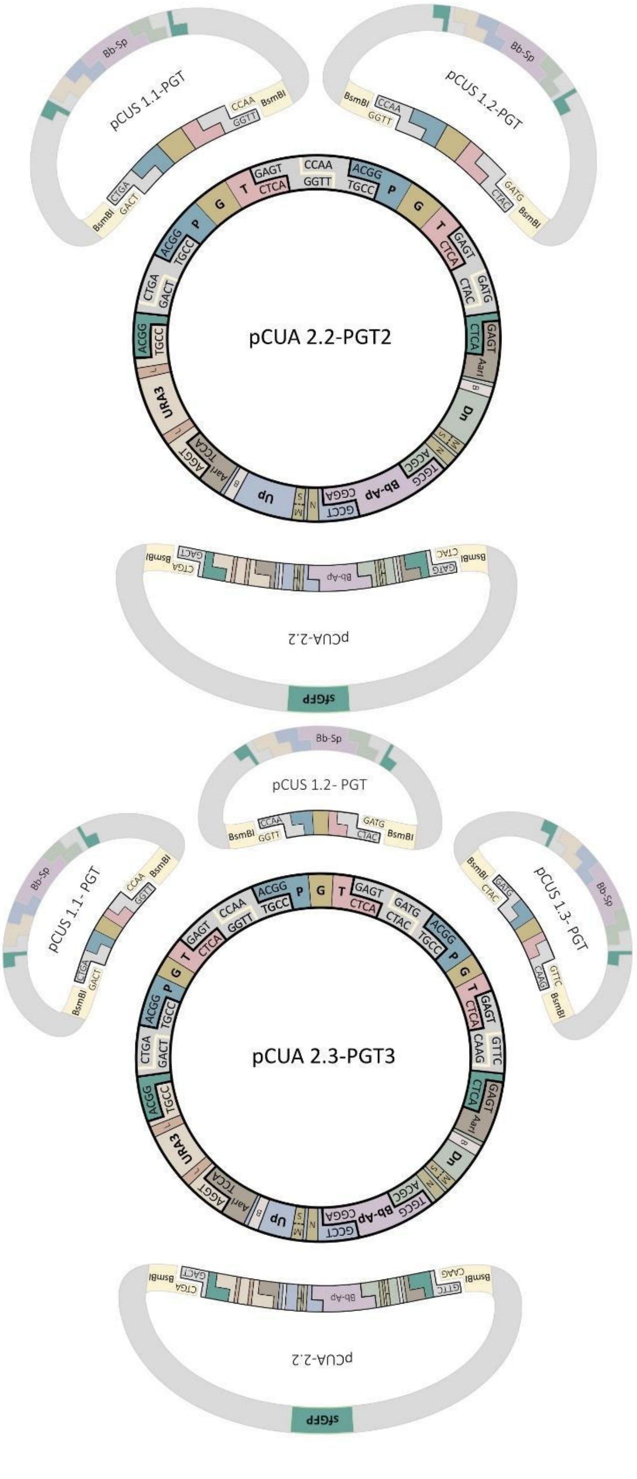

Fig. S15 Assembly of two TUs on the Lvl2.2 and three TUs on the Lvl2.3. Overhangs are shown for each part. Yellow indicates enzyme cut sites. Abbreviations: P – promoter, G – gene, T – terminator; B – barcode region; Bb-Sp, Bb-Ap – backbones with different antibiotic resistances; L – Lox sites; M – MssI sites; N – NotI sites; S – SmiI sites; Up, Dn – upstream and downstream homology arms.

1.4 (Del Module) Assembly of gene disruption construct

The Del Module utilises one-pot GG assembly and results in a vector that can be used to inactivate a gene in the *Y. lipolytica* genome. For this assembly, the empty pDelUK-RG vector is required which contains a *Y. lipolytica URA3* marker flanked by two *E. coli* reporter genes - *mScarlet* (RFP) and *sfGFP*. During the GG reaction, both reporters are replaced by PCR products amplified from the *Y. lipolytica* genome, which contain the sequences for upstream and downstream

homology arms (IntUp and IntDn) (Fig. S16). For GG assembly, use Protocol 3.3 with LguI enzyme and thermal inactivation. Correct clones are selected by lack of both green and red fluorescence on LB-Km (Fig. S17). The resulting pDel-series vector is a ready-to-use construct for inactivation of any selected gene. Importantly, due to the burden caused by the expression of two fluorescent proteins, the empty vector pDelUK-RG has a low copy number. Therefore, a separate plasmid isolation protocol is provided (Protocol 3.4). Afterwards, assembled pDel-series plasmids can be isolated using the general miniprep protocols (*e.g.* Qiagen #27104).

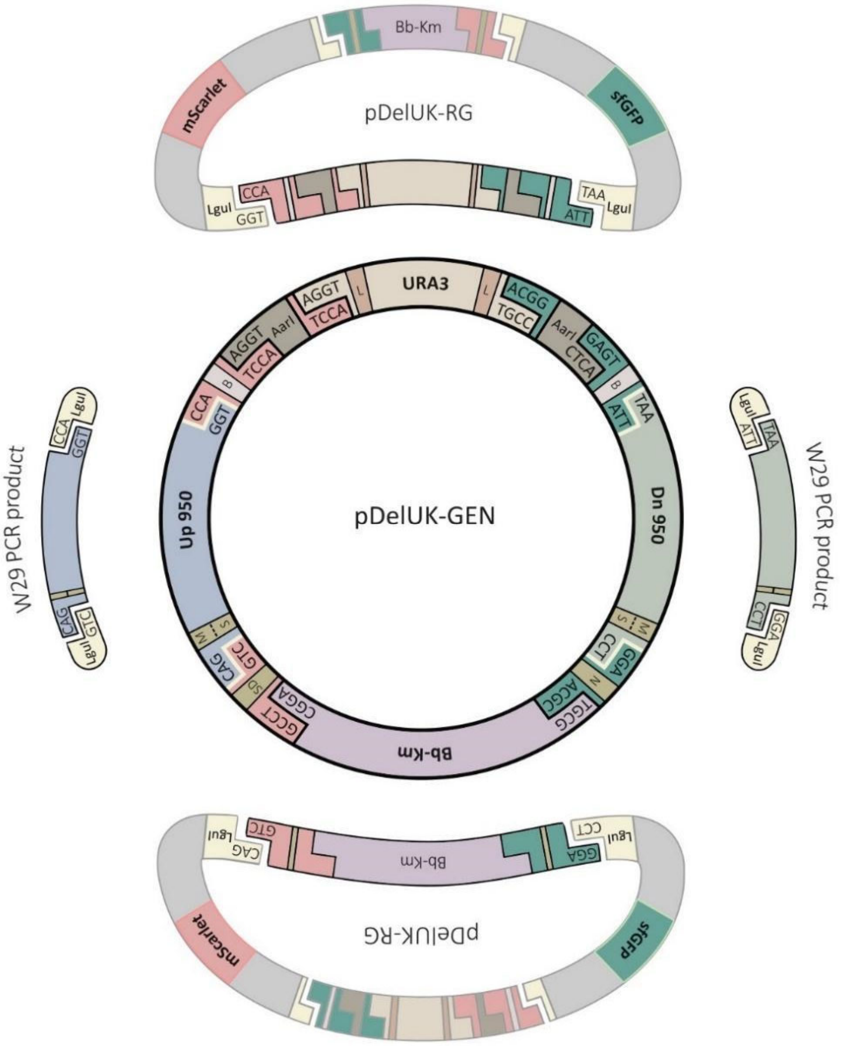

Fig. S16 Assembly of pDel-series plasmid. The same empty pDelUK-RG vector is shown at the top and the bottom. PCR products containing the homology regions from wild type *Y. lipolytica* strain are shown on the left and right. Overhangs are shown for each part. Yellow indicates enzyme cut sites. Abbreviations: B – barcode region; Bb-Km – backbones with Km-resistance gene; L – Lox sites; M – MssI sites; N – NotI site; SD – SgrDI site; S – SmiI sites; Up, Dn – upstream and downstream homology arms.

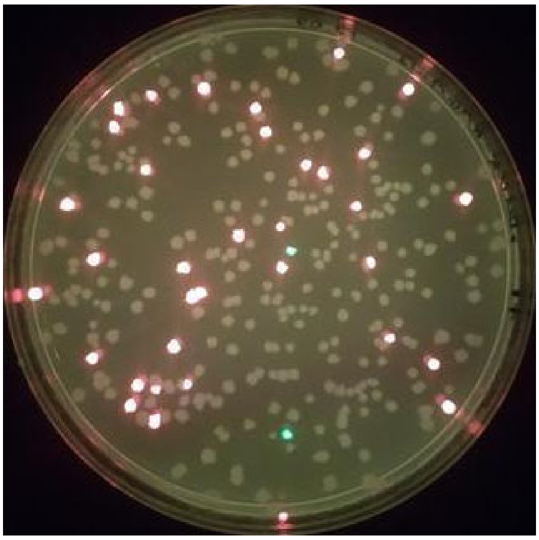

Fig. S17 Appearance of LB-Km plate containing *E. coli* transformed with GG assembly of pDel-series. Colonies that have been correctly assembled are not fluorescent under blue light (470nm excitation/580nm emission).

Lengths of homology arms may range from 400 - 1000 bp. Longer flanks are preferable for higher efficiency of homologous recombination, especially if marker-based integration is used without Cas9-helper application. However, the lengths of the selected homology arms may be limited by the presence of recognition sites (Table S3) used for their assembly and downstream applications. This issue is partially solved by the use of LguI and AarI, which recognize rarer 7-bp sites unlike most other Type IIs endonucleases which recognise 6 bp. LguI is required for assembly of pDel-series (Fig. S17), while AarI is utilised for homology arm exchange mechanism (Fig. S20). One more restriction enzyme is required for the linearization of final construction before *Y. lipolytica* transformation. Therefore, selected homology arms should not contain the recognition sites of at least one of the following enzymes: NotI, SgrDI, MssI or SmiI. All of them recognize rare 8-bp sites that greatly simplifies the task. The vector part of pDel-series contains standard SgrDI and NotI sites that flank the upstream and downstream homology arms respectively and can be used for linearization. Note that such linearization always leads to short non-homologous extensions (5 bp or more) from the both sides. Although, such extensions do not halt homologous integration, their effects on preciseness and efficiency of *Y. lipolytica* recombination still need to be clarified. Alternatively, homology arms can be designed in such way that halves of recognition sites are naturally present at the required positions and, therefore produced ends will show a perfect match with chromosomal sequences. For this purpose, MssI or SmiI sites are recommended. Both enzymes recognize 8 bp and produce blunt ends. Therefore, selected homology arms on the corresponding ends should contain either AAAC (half of MssI site) or AAAT (half of SmiI site). As an example of such 'scarless linearization' technique, we provide primers for *AAT1* gene disruption (Fig. S18). Note that homology arms have been chosen in such way that both of them contain halves of MssI sites as part of the natural sequences. Following this rule, we also designed most of the standard homology arms used for overexpression vectors of Lvl1 and Lvl2 (Table S5).

AAT1-Up-F	atgcGCTCTTCACAGGTTTAAACGCGTCTTTAACAGGCGAAAAAC
AAT1-Up-R	gataGCTCTTCTTGGCCTCCTTCTCGGCCTCTC
AAT1-Dn-F	atgcGCTCTTCATAATGTCGAGCAGGGCCACGAG
AAT1-Dn-R	gataGCTCTTCTTCCGTTTAAACTGTCCTACAGTTTACACAC
LguI MssI	

Fig. S18 Example primers used for cloning upstream and downstream homology arms of *AAT1*. This structure of primers can be used for the assembly of a disruption cassette using empty pDelUK-RG vector. Blue bases are MssI sites, while yellow bases are LguI sites. Lowercase bases are junk DNA to allow LguI to cut. Underlined are 3'-terminal annealing regions that need to be designed specifically for the gene of interest.

1.5 (Int Module) Exchange of homology arms

In comparison with other multigene overexpression toolkits, *YaliCraft* allows one-pot exchange of homology arms directly on overexpression constructs with one, two or three TUs. In this reaction, assembled Lvl1/Lvl2 overexpression constructs can receive homology arms from an empty Lvl1/Lvl2 vector or any disruption construct of the pDel-series. Homology arm exchange may be necessary to avoid the reassembly of TUs if multiple copies of the same overexpression construct need to be integrated, in case when an integration locus is already used or several alternative constructs were assembled for integration at the same locus. Besides, homology arm exchange enables quick and easy way to substitute one construction in the yeast genome with another. The one-pot GG protocols require only two days to get a redirected construct ready for yeast transformation. At the same time, the Int Module together with Cas Module (Section 1.7) represents the system for quick redirection of both components of Cas9-based integration system, *i.e.* donor and guide.

1.5.1 Exchange of homology arms between Lvl1 and Lvl2

An assembled Lvl2 plasmid with several TUs can be recombined with any available empty Lvl1 plasmid (Fig. S19). For the GG reaction, use Protocol 3.2 with AarI. Since the final construct also contains functional AarI sites, no thermal inactivation is applied after the final ligation step. The resultant plasmid with overexpression TUs and renewed homology arms (IntUp and IntDn) can be selected by lack of green fluorescence on LB-Sp plates.

A similar approach can be used in the opposite direction to transfer homology arms from any empty Lvl2 plasmid to an assembled Lvl1 plasmid with a single TU. The recombinant plasmid can be selected by lack of green fluorescence on LB-Ap.

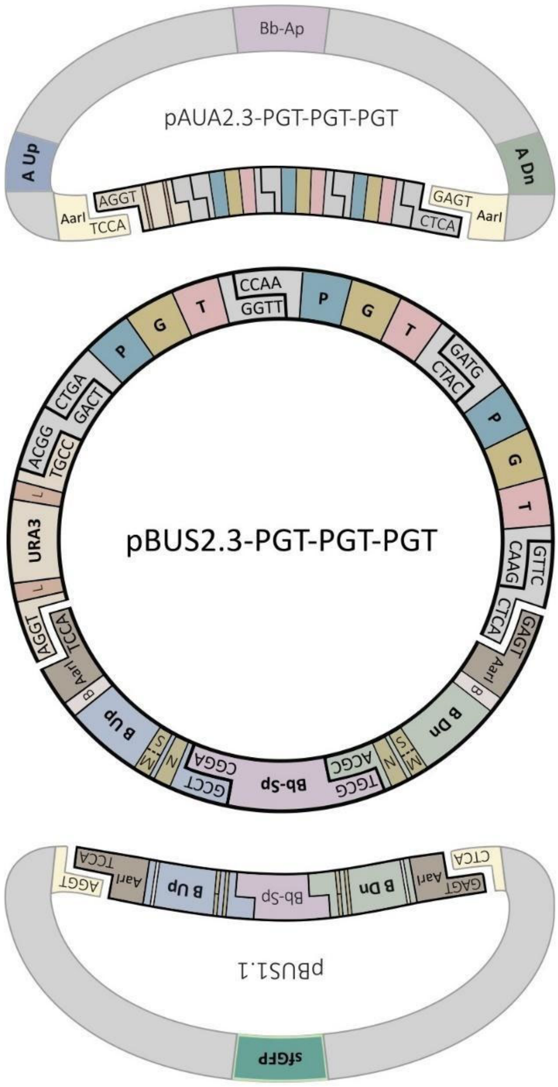

Fig. S19 Homology arms can be transferred from an empty Lvl1 vector (*e.g.* pBUS1.1) to an assembled Lvl2 plasmid (*e.g.* pAUA2.3-PGT-PGT-PGT) resulted in redirected vector (*e.g.* pBUS2.3-PGT-PGT-PGT). Abbreviations: P – promoter, G – gene, T – terminator; B – barcode region; Bb-Ap, Bb-Sp – backbones with different antibiotic resistances; L – Lox sites; M – MssI sites; N – NotI sites; S – SmiI sites; Up, Dn – upstream and downstream homology arms.

1.5.2 Transfer of homology arms from pDel-series to Lvl1 and Lvl2

Any disruption vector of the pDel-series can also be used as a donor of homology arms for overexpression constructs of Lvl1 or Lvl2 (Fig. S20). The orientation of AarI sites on the pDel-series makes this assembly irreversible because the new plasmid, when correctly assembled, does not contain any active AarI sites. To transfer homology arms between the pDel-series and Lvl1/2 plasmids, use GG reaction Protocol 3.1 with AarI enzyme and terminal inactivation. The recombinant plasmid with the overexpression construct (TUs) and renewed homology arms (IntUp and IntDn) can be selected for on LB-Km plates. Note that the resultant construct can be integrated in genomic loci with the already disrupted gene or in wild type gene, therefore, enabling simultaneous gene disruption and overexpression.

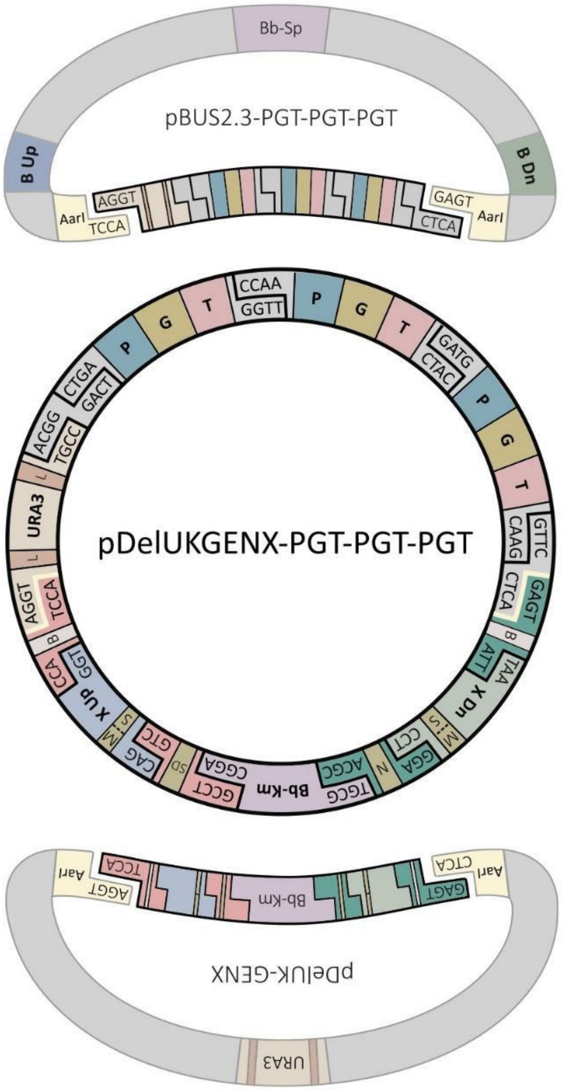

Fig. S20 Homology arms can be transferred from pDel-series plasmid (*e.g.* pDelUK-GENX) to an assembled Lvl2 plasmid (*e.g.* pBUS2.3-PGT-PGT-PGT) resulted in redirected vector (*e.g.* pDelUKGENX-PGT-PGT-PGT). Abbreviations: P – promoter, G – gene, T – terminator; B – barcode region; Bb-Km, Bb-Sp – backbones with different antibiotic resistances; L – Lox sites; M – MssI sites; N – NotI sites; SD – SgrDI site; S – SmiI sites; Up, Dn – upstream and downstream homology arms.

1.6 (MEx Module) Excision of yeast selectable marker

The MEx Module is designed for instant switching from a marker-free construction to a marker-based one. This becomes possible with construction of vector versions with and without a marker using the same GG assembly reaction mixture. For any assembly derived from the Exp, Del or Int Modules we recommend transforming GG reaction in two independent *E. coli* strains. One of them is a regular transformation host (such as DH5alpha, XL1-Blue *etc.*), while the other is *EcoCre* which under induction conditions (37 ⁰C) overexpresses Cre recombinase. This leads to instant *URA3* marker excision that is flanked by Lox66 and Lox71 sites in all constructs (Fig. S21). Transformants can be selected for on LB plates with an appropriate antibiotic depending on the plasmid backbone used. *EcoCre* is provided as part of the toolkit (Table S9). The protocol for preparing *EcoCre* competent cells is also provided (Protocol 3.6). Efficiency of the marker excision was 100% as

verified by restriction analysis and, therefore, the number of correct clones entirely depends on the GG reaction. As a result, the MEx Module saves on the DNA re-assemblies several working days every time when a marker-based construct is required for difficult modifications.

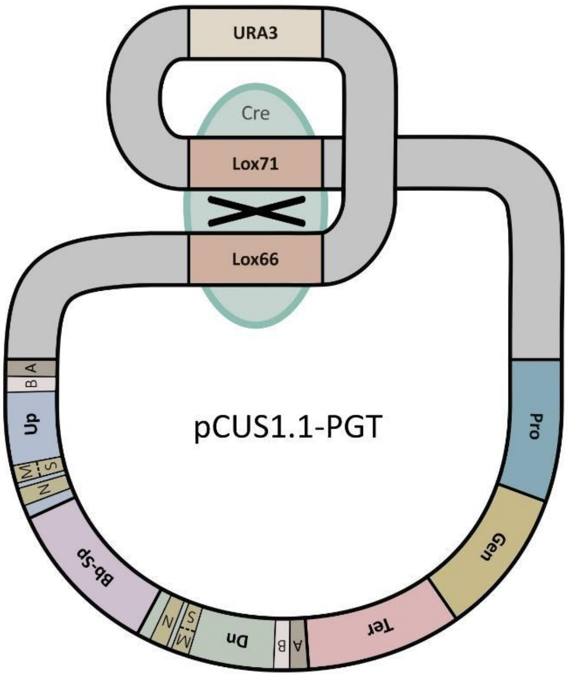

Fig. S21 Excision of *URA3* marker by *EcoCre* strain. Lvl1.1 plasmid is used here as an example to show how Cre loops-out the marker by site-specific recombination of the Lox66 and Lox71 sites. Abbreviations: Pro – promoter, Gen – gene, Ter – terminator; B – barcode region; Bb-Sp – backbones with Sp-resistances; A – AarI sites; M – MssI sites; N – NotI sites; S – SmiI sites; Up, Dn – upstream and downstream homology arms.

1.7 (Cas Module) Re-encoding Cas9-helper

The Cas Module allows redirection of episomal Cas9-helper plasmid to introduce double-strand breaks at any desired loci of the *Y. lipolytica* genome. An assembled Cas9-helper plasmid contains all the required genetic elements supporting this function *i.e.* a *Y. lipolytica* autonomously replicating sequence (ARS), nourseothricin (Nat) resistance marker, *Streptococcus pyogenes cas9* gene with nuclear localization signal (NLS), and Pol III-driven TU expressing guide (sgRNA) that includes a variable 20 bp recognition sequence. Only this short guide sequence needs to be changed to redirect Cas9-helper to new genomic loci.

The empty pCasNA-RK vector for this sequence contains a two-marker cassette with Km- resistance and streptomycin (St) susceptibility. The latter is an *rpsL* gene which enables counterselection in media with St. This counterselection is used to substitute the cassette with a 20 bp recognition sequence using oligonucleotide (oligo) recombineering (Fig. S22). To assemble new Cas9-helpers a 90-base oligo which contains these 20 nucleotides (nt) flanked by 35 nt homology regions on either side which recombines with the empty pCasNA-RK plasmid (Fig. S23) is required.

Specific 20 nt corresponding to the locus of interest can be designed using the ChopChop tool (http://chopchop.cbu.uib.no/). It is important to mention that for the purpose of marker-free integration, Cas9 should not recognize and cleave the donor construct, and therefore, these 20 bp need to be selected from the region between the upstream and downstream homology arms.

The assembly of a Cas9-helper plasmid requires mixing 70 ng of pCasNA-RK with 1.0 ug (35 pmol) of the 90 bp oligo followed by transformation of *EcoRed*. Recombination takes place inside the transformed strain which under induction conditions (37 ^{0}C) overexpresses three phage-derived lambda Red proteins: Gam, Exo, and Beta. *EcoRed* is provided as part of the toolkit (Table S9). The protocol for *EcoRed* competent cell preparation is also provided (Protocol 3.5). Transformants can be selected for at 30 ^{0}C on LB-Ap-St plate, verified using restriction digestion (*e.g.* with HindIII). If sequencing is required, primers sgRNA-seq-F and sgRNA-seq-R can be used (Table S10).

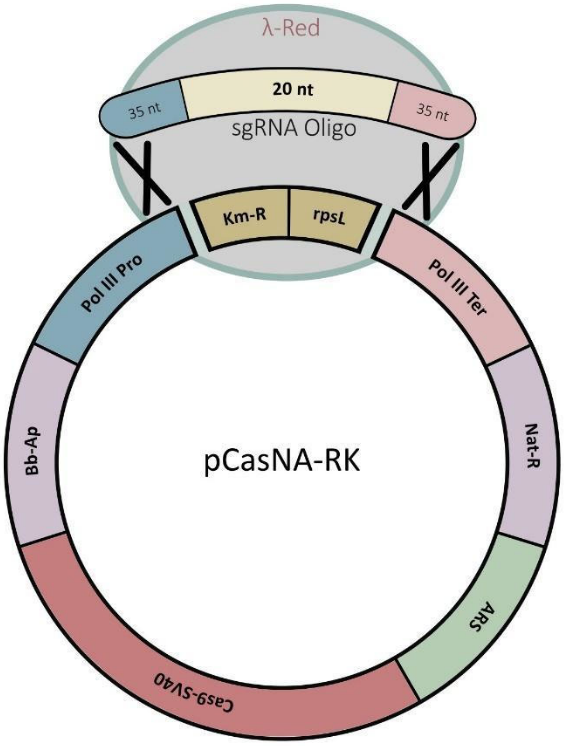

Fig. S22 Re-encoding of the guide sequence by oligo recombineering with empty Cas9-helper plasmid pCasNA-RK. The single-stranded oligonucleotide is 90 bp long and contains a 20 bp recognition sequence. Recombination occurs in the *EcoRed* strain. Transformed colonies are selected based on Sp-resistance which is a result of *rpsL* marker elimination. Abbreviations: Cas9-SV40 - *cas9* gene with SV40 NLS; Km-R - Km-resistance gene; Nat-R – Nat-resistance gene; Pol III Pro - hybrid promoter SCR1'- tRNA^{Gly} of RNA polymerase III; Pol III Ter - terminator of RNA polymerase III.

Recognition sequence in the genome	(**CACGAGCTTGAGCACTCGAGCGG**)
TCGATGGGCCCCCGGTTCGATTCCGGGTCGGCGCA**CACGAGCTTGAGCACTCGAG**GTTTTAGAGCTAGAAATAGCAAGTTAAAATAAGGC	

Fig. S23 Structure of 90-base single-stranded oligo used for assembly of pCas-series plasmids. An example for assembling a pCasNA-URA3 helper plasmid that cuts inside the *URA3* gene is shown. Green, 35-base homology regions to plasmid sequence; these are kept the same for all targets. Blue, conservative NGG sequence of protospacer adjacent motif (PAM) that should not be included in the sequence of the oligo. Yellow, 20 bases of the recognition sequence - the only part that need to be changed during design of a new oligo.

Several alternative approaches have been used to change the 20 bp recognition sequence on episomal Cas9-helpers, including USER cloning[9] and GG assembly[10] as the most reliable and fast. For all methods involving propagation in *E. coli*, a CRISPR/Cas9 modification cannot be introduced in less than four days due to the limitation of the growth rates, *i.e.* one day (from day 1 to day 2) for cultivation of the bacteria and two days (from day 2 to day 4) for the yeast. Cloning-free assembly, which skips the E. coli step by using the gap repair mechanism in *S. cerevisiae,* enables yeast transformant isolation on day 3[11, 12]. However, this method is not applicable for most non-*Saccharomyces* yeasts and therefore is not considered here. Alternatively, GG reaction mixture can be directly transformed in yeast[13]. However, this protocol still takes four days, since the transformed yeast requires a longer recovery time. The undeniable advantage of the cloning-based techniques is that assembled plasmids can be used repeatedly many times without extra manipulations and costs such as PCR, restriction or GG reaction. Considering all the above, we have developed a recombineering-based approach, which is easier in implementation than other cloning-based methods. This cloning-free technique requires less effort and time, while producing ready-to-use plasmids, and it is suitable for all yeast species regardless of specific peculiar recombination machinery (Table S4).

Table S4 Comparison of alternative approaches of Cas9-helpers assembly

Assembly method	Day 1	Day 2	Day 3	Day 4	Total time spent	Reference
**Plasmid-required systems**						
**GG assembly (with cloning)**	● Phosphorylating oligos<br>● Annealing oligos<br>● GG assembly<br>● *E. coli* transformation	● Colony PCR<br>● Culturing and miniprep<br>● Yeast transformation				10
**USER cloning**	● Annealing oligos<br>● USER reaction<br>● *E. coli* transformation	● Colony PCR<br>● Culturing and miniprep<br>● Yeast transformation	Yeast colony growth	Yeast colon<br>y PCR	4 days	9
**Recombineering**	● *E. coli* transformation	● Culturing and miniprep<br>● Restriction analysis<br>● Yeast transformation				this study
**Cloning-free system**						
**GG assembly (cloning-free)**	● PCR and purification<br>● GG assembly<br>● Yeast transformation	● Plating after recovery	Yeast colony growth	Yeast colon<br>y PCR	4 days*	13

* - the time mentioned in original paper was 3 days, which was the time interval (from day 1 to day 4) and did not include the first experimental day

1.8 (Pro Module) Introduction of new promoters

The Pro Module is designed to build and test new *Y. lipolytica* promoters. The vector pProUA-mScarlet is required for GG assembly of new promoters (Fig. S24). In the same way as other genetic elements from the Lvl0 Module, promoters assembled on the pProUA-series can be used to assemble TUs on Lvl1 plasmids using BsaI (Fig. S14). However, in contrast to plasmids in the Lvl0 Module, the pProUA-series contains humanized *Renilla* green fluorescent protein gene (*hrGFP*) which is codon-optimized for expression in *Y. lipolytica* and regulated by the promoter of interest. Such plasmids can be integrated into the standard IntC2 locus of the *Y. lipolytica* genome using specific Cas9-helper (pCasNA-IntC2) and the integration confirmed using *URA3* marker. Therefore, assembled pPro-series could be used for both purposes: for assembly of TUs and for promoter characterisation assays in the yeast (Section 2.6). As an example, the sequences of primers used for cloning the *Y. lipolytica ALK1* promoter are shown in Fig. S25. For GG assembly, use Protocol 3.1 with BpiI enzyme and thermal inactivation. Clones are selected for on LB-Ap by lack of red fluorescence.

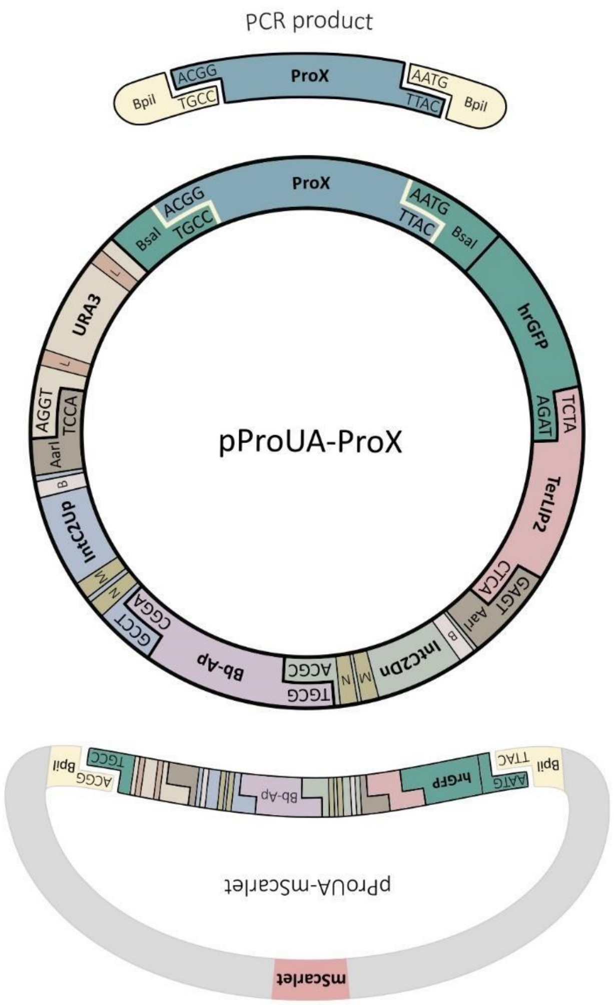

Fig. S24 Assembly of pProUA-series with new promoter. Note that the resultant plasmid contains two BsaI sites which enable assembly on a Lvl1 vector. Yellow indicates enzyme cut sites. Abbreviations: ProX – promoter to be screened; TerLIP2 – terminator of *LIP2* gene; IntC2Up, IntC2Dn – IntC2 upstream and downstream homology arms; B – barcode region; Bb-Ap – backbones with Ap-resistances; L – Lox sites; M – MssI sites; N – NotI sites.

ALK1-F	gcatGAAGACTCACGGACTGACTTGATACGCAACTG
ALK1-R	atgcGAAGACAGCATTGTGCAGGAGTATTCTGGGGAG
Bpil Start codon	

Fig. S25 Structure of primers for assembly of pProUA-series. Example here shows primers for cloning the *ALK1* promoter of *Y. lipolytica*. Lowercase are junk DNA to allow the Type IIs enzyme to cut. Underlined are 3'-terminal annealing regions that need to be redesigned when adding a new promoter.

2. YARROWIA ENGINEERING

YaliCraft provides the user with a high degree of freedom in the order of assembling genetic elements and eliminates the need to rebuild plasmids from scratch when switching between different integration approaches or alternative loci. A schematic representation of potential iterations of marker-free and marker-based approaches is proposed in Figure S26. As it can be seen from the estimated time periods, the marker-free technology presented here improves the rate of metabolic engineering in *Y. lipolytica* at least twofold when compared to approaches using

integration of a single selectable marker, while at the same time allowing the user to return to a marker-based approach without extra assembly steps.

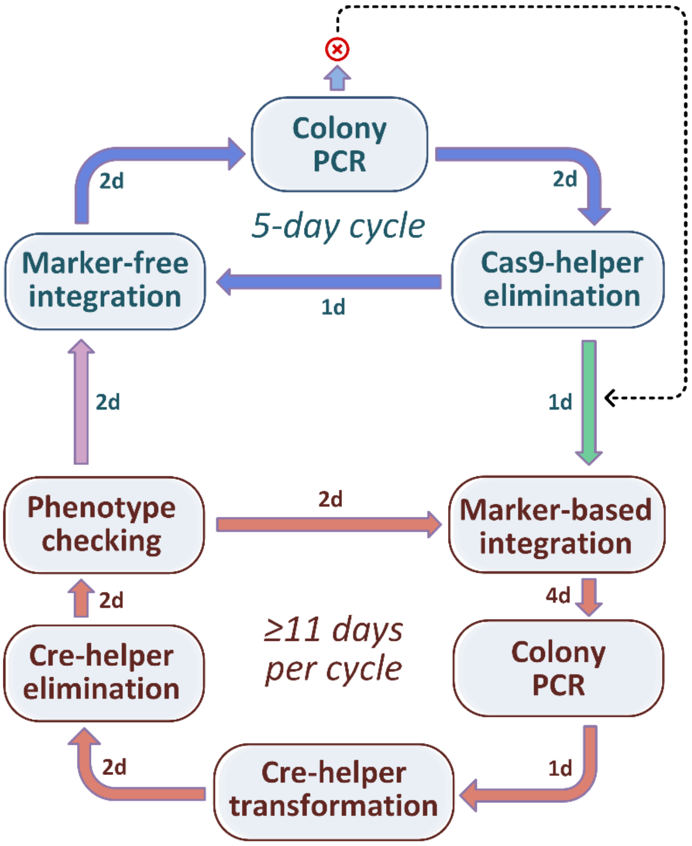

Fig. S26 Alternation of marker-free and marker-based integration techniques during *Y. lipolytica* engineering. Blue arrows, marker-free integration cycle. Red arrows, marker-based integration cycle. The marker-free cycle is less than half in duration and can be applied repeatedly as the main strategy. However, if no correct integrative transformants were isolated using this approach (red cross), then (dash line) marker-based construction needs to be transformed (green arrow). After the marker recovery procedure has been completed, the marker-free integration cycle can be restarted (purple arrow). The number of days required for each step and whole cycles are based on experimental procedures (Sections 2.2, 2.3 and 2.5).

2.1 Functional characterization of standard integration loci

Initially, 16 intergenic regions in *Y. lipolytica* genome were selected based on the following criteria: 1) located at least 1000 bp from the start codon and 500 bp from the stop codon of the nearest annotated open-reading frames, and 2) separated from the chromosome ends and from each other at least by 200 kb. The loci on each chromosome have been numbered and named accordingly as IntA1, IntA2, etc. They were chosen for designing homology arms (HA) 500-bp up and down the integration site. One extra set of integration flanks was designed with Zeta sequences instead of HAs. Zeta sequences do not show any homology to *Y. lipolytica* W29 and are usually used for random genomic integration.

For each integration locus, a set of five assembled empty vectors (Lvl1.1, Lvl1.2, Lvl1.3, Lvl2.2, and Lvl2.3) and Cas9-helper have been provided (Table S8). To characterise each locus, Lvl1.1 plasmid was assembled with a *hrGFP* reporter gene under the control of the *TEF1* promoter and *LIP2* terminator. Obtained plasmids were integrated as described in Section 2.6. Efficiency of Cas9-mediated integration together with other essential data for standard loci are summarized in

Table S5. The relative expression levels in different loci are provided on the Figure S27. Notably, in our case, the 'position effect' changes gene expression levels less than two times, which is very similar to previously published data for *Y. lipolytica*[9].

Table S5 Summarized data about 16 standard integration loci and corresponding homology arms

Integration locus	Chromosomal sequence GenBank#	IntUp homology arm position on the chromosome	IntDn homology arm position on the chromosome	Left flank integration primer (product with second primer Barcode1-R)	Right flank integration primer (product with second primer Barcode2-F)	20bp of sgRNA used on Cas9-helper	Enzyme for vector linearization	Integration efficiency % (number of correct clones among tested)
**IntB8**	CP017554.1	1280877..1281379	1281771..1282286	IntB8-chr-F (716bp)	IntB8-chr-R (756bp)	AAGTTCAAGATACTATACCC	MssI/NotI	75% (3/4)
**IntB11**	CP017554.1	2408220..2408818	2409833..2410434	IntB11-chr-F (813bp)	IntB11-chr-R (918bp)	AATGTTTGAGAGACAACGAC	SmiI/NotI	63% (5/8)
**IntC2**	CP017555.1	161071..161570	162857..163356	IntC2-chr-F (594bp)	IntC2-chr-R (601bp)	TCAAAAGTCAGTGTGAGGGG	MssI/NotI	75% (6/8)
**IntC7**	CP017555.1	1095374..1095882	1096160..1096676	IntC7-chr-F (685bp)	IntC7-chr-R (665bp)	TTATGAGACCCGATACAAGG	SmiI/NotI	50% (3/6)
**IntC13**	CP017555.1	2646315..2646874	2647563..2648077	IntC13-chr-F (713bp)	IntC13-chr-R (826bp)	AGACATGATGCATATACACA	SmiI/NotI	63% (5/8)
**IntC14**	CP017555.1	3133312..3133958	3134647..3135244	IntC14-chr-F (852bp)	IntC14-chr-R (858bp)	TCTCCATAGGTGTAGAACCA	SmiI/NotI	38% (3/8)
**IntD6**	CP017556.1	1494593..1495114	1495784..1496287	IntD6-chr-F (776bp)	IntD6-chr-R (696bp)	AAGCTTAATAAACAGCAAAG	NotI	75% (6/8)
**IntD12**	CP017556.1	2194396..2194895	2195646..2196145	IntD12-chr-F (595bp)	IntD12-chr-R (607bp)	GGTGGTGGTTATAATCGCTG	MssI/NotI	63% (5/8)
**IntE6**	CP017557.1	895599..896101	896482..896990	IntE6-chr-F (690bp)	IntE6-chr-R (698bp)	TACGAGTAGAGACGTAAACG	SmiI/NotI	67% (2/3)
**IntE8**	CP017557.1	1739277..1739776	1740717..1741216	IntE8-chr-F (589bp)	IntE8-chr-R (597bp)	AGGATAGTGCTTGTGTCCAG	SmiI/NotI	75% (3/4)
**IntE12**	CP017557.1	2831926..2832470	2832716..2833196	IntE12-chr-F (881bp)	IntE12-chr-R (1033bp)	ACCCCTATAGCCCAAACTGT	SmiI/NotI	63% (5/8)
**IntE15**	CP017557.1	3368514..3369040	3369472..3369975	IntE15-chr-F (715bp)	IntE15-chr-R (712bp)	TGCACTCTACAGGAACACTC	SmiI/NotI	75% (6/8)
**IntE16**	CP017557.1	3975493..3976035	3976233..3976833	IntE16-chr-F (817bp)	IntE16-chr-R (815bp)	TACTACTTGTAGTAGGACCA	SmiI and MssI/NotI	100% (8/8)
**IntF8**	CP017558.1	2626127..2626644	2627341..2627851	IntF8-chr-F (743bp)	IntF8-chr-R (720bp)	AGAGATCTATATGGTTAACG	MssI/NotI	63% (5/8)
**IntF9**	CP017558.1	2834729..2835240	2836124..2836625	IntF9-chr-F (814bp)	IntF9-chr-R (628bp)	CACGAAGAGAGGTAAAACAG	SmiI/NotI	100% (8/8)
**IntF11**	CP017558.1	3458258..3458790	3459091..3459592	IntF11-chr-F (799bp)	IntF11-chr-R (711bp)	AGTGGTATGGCTCTTCACAC	MssI/NotI	50% (2/4)

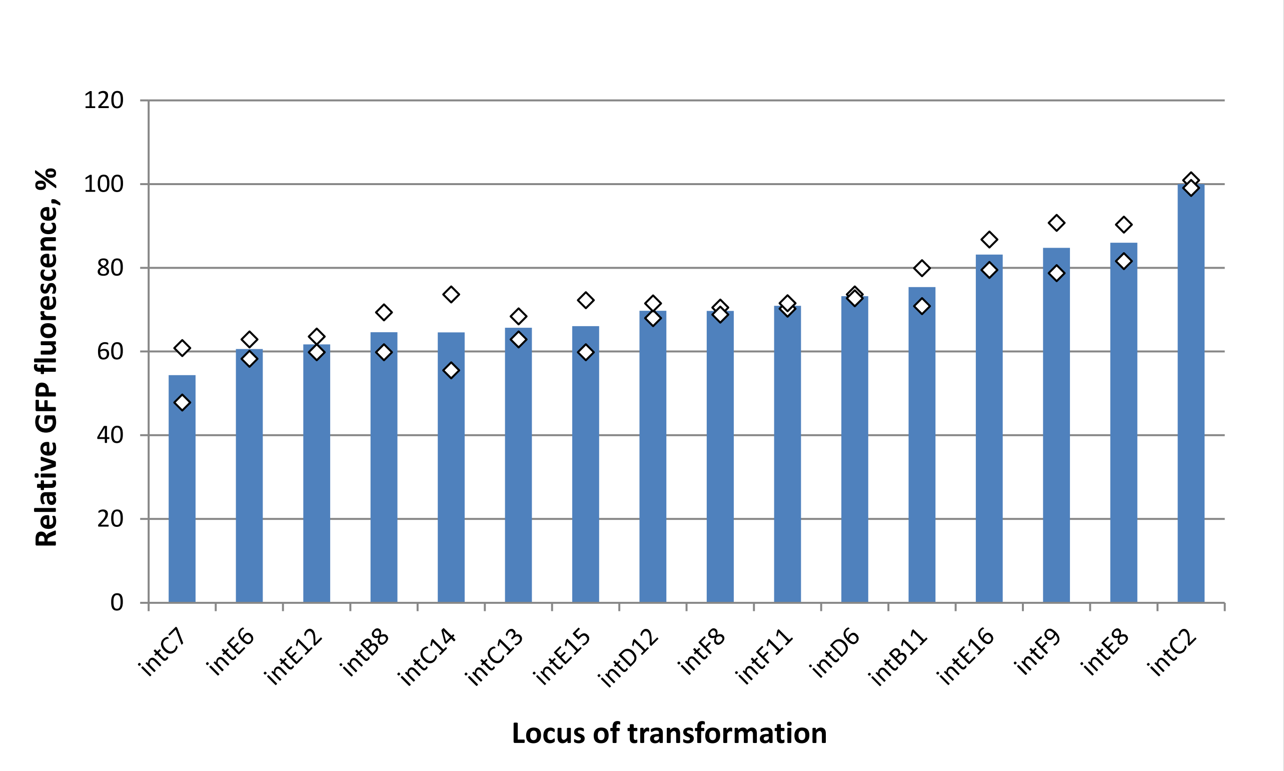

Fig. S27 Comparison of *hrGFP* expression under the control of *TEF1* promoter and *LIP2* terminator in 16 standard integration loci. The fluorescence (528 nm) was measured for cells growing in YPD medium in a plate reader after excitation at 485 nm. The values were normalized on biomass (OD_{600}) and auto-fluorescence of the parental strain S234 was extracted.

2.2 Marker-free CRISPR/Cas9-mediated integration

Once a vector with the appropriate integrating HA has been chosen, the user can proceed to the plasmid construction. It is recommended to construct both marker-based and marker-free vectors. Using the marker-free construct is suggested to be attempted first given its shorter transformation cycle. The Cas9-helper (encoding Cas9 and guide) and integration construct (donor) need to be isolated in amounts sufficient for transformation. As an aside, we recommend verifying the plasmid concentrations based on gel electrophoresis, rather than solely on spectrophotometry. Spectrometry allows estimate total amount of DNA along with other impurities, while the fragment of interest may comprise a small portion in the mix or even be destroyed by nucleases, such as endonuclease A. The donor plasmid needs to be linearized using a restriction enzyme (Table S3 and Table S5), followed by purification and concentration using a gel purification kit (*e.g.* Zymoclean D4002, Cambridge Bioscience). The gel purification of integrating fragment is not required.

A single transformation requires 500ng of undigested Cas9-helper plasmid and between 500ng and 2000ng (larger constructs require more DNA) of integrative fragment that need to be mixed together in a total volume of 10-15 µL. The *Δku70* strain should be used for marker-free *Y. lipolytica* transformation. Examples of appropriate strains include W29ΔuraΔku70 or W29Δku70ura+ (Table S9). The standard *Y. lipolytica* protocol needs to be applied for the transformation (Protocol 3.8). After heat shock and the single wash step, cells need to be plated on YPD with Nat (Section 4.1). Cells should be left to incubate for two days, after which several (3 to 8) large colonies are chosen and verified using colony PCR (Protocol 3.9). Some background growth of non-transformant clones can be also observed especially after prolonged incubation, however this mock colonies usually smaller than true transformants and can be distinguished based on colony sizes. Standard primers for colony PCR are suggested for both left and right flanks for all sixteen

integration loci (Table S5 and Table S10). If pDel-series was used, two new primers need to be designed corresponding to chromosomal regions flanking the insertion.

Successfully verified colonies then need to be streaked to single colonies and further incubated for 2 days on YPD plates. It is important to use medium without antibiotic at this step, because longer exposure of culture to Nat increases the chance of selecting a resistant clone, as described below. In the majority of cases, this procedure leads to elimination of unstable episomal Cas9-helper. From this point, the whole marker-free integration procedure can be repeated with a new construct. Nonetheless, we recommend using two independent transformants for the next transformation round. This might be helpful in the case that a Nat-resistant mutant was isolated or transformant which carries the helper plasmid integrated in the genome. If this is the case, after two days' incubation, there would be a lawn instead of individual clones on the transformation plate. Overall, a single round of marker-free integration (from one transformation experiment to another) takes five days.

2.3 Marker-based integration

The marker-based integration approach needs to be applied if marker-free integration did not lead to isolation of correct transformants. In that case, the marker-based version of the same integrative construct can be used. First, the assembled overexpression or deletion plasmid needs to be linearized using a restriction enzyme (Table S3 and Table S5) followed by column purification. Then, the concentration of the target fragment needs to be verified, preferably by electrophoresis - a single transformation requires 500 ng. For this, use the standard *Y. lipolytica* protocol for transformation (Protocol 3.8). Transformed cells need to be plated on YNBDcas (Section 4.1) and incubated for two days at 30°C. YNBDcas, is minimal media with glucose supplemented with casamino acids which allows URA+ transformants to grow faster as well as support growth of transformants with detrimental phenotypes that cannot be isolated on media with glucose as the single source of carbon.

Next, to select successful transformants, single colony isolation needs to be applied using the same YNBDcas medium that requiring another two days of incubation. Correct integration of isolated transformants needs to be verified by colony PCR (Section 2.2). Overall, the marker-based integration procedure takes at least five days (from one transformation experiment to another). However, the resulting strain is prototrophic for uracil, so the *URA3* marker needs be removed before the next round of marker-based integration. The suggested marker recovery procedure is provided in Section 2.5.

2.4 Combining marker-based and Cas9-mediated integration

To stimulate homologous recombination, reduce the fraction of transformants with off-target integration and improve the strength of selection, marker-based integration can be combined with CRISPR/Cas9. This approach might be useful for introducing difficult modifications that cannot be isolated using other techniques due to detrimental phenotypes.

Briefly, both DNAs, Cas9-helper and integration construct (donor), need to be prepared and transformed in *Y. lipolytica* as described in Section 2.2, with the only difference that donor fragment needs to include *URA3* marker. After the heat shock and a single washing step, transformed cells need to be incubated overnight in 2 mL of liquid YPD-Nat. The cell culture then needs to be washed with saline solution and plated on YNBDcas in serial dilutions. Transformants need to be verified by colony PCR (Section 2.2) and single colonies need to be isolated on YPD. Elimination of the Cas9-helper can be confirmed by comparing the growth on YPD medium with and without Nat. For this purpose, several colonies (3 to 8) along with the parental strain as control need to be restreaked to single colonies on YPD plates without Nat to promote elimination of the Cas9-helper. After two days, clones need to be picked and resuspended in 25 µL of sterile saline solution. 3 µL should be plated

on each of the plates: YPD and YPD-Nat. Growth needs to be screened after two days. Strains of interest (without Cas9-helper) should grow only on YPD plate without Nat, in the same way the control strain does. Finally, the marker needs to be recovered using procedure described in Section 2.5.

It is noteworthy to mention that selection on minimal media by complementation of uracil auxotrophy does not work well immediately after co-transformation of the Cas9-helper and donor with the *URA3* marker (data not shown). In order to obtain *URA3* functional expression prolonged (*e.g.* overnight) incubation in rich media is required.

2.5 Selectable marker recovery

Lvl0 plasmid pYalC-URA3 contains URA3 flanked by Lox sites - Lox66 and Lox71. The Lox sites enable permanent removal of *URA3* from any derivative construct whether it is present in *Y. lipolytica* or bacteria overexpressing Cre recombinase. In the MEx module, excision in *EcoCre* is used for the isolation of marker-free constructs (Section 1.6). In yeast, episomal Cre-helper plasmid (pCreHA) is used for removing the *URA3* marker from constructs integrated into the *Y. lipolytica* genome. To achieve this, *Y. lipolytica* needs to be transformed with 500 ng of undigested pCreHA. Transformants need to be selected on YPD-Hyg-Urd (hygromycin and uridine). After two or three days of incubation, several colonies need to be restreaked to single colonies on YPD plates without Hyg to promote elimination of the Cre-helper. After another two days, clones need to be picked and resuspended in 25 µL of sterile saline solution. 3 µL should be plated on each of the plates containing different medium compositions (Table S6). Growth needs to be screened after two days. Strains of interest should contain neither *URA3* marker (no growth without Urd) and pCreHA helper (no growth with Hyg). The same suspensions of cells can be used as seeding for next round of integrative transformation. Therefore, single procedure of marker elimination (from Cre-helper transformation to the next transformation experiment) takes at least six days.

Table S6 Identification of *URA3* excision and pCreHA elimination based on phenotypic tests

**Genotype of the strain**		**Observed phenotype of the strain**			
URA3	pCreHA	YNBDcas	YNBDcas with Urd	YPD with Hyg	YPD
+	+	+	+	+	+
+	-	+	+	-	+
-	+	-	+	+	+
-	-	-	+	-	+

In some cases, longer incubation is required to assure both marker and helper have been eliminated. For this purpose, below we describe a two-step procedure to get rid of *URA3* marker first and then the pCreHA helper. That makes the protocol longer, but much more efficient. In this case the 3 transformants selected above on YPD-Hyg-Urd media should be picked into separate tubes with 2 mL of liquid YPD-Hyg-Urd. One loop of the overnight culture from each tube needs to be streaked to single colony on YPD plate. A single isolated colony needs to be picked by toothpick and streaked onto two different plates: YNBDcas first, then using the same toothpick on YNBDCas with Urd. Among each progeny of the 3 independent transformants described above, single clones, which shows growth on YNBDcas with Urd, but does not on YNBDcas, need to be selected. These 3 clones need to be seeded in tubes with 2 mL of liquid YPD. One loop of the overnight culture from each tube needs to be streaked to single colony on YPD plate. A single isolated colony needs to be picked by toothpick and streaked onto two plates: YPD first, then using the same toothpick on YPD-

Hyg. Clones that show no growth on YPD with Hyg are free of both *URA3* marker and pCreHA helper and can be used for the next integration round.

2.6 Promoter library screening

Any promoter assembled on the pProUA-mScarlet vector in the Pro Module (Section 1.8) can be functionally tested by *hrGFP* expression in IntC2 locus. This locus was selected because it allows high expression levels, allowing novel promoters to be compared in the same genetic environment (Fig. S27). Alternatively, any promoter with compatible overhangs on the Lvl0 plasmid (Table S9) can be assembled on the empty pC2US1.1 vector with *hrGFP* gene (pGenC-hrGFP) and *LIP2* terminator (pTerC-LIP2). Both types of assembled plasmids can be used for *in vivo* promoter assay using following procedures.

For integration into the genome, the assembled pProUA-series plasmid needs to be linearized with MssI, purified and co-transformed together with episomal helper pCasNA-IntC2 (Table S8) into the W29ΔuraΔku70 strain (Table S9). All procedures should be performed as has been described for marker-free integration (Section 2.2). Once colonies have been formed on YPD-Nat, large transformants need to be picked and streaked on YNBDcas plate. In our hands, prototrophic clones isolated through such two-step selection procedure always contained marker-based construction into the correct IntC2 locus. The integration can be further confirmed by colony PCR (Protocol 3.9) using either IntC2-chr-F/Barcode1-R or Barcode2-F/IntC2-chr-R pairs of primers (Tables S10).

For promoter activity assay two independent transformants with each promoter need to be grown along with positive control, a strain containing the *TEF1* promoter, and negative control, W29Δku70ura+. Cultures should be grown in 2 mL of liquid YPD medium for 16 hours. The biomass needs to be precipitated, washed once with sterile saline solution and adjusted to OD_{600} 5.0. At this step, if identification of only strongest promoters is required, they can be selected visually using blue LED transillumination (*e.g.* DR46B, Clare Chemical Research) with orange filter (Fig. S28). If large library of promoters is analysed these seeding cultures can be stored at -80 ^{o}C using either 25% (v/v) glycerol or 8% (v/v) dimethylsulfoxide (DMSO) as a cryoprotectant. Note that glycerol induces the strongest catabolic repression in *Y. lipolytica*, therefore DMSO is preferred if the effects of other carbon sources than glycerol need to be studied[14]. Next, depending on the condition required these seedings need to be grown until the appropriate growth phase, following by green fluorescence measurement (*e.g.* excitation, 495 nm; emission, 535 nm) using either plate reader or flow cytometer. Using both controls, promoter strengths can be expressed in the scale where the parental strain W29Δku70ura+ (without GFP) corresponds to 0%, while the transformant with *TEF1* promoter is accepted as 100% of the activity.

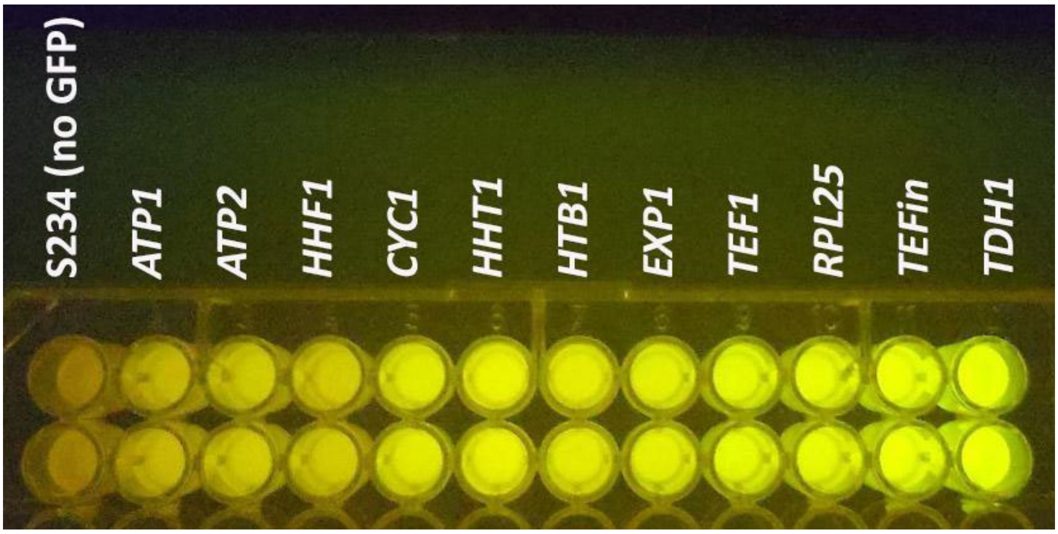

Fig. S28 Visual screening of promoter activities by hrGFP fluorescence in *Y. lipolytica* using pProUA-series. Comparison of different strength promoters is shown along with the parent strain S234 (W29Δku70ura+). Names of *Y. lipolytica* genes are shown that were used as the source of promoter sequences.

3. PROTOCOLS

3.1 GG reaction using BsaI/BsmBI/BpiI/AarI with thermal inactivation

1. Dilute all uncut plasmids to 50 nM.
2. Mix all other components, and then add enzymes as follows:

Component	For 10 µL Reaction
50 nM empty vector plasmid (backbone for assembly)	0.15 µL
50 nM other plasmids	0.5 µL
T4 DNA ligase buffer	1.0 µL
T7 DNA ligase	0.5 µL
Type IIs restriction enzyme (usually supplied as 10 U/µL)	0.5 µL
Nuclease-free water	to 10 µL

3. Place tube in thermocycler and run the following programme:

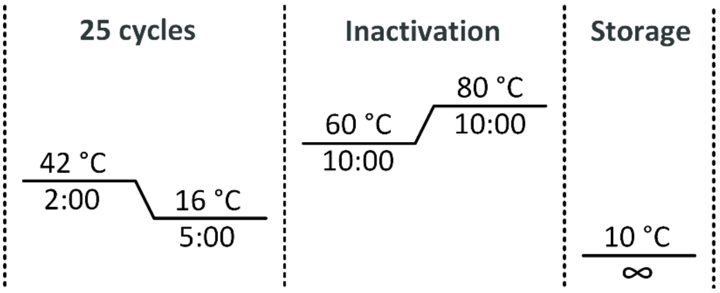

4. Add 20 ml of Nuclease-free water into a Petri dish and float a 0.025 µm MCE membrane (VSWP01300, Millipore) on the surface.
5. Drop 10 µL of GG reaction mixture on the membrane and let it dialyse for 15 min or more.
6. Collect dialyzed GG reaction mixture and electroporate in *E. coli* (Protocol 3.7).

(*for some GG reactions the efficiency of assembly and colony yield can be improved if concentration of plasmid and/or restriction enzyme are proportionally increased up to two times*)

3.2 GG reaction using AarI without thermal inactivation

1. Follow Protocol 3.1 until Step 3.
2. At the Step 3 use alternative thermocycler programme:

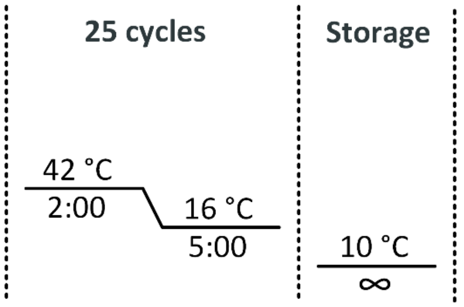

3.3 GG reaction using LguI with thermal inactivation

1. Follow Protocol 3.1 until Step 3.
2. At the Step 3 use alternative thermocycler programme:

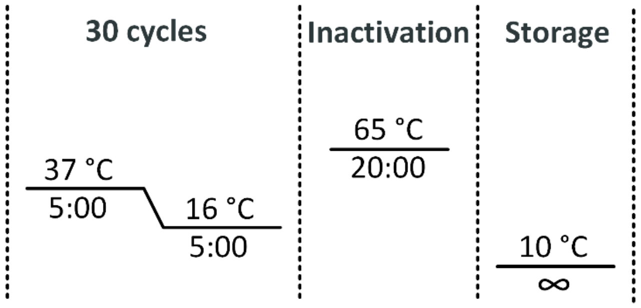

3.4 pDelUK-RG plasmid isolation

1. Seed 5 ml of LB-Km with a colony of transformed *E. coli* containing pDelUK-RG.
2. Use 500 µL of overnight culture to inoculate flask with 100mL of LB medium with Km.
3. Incubate at 37 ^{0}C with shaking (250 rpm) until the culture reaches OD_{600} 0.8
(the general rule for plasmid isolation from culture overexpressing fluorescent protein is collecting the biomass before the time point when bright colour is observed)
4. Collect the cells into a 50-mL tube by centrifuge at 10,000 x g for 6 min and remove the supernatant.
5. Refill the same tube again with leftover culture and repeat Step 4.
6. Add 2.5mL of Buffer P1 (#19051, Qiagen) into the tube and resuspend the cell pellet by vortexing.
(Ensure that RNase A has been added to Buffer P1)
7. Add 2.5mL Buffer P2 (#19052, Qiagen) and mix thoroughly by inverting the tube 4–6 times until the solution becomes viscous and slightly clear.
(Do not vortex, as this will result in shearing of genomic DNA)
8. Add 3.5mL Buffer N3 (#19052, Qiagen) and mix by inverting the tube 4–6 times.
9. Centrifuge for 10 min at 16,000 x *g* and transfer the supernatant to a fresh 50-mL tube with a pipette.
(try not to transfer white pellet and floating flakes)
10. Add 2mL of saturated solution of lithium chloride and vortex.
11. Add 14mL of isopropanol and vortex.
12. Centrifuge for 10 min at 16,000 x *g* and remove supernatant.
13. Spin shortly and remove the rest of the liquid using a pipette.
(Do not dry out the pellet)
14. Resuspend the pellet in small volume of water and transfer to 2-mL tube.
(use as little volume as possible)
15. Add 0.5 volumes of saturated lithium chloride solution and vortex.
16. Leave the tube at -20 ^{0}C for 20 min or longer.
17. Centrifuge the tube for 5 min at 13,000 x *g*.
18. Transfer supernatant by pipette to a new 2-mL tube.
19. Add 2.5 volumes of absolute ethanol and vortex.
20. Centrifuge for 7 min at 13,000 x *g* and remove the supernatant.
21. Spin shortly and remove the rest of the liquid using a pipette.
22. Dry the pellet at 37 ^{0}C for 15 min.
23. Dissolve the pellet in 50 µL of Buffer EB (#19086, Qiagen).
24. Check concentration by gel electrophoresis after restriction digest with NotI or SgrDI.
(usual concentration is 200 ng/µL)

3.5 *EcoRed* competent culture preparation

1. Seed *EcoRed* in a 250-mL flask containing 60mL of LB and grow overnight at 250 rpm and 30 ^{o}C. *(NB: EcoRed does not grow at 37 ^{o}C)*
2. Add 250 mL SOB medium to a 2L conical flask and inoculate with 25 mL of overnight *EcoRed* culture.
3. Grow with shaking (250 rpm, 30 ^{o}C) until the culture reaches an OD_{600} of 0.4-0.5.
4. Quickly transfer flasks into a pre-warmed shaking water bath (*e.g.* C76 NEWBM1248-0003, VWR) and incubate for 15 min at 42 ^{o}C with vigorous shaking.
5. Put flasks into an ice-bath, shake it gently to cool it down, and leave for 10 min. *(hereinafter, all steps should be done in the ice-bath)*
6. Pre-chill a large centrifuge (*e.g.* 5910R, Eppendorf) with an angle rotor and 50 mL tubes to 4 ^{o}C.
7. Centrifuge cells in 50-mL tubes for 10 min at 5,000 x *g* at 4°C and discard supernatant by pouring.
8. Refill the same tubes again and repeat the previous step.
9. Centrifuge tubes with the biomass for another 1 min at 5,000 x *g*.
10. Discard the rest of the supernatant by pipetting.
11. Add 1mL of ice-cold sterile 10% glycerol and gently resuspend the cell pellet by pipetting.
12. Add 30mL of ice-cold 10% glycerol and mix by inverting the tubes several times.
13. Centrifuge for 6 minutes at 5,000 x *g*.
14. Discard the supernatant by pouring.
15. Resuspend cells by pipetting in 500 µL of ice-cold 10% glycerol and transfer the suspension into a pre-chilled 1.5-mL tube.
16. Centrifuge the tube at 4°C in a pre-chilled centrifuge (*e.g.* Micro 17R, Thermo Fisher) for 1 min at 8,000 x *g*.
17. Pipette out the supernatant without disrupting the pellet.
18. Adjust cells to 500 µL by gradually adding 10% glycerol. *(the final volume of competent culture should be 1% of the original culture in SOB medium)*
19. Resuspend cells gently by pipetting.
20. Make 25 µL aliquots of competent *EcoRed* cells in pre-chilled tubes placed on ice.
21. Store the aliquots at -80 ^{o}C until use for electroporation.

3.6 *EcoCre* competent culture preparation

1. Add 60 ml of LB into a 250 ml flask and inoculate with *EcoCre*.
2. Grow overnight at 250 rpm and 37 ^{o}C.
3. Add 250 ml of SOB into two 2L flasks and inoculate each flask with 25 mL of overnight *EcoCre* culture.
4. Grow with shaking at 37 ^{o}C (250 rpm) until culture reaches OD_{600} 0.6.
5. Continue using Protocol 3.5 starting from the Step 5.

3.7 *EcoRed* and *EcoCre* electroporation

1. Take a 25-µL aliquot of competent cells from -80 ^oC and defrost on ice for 10 mins.
2. Cool a 1-mm electroporation cuvette on ice for 10 minutes.
3. Add DNA of interest directly to the tube containing competent cells and mix by pipetting gently.
4. Transfer mixture into the electroporation cuvette placing it between the electrodes.
5. Wipe the sides of the cuvette with a clean paper towel to avoid arcing.
6. Electroporate at 1800V in Eppendorf Eporator or equivalent under the setting recommended by manufacturer for bacteria.
(the time constant should be between 3-6 ms)
7. Immediately after the pulse, add 1 mL of SOB into the cuvette.
8. Mix once only by pipetting and transfer to 1.5-mL tube.
9. Incubate with shaking for 1 hour at 30 ^oC.
10. Plate on LB agar with appropriate antibiotics and grow at 30 ^oC overnight.
(for EcoCre culture different dilutions need to be plated depending on the assembly type. For EcoRed all transformed cells need to be plated)
11. All following cultivation of transformed cells need to be performed at 30 ^oC to prevent induction of recombination proteins.

3.8 *Y. lipolytica* transformation

(adapted from Chen et al.[15])
1. Streak *Y. lipolytica* strain to single colony from glycerol stock onto a YPD plate until single colonies can be seen.
2. Resuspend a single colony in 100 µL of sterile saline solution and spread it on YPD plate to grow overnight.
(if the strain is uracil auxotroph, supplying YPD with Urd)
3. Prepare transformation mixture in a 2-mL tube by combining:

Component	For 100 µL of Transformation Mixture
50% PEG 4000	90 µL
2M lithium acetate (pH 6.0)	5 µL
2M DTT	5 µL

and mix by pipetting.
4. From the overnight plate, pick a small loop of biomass and resuspend it in the transformation mixture.
(this amount of biomass visually correspond to the size of a match head and for the W29 strain it is equal to 1 mL of cell suspension with an OD_{600} of 10)
5. Boil 5 µL of salmon sperm DNA (#15632011, Thermo Fisher) for 8 mins at 95 ^oC, add to the mixture and mix with the loop.
6. Add ≤15 µL of transforming DNA to the mixture and mix with the loop. The preferred concentration of DNA varies with the protocol and has been described elsewhere. When the DNA must be digested or linearized (e.g. NotI), this should have been done before this step.
7. Incubate 30 min at 30 ^oC with moderate shaking.
8. Then heat shock for 30 min at 39 ^oC in a water bath.
9. Dilute the transformation mix with 1 mL of sterile saline solution and gently vortex.
10. Centrifuge the biomass for 2 min at 6,000 x *g*.
11. Remove the supernatant by pipette.
12. Carefully resuspend in 100 µL of saline solution by gently pipetting with a 1000-µL tip.
13. Plate on appropriate media.

3.9 *Y. lipolytica* colony PCR

(this protocol is appropriate for PCR products between 200-900 bp. Amplification of bigger fragments requires isolation of genomic DNA before colony PCR)

1. Pick a single colony of *Y. lipolytica* from freshly grown plate using a pipette tip and resuspend in 25 µL of sterile water.
2. Prepare a 1.1X PCR master mix using 2X Phire Plant Direct PCR Master Mix (#F-160, Thermo Fisher) and both primers with 0.5 µM final concentration.

For example, for 100 µL of PCR prepare 90 µL of 1.1X PCR master mix as follow:

Component	For 90 µL of 1.1X PCR master mix
2X Phire Plant Direct PCR Master Mix	50 µL
100 µM forward primer	0.5 µL
100 µM reverse primer	0.5 µL
Nuclease-free water	39 µL

3. Add 9 µL of 1.1X PCR master mix into a PCR tube.
4. Add 1 µL of cell suspension to the reaction mix for a total reaction volume of 10 µL.
5. Place tubes in a thermocycler and run the following programme:

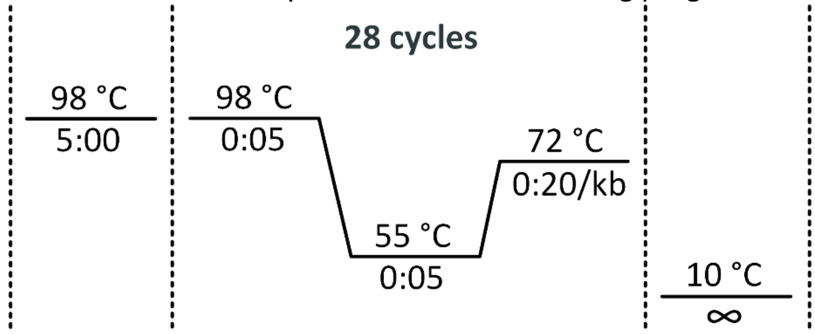

6. Run all of the reaction mixes in a 1% agarose gel.

4. APPENDIX
4.1 Media recipes

Table S7 List of media supplements

Abbreviated name	Full name	Final concentration (μg/mL)
Ap	Ampicillin	150
Km	Kanamycin	100
Cm	Chloramphenicol	10
St	Streptomycin	1000
Sp	Spectinomycin	75
Nat	Nourseothricin	250
Hyg	Hygromycin	450
Urd	Uridine*	300

* – Uridine is preferred to uracil, because of its better solubility.

Saline solution
Prepare 0.85% sodium chloride solution and autoclave.

SOB
1. Mix and autoclave the components below to make SOB(Mg-):
   20 g/L tryptone from casein
   5 g/L yeast extract
   0.6 g/L NaCl (sodium chloride)
   0.2 g/L KCl (potassium chloride)
2. Mix and autoclave the components below to make the Magnesium Mix:
   0.5M $MgCl_2$ (magnesium chloride)
   0.5M $MgSO_4$ (magnesium sulfate)
3. For 1L of SOB(Mg-) from Step 1, add 20mL of Magnesium Mix from step 2 under sterile conditions.

YPD
1. Mix and autoclave the components below:
   20 g/L tryptone from casein
   10 g/L yeast extract
2. Filter sterilize 400 g/L glucose solution.
3. Add glucose to final concentration 20 g/L under sterile conditions.
(other carbon sources can be applied instead of glucose).

YNBDcas
1. Mix and autoclave the components below:
   6.7g/L yeast nitrogen base without amino acids (Y0626, Sigma)
   4g/L casamino acids
2. Filter sterilize 400 g/L glucose solution.
3. Add glucose to final concentration 20 g/L under sterile conditions.
(other carbon sources can be applied instead of glucose).

4.2 List of plasmids

Table S8 List of *YaliCraft* plasmids (*continued on the next page*)

Addgene Number	Module	Plasmid Name	Plasmid Type	*E. coli* Marker	*E. coli* Fluorescence	Used for assembly	*Y. lipolytica* Marker	Application in Yali Module
175709	Lvl0	pYTK001	empty Lvl0	Cm	GFP	Lvl0		
175690	Lvl0	pYalA-Bb	Lvl0, Backbone	Ap	GFP	preLvl		
175711	Lvl0	pYalC-Bb	Lvl0, Backbone	Cm	GFP	preLvl		
175727	Lvl0	pYalK-Bb	Lvl0, Backbone	Km	GFP	preLvl		
175731	Lvl0	pYalS-Bb	Lvl0, Backbone	Sp	GFP	preLvl		
175712	Lvl0	pYalC-URA3	Lvl0, Selectable Marker	Cm		preLvl	URA3	
175710	Lvl0	pYalC-mCherry	Lvl0, *E.coli* RFP	Cm	RFP	preLvl		
175639	Lvl0	pYalC-IntB11Up	Lvl0, Homology Arm	Cm		preLvl		
175640	Lvl0	pYalC-IntB11Dn	Lvl0, Homology Arm	Cm		preLvl		
175715	Lvl0	pYalC-IntC2Up	Lvl0, Homology Arm	Cm		preLvl		
175716	Lvl0	pYalC-IntC2Dn	Lvl0, Homology Arm	Cm		preLvl		
175641	Lvl0	pYalC-IntC13Up	Lvl0, Homology Arm	Cm		preLvl		
175642	Lvl0	pYalC-IntC13Dn	Lvl0, Homology Arm	Cm		preLvl		
175717	Lvl0	pYalC-IntD12Up	Lvl0, Homology Arm	Cm		preLvl		
175718	Lvl0	pYalC-IntD12Dn	Lvl0, Homology Arm	Cm		preLvl		
175643	Lvl0	pYalC-IntE6Up	Lvl0, Homology Arm	Cm		preLvl		
175644	Lvl0	pYalC-IntE6Dn	Lvl0, Homology Arm	Cm		preLvl		
175719	Lvl0	pYalC-IntE8Up	Lvl0, Homology Arm	Cm		preLvl		
175720	Lvl0	pYalC-IntE8Dn	Lvl0, Homology Arm	Cm		preLvl		
175645	Lvl0	pYalC-IntE15Up	Lvl0, Homology Arm	Cm		preLvl		
175646	Lvl0	pYalC-IntE15Dn	Lvl0, Homology Arm	Cm		preLvl		
175647	Lvl0	pYalC-IntF8Up	Lvl0, Homology Arm	Cm		preLvl		
175648	Lvl0	pYalC-IntF8Dn	Lvl0, Homology Arm	Cm		preLvl		
175649	Lvl0	pYalC-IntF11Up	Lvl0, Homology Arm	Cm		preLvl		
175650	Lvl0	pYalC-IntF11Dn	Lvl0, Homology Arm	Cm		preLvl		
175713	Lvl0	pYalC-ZetaUp	Lvl0, Homology Arm	Cm		preLvl		
175714	Lvl0	pYalC-ZetaDn	Lvl0, Homology Arm	Cm		preLvl		
175691	Lvl0	pYalA-sfGFP1.1	Lvl0, *E.coli* GFP	Ap	GFP	empty Lvl1		
175692	Lvl0	pYalA-sfGFP1.2	Lvl0, *E.coli* GFP	Ap	GFP	empty Lvl1		
175693	Lvl0	pYalA-sfGFP1.3	Lvl0, *E.coli* GFP	Ap	GFP	empty Lvl1		
175728	Lvl0	pYalK-sfGFP2.2	Lvl0, *E.coli* GFP	Km	GFP	empty Lvl2		
175729	Lvl0	pYalK-sfGFP2.3	Lvl0, *E.coli* GFP	Km	GFP	empty Lvl2		
175705	Pro	pProUA-mScarlet	empty ProUA-vector	Ap	RFP	Pro-series	URA3	
175721	Lvl0	pProC-TEF1	Lvl0, Pro-series	Cm		Lvl1		
175623	Pro	pProUA-EXP1	ProUA-series	Ap		Lvl1	URA3	
175624	Pro	pProUA-TDH1	ProUA-series	Ap		Lvl1	URA3	
175625	Pro	pProUA-FBA1	ProUA-series	Ap		Lvl1	URA3	
175722	Lvl0	pGenC-hrGFP	Lvl0, Gen-series	Cm		Lvl1		
175723	Lvl0	pTerC-LIP2	Lvl0, Ter-series	Cm		Lvl1		
175724	Lvl0	pTerC-ScADH1	Lvl0, Ter-series	Cm		Lvl1		
175725	Lvl0	pTerC-ScPGK1	Lvl0, Ter-series	Cm		Lvl1		
175726	Lvl0	pTerC-ScENO2	Lvl0, Ter-series	Cm		Lvl1		
175669	Exp	pB8US1.1	empty Lvl1.1	Sp	GFP	Lvl1	URA3	Integration IntB8
175670	Exp	pB8US1.2	empty Lvl1.2	Sp	GFP	Lvl1	URA3	Integration IntB8
175671	Exp	pB8US1.3	empty Lvl1.3	Sp	GFP	Lvl1	URA3	Integration IntB8
175651	Exp	pB11US1.1	empty Lvl1.1	Sp	GFP	Lvl1	URA3	Integration IntB11
175652	Exp	pB11US1.2	empty Lvl1.2	Sp	GFP	Lvl1	URA3	Integration IntB11
175653	Exp	pB11US1.3	empty Lvl1.3	Sp	GFP	Lvl1	URA3	Integration IntB11
175735	Exp	pC2US1.1	empty Lvl1.1	Sp	GFP	Lvl1	URA3	Integration IntC2
175736	Exp	pC2US1.2	empty Lvl1.2	Sp	GFP	Lvl1	URA3	Integration IntC2
175737	Exp	pC2US1.3	empty Lvl1.3	Sp	GFP	Lvl1	URA3	Integration IntC2
175672	Exp	pC7US1.1	empty Lvl1.1	Sp	GFP	Lvl1	URA3	Integration IntC7
175673	Exp	pC7US1.2	empty Lvl1.2	Sp	GFP	Lvl1	URA3	Integration IntC7
175674	Exp	pC7US1.3	empty Lvl1.3	Sp	GFP	Lvl1	URA3	Integration IntC7
175654	Exp	pC13US1.1	empty Lvl1.1	Sp	GFP	Lvl1	URA3	Integration IntC13
175655	Exp	pC13US1.2	empty Lvl1.2	Sp	GFP	Lvl1	URA3	Integration IntC13
175656	Exp	pC13US1.3	empty Lvl1.3	Sp	GFP	Lvl1	URA3	Integration IntC13
175675	Exp	pC14US1.1	empty Lvl1.1	Sp	GFP	Lvl1	URA3	Integration IntC14
175676	Exp	pC14US1.2	empty Lvl1.2	Sp	GFP	Lvl1	URA3	Integration IntC14
175677	Exp	pC14US1.3	empty Lvl1.3	Sp	GFP	Lvl1	URA3	Integration IntC14
175678	Exp	pD6US1.1	empty Lvl1.1	Sp	GFP	Lvl1	URA3	Integration IntD6
175679	Exp	pD6US1.2	empty Lvl1.2	Sp	GFP	Lvl1	URA3	Integration IntD6
175680	Exp	pD6US1.3	empty Lvl1.3	Sp	GFP	Lvl1	URA3	Integration IntD6
175738	Exp	pD12US1.1	empty Lvl1.1	Sp	GFP	Lvl1	URA3	Integration IntD12
175739	Exp	pD12US1.2	empty Lvl1.2	Sp	GFP	Lvl1	URA3	Integration IntD12
175740	Exp	pD12US1.3	empty Lvl1.3	Sp	GFP	Lvl1	URA3	Integration IntD12
175657	Exp	pE6US1.1	empty Lvl1.1	Sp	GFP	Lvl1	URA3	Integration IntE6
175658	Exp	pE6US1.2	empty Lvl1.2	Sp	GFP	Lvl1	URA3	Integration IntE6
175659	Exp	pE6US1.3	empty Lvl1.3	Sp	GFP	Lvl1	URA3	Integration IntE6
175741	Exp	pE8US1.1	empty Lvl1.1	Sp	GFP	Lvl1	URA3	Integration IntE8
175742	Exp	pE8US1.2	empty Lvl1.2	Sp	GFP	Lvl1	URA3	Integration IntE8
175743	Exp	pE8US1.3	empty Lvl1.3	Sp	GFP	Lvl1	URA3	Integration IntE8
175681	Exp	pE12US1.1	empty Lvl1.1	Sp	GFP	Lvl1	URA3	Integration IntE12

Addgene Number	Module	Plasmid Name	Plasmid Type	*E. coli* Marker	*E. coli* Fluorescence	Used for assembly	*Y. lipolytica* Marker	Application in Yali Module
175682	Exp	pE12US1.2	empty Lvl1.2	Sp	GFP	Lvl1	URA3	Integration IntE12
175683	Exp	pE12US1.3	empty Lvl1.3	Sp	GFP	Lvl1	URA3	Integration IntE12
175660	Exp	pE15US1.1	empty Lvl1.1	Sp	GFP	Lvl1	URA3	Integration IntE15
175661	Exp	pE15US1.2	empty Lvl1.2	Sp	GFP	Lvl1	URA3	Integration IntE15
175662	Exp	pE15US1.3	empty Lvl1.3	Sp	GFP	Lvl1	URA3	Integration IntE15
175684	Exp	pE16US1.1	empty Lvl1.1	Sp	GFP	Lvl1	URA3	Integration IntE16
175685	Exp	pE16US1.2	empty Lvl1.2	Sp	GFP	Lvl1	URA3	Integration IntE16
175686	Exp	pE16US1.3	empty Lvl1.3	Sp	GFP	Lvl1	URA3	Integration IntE16
175663	Exp	pF8US1.1	empty Lvl1.1	Sp	GFP	Lvl1	URA3	Integration IntF8
175664	Exp	pF8US1.2	empty Lvl1.2	Sp	GFP	Lvl1	URA3	Integration IntF8
175665	Exp	pF8US1.3	empty Lvl1.3	Sp	GFP	Lvl1	URA3	Integration IntF8
175687	Exp	pF9US1.1	empty Lvl1.1	Sp	GFP	Lvl1	URA3	Integration IntF9
175688	Exp	pF9US1.2	empty Lvl1.2	Sp	GFP	Lvl1	URA3	Integration IntF9
175689	Exp	pF9US1.3	empty Lvl1.3	Sp	GFP	Lvl1	URA3	Integration IntF9
175666	Exp	pF11US1.1	empty Lvl1.1	Sp	GFP	Lvl1	URA3	Integration IntF11
175667	Exp	pF11US1.2	empty Lvl1.2	Sp	GFP	Lvl1	URA3	Integration IntF11
175668	Exp	pF11US1.3	empty Lvl1.3	Sp	GFP	Lvl1	URA3	Integration IntF11
175732	Exp	pZUS1.1	empty Lvl1.1	Sp	GFP	Lvl1	URA3	Integration random
175733	Exp	pZUS1.2	empty Lvl1.2	Sp	GFP	Lvl1	URA3	Integration random
175734	Exp	pZUS1.3	empty Lvl1.3	Sp	GFP	Lvl1	URA3	Integration random
175609	Exp	pB8UA2.2	empty Lvl2.2	Ap	GFP	Lvl2	URA3	Integration IntB8
175610	Exp	pB8UA2.3	empty Lvl2.3	Ap	GFP	Lvl2	URA3	Integration IntB8
175597	Exp	pB11UA2.2	empty Lvl2.2	Ap	GFP	Lvl2	URA3	Integration IntB11
175598	Exp	pB11UA2.3	empty Lvl2.3	Ap	GFP	Lvl2	URA3	Integration IntB11
175696	Exp	pC2UA2.2	empty Lvl2.2	Ap	GFP	Lvl2	URA3	Integration IntC2
175697	Exp	pC2UA2.3	empty Lvl2.3	Ap	GFP	Lvl2	URA3	Integration IntC2
175611	Exp	pC7UA2.2	empty Lvl2.2	Ap	GFP	Lvl2	URA3	Integration IntC7
175612	Exp	pC7UA2.3	empty Lvl2.3	Ap	GFP	Lvl2	URA3	Integration IntC7
175599	Exp	pC13UA2.2	empty Lvl2.2	Ap	GFP	Lvl2	URA3	Integration IntC13
175600	Exp	pC13UA2.3	empty Lvl2.3	Ap	GFP	Lvl2	URA3	Integration IntC13
175613	Exp	pC14UA2.2	empty Lvl2.2	Ap	GFP	Lvl2	URA3	Integration IntC14
175614	Exp	pC14UA2.3	empty Lvl2.3	Ap	GFP	Lvl2	URA3	Integration IntC14
175615	Exp	pD6UA2.2	empty Lvl2.2	Ap	GFP	Lvl2	URA3	Integration IntD6
175616	Exp	pD6UA2.3	empty Lvl2.3	Ap	GFP	Lvl2	URA3	Integration IntD6
175698	Exp	pD12UA2.2	empty Lvl2.2	Ap	GFP	Lvl2	URA3	Integration IntD12
175699	Exp	pD12UA2.3	empty Lvl2.3	Ap	GFP	Lvl2	URA3	Integration IntD12
175601	Exp	pE6UA2.2	empty Lvl2.2	Ap	GFP	Lvl2	URA3	Integration IntE6
175602	Exp	pE6UA2.3	empty Lvl2.3	Ap	GFP	Lvl2	URA3	Integration IntE6
175700	Exp	pE8UA2.2	empty Lvl2.2	Ap	GFP	Lvl2	URA3	Integration IntE8
175701	Exp	pE8UA2.3	empty Lvl2.3	Ap	GFP	Lvl2	URA3	Integration IntE8
175617	Exp	pE12UA2.2	empty Lvl2.2	Ap	GFP	Lvl2	URA3	Integration IntE12
175618	Exp	pE12UA2.3	empty Lvl2.3	Ap	GFP	Lvl2	URA3	Integration IntE12
175603	Exp	pE15UA2.2	empty Lvl2.2	Ap	GFP	Lvl2	URA3	Integration IntE15
175604	Exp	pE15UA2.3	empty Lvl2.3	Ap	GFP	Lvl2	URA3	Integration IntE15
175619	Exp	pE16UA2.2	empty Lvl2.2	Ap	GFP	Lvl2	URA3	Integration IntE16
175620	Exp	pE16UA2.3	empty Lvl2.3	Ap	GFP	Lvl2	URA3	Integration IntE16
175605	Exp	pF8UA2.2	empty Lvl2.2	Ap	GFP	Lvl2	URA3	Integration IntF8
175606	Exp	pF8UA2.3	empty Lvl2.3	Ap	GFP	Lvl2	URA3	Integration IntF8
175621	Exp	pF9UA2.2	empty Lvl2.2	Ap	GFP	Lvl2	URA3	Integration IntF9
175622	Exp	pF9UA2.3	empty Lvl2.3	Ap	GFP	Lvl2	URA3	Integration IntF9
175607	Exp	pF11UA2.2	empty Lvl2.2	Ap	GFP	Lvl2	URA3	Integration IntF11
175608	Exp	pF11UA2.3	empty Lvl2.3	Ap	GFP	Lvl2	URA3	Integration IntF11
175694	Exp	pZUA2.2	empty Lvl2.2	Ap	GFP	Lvl2	URA3	Integration random
175695	Exp	pZUA2.3	empty Lvl2.3	Ap	GFP	Lvl2	URA3	Integration random
175730	Del	pDelUK-RG	empty Del-vector	Km	GFP/RFP	Del-series	URA3	
175706	Del	pDelUA-KU70	Del-series	Ap			URA3	Disruption Ku70
175708	Cas	pCasNA-RK	empty Cas9-helper	Ap Km		Cas-series	Nat	
175632	Cas	pCasNA-IntB8	Cas9-helper	Ap			Nat	Integration IntB8
175626	Cas	pCasNA-IntB11	Cas9-helper	Ap			Nat	Integration IntB11
175702	Cas	pCasNA-IntC2	Cas9-helper	Ap			Nat	Integration IntC2
175633	Cas	pCasNA-IntC7	Cas9-helper	Ap			Nat	Integration IntC7
175627	Cas	pCasNA-IntC13	Cas9-helper	Ap			Nat	Integration IntC13
175634	Cas	pCasNA-IntC14	Cas9-helper	Ap			Nat	Integration IntC14
175635	Cas	pCasNA-IntD6	Cas9-helper	Ap			Nat	Integration IntD6
175703	Cas	pCasNA-IntD12	Cas9-helper	Ap			Nat	Integration IntD12
175628	Cas	pCasNA-IntE6	Cas9-helper	Ap			Nat	Integration IntE6
175704	Cas	pCasNA-IntE8	Cas9-helper	Ap			Nat	Integration IntE8
175636	Cas	pCasNA-IntE12	Cas9-helper	Ap			Nat	Integration IntE12
175629	Cas	pCasNA-IntE15	Cas9-helper	Ap			Nat	Integration IntE15
175637	Cas	pCasNA-IntE16	Cas9-helper	Ap			Nat	Integration IntE16
175630	Cas	pCasNA-IntF8	Cas9-helper	Ap			Nat	Integration IntF8
175638	Cas	pCasNA-IntF9	Cas9-helper	Ap			Nat	Integration IntF9
175631	Cas	pCasNA-IntF11	Cas9-helper	Ap			Nat	Integration IntF11
175707	Yali	pCreHA	Cre-helper	Ap			Hph	Marker recovery

Complete set of 147 *YaliCraft* plasmids may be ordered as two 96 well plates. Deposit 80045 with plasmids #175597-175689 and deposit 80046 with plasmids #175690-175743.

4.3 List of strains

Table S9 List of *YaliCraft* strains

VKPM Number	Strain name	Species (parent wt strain)	Genotype	Growth temperature
**B-14086**	*EcoRed*	*E. coli* (MG1655)	F^- *ilvG*$^-$ *rfb-50 rph-1* λ [*cI*^{857}*Δ(cro-attR)*] *rpsL*^{K43R}	30 °C
**B-14087**	*EcoCre*	*E. coli* (MG1655)	F^- *ilvG*$^-$ *rfb-50 rph-1* λ [*cI*^{857} *Δ(cro-attR) Δ(N-attL)::P1cre)*] *rpsL*^{K43R}	30 °C
**Y-4971**	W29Δura	*Y. lipolytica* (W29)	*Δura3*	30 °C
**Y-4972**	W29Δku70ura+	*Y. lipolytica* (W29)	*Δku70::URA3*	30 °C
**Y-4973**	W29ΔuraΔku70	*Y. lipolytica* (W29)	*Δura3 Δku70*	30 °C

VKPM - Russian National Collection of Industrial Microorganisms.
Modified lambda prophage sequences of both *EcoRed* and *EcoCre* strains are provided in GenBank format.

4.4 List of primers

Table S10 List of standard *YaliCraft* primers (*continued on the next page*)

Number	Name	Sequence	Application
**3158**	sgRNA-seq-R	ATCTGGGCCTCGTGATACG	sgRNA sequencing on pCas-series
**3159**	sgRNA-seq-F	GAGTGGTAAATCGCCTTCTTG	sgRNA sequencing on pCas-series
**3142**	Barcode1-R	ACTATCGGTAGAGCCAATTAG	Left flank checking from any construct
**3143**	Barcode2-F	GAAGTTGGTGAAAACATTTACG	Right flank checking from any construct
**4256**	IntB8-chr-F	TGCAACACACGACAGCTATC	Left flank checking from locus IntB8
**4257**	IntB8-chr-R	AAATCTCTAGCATCTCCAACG	Right flank checking from locus IntB8
**4640**	IntB11-chr-F	ATCGGTTGCAAAACGCTGAC	Left flank checking from locus IntB11
**4641**	IntB11-chr-R	TGACCCTTCGTTACGGACC	Right flank checking from locus IntB11
**3176**	IntC2-chr-F	GTGTGTCCAACCAAAGTGAG	Left flank checking from locus IntC2
**3177**	IntC2-chr-R	GTCAATCACAACAATGTCTCTC	Right flank checking from locus IntC2
**3178**	IntC7-chr-F	ATATCACATTGCAGTCACTCC	Left flank checking from locus IntC7
**3179**	IntC7-chr-R	TACTCCTCTGCGACCCTAC	Right flank checking from locus IntC7
**4642**	IntC13-chr-F	CCATGTGTCTATAAGTACAGC	Left flank checking from locus IntC13
**4643**	IntC13-chr-R	TAACCCTGACAGTTTGATAGC	Right flank checking from locus IntC13
**4250**	IntC14-chr-F	AACTTTCTGAAACTTGCTTTCC	Left flank checking from locus IntC14
**4251**	IntC14-chr-R	AACCAAACACGTGATCAAAGG	Right flank checking from locus IntC14
**5005**	IntD6-chr-F	AGGTGGTCAATGAGTTGTCG	Left flank checking from locus IntD6
**5006**	IntD6-chr-R	TACGTGTGTTTGGCTGTCTC	Right flank checking from locus IntD6
**3164**	IntD12-chr-F	CTGGCAACCACACCGACTC	Left flank checking from locus IntD12
**3165**	IntD12-chr-R	TTTCAGATGTGGACAATTCGG	Right flank checking from locus IntD12
**4454**	IntE6-chr-F	ACTCAACTGTGAATACTTCGG	Left flank checking from locus IntE6
**4455**	IntE6-chr-R	TCCTGGTTAAAGGGGGTTTC	Right flank checking from locus IntE6
**3170**	IntE8-chr-F	TGGGGTGTGTACTGTACTTG	Left flank checking from locus IntE8
**3171**	IntE8-chr-R	ATCAGATCATGATTGGAGAGC	Right flank checking from locus IntE8
**4307**	IntE12-chr-F	CTGGATCTTCCCTCATGGC	Left flank checking from locus IntE12
**4308**	IntE12-chr-R	TCCCCGACGCGAGATTTTC	Right flank checking from locus IntE12
**4456**	IntE15-chr-F	ACCTACCTCCTTCTATCTCC	Left flank checking from locus IntE15
**4457**	IntE15-chr-R	TCTGTAAGCTGAAGCACATTC	Right flank checking from locus IntE15
**4254**	IntE16-chr-F	TCGTGCATTTCGAAACACAAG	Left flank checking from locus IntE16
**4255**	IntE16-chr-R	ATGTAACACGTTCAGACCAGT	Right flank checking from locus IntE16

Number	Name	Sequence	Application
**4850**	IntF8-chr-F	CCAAGAGTTGAATAATGTAACC	Left flank checking from locus IntF8
**4851**	IntF8-chr-R	ACCTAGACTACCGCACCAC	Right flank checking from locus IntF8
**4258**	IntF9-chr-F	TGCCGTCGAGCCACTGTC	Left flank checking from locus IntF9
**4958**	IntF9-chr-R	AGTTACTACAAGTAACCTGAAG	Right flank checking from locus IntF9
**4460**	IntF11-chr-F	ACCTGACTGTTTACATCCGC	Left flank checking from locus IntF11
**4461**	IntF11-chr-R	TCAAGTTCTTGCACTCTGTTG	Right flank checking from locus IntF11

5. REFERENCES

1. Juretzek T, Le Dall M, Mauersberger S, Gaillardin C, Barth G, Nicaud J. Vectors for gene expression and amplification in the yeast *Yarrowia lipolytica*. *Yeast* **18**, 97-113 (2001).
2. Celinska E, Ledesma-Amaro R, Larroude M, Rossignol T, Pauthenier C, Nicaud JM. Golden Gate Assembly system dedicated to complex pathway manipulation in *Yarrowia lipolytica*. *Microbial biotechnology* **10**, 450-455 (2017).
3. Larroude M, Park YK, Soudier P, Kubiak M, Nicaud JM, Rossignol T. A modular Golden Gate toolkit for *Yarrowia lipolytica* synthetic biology. *Microbial biotechnology* **12**, 1249-1259 (2019).
4. He Q, Szczepanska P, Yuzbashev T, Lazar Z, Ledesma-Amaro R. *De novo* production of resveratrol from glycerol by engineering different metabolic pathways in *Yarrowia lipolytica*. *Metabolic engineering communications* **11**, e00146 (2020).
5. Horton RM, Hunt HD, Ho SN, Pullen JK, Pease LR. Engineering hybrid genes without the use of restriction enzymes: gene splicing by overlap extension. *Gene* **77**, 61-68 (1989).
6. Lee ME, DeLoache WC, Cervantes B, Dueber JE. A highly characterized yeast toolkit for modular, multipart assembly. *ACS synthetic biology* **4**, 975-986 (2015).
7. Damude HG*, et al.* High eicosapentaenoic acid producing strains of *Yarrowia lipolytica*. Patet Application US 2006115881 (2006).
8. Nicaud JM*, et al.* Protein expression and secretion in the yeast *Yarrowia lipolytica*. *FEMS yeast research* **2**, 371-379 (2002).
9. Holkenbrink C*, et al.* EasyCloneYALI: CRISPR/Cas9-based synthetic toolbox for engineering of the yeast *Yarrowia lipolytica*. *Biotechnology journal* **13**, e1700543 (2018).
10. Gowers GF*, et al.* Improved betulinic acid biosynthesis using synthetic yeast chromosome recombination and semi-automated rapid LC-MS screening. *Nature communications* **11**, 868 (2020).
11. Generoso WC, Gottardi M, Oreb M, Boles E. Simplified CRISPR-Cas genome editing for *Saccharomyces cerevisiae*. *Journal of microbiological methods* **127**, 203-205 (2016).
12. Horwitz AA*, et al.* Efficient multiplexed integration of synergistic alleles and metabolic pathways in yeasts via CRISPR-Cas. *Cell systems* **1**, 88-96 (2015).
13. Zhang Y*, et al.* A gRNA-tRNA array for CRISPR-Cas9 based rapid multiplexed genome editing in *Saccharomyces cerevisiae*. *Nature communications* **10**, 1053 (2019).
14. Yuzbasheva EY*, et al.* A metabolic engineering strategy for producing free fatty acids by the *Yarrowia lipolytica* yeast based on impairment of glycerol metabolism. *Biotechnology and bioengineering* **115**, 433-443 (2018).
15. Chen DC, Beckerich JM, Gaillardin C. One-step transformation of the dimorphic yeast *Yarrowia lipolytica*. *Applied microbiology and biotechnology* **48**, 232-235 (1997).
